# Supplementary material for: Exposure to duloxetine during pregnancy and risk of congenital malformations and stillbirth: A nationwide cohort study in Denmark and Sweden
Source: PLoS Med. 2021 Nov 22;18(11):e1003851. doi: 10.1371/journal.pmed.1003851 (PMC8654175; doi:10.1371/journal.pmed.1003851)
Supplement: S1 STROBE Checklist — (PDF) [file pmed.1003851.s001.pdf]

S1 Checklist

STROBE Statement—Checklist of items that should be included in reports of ***cohort studies***

Exposure to duloxetine during pregnancy and risk of congenital malformations and stillbirth: A nationwide cohort study in Denmark and Sweden

Authors

Mikkel Zöllner Ankarfeldt, PhD 1

Janne Petersen, PhD, associate professor 1, 2

Jon Trærup Andersen, PhD, associate professor 3, 4

Hu Li, MBBS, PhD 5

Stephen Paul Motsko, PharmD, PhD 5

Thomas Fast, PhD 6

Simone Møller Hede, MSc 6

Espen Jimenez-Solem, PhD, associate professor 1, 3, 4

1 Copenhagen Phase IV Unit (Phase4CPH), Department of Clinical Pharmacology and Center for Clinical Research and Prevention, Copenhagen University Hospital Bispebjerg and Frederiksberg, Copenhagen, Denmark

2 Section of Biostatistics, Department of Public Health, University of Copenhagen, Copenhagen, Denmark

3 Department of Clinical Pharmacology, Copenhagen University Hospital Bispebjerg and Frederiksberg, Copenhagen, Denmark

4 Faculty of Health and Medical Sciences, University of Copenhagen, Copenhagen, Denmark

5 Eli Lilly and Company, Indianapolis, IN, USA

6 Institute of Applied Economics & Health Research, Copenhagen, Denmark

Contact information for the corresponding author
Affiliation:
Copenhagen Phase IV Unit (Phase4CPH), Department of Clinical Pharmacology and Center for Clinical Research and Prevention, Copenhagen University Hospital Bispebjerg and Frederiksberg, Copenhagen, Denmark
Postal address:
Center for Clinical Research and Prevention
Frederiksberg Hospital
Hovedvejen opgang 5.
Nordre Fasanvej 57.
2000 Frederiksberg
DENMARK
Email address // telephone number:
Mikkel.Zoellner.Ankarfeldt@regionh.dk // +45 38163035

	Item No	Recommendation	Page No
**Title and abstract**	1	(*a*) Indicate the study's design with a commonly used term in the title or the abstract	Title page
		(*b*) Provide in the abstract an informative and balanced summary of what was done and what was found	Abstract section
**Introduction**			
Background/rationale	2	Explain the scientific background and rationale for the investigation being reported	Introduction section Paragraph 1 and 2
Objectives	3	State specific objectives, including any prespecified hypotheses	Introduction Paragraph 3
**Methods**			
Study design	4	Present key elements of study design early in the paper	Methods section<br><br>Paragraph 2
Setting	5	Describe the setting, locations, and relevant dates, including periods of recruitment, exposure, follow-up, and data collection	Methods section<br><br>Paragraph 2, and 4 (cohorts)
Participants	6	(*a*) Give the eligibility criteria, and the sources and methods of selection of participants. Describe methods of follow-up	Methods section<br><br>Paragraph 2 and 4 (cohorts)
		(*b*) For matched studies, give matching criteria and number of exposed and unexposed	Methods section<br><br>Paragraph 10 (statistical methods)
Variables	7	Clearly define all outcomes, exposures, predictors, potential confounders, and effect modifiers. Give diagnostic criteria, if applicable	Methods section

			Paragraph 5, 6, 7 (Exposure, comparison groups and outcome)
Data sources/ measurement	8*	For each variable of interest, give sources of data and details of methods of assessment (measurement). Describe comparability of assessment methods if there is more than one group	Partly applicable.<br><br>Methods Section<br><br>Paragraph 2 (describe data sources)<br><br>Paragraph 5, 6 and 7 (describe exposure and outcome measurements)<br><br>Paragraph 11 (describe covariates measurements)
Bias	9	Describe any efforts to address potential sources of bias	Methods section<br><br>Paragraph 1 (prespecified protocol)<br><br>Paragraph 6 (describe comparator used)<br><br>Paragraph 7 (use of international recognized assessment of the malformation outcome)<br><br>Paragraph 9, 10, 11 (confounder issues)<br><br>Paragraph 12 (missing data)<br><br>Paragraph 14 (sensitivity analyses)<br><br>Paragraph 15 (validation of programming)

Study size	10	Explain how the study size was arrived at	Method section<br><br>Paragraph 2 (all registered births and stillbirths)
Quantitative variables	11	Explain how quantitative variables were handled in the analyses. If applicable, describe which groupings were chosen and why	Methods section<br><br>Paragraph 11 (covariates)
Statistical methods	12	(*a*) Describe all statistical methods, including those used to control for confounding	Methods section<br><br>Paragraph 8, 9, 10 (statistical methods)
		(*b*) Describe any methods used to examine subgroups and interactions	N/A
		(*c*) Explain how missing data were addressed	Methods section<br><br>Paragraph 12
		(*d*) If applicable, explain how loss to follow-up was addressed	N/A
		(*e*) Describe any sensitivity analyses	Methods section<br><br>Paragraph 14 (sensitivity analyses)
**Results**			
Participants	13*	(a) Report numbers of individuals at each stage of study—eg numbers potentially eligible, examined for eligibility, confirmed eligible, included in the study, completing follow-up, and analysed<br>(b) Give reasons for non-participation at each stage<br>(c) Consider use of a flow diagram	Figure 1 (Flowchart)
Descriptive data	14*	(a) Give characteristics of study participants (eg demographic, clinical, social) and information on exposures and potential confounders	Results section<br><br>Paragraph 1
		(b) Indicate number of participants with missing data for each variable of interest	Results section<br><br>Paragraph 1
		(c) Summarise follow-up time (eg, average and total amount)	N/A
Outcome data	15*	Report numbers of outcome events or summary measures over time	Methods section<br><br>Paragraph 1

Main results	16	(*a*) Give unadjusted estimates and, if applicable, confounder-adjusted estimates and their precision (eg, 95% confidence interval). Make clear which confounders were adjusted for and why they were included	Result section<br><br>Major malformation: paragraph 2 and figure 2<br><br>Minor malformation: Paragraph 3 and figure 2<br><br>Stillbirth: Paragraph 5 and figure 3.
		(*b*) Report category boundaries when continuous variables were categorized	N/A
		(*c*) If relevant, consider translating estimates of relative risk into absolute risk for a meaningful time period	N/A
Other analyses	17	Report other analyses done—eg analyses of subgroups and interactions, and sensitivity analyses	Result section<br><br>Individual malformation subtypes: Paragraph 4.<br><br>Major malformation sensitivity analyses: Paragraph 2<br><br>Minor malformation sensitivity analyses: Paragraph 3<br><br>Stillbirth sensitivity analyses: Paragraph 5
**Discussion**			
Key results	18	Summarise key results with reference to study objectives	Discussion section<br><br>Paragraph 1
Limitations	19	Discuss limitations of the study, taking into account sources of potential bias or imprecision. Discuss both direction and magnitude of any potential bias	Discussion section<br><br>Paragraph 4-7
Interpretation	20	Give a cautious overall interpretation of results considering objectives, limitations, multiplicity of analyses, results from similar studies, and other relevant evidence	Discussion section<br><br>Paragraph 2-3
Generalisability	21	Discuss the generalisability (external validity) of the study results	Discussion section<br><br>Paragraph 8
**Other information**			
Funding	22	Give the source of funding and the role of the funders for the present study and, if applicable, for the original study on which the present article is based	Acknowledgement and funding section<br>Paragraph 1

*Give information separately for exposed and unexposed groups.

Note: An Explanation and Elaboration article discusses each checklist item and gives methodological background and published examples of transparent reporting. The STROBE checklist is best used in conjunction with this article (freely available on the Web sites of PLoS Medicine at http://www.plosmedicine.org/, Annals of Internal Medicine at http://www.annals.org/, and Epidemiology at http://www.epidem.com/). Information on the STROBE Initiative is available at http://www.strobe-statement.org.
