# Supplementary material for: Exposure to duloxetine during pregnancy and risk of congenital malformations and stillbirth: A nationwide cohort study in Denmark and Sweden
Source: PLoS Med. 2021 Nov 22;18(11):e1003851. doi: 10.1371/journal.pmed.1003851 (PMC8654175; doi:10.1371/journal.pmed.1003851)
Supplement: S1 Protocol — (PDF) [file pmed.1003851.s002.pdf]

Observational Study to Assess Maternal and Fetal Outcomes Following Exposure to Duloxetine: Denmark and Sweden National Pregnancy Registry Study

Eli Lilly and Company
Indianapolis, Indiana USA 46285

Non-interventional PASS Protocol Electronically Signed and Approved by Lilly:
12 October 2017
Non-interventional PASS Protocol Amendment (a) Electronically Signed and Approved by Lilly:

Approval Date: 04-Jun-2018 GMT

LY248686

Post-authorisation Safety Study (PASS) Information

Title	Observational Study to Assess Maternal and Fetal Outcomes Following Exposure to Duloxetine
Version identifier	Draft 1.0
Date of last version	Approval date can be found at the bottom of the preceding page.
EU PAS Register No:	EUPAS20253
Active substance	http://www.whocc.no/atc_ddd_index/ ATC code N06AX21, duloxetine
Medicinal products:	Cymbalta, Ariclaim, Xeristar, Yentreve, Duloxetine Lilly
Product reference:	LY248686
Procedure number:	N/A
Marketing authorisation holder	Eli Lilly and Company
Requested by a regulator	Yes
Joint PASS	No
Research question and objectives	**Primary Objectives**<br>To assess the relative risk of major and minor congenital malformations respectively – comparing first trimester duloxetine exposure to comparators (unexposed to duloxetine, selective serotonin reuptake inhibitor (SSRI)-exposed, venlafaxine/serotonin and norepinephrine reuptake inhibitor (SNRI)-exposed, duloxetine discontinuator).<br>a. Events to be assessed through first year of life among infants.<br>**Secondary Objectives**<br>To assess the risk of non-live birth (spontaneous abortion, elective termination, stillbirth) preterm delivery. Small for Gestational Age – comparing early or late exposure to duloxetine to comparators (unexposed to duloxetine, SSRI-exposed, venlafaxine/SNRI-exposed, duloxetine discontinuator).
Countries of study	Denmark, Sweden
Author	PPD [redacted]

Marketing Authorisation Holder

Marketing authorisation holder (MAH)	Eli Lilly and Company<br>Indianapolis, Indiana USA 46285
MAH contact person	PPD<br><br>Eli Lilly and Company<br>Lilly Corporate Center<br>Phone: PPD<br>Email: PPD<br><br>PPD<br><br>Eli Lilly and Company<br>Lilly Corporate Center<br>Phone: PPD<br>Email: PPD

Revision History

Amendment or update No.	Date	Section of protocol	Amendment or update	Reason
1	21 March 2018	All	Amendment	Exclusion of Finland and Norway

Abbreviation: No. = number.

1. Table of Contents

Section **Page**

Revision History 4
1. Table of Contents 5
2. List of Abbreviations 10
3. Responsible Parties 11
4. Abstract 12
5. Amendments and Updates 14
6. Milestones 15
7. Rationale and Background 16
7.1. Treatment of Depression during Pregnancy 16
7.2. Safety of Antidepressants in Pregnant Women 16
7.2.1. Major Congenital Malformations 17
7.2.2. Preterm Delivery and Small for Gestational Age 17
7.2.3. Spontaneous and Elective Abortions 18
7.2.4. Stillbirths 18
7.3. Duloxetine 18
8. Research Question and Objectives 20
9. Research Methods 21
9.1. Study design 21
9.1.1. Rationale for the Design and Data Source 24
9.2. Setting 25
9.2.1. Study Population 25
9.3. Variables 32
9.3.1. Definition of exposure 32
9.3.2. Definition of Outcome 32
9.3.2.1. Major Congenital Malformation 32
9.3.2.2. Minor Congenital Malformations 32
9.3.2.3. Spontaneous Abortion 32
9.3.2.4. Stillbirth 32
9.3.2.5. Preterm Birth 32
9.3.2.6. Small for Gestational Age 32
9.3.3. Covariates 32
9.4. Data Sources 35
9.4.1. The Health System in Denmark and Sweden 35
9.4.2. Prescription Data 36

9.4.3. The Medical Birth Registers 37
9.4.4. Validity 37
9.4.5. Education and Income Data 39
9.4.6. Quality Assurance and Quality Control 41
9.4.7. Study Time Frame and Lag Time Issues 41
9.5. Study Size 41
9.6. Data Management 42
9.6.1. Data to Be Collected 43
9.6.1.1. Site/Physician Questionnaire 43
9.6.1.2. Patient Data 43
9.6.1.3. Missing Data 43
9.6.1.4. Patient Withdrawal 43
9.6.1.5. Lost to Follow-up Patients 43
9.6.2. File Retention and Archiving 43
9.7. Data Analysis 43
9.7.1. Sensitivity Analyses 45
9.8. Quality Control 45
9.9. Limitations of the Research Methods 45
9.10. Other Aspects 47
10. Protection of Human Subjects 48
11. Management and Reporting of Adverse Events/Adverse Reactions 49
12. Plans for Disseminating and Communicating Study Results 50
13. References 51

List of Tables

Table		Page
Table 1.	Summary of Study Design Including Relevant Time Periods for Mothers and Offspring, Duloxetine Exposure Windows, Outcome Assessment Windows, and Covariate Assessment Windows	23
Table 2.	Risk Factors for Study Outcome Unmeasured or Poorly Measured in the Danish and Swedish Registers	35
Table 3.	Background Information from Danish and Swedish Birth Registries with 2011 as an Example	38
Table 4.	Description of Data Sources and Their Respective Variables of Interest Used in this Study	40
Table 5.	Power to Detect Associations Based on the Number Exposed and Relative Risks	42

List of Figures

Figure		Page
Figure 1.	Flow diagram showing the composition of the study population for the major congenital malformations outcome.	28
Figure 2.	Flow diagram showing the composition of the study population for the SGA and preterm outcomes.	29
Figure 3.	Flow diagram showing the composition of the study population for the perinatal mortality outcomes.	30
Figure 4.	Flow diagram showing the composition of the study population for the spontaneous and elective abortion outcomes.	31

List of Annexes

Annex		Page
Annex 1.	List of Standalone Documents	59
Annex 2.	ENCePP Checklist for Study Protocols	60
Annex 3.	Additional Information	68
Annex 4.	Observational Study Protocol Amendment (a) Summary: Observational Study to Assess Maternal and Fetal Outcomes Following Exposure to Duloxetine: Denmark and Sweden National Pregnancy Registry Study	79

2. List of Abbreviations

Term	Definition
ATC	Anatomical Therapeutic Chemical Classification
BMI	body mass index
CI	confidence interval
ENCePP	European Network of Centres for Pharmacoepidemiology and Pharmacovigilance
ERB	ethical review board
ICD-10	International Classification of Diseases 10th revision
LMP	last menstrual period
OR	odds ratio
OTC	over-the-counter
PASS	Post-Authorisation Safety Study
PS	Propensity Score
RR	relative risk
SGA	Small for Gestational Age
SNRI	serotonin and norepinephrine reuptake inhibitor
SSRI	selective serotonin reuptake inhibitor

3. Responsible Parties

Not applicable.

4. Abstract

- **Title**

  Observational Study to Assess Fetal Outcomes Following Exposure to Duloxetine.

  Version: 1.0

  PPD

- **Rationale and background**

1. There are no published large, controlled randomised clinical trials examining the safety of duloxetine in pregnancy. Given the limitations of spontaneous adverse reports and the sparse published literature, there is currently limited information regarding the safety of duloxetine in pregnancy.

- **Research question and objectives**

  The primary objective is to assess the relative risk (RR) of major and minor congenital malformations overall – comparing first-trimester duloxetine exposure to comparators (unexposed to duloxetine, selective serotonin reuptake inhibitor [SSRI]-exposed, venlafaxine/ Serotonin and Norepinephrine Reuptake Inhibitor [SNRI]-exposed, duloxetine discontinuator). The secondary objective to assess the risk of non-live birth (spontaneous abortion, elective termination, stillbirth), preterm delivery, Small for Gestational Age (SGA), – comparing early or late exposure to duloxetine to comparators (unexposed to duloxetine, SSRI-exposed, venlafaxine/SNRI-exposed, duloxetine discontinuator).

- **Study design**

  Retrospective cohort, comparative observational study

- **Population**

  Pregnant women included in Medical Birth Registries of Denmark and Sweden, or with a diagnosis of spontaneous or elective abortion between 2004 and 2015

- **Variables**

  Exposure to duloxetine is defined by redemption of a prescription for duloxetine. The following information (possible confounders) will be used as covariates in the analyses of the association between duloxetine exposure and the chosen outcomes: maternal demographic characteristics (age, education, income), comedication, and comorbidity. Smoking and body mass index (BMI) depending on the availability in each register.

- **Data sources**

  Medical Birth Registries and National Patient Registries from Denmark and Sweden. Information on abortions will be available from Denmark, and, if national local legislation so permits, from Sweden.

- **Study size**
  Approximately 3000 pregnancies exposed to duloxetine during the first trimester, and 500 to 1000 pregnancies exposed in late pregnancy are projected in the period from 2014 to 2015. The reference groups meeting the study inclusion/exclusion criteria will consist of the following:
  (i). women not exposed to duloxetine during pregnancy,
  (ii). women exposed to SSRI,
  (iii). women exposed to another SNRI, that is, venlafaxine, and
  (iv). women exposed to duloxetine up to 12 months before Last Menstrual Period (LMP), but not during pregnancy.
- **Data analysis**
  Results will be presented for 4 levels of adjustment:
  (i). unadjusted,
  (ii). adjusted for known and available confounders: country, birth year, maternal age, birth order, smoking, comedication, comorbidity, and socioeconomic status, and
  (iii). Propensity Score (PS) matched analysis. In order to address a possible confounding by indication in this study, a PS matched analysis will be performed. To evaluate the association between duloxetine use and specified outcomes, a PS for the likelihood of being exposed to duloxetine by multivariate logistic regression analysis conditional on baseline covariates (country of origin, birth year, maternal age, birth order, smoking, comedication, comorbidity, and socioeconomic status) will be calculated. Each case will be matched to 4 controls in the background population (see reference groups) on the basis of the PS. Stratified analyses for women with and without a diagnosis of depression will be performed.

2. **Milestones:** Analyses are expected to start by 02 September 2018 and will be completed by 26 February 2019.

5. Amendments and Updates

The Amendment summary is provided in Annex 4.

6. Milestones

Milestone	Planned date
Start of data collection	30 May 2018
End of data collection	31 Dec 2018
Registration in the EU PAS register	02 Aug 2017
Final report of study results	28 Mar 2019

Abbreviation: EU PAS = European Union Post-Authorisation Study.

7. Rationale and Background

7.1. Treatment of Depression during Pregnancy

Studies suggest that depression is common during pregnancy and up to 15% of pregnant women suffer from depression or depressive symptoms,[1–4] about 10% develop major depression,[5] and up to 13% are treated with medications.[6–9] Use of antidepressants in pregnant women has grown steadily over time.[6–12] In Denmark, a study reported that between January 1997 and January 2010 the percentage of pregnant women exposed to an antidepressant increased from 0.2% in 1997 to 3.2% in 2009.[9] Selective Serotonin Reuptake Inhibitors are the most commonly used antidepressants worldwide and in Denmark and Sweden,[9,10,13] followed by serotonin and norepinephrine reuptake inhibitors (SNRIs).[9,14]

Treatment of depression with antidepressants during pregnancy is indicated for some women to control their symptoms.[15] Antidepressants have been proven to control mood effectively and reduce risks associated with untreated depression for both the mother and her offspring.[16–18] Untreated mood disorders in the mother may have consequences for both the mother and her offspring.[17,19–21] It is speculated, however, that a significant number of pregnant women are not treated for their depression,[22–24] and around 60% of women using an antidepressant before pregnancy do not continue through the first trimester.[9,14,25]

In addition, depression and anxiety may increase the risk for obstetric complications, puerperal pathologies, and impaired fetal and postnatal development including gestational hypertension and subsequent preeclampsia, bleeding, prematurity, and SGA.[24,26,26–44] However, as most studies did not assess the potential independent effect of medications,[29,42] it remained unclear whether such associations are due to biologic or behavioral factors intrinsic to women with mood disorders, medications used to treat the disorder, or a combination of both. Furthermore, women suffering from depression are more likely to smoke or use alcohol or other substances, which may confound the association between depression and pregnancy outcomes.[43,44]

7.2. Safety of Antidepressants in Pregnant Women

There has been concern about the safety of antidepressant use during pregnancy. The risks of several maternal complications, including preeclampsia, bleeding, and the requirement for a Cesarean section have been reported to be increased among women taking antidepressants during pregnancy.[45] In some studies, first-trimester exposure to certain SSRIs has been associated with some specific birth defects,[46–50] whereas SSRI use late in pregnancy has been associated with pulmonary hypertension of the newborn,[51] prematurity,[51–53] low birth weight,[52,53] SGA,[54] and various neonatal complications.[52,53,55,56] However, other studies have not found these associations.[57–63] Moreover, as most studies did not assess the potential independent effects of medications and depression severity, it has been unclear to what extent such associations are due to biologic or behavioural factors intrinsic to women with mood disorders (such as smoking, substance abuse, or poor diet), medications used to treat the disorder, or a combination of both. It is of note that for some outcomes such as pulmonary hypertension of the newborn, studies

demonstrated that the increased risk initially suggested is modest (odds ratio [OR] 1.51; 95% confidence interval [CI], 1.35 to 1.69) and the absolute risk (0.3%) is small.[64] These small absolute risks increase need to be taken into consideration when evaluating the clinical impact of treatment during pregnancy. Although the RR might be increased, the absolute risk still remains small. Data regarding the safety of SNRIs during pregnancy are sparse. It will therefore be proposed to focus on evaluating the association between maternal use of one specific SNRI, duloxetine, during pregnancy and the risk of the following pregnancy outcomes: major and minor congenital malformations, preterm delivery, SGA, stillbirths, and spontaneous and elective abortions. These outcomes have been associated with other antidepressants, in the literature (see sections below).

7.2.1. Major Congenital Malformations

One of the most concerning adverse effects of medications during pregnancy is teratogenicity. In Denmark and Sweden, approximately 3% of all infants are born with serious birth defects.[63] Deaths due to birth defects is one of the leading causes of infant mortality. Some of the more recent evidence on this topic has clearly demonstrated the impact of confounding by the underlying indication of depression using a variety of different methodological approaches: restriction of the cohort to women with a diagnosis of depression,[65] sibling-controlled analyses,[66] and comparison between pregnancies with exposure to SSRIs during the first trimester versus pregnancies with paused SSRI treatment.[63] Evidence for non-SSRI antidepressants is scarce. In general, studies have found no association between SNRIs and major malformations; but they were based on small exposed cohorts.[67,68] In contrast to the single-action antidepressants, SSRIs, SNRIs (e.g., duloxetine) are dual-action, affecting not only serotonin but also norepinephrine levels in the brain.[69] This different mode of action could be associated with a different safety profile, which calls for further studies.

7.2.2. Preterm Delivery and Small for Gestational Age

These outcomes are leading causes of maternal and/or perinatal mortality and morbidity.[70–72] Low birth weight can be the result of prematurity or of fetal growth retardation or restriction. Prematurity (<37 weeks of gestation) accounts for approximately 10% of all births and is the leading cause of perinatal deaths[70] and long-term disabilities.[70] Infants with growth restriction are born smaller than their peers with the same gestational age at birth. Based on the distribution of birth weights within levels of gestational age, a newborn with a birth weight below the 10th percentile is considered SGA. Infants who are SGA may be term or preterm. Infants who are SGA are also at a greater risk of death and are more likely to develop diabetes, cardiovascular disease, schizophrenia, and other serious conditions.[70,71] Maternal use of SSRIs during pregnancy has been associated with prematurity,[52,53,55,73] low birth weight,[52,53] and SGA.[52,58] However, evidence is conflicting.[74] Both SSRIs and SNRIs affect serotonin levels and can therefore, in theory, be expected to be associated with the same side effects. This might be the reason why some studies have also reported an increased risk of prematurity and SGA in patients treated with non-SSRI antidepressants.[52,55] On the other hand, as previously mentioned, there are concerns about the potential adverse effects of depression itself. Psychological conditions such

as stress, anxiety, and depression may elevate the risk of these outcomes through increased activity of the hypothalamus–pituitary–adrenal axis and release of corticotropin-releasing hormone or other vasoactive hormones and neuroendocrine transmitters.[32,75,76] Whether these risks extend to SNRIs remains unclear.

7.2.3. Spontaneous and Elective Abortions

Since Bassiouni and Rafei showed that women who experienced miscarriage had a higher concentration of serotonin in the blood compared with women giving birth, there has been a great concern regarding treatment with SSRIs, and other antidepressants affecting serotonin levels.[77] Although several studies have investigated the risk of miscarriage in pregnant women undergoing treatment with SSRIs, the results are contradictory[73,78–84] and only a few studies have addressed a potential confounding by indication.[85] Serotonin and norepinephrine reuptake inhibitors have not been studied for a possible association with abortions, and there is a need for studies addressing the issue.

7.2.4. Stillbirths

As mentioned above, studies have identified a possible association between antidepressant exposure and abortions, congenital malformations, and other pregnancy outcomes. Some of these conditions and malformations are potentially fatal in utero, but information on the risk of stillbirth for children has primarily been studied for SSRIs.[86,87] Knowledge on the risks associated with exposure to SNRIs, such as duloxetine, is very limited and needed. Furthermore, large cohorts are needed to assess the risk of this rare outcome, with an incidence of 0.3% to 0.4% in Denmark and Sweden.[88]

7.3. Duloxetine

Duloxetine is a selective SNRI approved in the United States and Europe in 2004. It is currently indicated for the treatment of major depressive disorder, generalised anxiety disorder, stress urinary incontinence, and diabetic peripheral neuropathic pain in Europe. These conditions are common among women of childbearing age.[89] Information from postmarketing surveillance systems suggests a similar pattern of adverse pregnancy outcomes in women using duloxetine during pregnancy compared to the general population.[90–92] These symptoms are characterised by jitteriness, poor muscle tone, weak cry, respiratory distress, hypoglycemia, low Apgar score, and seizures.[93] On the other hand, 2 similar cases reported no signs of withdrawal syndrome.[94,95] One uncontrolled pregnancy registry including 168 live births prenatally exposed to duloxetine reported 3 major malformations (1.8%), which was considered within the expected baseline range in that population.[96] One study based on the Swedish Birth Registry identified 286 live-born infants exposed to duloxetine in the first trimester, 7 were born with malformations (RR, 0.8; 95% CI, 0.32 to 1.64) compared to unexposed.[97]

A recent review concluded that the evidence for duloxetine is limited but does not suggest a clinically important increased risk of major congenital malformations.[98] However, there are no published large controlled studies examining the safety of duloxetine in pregnancy.

A Danish register-based study showed an increased risk of spontaneous abortions associated with use of duloxetine during pregnancy (unadjusted RR, 2.12; 95% CI, 1.52 to 2.96);[84] however, the results were not adjusted for confounders and the sample size was small. Importantly, the statistical analyses did not take time-to-event analysis into consideration, in contrast to other studies analysing the same outcome.[85] Given the limitations of spontaneous adverse reports and the small sample size of the registry, additional information is needed to support conclusions about the safety of duloxetine. Moreover, there is no robustly designed study on the risk of other adverse outcomes such as preterm birth, SGA, or spontaneous and elective abortions.

8. Research Question and Objectives

The objective of this study is to provide a systematic evaluation on the safety of duloxetine in pregnant women. Therefore, the risk of fetal outcomes in relation to duloxetine in a population-based cohort of pregnant women redeeming a prescription for duloxetine, before or during pregnancy, will be quantified. The RR of adverse events in pregnancies exposed during etiologically relevant periods relative to a cohort of women with similar underlying disease, but not treated with duloxetine, will be estimated.

The study objectives are as follows:

To assess the safety of duloxetine for the *developing foetus and the newborn*. Specifically:

Primary Objectives

To assess the RR of major and minor congenital malformations respectively – comparing first-trimester duloxetine exposure to comparators (unexposed to duloxetine, SSRI-exposed, venlafaxine/SNRI-exposed, duloxetine discontinuator). The events will be assessed at birth and through first year of life among infants.

Secondary Objectives

3. To assess the risk of non-live birth (spontaneous abortion, elective termination, stillbirth), preterm delivery, SGA – comparing early or late exposure to duloxetine to comparators (unexposed to duloxetine, SSRI-exposed, venlafaxine/SNRI-exposed, duloxetine discontinuator).

9. Research Methods

9.1. Study design

The study will be a retrospective observational study based on nationwide registers from Denmark and Sweden. All pregnancies, in the 2 countries, ending in induced abortion, spontaneous abortions or birth, and their offspring will be included in the cohort. Due to the birth registries' high completeness, over 99% of all live births and stillbirths will be included in the cohort.[99–102] The study period will be between 2004 and 2015. If more recent data than 2015 are available in the 2 countries, they will be included in the study. Due to the unique personal identification number given to all citizens, it is possible to link the cohort with other registers relevant for the analyses. In the registers, the personal identification number is encrypted, whereby individuals cannot be identified.

Maternal exposure to duloxetine, or other medications, will be defined as redemption of a prescription for duloxetine at a community pharmacy, during the etiologically relevant time period.

The primary study outcome is

- Major and minor malformations

Secondary outcomes are

- Non-live birth: spontaneous abortions, elective termination, and stillbirth
- Preterm delivery
- SGA

Information on these outcomes will be gathered from the national birth registries and national hospital registries, where diagnoses and procedures for inpatients and outpatients are recorded.

Gestational age is recorded in the birth registries and is based on the date of the last menstrual period (LMP) and/or ultrasound estimates.

Four comparison groups have been chosen:

1. Women not exposed to duloxetine during the defined time-period
4. Women exposed to SSRIs
5. Women exposed to another SNRI; venlafaxine
6. Women exposed to duloxetine before, but not during pregnancy, to account for possible confounding by indication

Formal comparisons will only be performed if the sample size meets the requirement.

The main effect measure will be the relative risk (RR or HR) of the outcome associated with duloxetine exposure during the defined time-period.

The defined time period will depend on the chosen outcome.

- For the study of congenital malformations, the period of interest is the first trimester where the foetus' organs are developed.
- For the analyses of preterm delivery and SGA, 2 time periods of interest are defined. The first 20 weeks of gestation (early exposure), and Week 20 to 35 (late exposure). Early exposure is based on a possible risk of impairing placentation. Late exposure is used to analyse exposure closer to the outcome.
- For the analyses of stillbirth, the time period of interest is the whole pregnancy, divided into the first 20 weeks of gestation (early exposure), and Week 20 to delivery (late exposure). Stillbirth is defined as a child showing no signs of life at birth after 22 completed weeks of gestation.
- For the analyses of spontaneous and elective abortions the time period of interest is the first 20 weeks of pregnancy, or until the time of abortion.

Table 1. Summary of Study Design Including Relevant Time Periods for Mothers and Offspring, Duloxetine Exposure Windows, Outcome Assessment Windows, and Covariate Assessment Windows

Outcome	Relevant Time Period – Mother	Relevant Time Period – Offspring	Duloxetine Exposure Window	Outcome Assessment Window	Covariate Assessment Window
Congenital malformations	90 days prior to the LMP to 30 days after delivery	12 months after delivery (unless died)	Redeemed in the first trimester	Delivery to 12 months post-delivery	Comedication: up to one year prior to conception<br>Comorbidity: up to 1 year before conception<br>Education: years of education till the time of conception<br>Household income: the year of conception.
Stillbirth	90 days prior to the LMP to delivery	NA	Redeemed LMP to birth	LMP to birth	Comedication: up to 1 year prior to conception<br>Comorbidity: up to 1 year before conception<br>Education: years of education till the time of conception<br>Household income: the year of conception.
Spontaneous and elective abortions	90 days prior to the LMP to Week 20 or time of abortion	NA	Redeemed from 1 month prior to LMP to date of abortion	LMP to abortion, or Week 20 of gestation	Comedication: up to 1 year prior to conception<br>Comorbidity: up to 1 year before conception<br>Education: years of education till the time of conception<br>Household income: the year of conception.
**Early Exposure**					
Preterm	90 days prior to the LMP to 30 days after delivery	1 month after delivery (unless died)	Redeemed LMP to LMP+140	Delivery to 1 month post-delivery	Comedication: up to 1 year prior to conception<br>Comorbidity: up to 1 year before conception<br>Education: years of education till the time of conception<br>Household income: the year of conception.

Outcome	Relevant Time Period – Mother	Relevant Time Period – Offspring	Duloxetine Exposure Window	Outcome Assessment Window	Covariate Assessment Window
Small for gestational age	90 days prior to the LMP to 30 days after delivery	1 month after delivery (unless died)	Redeemed LMP to LMP+140	Delivery to 1 month post-delivery	Comedication: up to 1 year prior to conception<br>Comorbidity: up to 1 year before conception<br>Education: years of education till the time of conception<br>Household income: the year of conception.
**Late Exposure**					
Preterm	90 days prior to the LMP to 30 days after delivery	1 month after delivery (unless died)	Redeemed LMP+141 to delivery	Delivery to 1 month post-delivery	Comedication: up to 1 year prior to conception<br>Comorbidity: up to 1 year before conception<br>Education: years of education till the time of conception<br>Household income: the year of conception.
Small for gestational age	90 days prior to the LMP to 30 days after delivery	1 month after delivery (unless died)	Redeemed LMP+141 to delivery	Delivery to 1 month post-delivery	Comedication: up to 1 year prior to conception<br>Comorbidity: up to 1 year before conception<br>Education: years of education till the time of conception<br>Household income: the year of conception.

Abbreviations: LMP = last menstrual period; NA = not applicable.

9.1.1. Rationale for the Design and Data Source

Noninterventional, observational studies are a cornerstone in studying the associations between drug exposure during pregnancy and negative birth outcomes. Before authorisation, a medication's efficacy and adverse effects are identified in clinical trials where pregnant women often are excluded. The knowledge of medications' influence on pregnant women and their offspring are therefore almost solely based on postmarketing observational studies after the medication has been on the market for a considerable amount of time. For these studies, health care utilisation databases, such as the national health registers, are often relied on. They provide prospectively collected information for whole nations and allow the study of multiple outcomes. Inclusion of whole nations reduces the risk of selection bias, which gives the studies high

generalisability. Furthermore, the large sizes of these datasets have the potential to generate enough statistical power to examine rare outcomes (e.g., stillbirth) and important subgroups (e.g., duloxetine users).

Although studies emerging from these databases lack the benefits of randomisation, if carefully designed, the results have been shown to be valid and informative, particularly when evaluating unintended drug effects.

The national health registers comprise a unique cohort for the study of pregnant women in Europe, due to the registers' size, quality, and long follow-up time. They have been widely used in observational studies dealing with drugs' possible effect on the offspring. There are some limitations of registers that need to be taken into consideration. These are discussed in Section 9.9.

9.2. Setting

9.2.1. Study Population

The basis for all the analyses will be data from national birth registers of Denmark and Sweden and national patient registries.

1) **Analyses of major congenital malformations**
   a) Inclusion criteria:
      1. Base cohort to include all pregnancies ending in a live birth from the national birth registries of the 2 countries with linked offspring from 2004 to 2015
      2. Information on mother available 12 months prior to the LMP until 1 month post-delivery
      3. Information on offspring available up to 12 months after the delivery
   b) Exclusion criteria:
      1. Pregnancies where information on the mother is unavailable from 12 months prior to the LMP until 1 month post-delivery.
      2. Pregnancies with a chromosomal abnormality based on at least 1 inpatient or outpatient diagnosis of Q87.1, Q87.4, Q9X (International Classification of Diseases 10th revision [ICD-10]) within the first 12 months of the date of birth.
      3. Pregnancies complicated by outpatient exposure to definite teratogens including warfarin, antineoplastic agents, isotretinoin, misoprostol, lithium, and thalidomide from LMP through LMP plus 90 days (i.e., days of exposure overlap with first trimester).

2) **Analyses of preterm delivery and SGA**
   a) This cohort will include all live births with the following inclusion and exclusion criteria:
   b) Inclusion criteria:
      1. Base cohort will include pregnancies drawn from the Danish and Swedish birth registers with linked offspring.

2. Information on mother available 12 months prior to the LMP until 1 month post-delivery.
3. For the outcomes of preterm delivery and SGA, information on the offspring for at least 1 month after the delivery is required, unless the infant died prior to the end of the first month, in which case age at death will be the time of exclusion from the analyses.

c) Exclusion criteria:
1. Pregnancies where information on the mother is unavailable from 12 months prior to the LMP until 1 month post-delivery.
2. Pregnancies for which information on gestational age is missing or implausible.
3. Offspring where information on birth weight is missing.
4. Pregnancies in which duloxetine is dispensed in the 3 months prior to the LMP but not during the first trimester (to ensure that there is not misclassification of the non-exposed), except for the analyses using these duloxetine discontinuers as the reference group.

3) Analyses of stillbirths

a) This cohort will be similar to that used to study major congenital malformations, with the exception of the inclusion of stillbirths (deaths after 22 weeks of gestation).

b) Inclusion criteria:
1. Base cohort to include all pregnancies drawn from the national birth registries of Denmark and Sweden with linked offspring from 2004 to 2015
2. Information on mother available 12 months prior to the LMP until delivery

c) Exclusion criteria:
1. Pregnancies where information on the mother is unavailable from 12 months prior to the LMP until delivery
2. Pregnancies for which information on gestational age is missing or implausible

4) Analyses of spontaneous and elective abortions

a) The study population used for this outcome will highly depend on the availability of data from the 2 countries and their legislation. It is unclear if abortion data from both countries will be accessible.

b) Data from Denmark will be available and has previously been used to estimate risk of abortion among duloxetine-exposed pregnant women.[84] The study only included data from 1997 to 2008. Data are not available from Sweden. The information on abortions are available only on an aggregated level and without unique personal identification number. The Swedish government wants to make it possible to register abortions in the National Patient Register but it has not been decided when this will become effective.

c) Base cohort to include all pregnancies from the national birth registries of the 2 countries with linked offspring and all women with a diagnosis of spontaneous or elective abortion from the national hospital registers, from 2004 to 2015.

d) Inclusion criteria:
   1. Information on mother available 12 months prior to the LMP until 1 month post-delivery/abortion.

e) Exclusion criteria:
   1. Missing information on gestational length or date of abortion

The flowcharts of cohort selection are illustrated in Figure 1, Figure 2, Figure 3, and Figure 4.

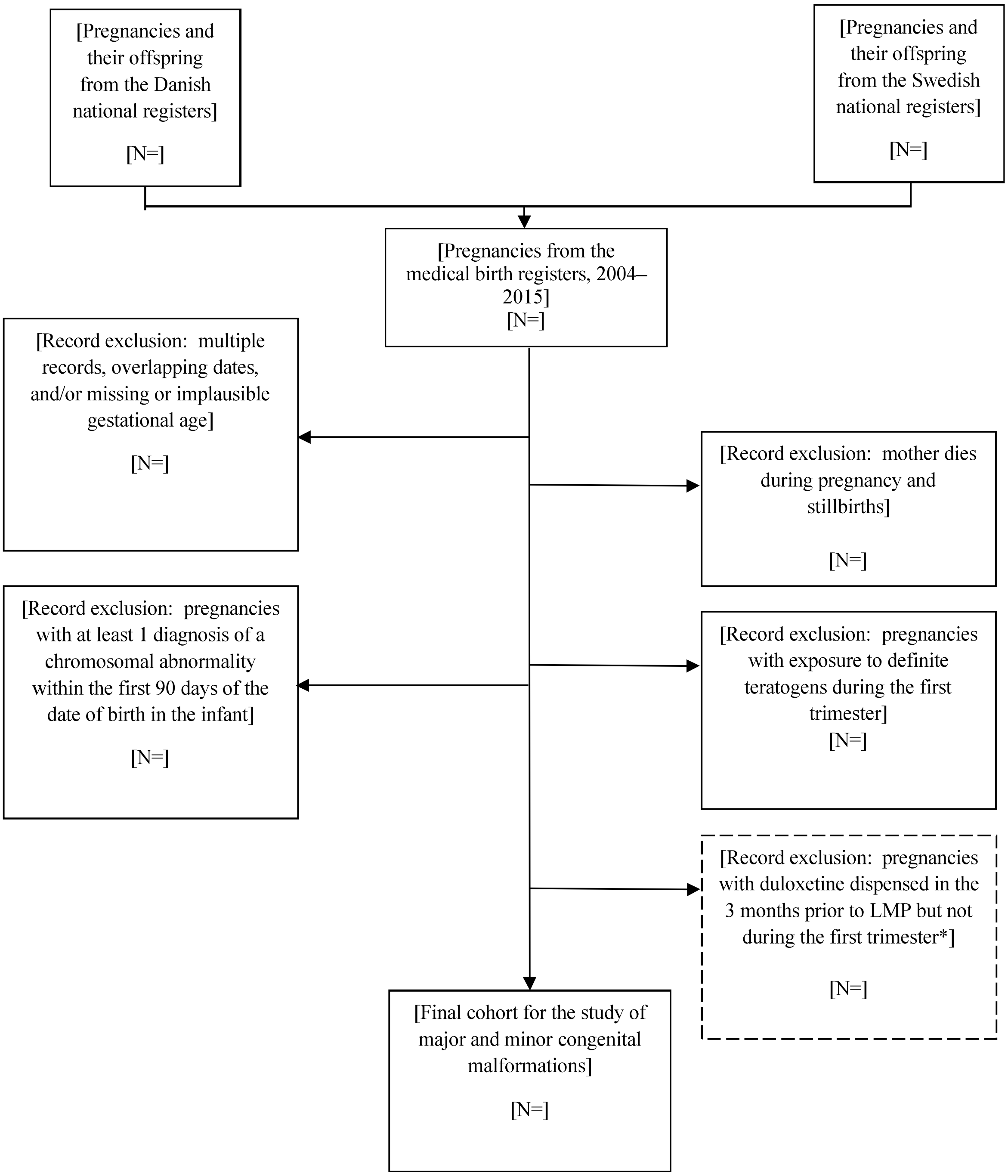

* Except in the analyses using duloxetine discontinuers as the reference group.

Figure 1. **Flow diagram showing the composition of the study population for the major congenital malformations outcome.**

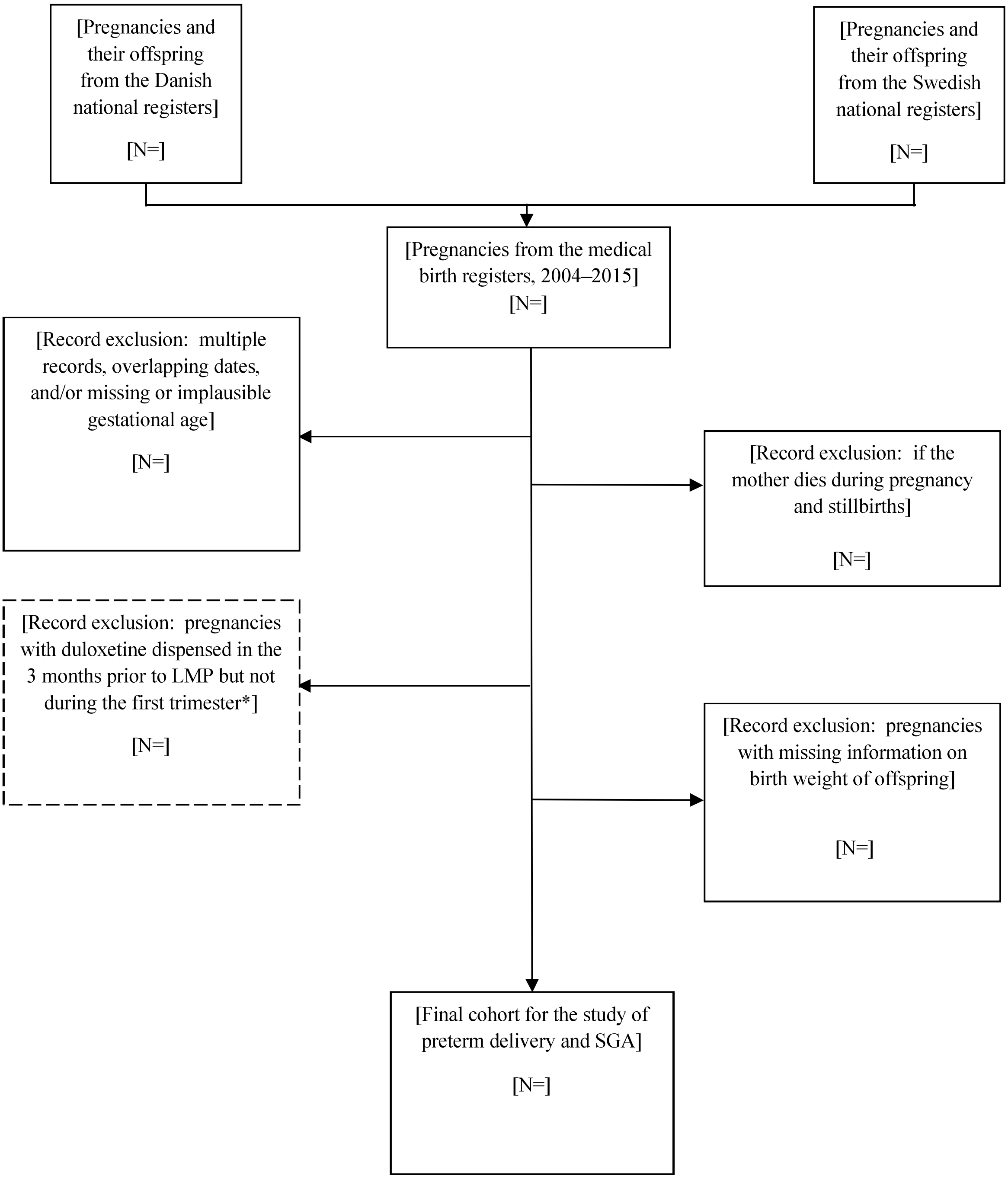

* Except in the analyses using duloxetine discontinuers as the reference group.

Figure 2. Flow diagram showing the composition of the study population for the SGA and preterm outcomes.

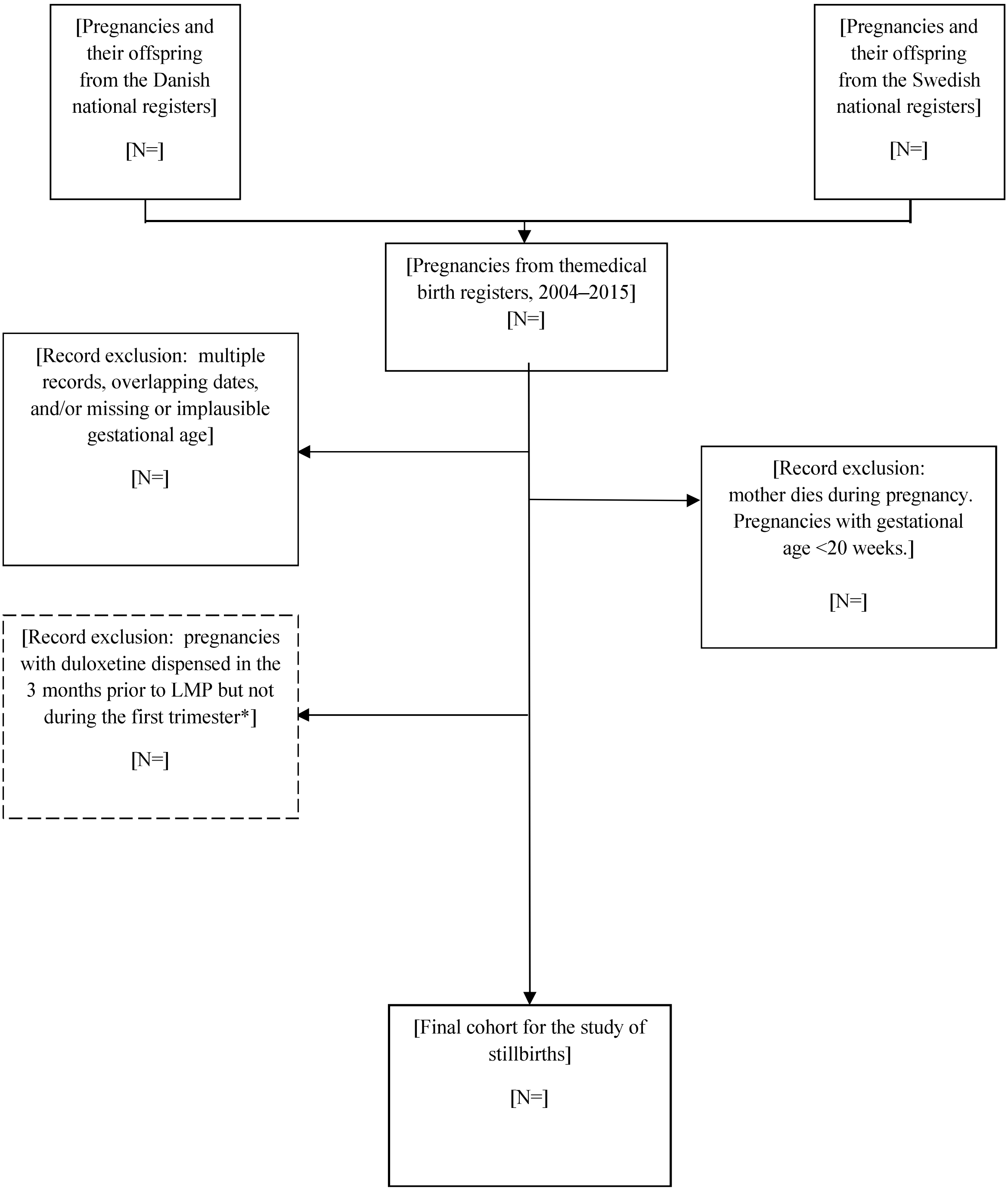

* Except in the analyses using duloxetine discontinuers as the reference group.

Figure 3. Flow diagram showing the composition of the study population for the perinatal mortality outcomes.

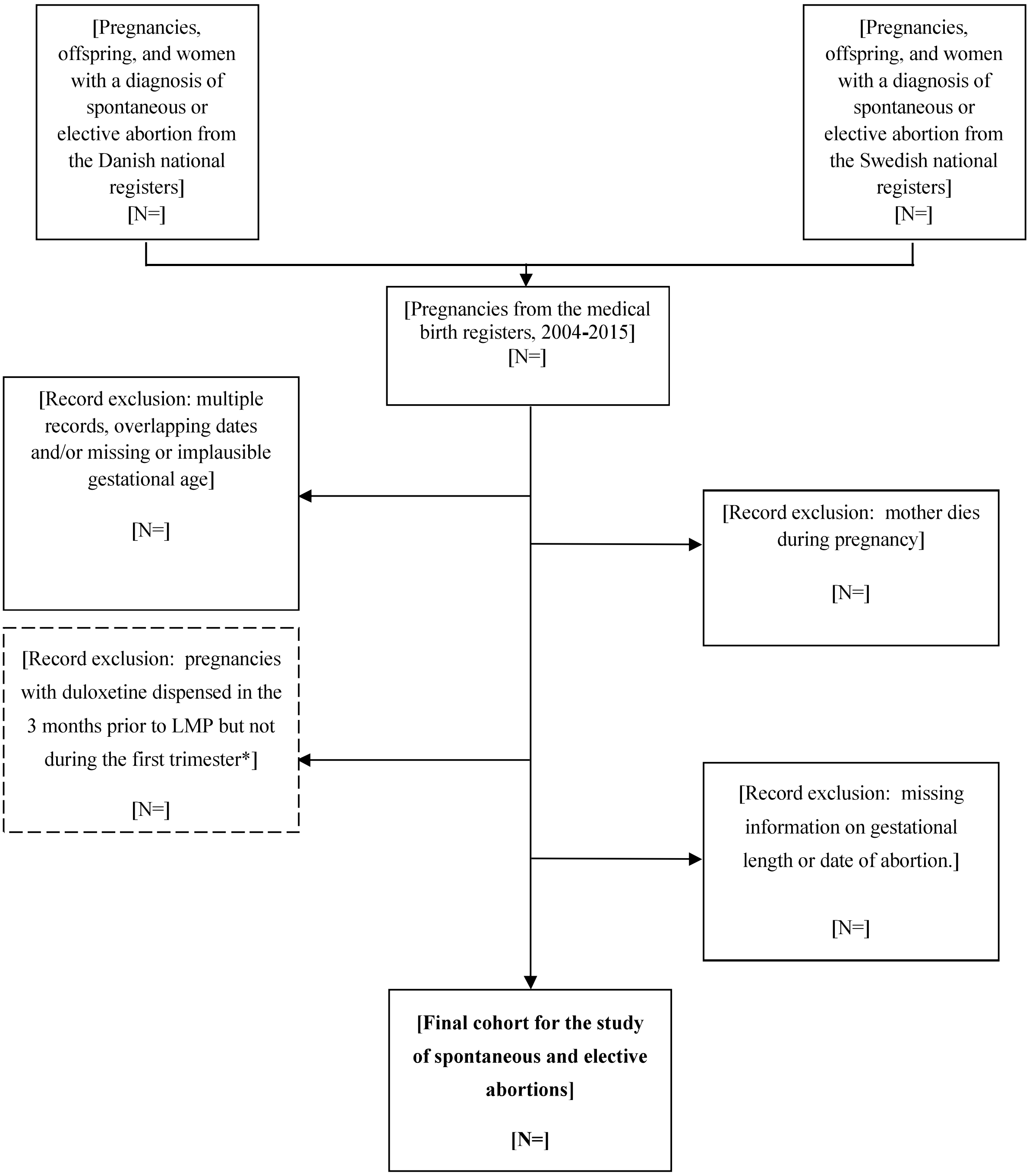

* Except in the analyses using duloxetine discontinuers as the reference group.

Figure 4. Flow diagram showing the composition of the study population for the spontaneous and elective abortion outcomes.

9.3. Variables

9.3.1. Definition of exposure

Exposure is defined as having dispensed at least 1 prescription of duloxetine with the Anatomical Therapeutic Chemical Classification (ATC) code N06AX21 within the given time window.

9.3.2. Definition of Outcome

9.3.2.1. Major Congenital Malformation

Major congenital malformation is defined as a record of an ICD-10 diagnosis from Q00-Q99 according to the EUROCAT classification of major congenital malformations version 1.4 (see Annex 3). All diagnoses within the first year of life or until death are included.

9.3.2.2. Minor Congenital Malformations

The ICD-10 records will be used to define minor malformations according to the EUROCAT classification of major congenital malformations version 1.4 (see Annex 3).

9.3.2.3. Spontaneous Abortion

Spontaneous abortion is defined as a record of an ICD-10 diagnosis:

All registered cases of miscarriage are identified by the following codes: O021 and O03 according to the ICD-10 and all records of induced abortion according to ICD-10 codes O04, O05, and O06. Fetal deaths before 22 completed weeks of gestation is defined as spontaneous abortion.

9.3.2.4. Stillbirth

Stillbirth is defined as a child birth showing no signs of life at birth. Fetal deaths that occur after 22 completed weeks of gestation is defined as stillbirth. The method by which data on perinatal mortality are recorded has been described previously.[103]

9.3.2.5. Preterm Birth

Preterm birth is defined as a live birth before the 37th week of gestation.

9.3.2.6. Small for Gestational Age

Small for gestational age is defined as foetuses with growth restrictions that are born smaller than their peers with the same gestational age at birth. Based on the distribution of birth weights within levels of gestational age, a newborn with a birth weight below the 10th percentile is considered SGA.

9.3.3. Covariates

To account for potential confounders, analyses will be adjusted for the following covariates: country of residence, birth year, maternal age, number of previous spontaneous abortions, birth order, smoking, comedication, comorbidity, and socioeconomic status.

For comorbidity, the following conditions will be considered: hyper- and hypothyroidism ICD-10: E05; hypertension ICD-10: I10-I15; diabetes ICD-10: E10-E14 (diagnosed within 5 years before pregnancy) and O24 (diabetes mellitus during pregnancy); renal failure ICD-10: N17-N19 (diagnosed within 1 year before pregnancy); obesity ICD-10: E66 (diagnosed within 1 year before pregnancy), and so on.

Regarding comedication, the following will be considered: anticonvulsants, antipsychotics, antidepressants, antidiabetics including insulin, antihypertensives, antithyroid preparations, anxiolytics, non-steroidal anti-inflammatory drugs, opioids, thyroid preparations, and so on (Table 1).

The underlying indications for treatment with duloxetine are expected to be important confounders, either due to a direct effect of the conditions or due to lifestyle or other factors associated with the conditions. Indications for therapy will therefore be measured using diagnoses within 1 year before pregnancy and accounted for in the analyses through the use of PS. It will also be attempted to account for the severity of the underlying indications (e.g., depression) through the use of surrogate measures (co-prescribed medications and measures of healthcare use intensity such as the number of psychiatric admissions). Other important potential confounders include chronic comorbid conditions (on the assumption that those with a higher burden of comorbid illness may be more likely to use a SNRI) including diabetes, hypertension, and renal disease. These will be measured directly using diagnosis claims for these conditions. Exposure to medications used as treatments for these conditions (e.g., antihypertensive medications, insulin, oral diabetes medications) as markers of their severity will also be measured. Patient demographic characteristics, if they are associated with treatment and outcome, may also be important confounders and will be accounted for in the analyses.

Therefore, different groups of covariates that could potentially confound the association between duloxetine exposure and the outcomes of interest will be considered. The outcomes of interest are maternal demographic characteristics, comorbid medical conditions, obstetric characteristics/conditions, and maternal medications. The included covariates are selected as they are potential risk factors for the outcomes or potential proxies for such risk factors.

The use of medications up to 1-year period before pregnancy, which may be markers for the presence or the severity of comorbid illness, will be assessed. For the analysis of congenital malformations, the use of suspected teratogenic medications will also be assessed.

A greater disparity in baseline characteristics before adjustment indicates a higher likelihood for unmeasured confounding factors to play a role in the association. Balance in characteristics after adjustment indicates a lower risk of confounding by both measured and unmeasured characteristics. However, unmeasured confounders may still bias the estimate, particularly if not correlated with the measured characteristics.

In Table 2, known or suspected risk factors for the study outcomes that are either unmeasured or poorly measured are presented. These factors are unlikely to be important confounders for the

planned analyses. To bias the results, the risk factor would need to be imbalanced between the duloxetine exposed and unexposed, within the levels of the measured covariates included in the PS. The most concerning potential sources of residual confounding in the planned analyses are smoking status, alcohol use, and drug abuse. The other unmeasured or poorly measured risk factors are not recognised determinants of treatment with a SNRI, making this scenario unlikely. However, to address the potential for residual confounding by these and other factors not accounted for by measured covariates, comparator groups (in addition to a non-exposed comparator) will be used in the analyses. The comparator groups that are more likely to be exchangeable with the duloxetine user group includes women exposed to venlafaxine, SSRIs, and women who discontinue duloxetine prior to pregnancy. Confounding by indication is a term used when a variable is a risk factor for a disease among non-exposed persons and is associated with the exposure of interest in the population from which the cases derive, without being an intermediate step in the causal pathway between the exposure and the disease. If depression is causally linked to any of the outcomes studied, the risk estimate of use of duloxetine in pregnancy (primarily used in the treatment of depression) could thereby be falsely increased. Confounding by indication could be estimated easier by using multiple comparison groups, for example, women suffering from a depression (using different proxies).

Table 2. Risk Factors for Study Outcome Unmeasured or Poorly Measured in the Danish and Swedish Registers

Congenital Malformations	Preterm Birth	Small for Gestational Age
• **Obesity;**<br>• **<u>Infections:</u> Toxoplasmosis; Rubella; Cytomegalovirus; Herpes; Syphilis; Varicella; Parvovirus B19; Zika virus; Lymphocytic choriomeningitis virus (LCMV); Influenza;**<br>• **<u>Physical and environmental agents:</u> Lead; Ionising radiation; Fever/ hyperthermia; Fish consumption-related methylmercury exposure**<br>• **Family history**<br>• **Smoking**<br>• **Alcohol use**	• Life events (divorce, separation, death)<br>• Occupational risk factors<br>• Uterine anomaly, including diethylstilbestrol-induced changes in uterus and leiomyomas<br>• History of second-trimester abortion<br>• History of cervical surgery<br>• Sexually transmitted infections<br>• Bacteriuria<br>• Periodontal disease<br>• Vaginal bleeding, especially in more than one trimester<br>• Previous preterm delivery<br>• Substance abuse<br>• Smoking<br>• Poor nutrition and low body mass index<br>• Low level of educational achievement<br>• Family history of preterm birth, especially maternal first-degree family history of spontaneous preterm birth, particularly if the pregnant woman herself was born preterm<br>• Environmental factors	• Fetal infection<br>• Confined placental mosaicism<br>• Family history<br>• Assisted reproductive technologies<br>• Low prepregnancy weight<br>• Poor gestational weight gain<br>• Malabsorption<br>• Malnutrition<br>• Residing at high altitude<br>• Short interpregnancy interval

9.4. Data Sources

9.4.1. The Health System in Denmark and Sweden

In Denmark and Sweden, about 80% of the funding of the health care comes from public sources. County or regional councils provide most of the health care services. Capitation in combination with service fees is used for all Danish general practitioners, while various fee-for-service systems are used in Sweden.[104]

All Nordic countries had global hospital budgeting in the 1980s; since then, other systems, predominantly combinations of diagnosis-related group financing and global budgets, have been implemented. The amounts of resources devoted to health care are about the same in Denmark and Sweden whether measured by the proportion of gross domestic product devoted to health care or by hospital beds or doctor/patient ratios. In monetary terms, Denmark spends more than

Sweden. Despite similar amounts of resources, they are quite differently used across the countries. Differences of a factor of 2 or more are observed for pharmaceuticals, for example. Despite all these differences, the health care systems are quite similar when seen in a global perspective.

Denmark and Sweden offer excellent opportunities to assess long-term effects to exposure during foetal life. Through the 10- or 11-digit code assigned to each citizen, included in the national registers, it is possible to link information from different registers and thereby follow each individual from the beginning of life until death. The national registers, which are constructed in a similar way and with similar contents, have been used for numerous studies and contributed to important scientific works. Rare exposures and rare outcomes demand very large databases, and as both are small countries with a population ranging from 5.7 million in Denmark to 10 million in Sweden, the data in each country are probably too sparse to evaluate associations between specific drugs and specific malformations or other rare outcomes.

A large dataset can be accomplished by combining information from the health registers in the 2 countries. Women planning a pregnancy and their physicians are entitled to get as reliable information as possible concerning risks with medication that can be used during pregnancy and this can only be achieved through rich data sets combined with high-quality studies.

9.4.2. Prescription Data

Both countries have a nationwide prescription database containing electronically submitted information on prescriptions dispensed by pharmacies. In total, Denmark and Sweden databases cover the countries' 16 million inhabitants. Data from the autonomic regions of the Faroe Islands and Greenland are not included in the Danish data. The data collected are determined by country-specific regulations but all include information on the prescriptions together with information from different administrative registries. Data are transferred electronically monthly from pharmacies to the prescription database. According to the legislation, no informed consent is required for collection of the prescription data, but individuals may see information about themselves if they make an enquiry. When the registry data are used for research purposes, the possible findings cannot be used for decisions concerning individual patients. The national prescription databases in Denmark and Sweden cannot be used for supervision of either individual patients or prescribers. These registers include purchased prescription of drugs that are both reimbursed those that are not.

All individuals/patients included in the prescription databases have a unique personal identifier based on their person identification number, permitting linkage between various population-based data sources. Some prescription databases routinely include the date of death and migration, whereas others need to be linked to this information. With regard to drug exposure, the article number is a unique identifier for each drug formulation of a medicinal product used in the Nordic countries. This number constitutes the link to other registries providing detailed information on dispensed drugs. The drugs are classified according to the global ATC system. Numbers of WHO's defined daily doses dispensed are recorded, as well as the number of packages and the reimbursement code. There are several challenges in using these

data. Firstly, the reimbursement system differs between the 2 countries. Secondly, the indication for the prescription is not yet fully recorded in the databases. The dispensing (redemption) date and retail price are included in all the registries, but the prescription date is at present not included in the Danish prescription databases.

The majority of sales of nonprescription over-the-counter (OTC) medicines are not in the prescription databases. Only OTC medicines prescribed and dispensed to individual patients, for example, for obtaining reimbursement in chronic diseases, are included. The indication for use and the prescribed dose are to some extent included, but only as free text and thus not easily available for research purposes. Patient level data on drug use in hospitals and other institutions are not collected routinely. None of the registries have complete data on vaccines.

9.4.3. The Medical Birth Registers

Both countries have kept medical birth registers for decades, all with compulsory notification. All live births as well as stillbirths from varying gestational ages in the different countries are notified to the registers. All registers contain basic information on the mother, the neonate, and the father. Linkage to other registers and national databases using the personal identity numbers can provide additional data on diseases and medical conditions of the mother, the father, and the neonate, as well as on social conditions, education, prescribed medications, and social security/insurance data. Linkages require study-specific permission from national authorities, which is usually obtained. Thus, it is possible to conduct longitudinal and intergenerational studies and even in some instances include information on relatives and offspring within the period of registration.[103,105]

Diagnoses are registered as ICD-10 codes, increasingly with unlimited numbers. The international origin of the codes for some main groups created through the registers allows for cross-country research on large populations within the countries. However, codes for each individual case are assigned on national platforms and this may involve minor differences between the countries. Birth notification forms are linked to or part of the system and thus to population census offices.

9.4.4. Validity

Systematic validation of data is essential for the credibility of register-based research. Validation of variables for specific studies has been carried out in all registers, but they cover different periods and have only been applied to selected conditions. Overall, these validation studies have found the registers valid with only few missing values.[103,104]

Table 3. Background Information from Danish and Swedish Birth Registries with 2011 as an Example

	Denmark	Sweden
**Number of births/infants (2011)**	58,717/59,666	108,211/10,9766
**Number of obstetric units (2011)**	25	45
**Number of home deliveries, planned/unplanned**	555	Not available
**Missing data: gestational age (%)**	706 (0.1%)	16 (0.01%)
**Missing data: birthweight (%)**	989 (0.2%)	128 (0.12%)
**Missing data: parity (%)**	1172 (0.2%)	0
**Induction of labor**	20.4%	14.0%
**Elective cesaerean section**	9.9%	6.9%
**Episiotomy**	3.4%	5.1%
**In vitro fertilisation**	5.7%	3.7%

The specific outcomes regarding this study have primarily been validated in the Danish registries, probably due to the fact that Denmark was the first country to allow the use of administrative health registries in this research. Several studies have validated the quality of different diagnoses. In general, more than 99% of all hospital contacts are registered in the Danish National Hospital Register, specifically, more than 99% of all births are recorded in the National Danish Medical Birth Registry.~~[106]~~[102] The quality of the malformation diagnoses has been validated and found to have a predictive value of 88% for having a congenital malformation, with a completeness of 90%. Any misclassification of the diagnoses is most probably random.[107] Diagnoses of heart defects have been validated in another study and have been found to have a positive predictive value of 98.4.[108] Furthermore, in Denmark, the diagnosis of spontaneous abortions has been validated and found to have a positive predictive value of 97.4.[109] If women experience a miscarriage without recognising it or do not contact a doctor, the number of

registered miscarriages will be underestimated. This underreporting has been estimated to be 30% and is probably due to miscarriages early in pregnancy.[110] The date of abortion is always included, but the gestational length can be missing. The rate has not been evaluated systematically, but based on experience the number is generally low.

The validity of the information on stillbirths has not been estimated, but as it is statutory by law to register stillbirths, it is believed that the information is of high quality and complete.

There is no reason to believe that the validity of the different outcome variables should have a different level of validity in Sweden.[100]

9.4.5. Education and Income Data

All Nordic countries have high-quality education and income data on each citizen in the country. Registers that all have been used for research purposes several times earlier are based on national statistics on education and tax reports. Not all education is comparable and therefore an adjusted variable on educational length will be made and used as a proxy for level of education. Income data will be indexed by a fixed year and indexed to a currency rate and categorised in quartiles.[106,108,110–117]

Table 4. Description of Data Sources and Their Respective Variables of Interest Used in this Study

Name of register	Description	Variables of interest
**Denmark**		
The Danish National Patient Registry[118]	Information on all patients in contact with a Danish hospital.	Discharge diagnoses and their date.
The Danish National Prescription Registry[119,120]	Contains information on the total redemption of prescriptions in Denmark at community pharmacies since 1994. Data are held by Statistics Denmark.	Date, type, strength, and quantity of drug dispensed.
Medical Birth Registry[121]	Registration is to monitor the health of the newborns and of the quality of the antenatal and delivery care services.	Mothers ' age, parity, body mass index, and smoking. Offspring's time of gestation and conception.
The Danish Civil Registration System[122,123]	Information on all Danish citizens, including date of death, immigration or emigration.	Date of death or emigration
The Danish Education Register[124]	Information on education, level, and length on all people educated in Denmark or immigrated to Denmark.	Education level and length
The Danish register on personal income and transfer payments[~~42~~133]	Information on income and tax payment, earned income, pensions, and benefits.	Household income before tax
**Sweden**		
National Patient Register[125]	Information on all completed in- and out-patient admissions at public hospitals.	Hospital admission and discharge, diagnoses, surgery, including dates. Diagnoses of malformations and comorbidity.
The Swedish Prescribed Drug Registry[126]	Contains information on the total redemption of prescriptions in Sweden since 2005.	Date, type, strength, and quantity of drug dispensed.
Swedish Medical Birth Registry[103]	Contains data on practically all deliveries in Sweden. The register's key data contain information about prenatal care, delivery care, and neonatal care.	Infant diagnoses, smoking, etc.
The Swedish Population Registry[127]	Information on all Swedish citizens, including date of death, immigration or emigration.	Date of death or emigration
The Swedish Register of Education[128]	Information on education, level. and length on all people educated in	Education level and length

Name of register	Description	Variables of interest
	Sweden or immigrated to Sweden.	
The Swedish Income Register[129]	Information on income and tax payment, earned income, pensions, and benefits.	Household income before tax

9.4.6. Quality Assurance and Quality Control

All aspects of data analysis will be conducted according to standard procedures of the Research Group of Drugs in Pregnancy, Copenhagen University Hospital. All statistical- and programming procedures will be conducted by 1 analyst and validated by another. For all data processing and analysis steps, the validation analyst will review the programme along with input and output data sets, and for select steps of the project will employ double programming techniques to reduce the potential for programming errors.

9.4.7. Study Time Frame and Lag Time Issues

Data from the registers for all outcomes including the abortions will cover the period from 2004 to 2015.

9.5. Study Size

According to Statistics Denmark and the Swedish National Board of Health and Welfare, the prevalence of duloxetine exposure is 0.5% (mean prevalence between 2006 and 2015 for Denmark and Sweden), among women of fertile age (20 to 39 years old). Assuming that the prevalence is similar for pregnant women, where approximately 60% do not continue treatment throughout the first trimester, approximately 3000 patients exposed to duloxetine during the first trimester is projected in Sweden and Denmark during the study period. The frequency of exposure decreases during pregnancy such that approximately 500 to 1000 exposed during the "late pregnancy" exposure window is projected. It is estimated that the power to detect significant differences (alpha = 0.05, 2-sided) at various numbers of exposed women and levels of RR for outcomes assuming a prevalence in the unexposed of 15% (e.g., elective abortion),[85] 10% (e.g., preterm delivery, SGA, spontaneous abortions),[85,130] 3% (e.g., major malformations), 1% (e.g., cardiac malformations),[63] 0.5% (e.g., stillbirths), and 0.1% (e.g., rare malformations).[63] The background population are all pregnancies not exposed to duloxetine. Matching will be performed (1:4) for the PS matched analyses. Given that the number of exposed in the cohort will be 3000, the study will have 99% power to detect a RR difference of 1.5 for the primary study outcome, major malformation (see Table 5). The background risk of major malformations is 3%.

Table 5. Power to Detect Associations Based on the Number Exposed and Relative Risks

	RR					RR				
**Exposed**	**1.25**	**1.5**	**2**	**3**	**5**	**1.25**	**1.5**	**2**	**3**	**5**
	RISK IN UNEXPOSED: 10%*					RISK IN UNEXPOSED: 3%**				
150	0.16	0.44	0.90	1.00	1.00	0.08	0.18	0.46	0.87	1.00
300	0.27	0.70	0.99	1.00	1.00	0.12	0.29	0.70	0.99	1.00
450	0.36	0.85	1.00	1.00	1.00	0.15	0.39	0.84	1.00	1.00
600	0.45	0.93	1.00	1.00	1.00	0.18	0.48	0.92	1.00	1.00
750	0.53	0.97	1.00	1.00	1.00	0.20	0.55	0.96	1.00	1.00
900	0.60	0.99	1.00	1.00	1.00	0.23	0.62	0.98	1.00	1.00
1,050	0.67	0.99	1.00	1.00	1.00	0.26	0.68	0.99	1.00	1.00
1,200	0.72	1.00	1.00	1.00	1.00	0.29	0.74	1.00	1.00	1.00
1,350	0.77	1.00	1.00	1.00	1.00	0.32	0.78	1.00	1.00	1.00
1,500	0.81	1.00	1.00	1.00	1.00	0.34	0.82	1.00	1.00	1.00
3,000	0.95	1.00	1.00	1.00	1.00	0.67	0.99	1.00	1.00	1.00
	RISK IN UNEXPOSED: 1%***					RISK IN UNEXPOSED: 0.1%****				
150	0.06	0.10	0.22	0.50	0.87	0.04	0.06	0.09	0.15	0.26
300	0.07	0.14	0.34	0.73	0.99	0.04	0.06	0.11	0.20	0.38
450	0.08	0.18	0.45	0.86	1.00	0.05	0.07	0.13	0.24	0.48
600	0.09	0.22	0.54	0.93	1.00	0.05	0.08	0.14	0.29	0.57
750	0.11	0.25	0.62	0.97	1.00	0.05	0.08	0.16	0.32	0.64
900	0.12	0.29	0.69	0.99	1.00	0.05	0.09	0.17	0.36	0.71
1,050	0.13	0.32	0.74	0.99	1.00	0.05	0.09	0.18	0.40	0.76
1,200	0.14	0.35	0.79	1.00	1.00	0.06	0.10	0.20	0.43	0.80
1,350	0.15	0.38	0.83	1.00	1.00	0.06	0.10	0.21	0.46	0.84
1,500	0.15	0.41	0.86	1.00	1.00	0.06	0.10	0.22	0.49	0.87
3,000	0,28	0.78	1.00	1.00	1.00	0.06	0.13	0.41	0.93	1.00

* This is based on an assumption of 10% risk among unexposed.
** This is based on an assumption of 3% risk among unexposed.
*** This is based on an assumption of 1% risk among unexposed.
**** This is based on an assumption of 0.1% risk among unexposed.

9.6. Data Management

All data management and statistical analysis will be performed using SAS version 9.4. Data will be managed and stored as required by relevant national laws and regulations. All analyses will be performed on the basis of pseudoanonymised data and performed at a secure server system for each country separately. All statistical analyses will be performed by 2 separate investigators to ensure robustness of the findings.

In Denmark, the Act on Processing of Personal Data does not require ethical permission or obtained consent for anonymised retrospective register studies. Approval from the respective National Data Protection Agencies in the 2 countries will be required before the start of the study.

Data from the 2 countries will be gathered at Statistics Denmark, as Denmark is the only country not allowing data to cross their border. Statistics Denmark is a secure facility that hosts a state-of the-art, secure computing environment. Access to data will be through a secure and encrypted virtual private network connection. Individual access requires clearance by Statistics Denmark.

9.6.1. Data to Be Collected

9.6.1.1. Site/Physician Questionnaire

Not relevant.

9.6.1.2. Patient Data

Data will be collected from the national registers as described under Section 9.4.

9.6.1.3. Missing Data

In general, the completeness of data is very high and the number of missing data low. The majority of adjustment variables have less than 1% missing values although smoking and information on maternal weight have up to 5 % missing values. To control for missing data, a single imputation method using the Mode substitution method will be used.

9.6.1.4. Patient Withdrawal

Not relevant.

9.6.1.5. Lost to Follow-up Patients

The maximum follow-up time is 1 year from birth. Only if a child dies or emigrates from the country it is lost to follow-up. It is extremely rare for a family to emigrate within the first year after birth. Thus, lost to follow-up should not be a practical issue.

9.6.2. File Retention and Archiving

Datasets and analytic programmes will be kept on a secure server and archived per Lilly record retention procedures. If the study is being conducted by a third party, the datasets and analytic programmes will be stored according to the vendor's procedures.

9.7. Data Analysis

The analytic approach will be the same for all the outcomes, except spontaneous and elective abortions.

The reference groups will consist of:

a) women not exposed to duloxetine during the relevant exposure window;
b) women exposed to SSRI;
c) women exposed to venlafaxine; and

d) women exposed to duloxetine up to 12 months before LMP, but not during pregnancy.

Differences in basic characteristics for the duloxetine-exposed and reference group will be compared. Focus will be on country of origin, birth year, maternal age, birth order, smoking, comedication, comorbidity, and socioeconomic status. Baseline characteristics will be compared with chi-square tests for categorical variables. For the chosen outcome, absolute risks and unadjusted RRs with their 95% CI will be calculated.

Results will be presented for 3 levels of adjustment:
Primary comparison:

1. PS matched analysis. In order to address possible confoundings in this study, a PS for the likelihood of being exposed to duloxetine by multivariable logistic regression analysis conditional on baseline covariates (country of origin, birth year, maternal age, birth order, smoking, socioeconomic status, comedication, comorbidity, and duloxetine indications [i.e., diagnosis of depression, anxiety, stress urinary incontinence, and diabetic peripheral neuropathic]) will be calculated. The analysis is based on Caliper matching with a maximum tolerated difference between matched subjects in a “non-perfect” matching intention at 0.2 standard deviation as the default. Each case will be matched to 4 controls in the background population (see reference groups) on the basis of the PS using a Greedy matching algorithm. Both a graphical balance check and statistical test will be used to check the balance of the PS model.

Secondary comparison:

2. unadjusted,
3. adjusted for known and available confounders: country, birth year, maternal age, birth order, smoking, comedication, comorbidity, and socioeconomic status

For all outcomes, except abortions, a linear logistic regression model will be used to estimate the OR for the dichotomous outcome.

For induced and spontaneous abortions, a Cox proportional hazard model with gestational age as the underlying time scale and with further adjustment for the above-mentioned covariates will be used. Cox proportional hazard regression models with exposure to duloxetine as a time-dependent variable and time from conception to miscarriage as outcome. Time to birth or induced abortion will be considered as censoring variables. Prescriptions redeemed after miscarriage or censoring will not be included in the analyses. Estimates will be presented as hazard ratios with 95% CI. A Cox proportional hazard model is used to take time to outcome into account and will give the best risk estimation. Due to lack of availability, data from Denmark will be used to estimate the risk of miscarriage. Information on spontaneous abortions from Sweden will be gathered in accordance with national law.

9.7.1. Sensitivity Analyses

To test the robustness of the findings, sensitivity analyses may be performed. The overall findings will be interpreted in the light of the results of prespecified sensitivity analyses:

- Re-definition of exposure to having redeemed >1 prescription for duloxetine during the relevant time window.
- Redefinition of exposure to cover days' supply that overlaps with the relevant time window. The exposure will be calculated based on the number of redeemed pills and their strength compared to the WHO's daily defined dose.
- Restriction of the cohort to the first pregnancy occurring within the study period.
- Inclusion of BMI in the statistical model as covariate for pregnancies where information on BMI is available.

9.8. Quality Control

All data gathering and analyses will be overseen by 2 pharmacoepidemiologists experienced in the field of drug exposure during pregnancy. Programming for this project will be conducted by a primary analyst and validated by a separate analyst (validation analyst). For all data processing steps, the validation analyst will review the programme along with input and output data sets. For the analysis steps of the project, double programming techniques to reduce the potential for programming errors will be employed.

9.9. Limitations of the Research Methods

The national health registers are unique due to their completeness and follow-up time. They are recognised internationally, and widely used for epidemiological studies of a wide variety of medical issues. Data are gathered prospectively, but analyses are made retrospectively. The main limitation is therefore centered around the potential for misclassification.

1. Exposure misclassification: exposure is defined by the redemption of a prescription. Although the medication has been prescribed, dispensed, redeemed, and paid for, there is a probability that the patient has not ingested the drug. In sensitivity analyses, a stricter definition will be used and it is required that women have to fill in >1 prescription, under the assumption that filling multiple prescriptions increases the likelihood that the medication is being taken as prescribed. There is no risk of recall bias given that data are not based on interviews and the prescription registers include more than 98% of all redeemed prescription at community pharmacies.[131] There is no risk of false negatives given that duloxetine is not available OTC.
2. Outcome misclassification: the outcomes chosen for this study have been used in multiple previously published peer-reviewed studies.[66,85,87,~~132,133~~130]The outcomes have a high positive predictive value and is regarded as having a high validity. Regardless of this, some potential for outcome misclassification remains.
   - Using registers to analyse the risk of abortion, like in this study, does not allow for identification of the earliest abortions unrecognised by the women because they

are not recorded in the registers. Hypothetically, the risk of the outcome will be underestimated if the exposure (i.e., duloxetine) specifically is associated with these early abortions. If women experiencing an abortion do not chose to contact a hospital, the number of registered abortions would be underestimated. Studies have shown that the underreporting is 25% and is probably the result of abortions in the early pregnancy.~~[116]~~[132]If women with exposure during pregnancy are more likely to report an abortion than unexposed, it could lead to a false increased risk of the outcome.

3. Unmeasured confounders: information on potentially confounding lifestyle factors such as alcohol and drug abuse/dependence is not available. Information on smoking, and to a certain extent BMI, is available. It is believed that by adjusting for socioeconomic status and smoking, there will also be adjustments for the missing confounders due their close association. Other important unmeasured confounders are the indication for treatment with an antidepressant, and the severity of depression, which can lead to confounding by indication. In addition, there can be other health conditions, not available through the registers, related to patients treated with duloxetine. Having several comparison groups of women using different types of antidepressants and a comparison group of discontinuers will help in the attempt to detangle the effect of medications from the underlying maternal illness.
4. OTC medication and illicit drugs: information is not recorded on an individual basis in the available registers, if they are not prescribed. OTC medications are however not expected to be strong confounders, and missing information on OTC medications is expected to have very limited impact on the study. Illicit drug use is not believed to be a challenge in the studied population.
5. Breastfeeding: the available register does not have reliable information on breastfeeding.
6. Low statistical power: some of the outcomes in this study are rare (e.g., neonatal mortality, specific congenital malformations). Although the size of the cohort is considerable, there might be limited statistical power to detect small increases in risk. These small increases might, however, not be clinically relevant due to the small absolute numbers.
7. Completeness of depression diagnosis: patient diagnosis are only recorded in the national registers if the patient has had contact with a hospital. Depression (especially mild and moderate) is most often treated in the primary sector, without contact to the secondary sector (i.e., hospitals). It is therefore probable that most patients redeeming a prescription for duloxetine, or any other antidepressant, do not have a recorded diagnosis, and the indication for their treatment is therefore an assumption. This also applies to other diagnoses mainly treated in the primary sector, for example, mild infections, migraine, mild/moderate pain, and so on.

As the national health registers from Denmark and Sweden cover the whole nations, there is minimal risk of selection bias. Therefore, the findings from this study should be generalizable,

as the limitations in this study are not expected to affect the biologic relations studied. It is important, however, to acknowledge that selection of the comparable cohort may influence the internal validity of the study. The premise of generalisability depends therefore on a well-designed comparable cohort.

9.10. Other Aspects

Not applicable.

10. Protection of Human Subjects

Observational studies will be submitted to ethical review boards (ERBs) for approval whenever required by local law. Regulatory authorities will be notified and approval sought as required by local laws and regulations. Progress reports will be submitted to ERBs and regulatory authorities as required by local laws and regulations.

This study will be conducted in accordance with applicable laws and regulations of the region, country, or countries where the study is being conducted, as appropriate.

The identity of human subject included in the study will not be traceable, as the personal identification number will be encrypted. Furthermore, no aggregated data including less than 3 cases will be presented in the results. It is believed that these measures ensure the anonymity of the patients making up the cohort.

11. Management and Reporting of Adverse Events/Adverse Reactions

During the course of secondary use of data in observational research, information pertaining to adverse reactions will not be discovered because the study does not involve identifiable patient data associated with a Lilly product. Data in this study are being analysed in aggregate only, study data sets do not include safety measures, and there will be no medical chart review or review of free text data fields.

12. Plans for Disseminating and Communicating Study Results

The final report will be shared with the US Food and Drug Administration and European Medicines Agency. Manuscripts describing this work will be submitted for publication in peer review journals. Findings may also be submitted for presentation at scientific conferences. Results will be disclosed on the European Network of Centres for Pharmacoepidemiology and Pharmacovigilance (ENCePP) EU PAS register.

13. References

1. Evans, J., Heron, J., Francomb, H., Oke, S. & Golding, J. Cohort study of depressed mood during pregnancy and after childbirth. *BMJ* 2001, 257–60.
2. Gotlib, I. H., Whiffen, V. E., Mount, J. H., Milne, K. & Cordy, N. I. Prevalence rates and demographic characteristics associated with depression in pregnancy and the postpartum. *J Consult Clin Psychol* 1989, 269–74.
3. Bennett, H. A., Einarson, A., Taddio, A., Koren, G. & Einarson, T. R. Prevalence of depression during pregnancy: systematic review 1. *Obstet.Gynecol.* 103, 698–709 (2004).
4. Chatillon, O. & Even, C. [Antepartum depression: Prevalence, diagnosis and treatment. *Encephale* 36, 443–451 (2010).
5. Kendell, R. E., Wainwright, S., Hailey, A. & Shannon, B. The influence of childbirth on psychiatric morbidity. *Psychol. Med.* 1976, 297–302.
6. Reefhuis, J., Rasmussen, S. A. & Friedman, J. M. Selective serotonin-reuptake inhibitors and persistent pulmonary hypertension of the newborn. *N Engl J Med* 354, 2188–2190 (2006).
7. Cooper, W. O., Willy, M. E., Pont, S. J. & Ray, W. A. Increasing use of antidepressants in pregnancy. *AmJ ObstetGynecol* 196, 544–545 (2007).
8. Andrade, S. E. *et al.* Use of antidepressant medications during pregnancy: a multisite study. *AmJ ObstetGynecol* 198, 194–195 (2008).
9. Jimenez-Solem, E. *et al.* Prevalence of antidepressant use during pregnancy in Denmark, a nation-wide cohort study. *PloS One* 8, e63034 (2013).
10. Kaufman, D. W., Kelly, J. P., Rosenberg, L., Anderson, T. E. & Mitchell, A. A. Recent patterns of medication use in the ambulatory adult population of the United States. The Slone Survey. *J. Am. Med. Assoc.* 2002, 337–44.
11. Riley, Fuentes-Afflick, Jackson, Escobar, Brawarsky, Schreiber, Haas. Correlates of prescription drug use during pregnancy. *J Womens Health* 2005, 401–9.
12. Mitchell, A. A., Gilboa, S. M., Werler, M. M. & Kelley, K. E. Louik C, Hernandez-Diaz S. Medication use during pregnancy, with particular focus on prescription drugs: 1976-2008. *Am J Obstet Gynecol* 2011, 1–8.
13. Andrade, S. E. *et al.* Prescription drug use in pregnancy. *Am J Obstet Gynecol* 191, 398–407 (2004).
14. Huybrechts, K. F., Palmsten, K. & Mogun, H. National trends in antidepressant medication treatment among publicly insured pregnant women. *Gen. Hosp. Psychiatry* 2013, 265–71.
15. Moses-Kolko, E. L. & Roth, E. K. Antepartum and postpartum depression: healthy mom, healthy baby. *J Am Med Womens Assoc* 2004, 181–91.
16. Cohen, L. S. *et al.* Relapse of major depression during pregnancy in women who maintain or discontinue antidepressant treatment. *JAMA* 295, 499–507 (2006).
17. Altshuler, L. L., Cohen, L. S. & Moline, M. L. Treatment of depression in women: a summary of the expert consensus guidelines. *J. Psychiatr. Pract.* 2001, 185–208.
18. Bonari, L. *et al.* Perinatal risks of untreated depression during pregnancy. *Can.J.Psychiatry* 49, 726–735 (2004).
19. Frank, E., Kupfer, D. J. & Perel, J. M. Three-year outcomes for maintenance therapies in recurrent depression. *Arch Gen Psychiatry* 1990, 1093–9.
20. O'Hara, M. W., Neunaber, D. J. & Zekoski, E. M. Prospective study of postpartum depression: prevalence, course, and predictive factors. *J. Abnorm. Psychol.* 1984, 158–71.
21. Hirschfeld, R. M., Keller, M. B. & Panico, S. The National Depressive and Manic-Depressive Association consensus statement on the undertreatment of depression. *Jama* 1997, 333–40.

22. Flynn, H. A., Blow, F. C. & Marcus, S. M. Rates and predictors of depression treatment among pregnant women in hospital-affiliated obstetrics practices. *Gen. Hosp. Psychiatry* 2006, 289–95.
23. Marcus, S. M., Flynn, H. A., Blow, F. C. & Barry, K. L. Depressive symptoms among pregnant women screened in obstetrics settings. *J Womens Health* 2003, 373–80.
24. Scholle, S. H., Haskett, R. F., Hanusa, B. H., Pincus, H. A. & Kupfer, D. J. Addressing depression in obstetrics/gynecology practice. *Gen. Hosp. Psychiatry* 2003, 83–90.
25. Ververs, T. *et al.* Prevalence and patterns of antidepressant drug use during pregnancy. *Eur.J.Clin.Pharmacol.* 62, 863–870 (2006).
26. Field, T., Diego, M. & Hernandez-Reif, M. Prenatal depression effects on the fetus and newborn: a review. *Infant Behav. Dev.* 2006, 445–55.
27. Chung, T. K. H., Lau, T. K., Yip, A. S. K., Chiu, H. F. K. & Lee, D. T. S. Antepartum Depressive Symptomatology Is Associated With Adverse Obstetric and Neonatal Outcomes. *Psychosom. Med.* 63, 830–834 (2001).
28. Sandman, C. A., Wadhwa, P. D., Chicz-DeMet, A., Dunkel-Schetter, C. & Porto, M. Maternal stress, HPA activity, and fetal/infant outcome. *Ann. N. Y. Acad. Sci.* 1997, 266–75.
29. Kurki, T., Hiilesmaa, V. K., Raitasalo, R., Mattila, H. & Ylikorkala, O. Depression and anxiety in early pregnancy and risk for preeclampsia. *Obstet. Gynecol.* 2000, 487–90.
30. Michel-Wolfromm, H. The psychological factor in spontaneous abortion. *J. Psychosom. Res.* 1968, 67–71.
31. Preti, A., Cardascia, L. & Zen, T. Obstetric complications in patients with depression–a population-based case-control study. *J. Affect. Disord.* 2000, 101–6.
32. Paarlberg, K. M., Vingerhoets, A. J., Passchier, J., Dekker, G. A. & Van Geijn, H. P. Psychosocial factors and pregnancy outcome: a review with emphasis on methodological issues. *J. Psychosom. Res.* 1995, 563–95.
33. Dayan, J., Creveuil, C. & Herlicoviez, M. Role of anxiety and depression in the onset of spontaneous preterm labor. *Am. J. Epidemiol.* 2002, 293–301.
34. Dayan, J., Creveuil, C. & Marks, M. N. Prenatal depression, prenatal anxiety, and spontaneous preterm birth: a prospective cohort study among women with early and regular care. *Psychosom. Med.* 2006, 938–46.
35. Orr, S. T., James, S. A. & Blackmore Prince, C. Maternal prenatal depressive symptoms and spontaneous preterm births among African-American women in Baltimore. *Md. Am. J. Epidemiol.* 2002, 797–802.
36. Weinstock, M. Alterations induced by gestational stress in brain morphology and behaviour of the offspring. in *Progress in neurobiology 2001;65* 427–51.
37. McAnarney, E. R. & Stevens-Simon, C. Maternal psychological stress/depression and low birth weight. Is there a relationship? *Am. J. Dis. Child.* 789–92 (1960).
38. Steer, R. A., Scholl, T. O., Hediger, M. L. & Fischer, R. L. Self-reported depression and negative pregnancy outcomes. *J. Clin. Epidemiol.* 1992, 1093–9.
39. Hoffman, S. & Hatch, M. C. Depressive symptomatology during pregnancy: evidence for an association with decreased fetal growth in pregnancies of lower social class women. *Health Psychol* 2000, 535–43.
40. Zax, M., Sameroff, A. J. & Babigian, H. M. Birth outcomes in the offspring of mentally disordered women. *Am. J. Orthopsychiatry* 1977, 218–30.
41. Cohler, B. J., Gallant, D. H., Grunebaum, H. U., Weiss, J. L. & Gamer, E. Pregnancy and birth complications among mentally ill and well mothers and their children. *Soc. Biol.*1975, 269–78.

42. Qiu, C., Sanchez, S. E., Lam, N., Garcia, P. & Williams, M. A. Associations of depression and depressive symptoms with preeclampsia: results from a Peruvian case-control study. *BMC Womens Health* 2007, 15.
43. Zuckerman, B., Amaro, H., Bauchner, H. & Cabral, H. Depressive symptoms during pregnancy: relationship to poor health behaviors. *Am J Obstet Gynecol* 1989, 1107–11.
44. Smedberg, J., Lupattelli, A., Mardby, A. C., Overland, S. & Nordeng, H. The relationship between maternal depression and smoking cessation during pregnancy–a cross-sectional study of pregnant women from 15 European countries. *Arch Womens Ment Health* 2015, 73–84.
45. Stewart, D. E. Clinical practice. Depression during pregnancy. *N Engl J Med* 2011, 1605–11.
46. Louik, C., Lin, A. E., Werler, M. M., Hernandez-Diaz, S. & Mitchell, A. A. First-trimester use of selective serotonin-reuptake inhibitors and the risk of birth defects. *N.Engl.J.Med.* 356, 2675–2683 (2007).
47. Alwan, S., Reefhuis, J., Rasmussen, S. A., Olney, R. S. & Friedman, J. M. Use of selective serotonin-reuptake inhibitors in pregnancy and the risk of birth defects. *N.Engl.J.Med.* 356, 2684–2692 (2007).
48. Berard, A. *et al.* First trimester exposure to paroxetine and risk of cardiac malformations in infants: the importance of dosage. *Birth Defects ResB DevReprodToxicol* 80, 18–27 (2007).
49. Cole, J. A., Ephross, S. A., Cosmatos, I. S. & Walker, A. M. Paroxetine in the first trimester and the prevalence of congenital malformations. *Pharmacoepidemiol. Saf* 16, 1075–1085 (2007).
50. Wogelius, P. *et al.* Maternal use of selective serotonin reuptake inhibitors and risk of congenital malformations. *Epidemiology* 17, 701–704 (2006).
51. Chambers, C. D. *et al.* Selective serotonin-reuptake inhibitors and risk of persistent pulmonary hypertension of the newborn. *N Engl J Med* 354, 579–587 (2006).
52. Kallen, B. Fluoxetine use in early pregnancy. *Birth Defects ResB DevReprodToxicol* 71, 395–396 (2004).
53. Wen, S. W. *et al.* Selective serotonin reuptake inhibitors and adverse pregnancy outcomes. *Am.J.Obstet.Gynecol.* 194, 961–966 (2006).
54. Oberlander, T. F., Warburton, W., Misri, S., Aghajanian, J. & Hertzman, C. Neonatal outcomes after prenatal exposure to selective serotonin reuptake inhibitor antidepressants and maternal depression using population-based linked health data. *Arch. Gen. Psychiatry* 63, 898–906 (2006).
55. Davis, E. P. *et al.* Prenatal exposure to maternal depression and cortisol influences infant temperament. *J. Am. Acad. Child Adolesc. Psychiatry* 2007, 737–46.
56. Costei, A. M., Kozer, E., Ho, T., Ito, S. & Koren, G. Perinatal outcome following third trimester exposure to paroxetine. *Arch.Pediatr.Adolesc.Med.* 156, 1129–1132 (2002).
57. Pedersen, L. H., Henriksen, T. B., Vestergaard, M., Olsen, J. & Bech, B. H. Selective serotonin reuptake inhibitors in pregnancy and congenital malformations: population based cohort study. *BMJ* 339, b3569 (2009).
58. Davis, R. L. *et al.* Risks of congenital malformations and perinatal events among infants exposed to antidepressant medications during pregnancy. *Pharmacoepidemiol. Saf* 16, 1086–1094 (2007).
59. Ramos, E., St-Andre, M., Rey, E., Oraichi, D. & Berard, A. Duration of antidepressant use during pregnancy and risk of major congenital malformations. *Br.J.Psychiatry* 192, 344–350 (2008).
60. Gentile, S. Selective serotonin reuptake inhibitor exposure during early pregnancy and the risk of birth defects. *Acta Psychiatr. Scand.* 123, 266–275 (2011).
61. Margulis, A. V. *et al.* Use of selective serotonin reuptake inhibitors in pregnancy and cardiac malformations: a propensity-score matched cohort in CPRD. *Pharmacoepidemiol. Drug Saf.* 22, 942–951 (2013).

62. Ban, L. *et al.* Maternal depression, antidepressant prescriptions, and congenital anomaly risk in offspring: a population-based cohort study. *BJOG Int. J. Obstet. Gynaecol.* 121, 1471–1481 (2014).
63. Jimenez-Solem, E. *et al.* Exposure to selective serotonin reuptake inhibitors and the risk of congenital malformations: a nationwide cohort study. *BMJ Open* 2, (2012).
64. Huybrechts, K. F., Bateman, B. T. & Palmsten, K. Antidepressant use late in pregnancy and risk of persistent pulmonary hypertension of the newborn. *JAMA* 2015, 2142–51.
65. Huybrechts, K. F., Palmsten, K. & Avorn, J. Antidepressant use in pregnancy and the risk of cardiac defects. *N Engl J Med* 2014, 2397–407.
66. Furu, K., Kieler, H. & Haglund, B. Selective serotonin reuptake inhibitors and venlafaxine in early pregnancy and risk of birth defects: population based cohort study and sibling design. *BMJ* 2015, 1798.
67. Einarson, A. *et al.* Pregnancy outcome following gestational exposure to venlafaxine: a multicenter prospective controlled study. *Am.J.Psychiatry* 158, 1728–1730 (2001).
68. Oberlander, T. F. *et al.* Major congenital malformations following prenatal exposure to serotonin reuptake inhibitors and benzodiazepines using population-based health data. *Birth Defects ResB DevReprodToxicol* 83, 68–76 (2008).
69. Jain, R. Single-Action Versus Dual-Action Antidepressants. *Prim. Care Companion J. Clin. Psychiatry* 6, 7–11 (2004).
70. Halbreich, U. The association between pregnancy processes, preterm delivery, low birth weight, and postpartum depressions–the need for interdisciplinary integration. *Am J Obstet Gynecol* 2005, 1312–22.
71. McIntire, D. D., Bloom, S. L., Casey, B. M. & Leveno, K. J. Birth weight in relation to morbidity and mortality among newborn infants. *N Engl J Med* 1999, 1234–8.
72. Roberts, J. M. in *Endothelial Dysfunction in Preeclampsia. Seminars in Reproductive Endocrinology* 1998;16, 5–15.
73. Chambers, C. D., Johnson, K. A., Dick, L. M., Felix, R. J. & Jones, K. L. Birth outcomes in pregnant women taking fluoxetine. *N. Engl. J. Med.* 335, 1010–1015 (1996).
74. Huybrechts, K. F., Sanghani, R. S., Avorn, J. & Urato, A. C. Preterm birth and antidepressant medication use during pregnancy: a systematic review and meta-analysis. *PloS One* 2014, 92778.
75. Chrousos, G. P., Torpy, D. J. & Gold, P. W. Interactions between the hypothalamicpituitary-adrenal axis and the female reproductive system: clinical implications. *Ann Intern Med* 229–40 (1998).
76. Hendricks, S. K., SJ, R., Moore, D. E. & Brown, Z. A. Oligohydramnios associated with prostaglandin synthetase inhibitors in preterm labor. *Br. J. Obstet. Gynaecol.* 312–6 (1990).
77. Bassiouni, B. A. & Rafei, A. A. 5-Hydroxytryptamine (serotonin), copper and ceruloplasmin plasma concentrations in spontaneous abortion. *Eur. J. Obstet. Gynecol. Reprod. Biol.* 9, 81–88 (1979).
78. Pastuszak, A. *et al.* Pregnancy outcome following first-trimester exposure to fluoxetine (Prozac). *JAMA* 269, 2246–2248 (1993).
79. McElhatton, P. R. *et al.* The outcome of pregnancy in 689 women exposed to therapeutic doses of antidepressants. A collaborative study of the European Network of Teratology Information Services (ENTIS). *Reprod Toxicol* 10, 285–294 (1996).
80. Goldstein, D. J., Corbin, L. A. & Sundell, K. L. Effects of first-trimester fluoxetine exposure on the newborn. *Obstet.Gynecol.* 89, 713–718 (1997).
81. Kulin, N. A. *et al.* Pregnancy outcome following maternal use of the new selective serotonin reuptake inhibitors: a prospective controlled multicenter study. *JAMA* 279, 609–610 (1998).
82. Sivojelezova, A., Shuhaiber, S., Sarkissian, L., Einarson, A. & Koren, G. Citalopram use in pregnancy: Prospective comparative evaluation of pregnancy and fetal outcome. *Am. J. Obstet. Gynecol.* 193, 2004–2009 (2005).

83. Diav-Citrin, O. *et al.* Paroxetine and fluoxetine in pregnancy: a prospective, multicentre, controlled, observational study. *Br.J.Clin.Pharmacol.* 66, 695–705 (2008).
84. Kjaersgaard, M. I. *et al.* Prenatal antidepressant exposure and risk of spontaneous abortion - a population-based study. *PLoS.One.* 8, e72095 (2013).
85. Andersen, J. T., Andersen, N. L., Horwitz, H., Poulsen, H. E. & Jimenez-Solem, E. Exposure to selective serotonin reuptake inhibitors in early pregnancy and the risk of miscarriage. *Obstet. Gynecol.* 124, 655–661 (2014).
86. Stephansson, O. *et al.* Selective serotonin reuptake inhibitors during pregnancy and risk of stillbirth and infant mortality. *JAMA* 309, 48–54 (2013).
87. Jimenez-Solem, E. *et al.* SSRI Use During Pregnancy and Risk of Stillbirth and Neonatal Mortality. *Am. J. Psychiatry* 170, 299–304 (2013).
88. Rom, A. L. *et al.* A comparative study of educational inequality in the risk of stillbirth in Denmark, Finland, Norway and Sweden 1981–2000. *J Epidemiol Community Health* 66, 240–246 (2012).
89. Hudson, J. I. *et al.* Safety and tolerability of duloxetine in the treatment of major depressive disorder: analysis of pooled data from eight placebo-controlled clinical trials. *Hum. Psychopharmacol.* 2005, 327–41.
90. Hoog, S. L., Cheng, Y., Elpers, J. & Dowsett, S. A. Duloxetine and pregnancy outcomes: safety surveillance findings. *Int. J. Med. Sci.* 2013, 413–9.
91. Eyal, R. & Yaeger, D. Poor neonatal adaptation after in utero exposure to duloxetine. *Am J Psychiatry* 2008, 651.
92. Abdy, N. A. & Gerhart, K. Duloxetine withdrawal syndrome in a newborn. *Clin. Pediatr. (Phila.)* 2013, 976–7.
93. Koren, G., Matsui, D., Einarson, A., Knoppert, D. & Steiner, M. Is maternal use of selective serotonin reuptake inhibitors in the third trimester of pregnancy harmful to neonates? *Can. Med. Assoc. J.* 172, 1457–1459 (2005).
94. Briggs, G. G. *et al.* Use of duloxetine in pregnancy and lactation. *Ann. Pharmacother.* 2009, 1898–902.
95. Bellantuono, C., Marini, A. & Lucarelli, C. Infant health and neurodevelopmental outcomes following prenatal exposure to duloxetine. *Clin. Drug Investig.* 2013, 685–8.
96. Einarson, A. *et al.* Rates of major malformations in infants following exposure to duloxetine during pregnancy: a preliminary report. *J.Clin.Psychiatry* 73, 1471 (2012).
97. Kallen, B., Borg, N. & Reis, M. *The use of central nervous system active drugs during pregnancy.* 1221–86 (Basel, 2013).
98. Lassen, D., Ennis, Z. N. & Damkier, P. First-Trimester Pregnancy Exposure to Venlafaxine or Duloxetine and Risk of Major Congenital Malformations: A Systematic Review. *Basic Clin. Pharmacol. Toxicol.* 2016, 32–6.
99. Källén, B. & Olausson, P. O. No increased risk of infant hypospadias after maternal use of loratadine in early pregnancy. *Int. J. Med. Sci.* 3, 106–107 (2006).
100. Lohse, S. R. et al. Validation of spontaneous abortion diagnoses in the Danish National Registry of Patients. Clin. Epidemiol. 2, 247–250 (2010).
101. Larsen, H. *et al.* Predictive value and completeness of the registration of congenital abnormalities in three Danish population-based registries. *ScandJPublic Health* 31, 12–16 (2003).
102. Knudsen, L. B. & Olsen, J. The Danish Medical Birth Registry. *Dan.Med.Bull.* 45, 320–323 (1998).
103. Centre for Epidemiology, National Board of Health and Welfare. The Swedish Medical Birth Register: A Summary of Content and Quality. (2003).
104. Kristensen, J., Langhoff-Roos, J., Skovgaard, L. T. & Kristensen, F. B. Validation of the Danish Birth Registration. *J.Clin.Epidemiol.* 49, 893–897 (1996).

105. Langhoff-Roos, J. *et al.* The Nordic medical birth registers--a potential goldmine for clinical research. *Acta Obstet. Gynecol. Scand.* 93, 132–137 (2014).
106. Baadsgaard, M. & Quitzau, J. Danish registers on personal income and transfer payments. *Scand. J. Public Health* 39, 103–105 (2011).
107. Kristiansen, I. S. & Pedersen, K. M. [Health care systems in the Nordic countries—more similarities than differences?]. *Tidsskr. Den Nor. Laegeforening Tidsskr. Prakt. Med. Ny Raekke* 120, 2023–2029 (2000).
108. Jensen, V. M. & Rasmussen, A. W. Danish Education Registers. *Scand. J. Public Health* 39, 91–94 (2011).
109. Knudsen, L. B. & Børlum Kristensen, F. Monitoring perinatal mortality and perinatal care with a national register: content and usage of the Danish Medical Birth Register. *Community Med.* 8, 29–36 (1986).
110. Andersen, J. T. *et al.* Diclofenac/misoprostol during early pregnancy and the risk of miscarriage: a Danish nationwide cohort study. *Arch. Gynecol. Obstet.* 294, 245–250 (2016).
111. The Swedish Register of Education.
112. Ringbäck Weitoft, G., Ericsson, O., Löfroth, E. & Rosén, M. Equal access to treatment? Population-based follow-up of drugs dispensed to patients after acute myocardial infarction in Sweden. *Eur. J. Clin. Pharmacol.* 64, 417–424 (2008).
113. Johnell, K., Weitoft, G. R. & Fastbom, J. Education and use of dementia drugs: a registerbased study of over 600,000 older people. *Dement. Geriatr. Cogn. Disord.* 25, 54–59 (2008).
114. Haider, S. I., Johnell, K., Ringbäck Weitoft, G., Thorslund, M. & Fastbom, J. Patient educational level and use of newly marketed drugs: a register-based study of over 600,000 older people. *Eur. J. Clin. Pharmacol.* 64, 1215–1222 (2008).
115. The Finish Register of Completed Education and Degrees and student flow statistics.
116. Epland, J. Towards a Register-based Income Statistics - the Construction of the Norwegian Income Register.
117. Webster, P. C. Sweden's health data goldmine. *CMAJ Can. Med. Assoc. J.* 186, E310 (2014).
118. Ludvigsson, J. F. *et al.* Registers of the Swedish total population and their use in medical research. *Eur. J. Epidemiol.* 31, 125–136 (2016).
119. Swedish Register of education. Available at: http://www.jpidataproject.eu/Home/Database/348?topicId=4. (Accessed: 27th July 2017)
120. Statistics Sweden. *Statistiska Centralbyrån* Available at: http://www.scb.se/en/. (Accessed:27th July 2017)
121. Meidahl Petersen, K. *et al.* β-Blocker treatment during pregnancy and adverse pregnancy outcomes: a nationwide population-based cohort study. *BMJ Open* 2, (2012).
122. Sørensen, H. T., Hansen, I., Ejlersen, E., Sabroe, S. & Hamburger, H. Identification of patients treated with strong analgesics: an assessment of two Danish information systems with respect to epidemiological research. *J. Med. Syst.* 20, 57–65 (1996).
123. Kieler, H. *et al.* Use of antidepressants and association with elective termination of pregnancy: population based case-control study. *BJOG Int. J. Obstet. Gynaecol.* 122, 1618–1624 (2015).
124. Andersen, J. T. *et al.* Clarithromycin in early pregnancy and the risk of miscarriage and malformation: a register based nationwide cohort study. *PloS One* 8, e53327 (2013).
125. Ludvigsson J. External review and validation of the Swedish national inpatient register [Internet]. [cited 2017 Jul 22]. Available from: https://www-ncbi-nlm-nih-gov.ep.fjernadgang.kb.dk/pmc/articles/PMC3142234/

126. Wettermark B, Hammar N, Fored CM, MichaelFored C, Leimanis A, Otterblad Olausson P, et al. The new Swedish Prescribed Drug Register--opportunities for pharmacoepidemiological research and experience from the first six months. Pharmacoepidemiol Drug Saf. 2007 Jul;16(7):726–35.
127. Ludvigsson JF, Almqvist C, Bonamy A-KE, Ljung R, Michaëlsson K, Neovius M, et al. Registers of the Swedish total population and their use in medical research. Eur J Epidemiol. 2016 Feb;31(2):125–36.
128. Swedish Register of education [Internet]. [cited 2017 Jul 27]. Available from: http://www.jpi-dataproject.eu/Home/Database/348?topicId=4
129. Statistics Sweden [Internet]. Statistiska Centralbyrån. [cited 2017 Jul 27]. Available from: http://www.scb.se/en/
130. Meidahl Petersen K, Jimenez-Solem E, Andersen JT, Petersen M, Brødbæk K, Køber L, et al. β-Blocker treatment during pregnancy and adverse pregnancy outcomes: a nationwide population-based cohort study. BMJ Open. 2012;2(4).
131. Gaist D, Sorensen HT, Hallas J. The Danish prescription registries. DanMedBull. 1997 Sep;44(0907–8916 (Print)):445–8.
132. Buss L, Tolstrup J, Munk C, Bergholt T, Ottesen B, Grønbaek M, et al. Spontaneous abortion: a prospective cohort study of younger women from the general population in Denmark. Validation, occurrence and risk determinants. Acta Obstet Gynecol Scand. 2006;85(4):467–75.
133. Baadsgaard M, Quitzau J. Danish registers on personal income and transfer payments. *Scand J Public Health*. 2011;39(7 suppl):103-105.

Annex 1. List of Standalone Documents

Not applicable.

Annex 2. ENCePP Checklist for Study Protocols

Not applicable.

Study title:

Observational Study to Assess Fetal Outcomes Following Exposure to Duloxetine

Study reference number:

FJ1-MC-B059

**Section 1: Milestones**	**Yes**	**No**	**N/A**	**Section Number**
1.1. Does the protocol specify timelines for				
1.1.1. Start of data collection	☒	☐	☐	6
1.1.2. End of data collection	☒	☐	☐	6
1.1.3. Study progress report(s)	☐	☐	☒	
1.1.4. Interim progress report(s)	☐	☐	☒	
1.1.5. Registration in the EU PAS register	☐	☐	☐	
1.1.6. Final report of study results.	☒	☐	☐	6

Comments:

**Section 2: Research question**	**Yes**	**No**	**N/A**	**Section Number**
2.1. Does the formulation of the research question and objectives clearly explain:				
2.1.1. Why the study is conducted? (e.g., to address an important public health concern, a risk identified in the risk management plan, an emerging	☒	☐	☐	7

**Section 2: Research question**	**Yes**	**No**	**N/A**	**Section Number**
safety issue)<br>2.1.2. The objective(s) of the study?	☒	☐	☐	8
2.1.3. The target population? (i.e., population or subgroup to whom the study results are intended to be generalised)	☒	☐	☐	8
2.1.4. Which formal hypothesis(-es) is (are) to be tested?	☒	☐	☐	8
2.1.5. If applicable, that there is no *a priori* hypothesis?	☐	☐	☒	

Comments:

**Section 3: Study design**	**Yes**	**No**	**N/A**	**Section Number**
3.1. Is the study design described? (e.g., cohort, case-control, cross-sectional, new or alternative design)	☒	☐	☐	9.1
3.2. Does the protocol specify whether the study is based on primary, secondary, or combined data collection?	☒	☐	☐	9.1
3.3. Does the protocol specify measures of occurrence? (e.g., incidence rate, absolute risk)	☒	☐	☐	9.5
3.4. Does the protocol specify measure(s) of association? (e.g., relative risk, odds ratio, excess risk, incidence rate ratio, hazard ratio, number needed to harm [NNH] per year)	☒	☐	☐	9.7
3.5. Does the protocol describe the approach for the collection and reporting of adverse events/adverse reactions? (e.g., adverse events that will not be collected in case of primary data collection)	☐	☐	☒	

Comments:

**Section 4: Source and study populations**	**Yes**	**No**	**N/A**	**Section Number**
4.1. Is the source population described?	☒	☐	☐	9.4
4.2. Is the planned study population defined in terms of:				
4.2.1. Study time period?	☒	☐	☐	9.2.1
4.2.2. Age and sex?	☒	☐	☐	9.2.1
4.2.3. Country of origin?	☒	☐	☐	9.1
4.2.4. Disease/indication?	☒	☐	☐	9.2.1
4.2.5. Duration of follow-up?	☐	☐	☒	
4.3. Does the protocol define how the study population will be sampled from the source population? (e.g., event or inclusion/exclusion criteria)	☒	☐	☐	9.2.1

Comments:

**Section 5: Exposure definition and measurement**	**Yes**	**No**	**N/A**	**Section Number**
5.1. Does the protocol describe how the study exposure is defined and measured? (e.g., operational details for defining and categorising exposure, measurement of dose and duration of drug exposure)	☒	☐	☐	9.3.1
5.2. Does the protocol address the validity of the exposure measurement? (e.g., precision, accuracy, use of validation substudy)	☐	☒	☐	
5.3. Is exposure classified according to time windows? (e.g., current user, former user, non-use)	☒	☐	☐	9.1
5.4. Is exposure classified based on biological mechanism of action and taking into account the pharmacokinetics and pharmacodynamics of the drug?	☒	☐	☐	9.1

Comments:

Section 6: Outcome definition and measurement	Yes	No	N/A	Section Number
6.1. Does the protocol specify the primary and secondary (if applicable) outcome(s) to be investigated?	☒	☐	☐	9.1 and 9.3.2
6.2. Does the protocol describe how the outcomes are defined and measured?	☒	☐	☐	9.3.2
6.3. Does the protocol address the validity of outcome measurement? (e.g., precision, accuracy, sensitivity, specificity, positive predictive value, prospective or retrospective ascertainment, use of validation substudy)	☒	☐	☐	9.4.4
6.4. Does the protocol describe specific endpoints relevant for Health Technology Assessment? (e.g., HRQoL, QALYs, DALYS, health care services utilisation, burden of disease, disease management)	☐	☐	☒	

Comments:

Section 7: Bias	Yes	No	N/A	Section Number
7.1. Does the protocol describe how confounding will be addressed in the study?	☒	☐	☐	9.9
7.1.1. Does the protocol address confounding by indication if applicable?	☒	☐	☐	9.1
7.2. Does the protocol address:	☐	☐	☐	
7.2.1. Selection biases (e.g. healthy user bias)	☐	☒	☐	
7.2.2. Information biases (e.g., misclassification of exposure and endpoints, time-related bias)	☒	☐	☐	9.9
7.3. Does the protocol address the validity of the study covariates?	☒	☐	☐	9.3.3

Comments:

**Section 8: Effect modification**	**Yes**	**No**	**N/A**	**Section Number**
8.1. Does the protocol address effect modifiers? (e.g., collection of data on known effect modifiers, subgroup analyses, anticipated direction of effect)	☐	☒	☐	

Comments:

**Section 9: Data sources**	**Yes**	**No**	**N/A**	**Section Number**
9.1. Does the protocol describe the data source(s) used in the study for the ascertainment of:				
9.1.1. Exposure? (e.g., pharmacy dispensing, general practice prescribing, claims data, self-report, face-to-face interview)	☒	☐	☐	9.4
9.1.2. Outcomes? (e.g., clinical records, laboratory markers or values, claims data, self-report, patient interview including scales and questionnaires, vital statistics)	☒	☐	☐	9.4
9.1.3. Covariates?	☒	☐	☐	9.4
9.2. Does the protocol describe the information available from the data source(s) on:				
9.2.1. Exposure? (e.g., date of dispensing, drug quantity, dose, number of days of supply prescription, daily dosage, prescriber)	☒	☐	☐	9.4
9.2.2. Outcomes? (e.g., date of occurrence, multiple event, severity measures related to event)	☒	☐	☐	9.4
9.2.3. Covariates? (e.g., age, sex, clinical and drug use history, comorbidity, comedications, lifestyle)	☒	☐	☐	9.4
9.3. Is a coding system described for:				
9.3.1. Exposure? (e.g. WHO Drug Dictionary, Anatomical	☒	☐	☐	9.3.1

**Section 9: Data sources**	**Yes**	**No**	**N/A**	**Section Number**
Therapeutic Chemical (ATC) Classification System)				
9.3.2. Outcomes? (e.g. International Classification of Diseases [ICD]-10, Medical Dictionary for Regulatory Activities [MedDRA])	☒	☐	☐	9.4.3
9.3.3. Covariates?	☒	☐	☐	9.4.3
9.4. Is the linkage method between data sources described? (e.g., based on a unique identifier or other)	☒	☐	☐	9.1

Comments:

**Section 10: Analysis plan**	**Yes**	**No**	**N/A**	**Section Number**
10.1. Is the choice of statistical techniques described?	☒	☐	☐	9.7
10.2. Are descriptive analyses included?	☒	☐	☐	9.7
10.3. Are stratified analyses included?	☒	☐	☐	9.7
10.4. Does the plan describe methods for adjusting for confounding?	☒	☐	☐	9.7
10.5. Does the plan describe methods for handling missing data?	☒	☐	☐	9.6.2.3
10.6. Is sample size and/or statistical power estimated?	X			9.5

Comments:

**Section 11: Data management and quality control**	**Yes**	**No**	**N/A**	**Section Number**
11.1. Does the protocol provide information on data storage? (e.g., software and IT environment, database maintenance and anti-fraud protection, archiving)	☒	☐	☐	9.6

**Section 11: Data management and quality control**	**Yes**	**No**	**N/A**	**Section Number**
11.2. Are methods of quality assurance described?	☒	☐	☐	9.8
11.3. Is there a system in place for independent review of study results?	☒	☐	☐	9.8

Comments:

**Section 12: Limitations**	**Yes**	**No**	**N/A**	**Section Number**
12.1. Does the protocol discuss the impact on the study results of:				
12.1.1. Selection biases?	☐	☒	☐	
12.1.2. Information biases?	☐	☒	☐	
12.1.3. Residual/unmeasured confounding?	☒	☐	☐	
(e.g., anticipated direction and magnitude of such biases, validation substudy, use of validation and external data, analytical methods)				9.9
12.2. Does the protocol discuss study feasibility? (e.g., study size, anticipated exposure, duration of follow-up in a cohort study, patient recruitment)	☒	☐	☐	9.5

Comments:

**Section 13: Ethical issues**	**Yes**	**No**	**N/A**	**Section Number**
13.1. Have requirements of Ethics Committee/Institutional Review Board been described?	☐	☐	☒	
13.2. Has any outcome of an ethical review procedure been addressed?	☐	☐	☒	
13.3. Have data protection requirements been described?	☒	☐	☐	9.6

Comments:

**Section 14: Amendments and deviations**	**Yes**	**No**	**N/A**	**Section Number**
14.1. Does the protocol include a section to document future amendments and deviations?	☐	☒	☐	

Comments:

**Section 15: Plans for communication of study results**	**Yes**	**No**	**N/A**	**Section Number**
15.1. Are plans described for communicating study results (e.g., to regulatory authorities)?	☒	☐	☐	12.3
15.2. Are plans described for disseminating study results externally, including publication?	☒	☐	☐	12.3

Comments:

Name of the main author of the protocol: PPD

Date: 22/08/2017

Signature: PPD

Annex 3. Additional Information

Main responsible parties

PPD

Major congenital malformations according to the EUROCAT classification of major congenital malformations version 1.4.

EUROCAT Subgroups BOLD	ICD-10	Comments
**All anomalies**	Q-chapter, D821	All Anomalies = ALL cases of congenital anomaly, excluding cases with only minor anomalies as defined below. Cases with more than 1 anomaly are only counted once in the "All Anomalies" subgroup.
**Nervous system**	Q00, Q01, Q02, Q03, Q04, Q05, Q06, Q07	
Neural Tube Defects	Q00, Q01, Q05	
Anencephalus and similar	Q00	

Encephalocele	Q01	Exclude if associated with anencephalus subgroup
Spina bifida	Q05	Exclude if associated with anencephalus or encephalocele subgroups
Hydrocephalus	Q03	Exclude hydranencephaly 74232. Exclude association with NTD subgroup
Severe microcephaly	Q02	Exclude association with NTD subgroup
Arhinencephaly / holoprosencephaly	Q041, Q042	
**Eye**	Q10-Q15	
Anophthalmos / microphthalmos	Q110, Q111, Q112	
Anophthalmos	Q110, Q111	
Congenital cataract	Q120	
Congenital glaucoma	Q150	
**Ear, face and neck**<br>Anotia	Q16, Q17, Q18<br>Q160	
**Congenital Heart Defects**	Q20-Q26	Exclude PDA with GA <37 weeks<br>Exclude peripheral pulmonary artery stenosis with GA < 37 weeks
Severe Congenital Heart Defect	Q200, Q201, Q203, Q204, Q212, Q213, Q220, Q224, Q225, Q226, Q230, Q232, Q233, Q234, Q251, Q252, Q262	ICD9-BPA has no code for HRH and double outlet right ventricle
Common arterial truncus	Q200	
Double outlet right ventricle	Q201	
Transposition of great vessels	Q203	
Single ventricle	Q204	
Ventricular septal defect	Q210	
Atrial septal defect	Q211	
Atrioventricular septal defect	Q212	
Tetralogy of Fallot	Q213	

Triscuspid atresia and stenosis	Q224	
Ebstein's anomaly	Q225	
Pulmonary valve stenosis	Q221	
Pulmonary valve atresia	Q220	
Aortic valve atresia/stenosis	Q230	
Mitral valve anomalies	Q232, Q233	
Hypoplastic left heart	Q234	
Hypoplastic right heart	Q226	
Coarctation of aorta	Q251	
Aortic atresia / interrupted aortic arch	Q252	
Total anomalous pulmonary venous return	Q262	
Patent ductus arteriosus as only congenital heart defect in term infants (gestational age +37 weeks)	Q250	Livebirths only
**Respiratory**	Q300, Q32-Q34	Exclude Q336
Choanal atresia	Q300	
Cystic adenomatous malformation of lung	Q3380	
**Oro-facial clefts**	Q35-Q37	Exclude association with holoprosencephaly or anencephaly subgroups
Cleft lip with or without cleft palate	Q36, Q37	Exclude association with holoprosencephaly or anencephaly subgroups
Cleft palate	Q35	Exclude association with cleft lip subgroup. Exclude association with holoprosencephaly or anencephaly subgroups
**Digestive system**	Q38-Q45, Q790	
Oesophageal atresia with or without trachea-oesophageal fistula	Q390-Q391	

Duodenal atresia or stenosis	Q410	Exclude if also annular pancreas subgroup
Atresia or stenosis of other parts of small intestine	Q411-Q418	
Ano-rectal atresia and stenosis	Q420-Q423	
Hirschsprung's disease	Q431	
Atresia of bile ducts	Q442	
Annular pancreas	Q451	
Diaphragmatic hernia	Q790	
**Abdominal wall defects**	Q792, Q793, Q795	
Gastroschisis	Q793	
Omphalocele	Q792	
**Urinary**	Q60-Q64, Q794	
Bilateral renal agenesis including Potter syndrome	Q601, Q606	Exclude unilateral
Multicystic renal dysplasia	Q6140, Q6141	
Congenital hydronephrosis	Q620	
Bladder exstrophy and / or epispadia	Q640, Q641	
Posterior urethral valve and / or prune belly	Q6420, Q794	
**Genital**	Q50-Q52, Q54-Q56	
Hypospadias	Q54	
Indeterminate sex	Q56	
**Limb**	Q65-Q74	
Limb reduction defects	Q71-Q73	
Club foot – talipes equinovarus	Q660	
Hip dislocation and / or dyspasia	Q650-Q652, Q6580, Q6581	
Polydactyly	Q69	
Syndactyly	Q70	

**Other anomalies/syndromes**		
Skeletal dysplasias	Q7402, Q77, Q7800, Q782-Q788,	
Craniosynostosis	Q750	
Congenital constriction bands / amniotic band	Q7980	
Situs inversus	Q893	
Conjoined twins	Q894	
Congenital skin disorders	Q80-Q82	
VATER/VACTERL	Q8726	
Vascular disruption anomalies	Q0435, Q411, Q412, Q418, Q710, Q712, Q713, Q720, Q722, Q723, Q730, Q793, Q795, Q7980,	
Laterality anomalies	Q206, Q240, Q3381, Q890, Q893	
Teratogenic syndromes with malformations	Q86	
Fetal alcohol syndrome	Q860	
Valproate syndrome	Q8680	
Maternal infections resulting in malformations	P350, P351, P371	
Genetic syndromes + microdeletions	Q4471, Q6190, Q7484, Q751, Q754, Q7581, Q87, Q936, D821	Exclude associations and sequences.<br>Exclude Q8703, Q8704, Q8706, Q8708, Q8724, Q8726<br>Exclude 759801, 759844, 759895

Minor malformations according to the EUROCAT classification version 1.4.

EUROCAT Classifications	ICD-10	Comments
**Head**		
Aberrant scalp hair patterning		ICD-10 is not available
Bony occipital spur		ICD-10 is not available
Brachycephaly		ICD-10 is not available
Compression facies	Q671	
Depressions in skull	Q6740	
Dolichocephaly	Q672	
Dysmorphic face	Q189	
Facial asymmetry	Q670	
Flat occiput		ICD-10 is not available
Frontal bossing / wide forehead		ICD-10 is not available
Plagiocephaly – head asymmetry	Q673	
Macrocephalus	Q753	
Metopic ridge		ICD-10 is not available
Metopic suture synostosis		ICD-10 is not available
Other congenital deformities of skull, face and jaw	Q674	
Third fontanel		ICD-10 is not available
**Eyes**		
Blue sclera	Q135	
Congenital ectropion	Q101	
Congenital entropion	Q102	
Crocodile tears	Q0782	
Downward slanting palpebral fissures		ICD-10 is not available
Dystopia canthorum		ICD-10 is not available
Epicanthic folds		ICD-10 is not available
Epicanthus inversus		ICD-10 is not available
Exophthalmos		ICD-10 is not available
Hypertelorism	Q752	
Hypotelorism		ICD-10 is not available
Other congenital malformations of eyelid	Q103	
Short palpebral fissures		ICD-10 is not available
Stenosis or stricture of lacrimal duct	Q105	
Synophrys	Q1880	
Upward slanting palpebral fissures		ICD-10 is not available
**Ears**		
Absent tragus		ICD-10 is not available
Accesorry auricle, preauricular appendage, tag, or lobule	Q170	
Asymmetric size	Q173	
Auricular pit		ICD-10 is not available

Bat ear, prominent ear	Q175	
Double lobule	Q170	
Lack of helical fold	Q173	
Low set ears	Q174	
Macrotia	Q171	
Microtia	Q172	
Narrow external auditory meatus		ICD-10 is not available
Posterior angulation	Q173	
Preauricular sinus or cyst	Q181	
Primitive shape	Q173	
Protuberant ears	Q173	
Unspecified and minor malformation of ear	Q179	
**Nose**		
Anomalies of philtrum		ICD-10 is not available
Broad nasal root, anomaly of nasal root		ICD-10 is not available
Deviation of nasal septum	Q6741	
Dysmorphic nose	Q189	
Notched alas		ICD-10 is not available
Small nares		ICD-10 is not available
**Oral regions**		
Aberrant frenula		ICD-10 is not available
Alveolar crest		ICD-10 is not available
Borderline small mandible/ minor micrognathia		ICD-10 is not available
Disturbances in tooth eruption		ICD-10 is not available
Enamel hypoplasia		ICD-10 is not available
Glossoptosis		ICD-10 is not available
High arched palate	Q3850	
Macrocheilia	Q186	
Macroglossia	Q382	
Macrostomia	Q184	
Malformed teeth		ICD-10 is not available
Microcheilia	Q187	
Microstomia	Q185	
Neonatal teeth		ICD-10 is not available
Ranula		ICD-10 is not available
Retrognathia	Q674	
Thin lips		ICD-10 is not available
Tongue tie or cyst of tongue	Q381	
**Neck**		
Congenital malformation of face and neck, unspecified	Q189	
Mild webbed neck		ICD-10 is not available
Other branchial cleft malformations	Q182	
Preauricular sinus or cyst	Q181	
Sinus, fistula or cyst of branchial	Q180	

cleft		
Torticollis	Q680	
**Hands**		
Accessory carpal bones	Q7400	
Arachnodactyly		ICD-10 is not available
Clinodactyly (5th finger)	Q6810	
Duplication of thumbnail		
Enlarged or hypertrophic nails	Q845	
Overlapping fingers		ICD-10 is not available
Short fingers (4th, 5th fingers)		
Single/abnormal palmar crease	Q8280	
Small fingers		ICD-10 is not available
Unusual dermatoglyphics		ICD-10 is not available
**Feet – Limb**		
Clicking hip subluxation or unstable hip	Q653-Q656	
Clubfoot of postural origin – other congenital deformities of feet	Q668	
Congenital deformity of feet, unspecified	Q669	
Congenital pes planus	Q665	
Enlarged or hypertrophic nails	Q845	
Gap between toes (1st-2nd)		ICD-10 is not available
Hallux varus – other congenital varus deformities of feet	Q663	
Metatarsus varus – other congenital valgus deformities of feet	Q666	
Metatarsus varus or metatarsus adductus	Q662	
Overlapping toes		ICD-10 is not available
Pes cavus	Q667	
Prominent calcaneus		ICD-10 is not available
Recessed toes (4th, 5th)		ICD-10 is not available
Short great toe		ICD-10 is not available
Syndactyly (2nd-3rd toes)		ICD-10 is not available
Talipes or pes calcaneovalgus	Q664	
**Skin**		
Accessory nipples	Q833	
Angioma		ICD-10 is not available
Cafe-au-lait spot		ICD-10 is not available
Depigmented spot		ICD-10 is not available
Hemangioma if no treatment is required		ICD-10 is not available
Heterochromia of hair		ICD-10 is not available
Hypoplasia of toe nails		ICD-10 is not available
Lymphangioma		ICD-10 is not available
Mongoloid spot (whites)	Q8252	
Neavus flammeus	Q8250	

Persistent lanugo		ICD-10 is not available
Pigmented naevus – congenital non-neoplastic naevus	Q825	
Strawberry naevus	Q8251	
Unusual placement of nipples/ wide spaced nipples		ICD-10 is not available
**Skeletal**		
Abortive 12th rib		ICD-10 is not available
Absence of rib	Q7660	
Accessory rib	Q7662	
Cervical rib	Q765	
Congenital bowing of femur	Q683	
Congenital bowing of fibula and tibia	Q684	
Congenital bowing of long bones of leg, unspecified	Q685	
Congenital bowing of upper limb		ICD-10 is not available
Congenital deformity of spine	Q675	
Congenital lordosis, postural	Q7643	
Cubitus valgus		ICD-10 is not available
Depressed sternum	Q676	
Fused rib, single		ICD-10 is not available
Genu recurvatum	Q6821	
Genu valgum		ICD-10 is not available
Genu varum		ICD-10 is not available
Prominent sternum	Q677	
Sacral dimple		ICD-10 is not available
Shieldlike chest, other congenital deformities of chest	Q678	
Spina bifida occulta	Q760	
Sternum bifidum	Q7671	
**Brain**		
Anomalies of septum pellucidum		ICD-10 is not available
Arachnoid cyst		ICD-10 is not available
Choroid plexus cyst		ICD-10 is not available
Periventricular leukomalacia		ICD-10 is not available
Single congenital cerebral cyst	Q0461	
**Cardiovascular**		
Absence or hypoplasia of umbilical artery, single umbilical artery	Q270	
Functional or unspecified cardiac murmur		ICD-10 is not available
Patent ductus arteriosus if GA <37 weeks	Q250 if GA <37 weeks	
Patent or persistent foramen ovale	Q2111	
Peripheral pulmonary artery stenosis	Q256 if GA < 37 weeks	
Persistent left superior vena cava	Q261	
Persistent right aortic arch	Q2541	

**Pulmonary**		
Accessory lobe of lung	Q331	
Azygos lobe of lung	Q3310	
Congenital laryngeal stridor	Q314	
Hyperplasia of thymus		ICD-10 is not available
Laryngomalacia	Q314, Q315	
Pleural effusion		ICD-10 is not available
Thymus involution		ICD-10 is not available
Tracheomalacia	Q320	
Vocal cord palsy		ICD-10 is not available
**Gastro-intestinal**		
Abdominal cyst		ICD-10 is not available
Anterior anus		ICD-10 is not available
Congenital cholestasis		ICD-10 is not available
Congenital mesenteric cyst		ICD-10 is not available
Diastasis recti		ICD-10 is not available
Functional gastro-intestinal disorders	Q4021, Q4320, Q4381, Q4382	
Hiatus hernia	Q401	
Inguinal hernia		ICD-10 is not available
Meckel's diverticulum	Q430	
Plica of anus		ICD-10 is not available
Pyloric stenosis	Q400	
Transient choledochal cyst		ICD-10 is not available
Umbilical hernia		ICD-10 is not available
**Renal**		
Hydronephrosis with a pelvis dilatation less than 10 mm		ICD-10 is not available
Hyperplastic and giant kidney	Q633	
Single renal cyst	Q610	
Vesico-ureteral-renal reflux	Q627	
**External genitals**		
Bifid scrotum	Q5521	
Congenital malformation of vulva	Q527	
Curvature of penis		ICD-10 is not available
Cysts of vulva		ICD-10 is not available
Deficient or hooded foreskin		ICD-10 is not available
Developmental ovarian cyst		ICD-10 is not available
Enlarged clitoris		ICD-10 is not available
Fusion of labia	Q525	
Hydrocele of testis		ICD-10 is not available
Hymen imperforatum	Q523	
Hypertrophia of hymen		ICD-10 is not available
Hypoplasia of penis		ICD-10 is not available
Phymosis		ICD-10 is not available
Prominent labia minora		ICD-10 is not available
Retractile testis	Q5520	
Transient ovarian cyst		ICD-10 is not available

Undescended testicle	Q53	
Unspecified ectopic testis		ICD-10 is not available
Vaginal skin tag		ICD-10 is not available
**Other**		
Congenital malformation, unspecified	Q899	
**Chromosomal**		
Balanced translocations or inversions in normal individuals	Q950, Q951	

Annex 4. Observational Study Protocol Amendment (a) Summary: Observational Study to Assess Maternal and Fetal Outcomes Following Exposure to Duloxetine: Denmark and Sweden National Pregnancy Registry Study

Header

Original version	FJ1-MC-B059 Non-interventional PASS Protocol
Updated version	FJ1-MC-B059 Non-interventional PASS Protocol Amendment (a)

Page number as in original Protocol

Page 1

Original version	**Headline**<br>Observational Study to Assess Maternal and Fetal Outcomes Following Exposure to Duloxetine: Denmark, Finland, Norway and Sweden National Pregnancy Registry Study<br><br>**Text below Confidential Information**<br>Non-interventional PASS Protocol Electronically Signed and Approved by Lilly: approval date provided below
Updated version	**Headline**<br>Observational Study to Assess Maternal and Fetal Outcomes Following Exposure to Duloxetine: Denmark and Sweden National Pregnancy Registry Study<br><br>**Text below Confidential Information**<br>Non-interventional PASS Protocol Electronically Signed and Approved by Lilly: 12 October 2017<br>Non-interventional PASS Protocol Amendment (a) Electronically Signed and Approved by Lilly:

Page 2

Original version	**Post-authorization Safety Study (PASS) Information**	
	Country(-ies) of study	Denmark, Sweden, Norway, Finland
Updated version	**Post-authorization Safety Study (PASS) Information**	
	Country(-ies) of study	Denmark, Sweden

Page 4

Revision history inserted.

Page 12-13

Original version	**Section 4 Abstract**<br>• Population<br>Pregnant women included in the Nordic countries' Medical Birth Registries, or with a diagnosis of spontaneous or elective abortion between 2004 and 2015<br>• Data sources<br>Medical Birth Registries and National Patient Registries from the Nordic countries (Denmark, Norway, Sweden and Finland). Information on abortions will be available from Denmark and Finland (induced abortions), and, if national local legislation so permits, from the remaining two countries.<br>• Study size<br>Approximately 1,500 pregnancies exposed to duloxetine during the first trimester, and 300-500 pregnancies exposed in late pregnancy are projected.<br>• Milestones: Analyses are expected to start by 13 July 2018 and will be completed by 4 December 2018.
Updated version	**Section 4 Abstract**<br>• Population<br>Pregnant women included in the Denmark and Sweden's Medical Birth Registries, or with a diagnosis of spontaneous or elective abortion between 2004 and 2015<br>• Data sources<br>Medical Birth Registries and National Patient Registries from Denmark and Sweden. Information on abortions will be available from Denmark, and, if national local legislation so permits, from Sweden.<br>• Study size<br>Approximately 3,000 pregnancies exposed to duloxetine during the first

<table>
<tr><td></td><td>trimester, and 500-1000 pregnancies exposed in late pregnancy are projected in the period from 2004 to 2015.
• Milestones: Analyses are expected to start by 02 September 2018 and will be completed by 26 February 2019.</td></tr>
</table>

Page 15

<table>
<tr><td>Original version</td><td>Section 6 Milestone
<table><tr><th>Milestone</th><th>Planned date</th></tr><tr><td>Start of data collection</td><td>15 February 2018</td></tr><tr><td>End of data collection</td><td>17 September 2018</td></tr><tr><td>Registration in the EU PAS register</td><td>02 August 2017</td></tr><tr><td>Final report of study results</td><td>17 March 2019</td></tr></table></td></tr>
<tr><td>Updated version</td><td>Section 6 Milestone
<table><tr><th>Milestone</th><th>Planned date</th></tr><tr><td>Start of data collection</td><td>30 May 2018</td></tr><tr><td>End of data collection</td><td>31 December 2018</td></tr><tr><td>Registration in the EU PAS register</td><td>02 August 2017</td></tr><tr><td>Final report of study results</td><td>28 March 2019</td></tr></table></td></tr>
</table>

Page 16

Original version	**Section 7.1 Treatment of Depression during Pregnancy**<br>Selective Serotonin Reuptake Inhibitors (SSRI)s are the most commonly used ADs worldwide and in the Nordic countries,[9,10,13] followed by Serotonin and Norepinephrine Reuptake Inhibitors (SNRI)s.[9,14]
Updated version	**Section 7.1 Treatment of Depression during Pregnancy**<br>Selective Serotonin Reuptake Inhibitors (SSRI)s are the most commonly used ADs worldwide and in Denmark and Sweden,[9,10,13] followed by Serotonin and Norepinephrine Reuptake Inhibitors (SNRI)s.[9,14]

Page 17

Original version	**Section 7.2.1. Major Congenital Malformations**<br>In the Nordic countries, approximately 3% of all infants are born with serious birth defects.[63] Deaths due to birth defects is one of the leading cause of infant mortality.
Updated version	**Section 7.2.1. Major Congenital Malformations**<br>In Denmark and Sweden, approximately 3% of all infants are born with serious birth defects.[63] Deaths due to birth defects is one of the leading causes of infant mortality.

Page 18

Original version	**Section 7.2.4 Stillbirths**<br>Furthermore, large cohorts are needed to assess the risk of this rare outcome, with an incidence of 0.2-0.4% in the Nordic countries.[88]
Updated version	**Section 7.2.4 Stillbirths**<br>Furthermore, large cohorts are needed to assess the risk of this rare outcome, with an incidence of 0.3-0.4% in Denmark and Sweden.[88]

Page 21

Original version	**Section 9.1 Study design**<br>The study will be a retrospective observational study based on nationwide registers from 4 Nordic countries: Denmark, Sweden, Norway and Finland. All pregnancies, in the four countries, ending in induced abortion, spontaneous abortions or birth, and their offspring will be included in the cohort.<br>If more recent data than 2015 are available in all Nordic countries they will be included in the study.
Updated version	**Section 9.1 Study design**<br>The study will be a retrospective observational study based on nationwide registers from Denmark and Sweden. All pregnancies, in the two countries, ending in induced abortion, spontaneous abortions or birth, and their offspring will be included in the cohort.<br>If more recent data than 2015 are available in the two countries they will be included in the study.

Page 25

Original version	**Section 9.1.1 Rationale for the design and data source**<br>For these studies, health care utilization databases, such as the Nordic national health registers, are often relied on<br>The Nordic health registers comprise a unique cohort for the study of pregnant women in Europe, due to the registers' size, quality and long follow-up time.<br><br>**Section 9.2.1 Study Population**<br>The basis for all the analyses will be data from the Nordic countries' national birth registers and national patient registries.<br>Base cohort to include all pregnancies ending in a live birth from the national birth registries of the Nordic countries with linked offspring from 2004 to 2015
Updated version	**Section 9.1.1 Rationale for the design and data source**<br>For these studies, health care utilization databases, such as the national health registers, are often relied on<br>The national health registers comprise a unique cohort for the study of pregnant women in Europe, due to the registers' size, quality and long follow-up time.<br><br>**Section 9.2.1 Study Population**<br>The basis for all the analyses will be data from Denmark and Sweden's national birth registers and national patient registries.<br>Base cohort to include all pregnancies ending in a live birth from the national birth registries of the two countries with linked offspring from 2004 to 2015

Page 26

Original version	**Section 9.2.1 Study Population**<br>Base cohort will include pregnancies drawn from the Nordic birth registers with linked offspring<br><br>Base cohort to include all pregnancies drawn from the national birth registries of the Nordic countries with linked offspring from 2004 to 2015
Updated version	**Section 9.2.1 Study Population**<br>Base cohort will include pregnancies drawn from the Danish and Swedish registers

	with linked offspring<br><br>Base cohort to include all pregnancies drawn from the national birth registries of Denmark and Sweden with linked offspring from 2004 to 2015

Page 27

Original version	**Section 9.2.1 Study Population**<br>The study population used for this outcome will highly depend on the availability of data from the Nordic countries and their legislation. It is unclear if abortion data from all the Nordic countries will be accessible.<br>Data from Denmark will be available, and data from Denmark has previously been used to estimate risk of abortion among duloxetine exposed pregnant women.[84] The study only included data 1997 till 2008. Inclusion of data from any other Nordic countries will strengthen the statistical power.<br><br>Base cohort to include all pregnancies from the national birth registries of the Nordic countries with linked offspring and all women with a diagnosis of spontaneous or elective abortion from the national hospital registers, from 2004 to 2015
Updated version	**Section 9.2.1 Study Population**<br>The study population used for this outcome will highly depend on the availability of data from the two countries and their legislation. It is unclear if abortion data from both countries will be accessible.<br>Data from Denmark will be available and has previously been used to estimate risk of abortion among duloxetine exposed pregnant women.[84] The study only included data from 1997 till 2008.<br><br>Base cohort to include all pregnancies from the national birth registries of the two countries with linked offspring and all women with a diagnosis of spontaneous or elective abortion from the national hospital registers, from 2004 to 2015

Page 28

Original version:

Figure 1 Flow diagram showing the composition of the study population for the major congenital malformations outcome

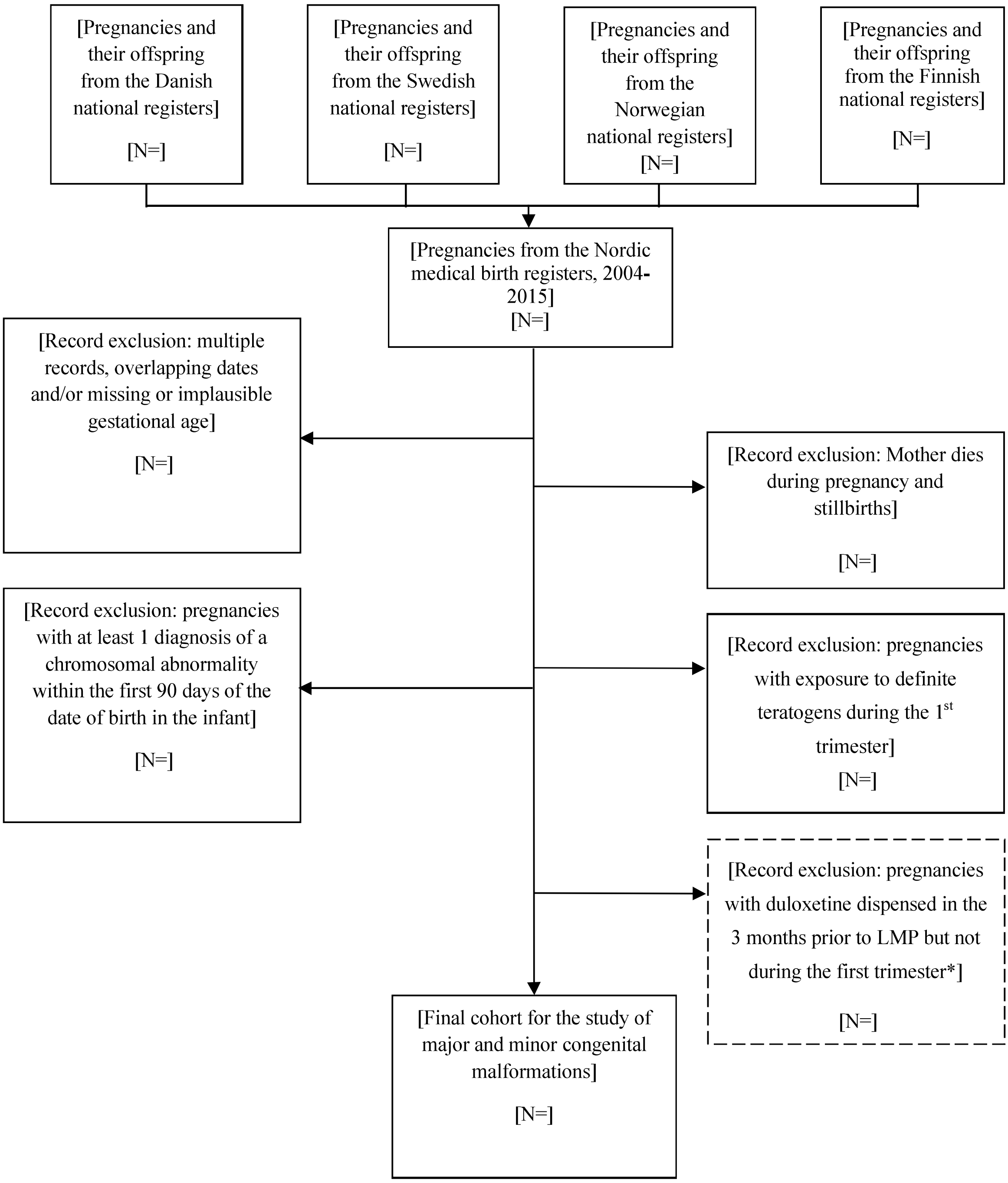

Updated version:

Figure 1 Flow diagram showing the composition of the study population for the major congenital malformations outcome

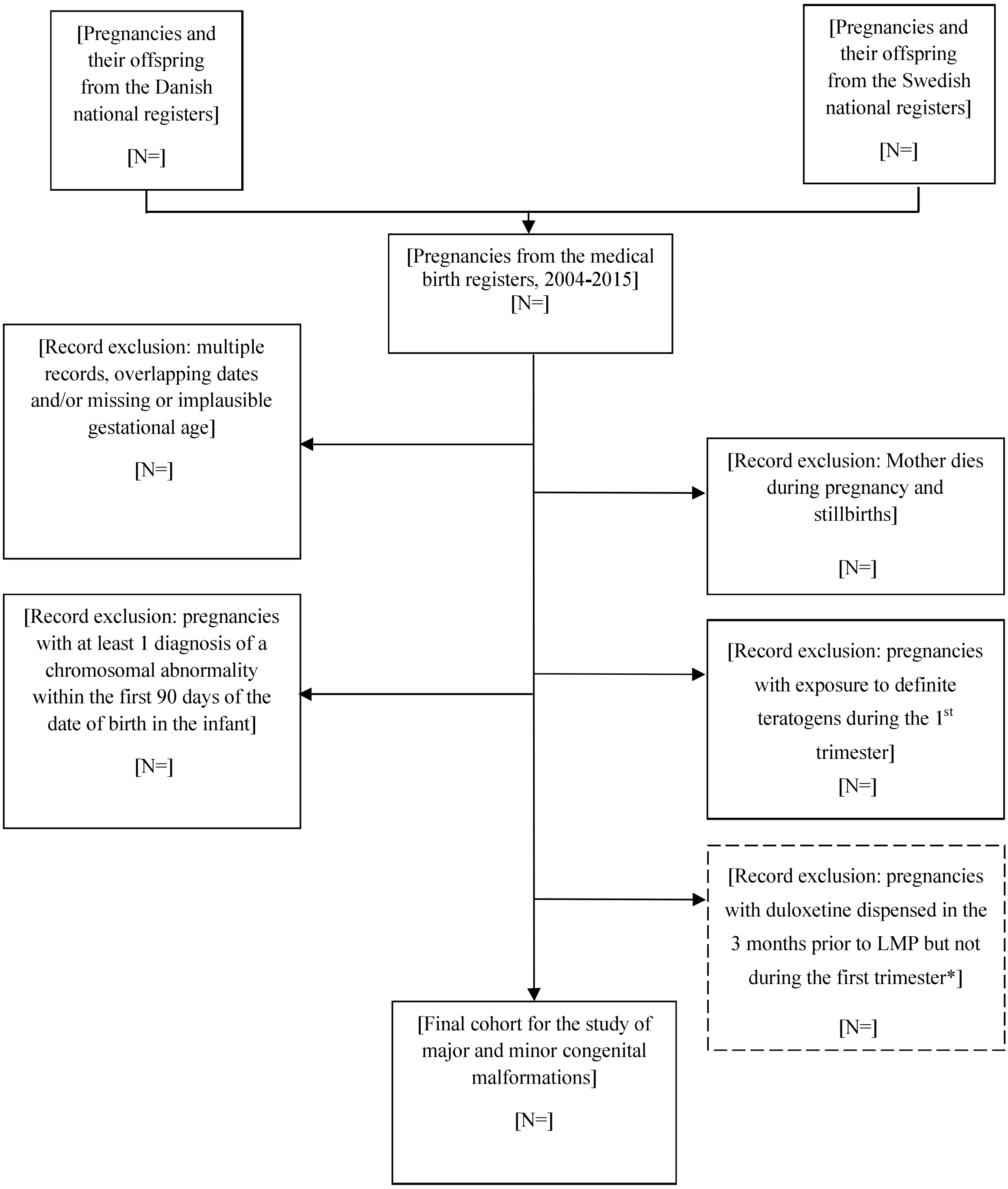

Page 29

Original version:

Figure 2: Flow diagram showing the composition of the study population for the SGA and preterm outcomes

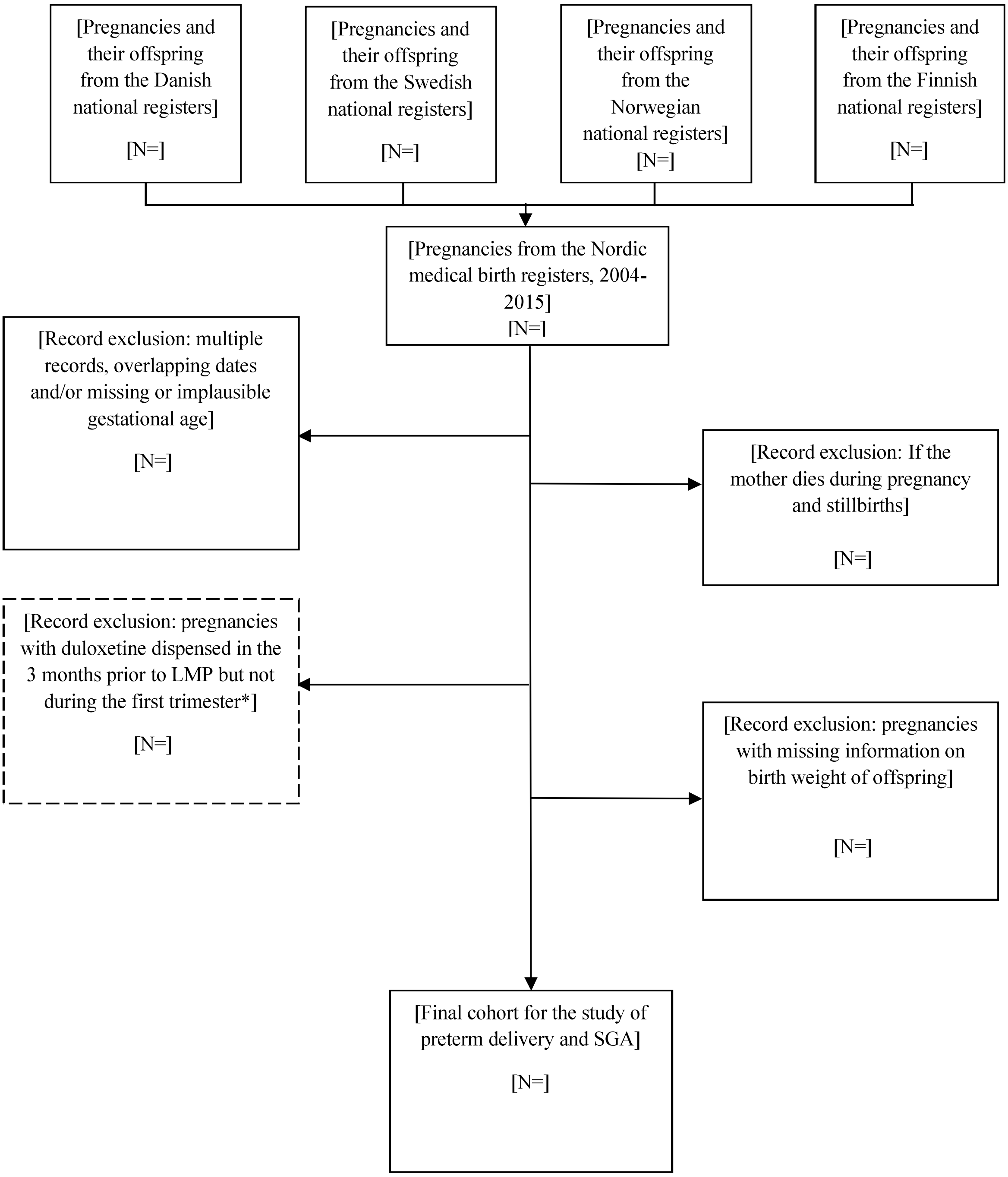

Updated version:

Figure 2: Flow diagram showing the composition of the study population for the SGA and preterm outcomes

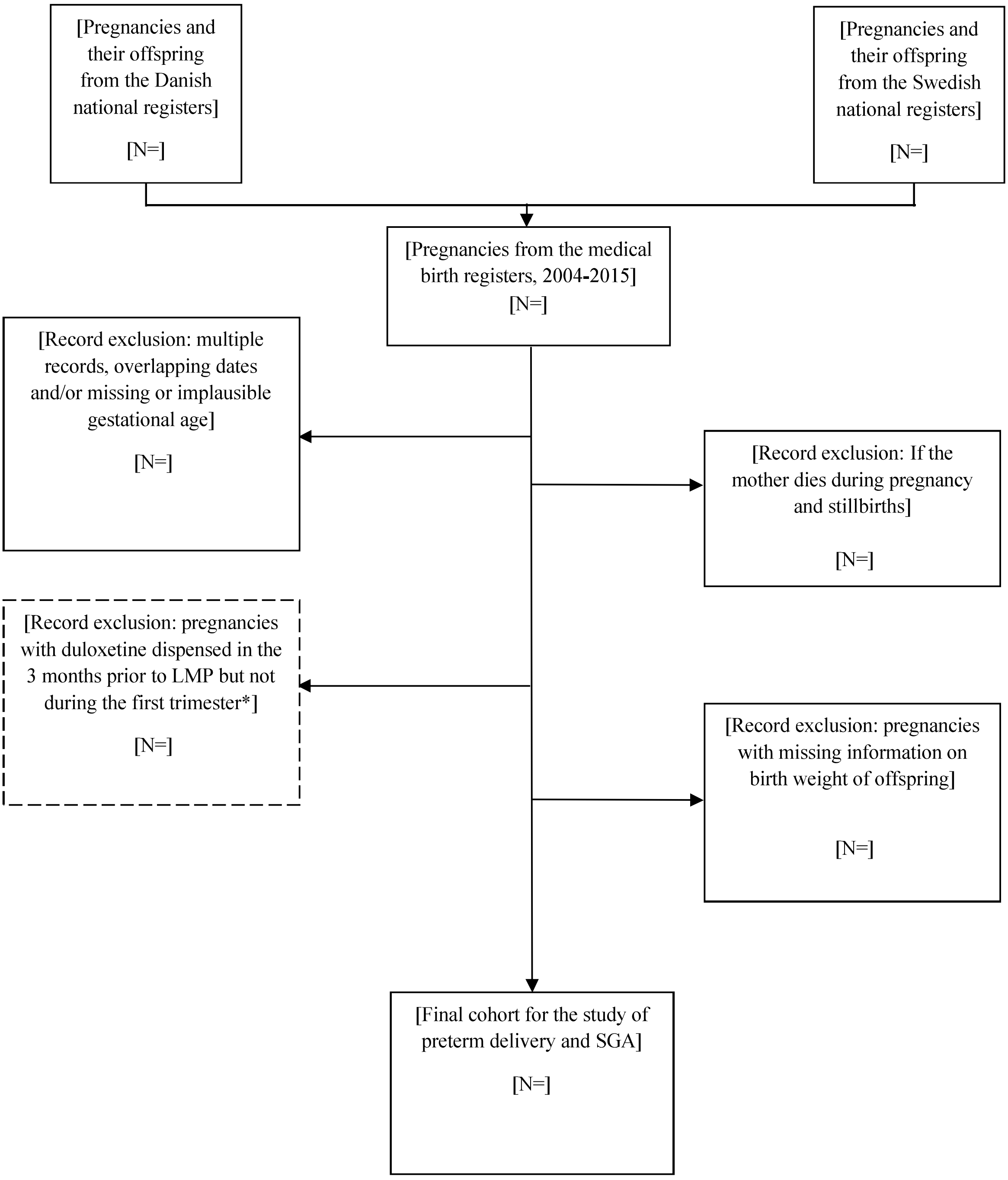

Page 30

Original version:

Figure 3: Flow diagram showing the composition of the study population for the perinatal mortality outcomes

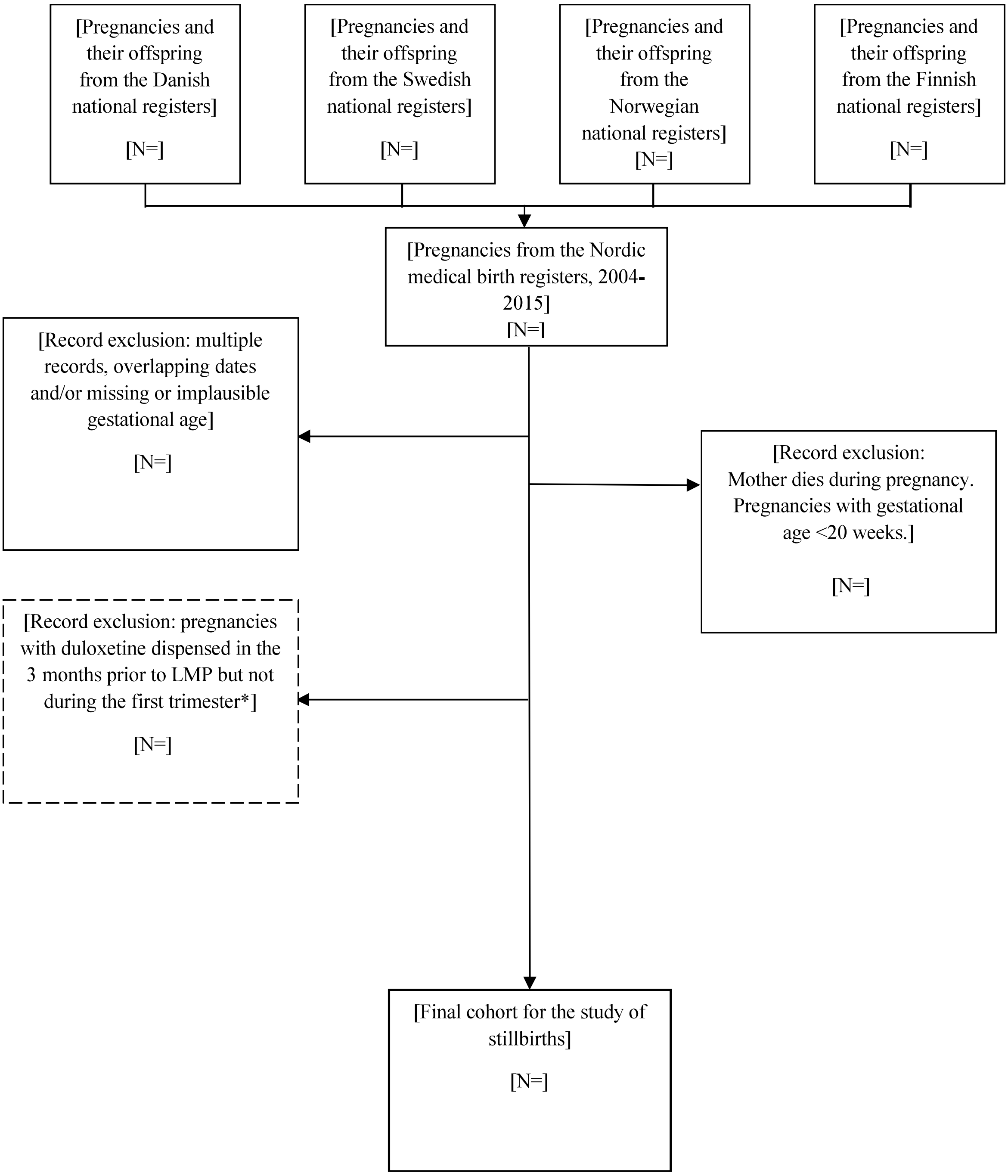

Updated version:

Figure 3: Flow diagram showing the composition of the study population for the perinatal mortality outcomes

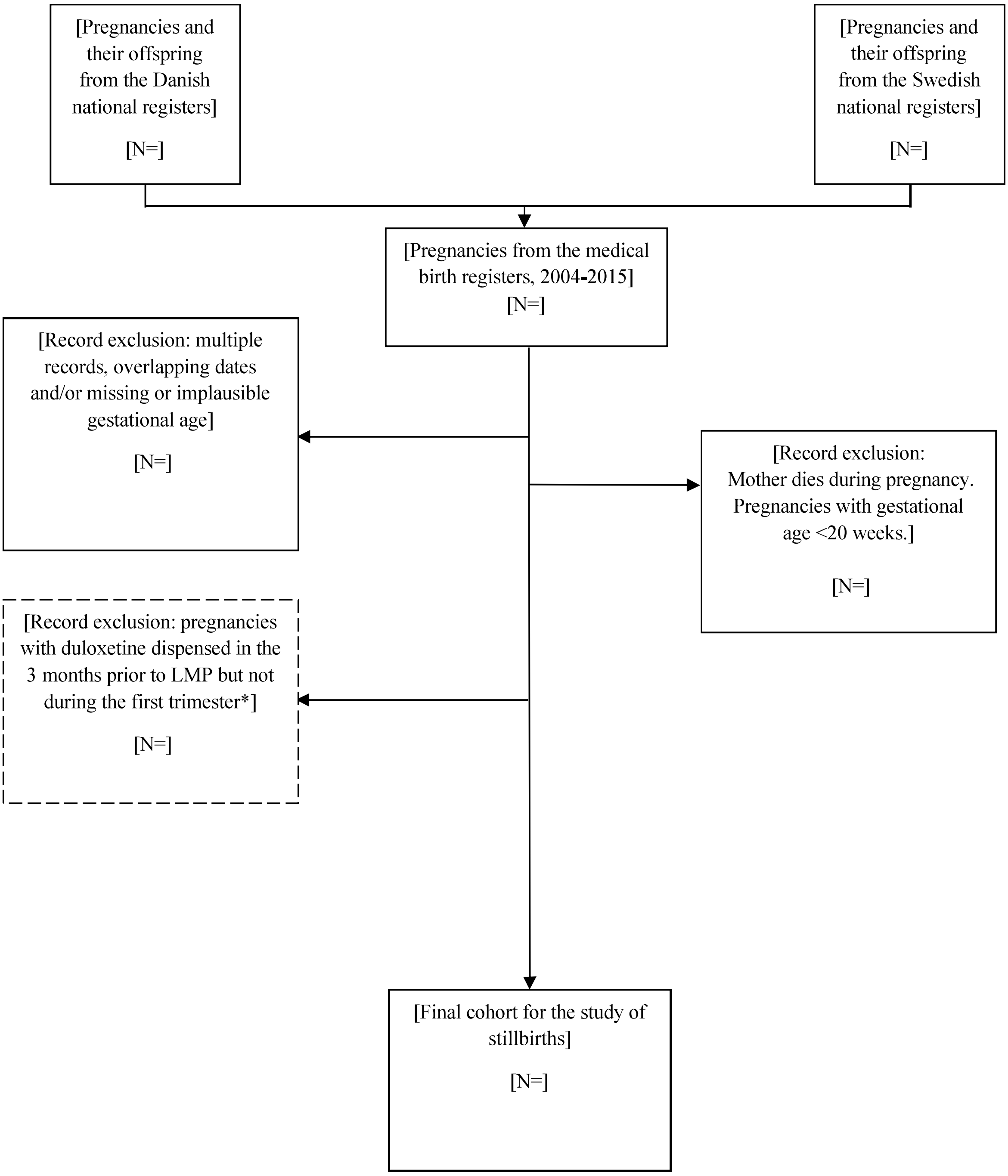

Page 31

Original version:

Figure 4: Flow diagram showing the composition of the study population for the spontaneous and elective abortion outcomes

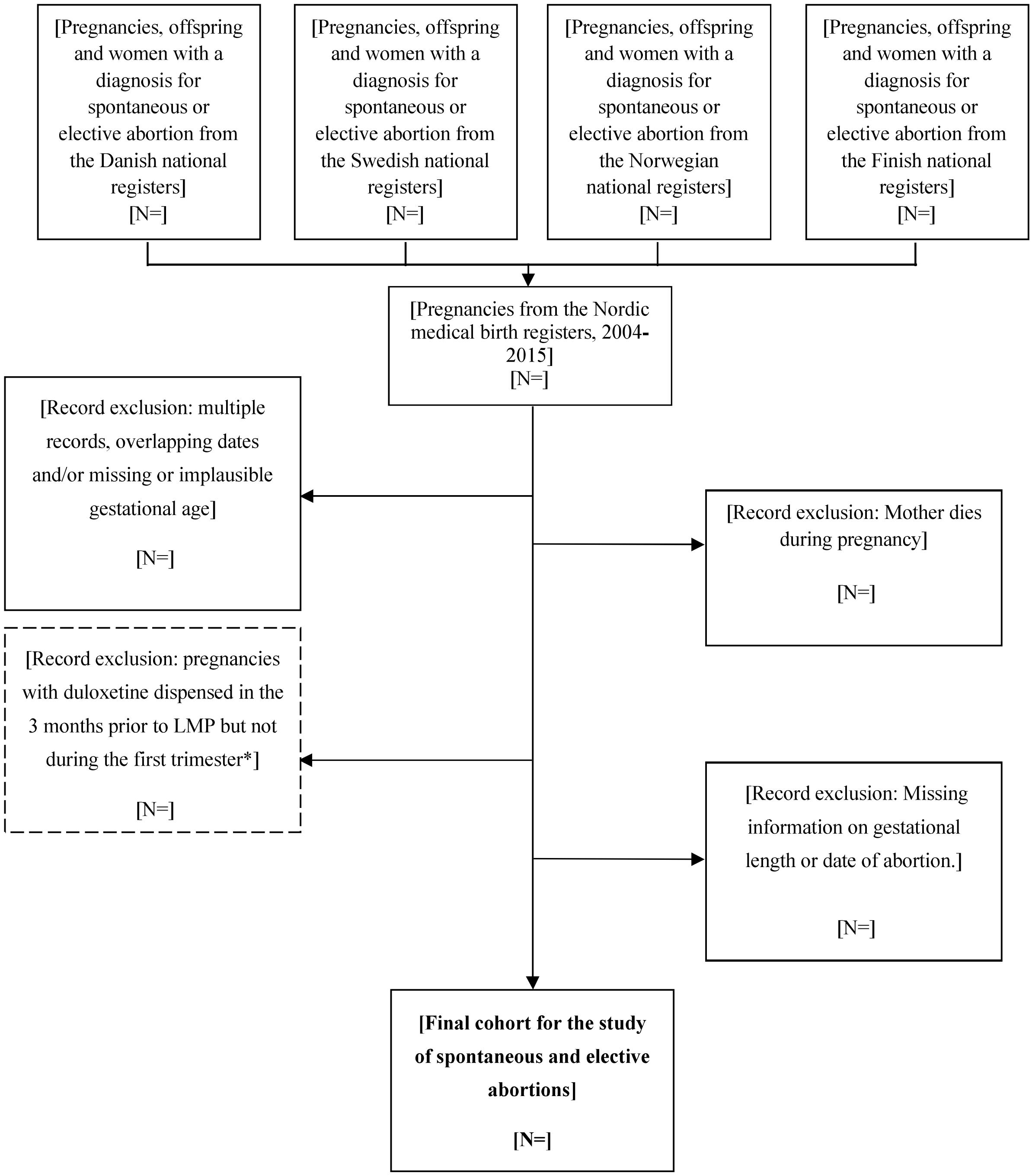

Updated version:

Figure 4: Flow diagram showing the composition of the study population for the spontaneous and elective abortion outcomes

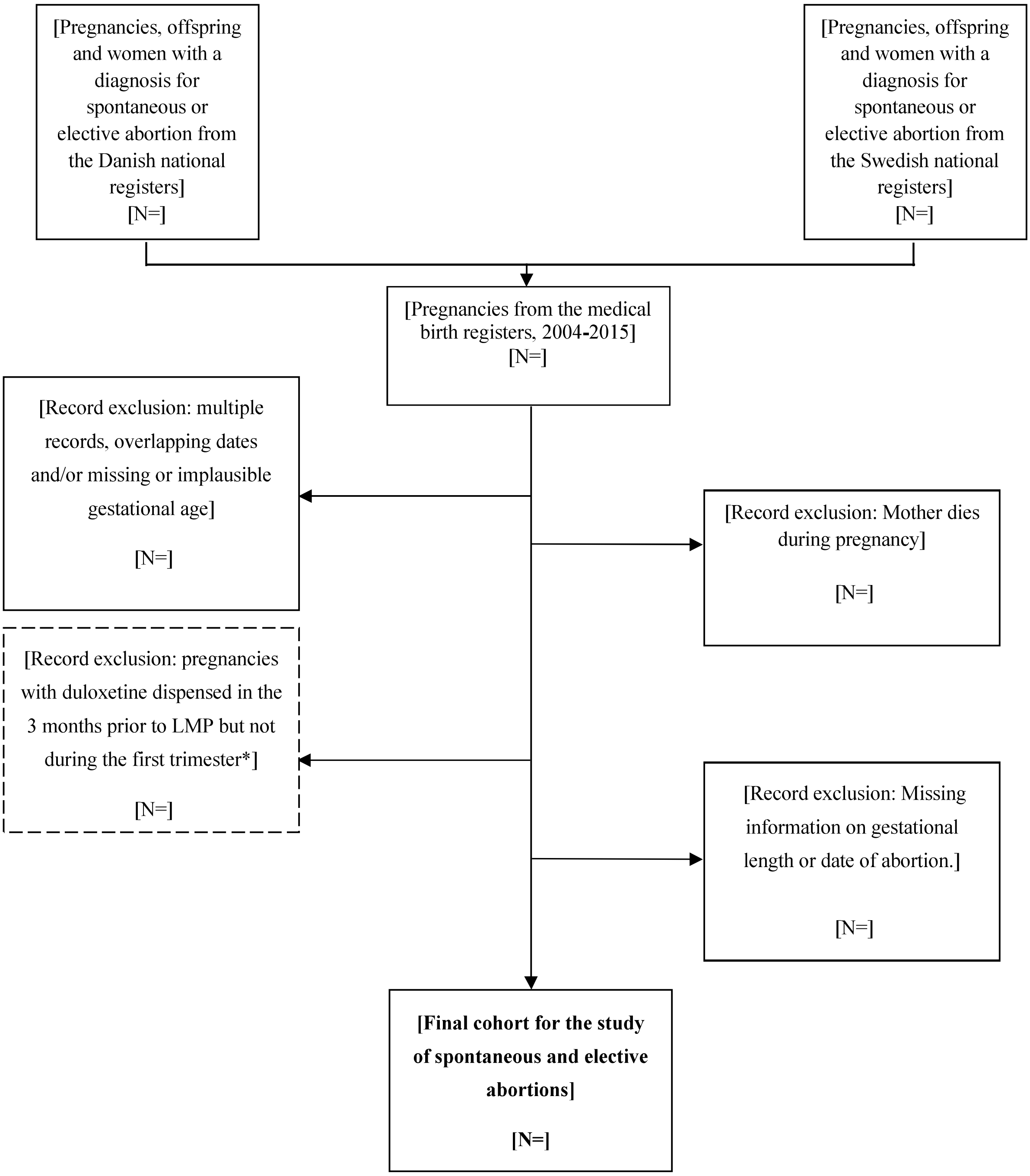

Page 34

Original version	**Section 9.3.3 Covariates**<br>Table 2: Risk factors for study outcome that are unmeasured or poorly measured in the Nordic Registers
Updated version	**Section 9.3.3 Covariates**<br>Table 2: Risk factors for study outcome that are unmeasured or poorly measured in the Danish and Swedish Registers

Page 36

Original version	**Section 9.4.1 The Nordic Health System**<br>Section concerning health system in Denmark, Finland, Norway and Sweden.
Updated version	**Section 9.4.1 The Health System in Denmark and Sweden**<br>Entire section has been updated and is now only focusing on Denmark and Sweden. Hence, Finland and Norway has been deleted from the text.

Page 37

Original version	**Section 9.4.2 Prescription data**<br>Section concerning prescription data in Denmark, Finland, Norway and Sweden.
Updated version	**Section 9.4.2 Prescription data**<br>Entire section has been updated and is now only focusing on Denmark and Sweden. Hence, Finland and Norway has been deleted from the text.

Page 38

Original version	**Section 9.4.3 The Medical Birth Registries**<br>Each of the Nordic countries has kept medical birth registers for decades, all with compulsory notification.<br><br>The international origin of the codes for some main groups created through the registers allows for cross-country research on large populations within the Nordic countries. However, codes for each individual case are assigning on national platforms

	and this may involve minor differences between the countries.<br><br>**Section 9.4.4 Validity**<br><br>Validation of variables for specific studies has been carried out in all Nordic registers, but they cover different periods and have only been applied to selected conditions. Overall these validation studies have found the registers valid and the only few missing values.
Updated version	**Section 9.4.3 The Medical Birth Registries**<br><br>Both countries have kept medical birth registers for decades, all with compulsory notification.<br><br>The international origin of the codes for some main groups created through the registers allows for cross-country research on large populations within the countries. However, codes for each individual case are assigned on national platforms and this may involve minor differences between the countries.<br><br>**Section 9.4.4 Validity**<br><br>Validation of variables for specific studies has been carried out in all registers, but they cover different periods and have only been applied to selected conditions. Overall these validation studies have found the registers valid with only few missing values. [103,104]

Page 38-39

Original version	**Section 9.4.4 Validity, Table 3**<br><br>Table 3: Background information from Nordic Birth Registries with 2011 as an example<br><br>Background information from Denmark, Finland, Norway and Sweden<br><br>Several studies have been validating the quality of different diagnoses.
Updated version	**Section 9.4.4 Validity, Table 3**<br><br>Table 3: Background information from Danish and Swedish Birth Registries with

	2011 as an example<br><br>Background information from Norway and Finland has been deleted. Background information from Sweden updated.<br><br>Several studies have validated the quality of different diagnoses.

Page 38-39

Original version	**Section 9.4.4 Validity, Table 3**<br>There is no reason to believe that the validity of the different outcome variables should have a different level of validity in Sweden.[100]<br><br>Background information from Denmark, Finland, Norway and Sweden
Updated version	**Section 9.4.4 Validity, Table 3**<br>Table 3: Background information from Danish and Swedish Birth Registries with 2011 as an example<br><br>Background information from Norway and Finland has been deleted.

Page 39

Original version	**Section 9.4.4 Validity**<br>There is no reason to believe that the validity of the different outcome variables should have a different level of validity in the other Nordic countries.[100]
Updated version	**Section 9.4.4 Validity**<br>There is no reason to believe that the validity of the different outcome variables should have a different level of validity in Sweden.[100]

Page 40

Original version	**Section 9.4 Data sources, Table 4**<br>List of data sources from Denmark, Finland, Norway and Sweden

Updated version	**Section 9.4 Data sources, Table 4**<br>List of data sources from Norway and Finland has been deleted.

Page 44

Original version	**Section 9.5 Study Size**<br>Study size in relation to Denmark, Finland, Norway and Sweden.
Updated version	**Section 9.5 Study Size**<br>Entire section (including Table 5) has been updated and is now only focusing on the study size for Denmark and Sweden. Hence, Finland and Norway has been excluded.

Page 46

Original version	**Section 9.6 Data Management**<br>Approval from the respective National Data Protection Agencies in the Nordic countries will be required before the start of the study.<br><br>Data from the four Nordic countries will be gathered at SD, as Denmark is the only country not allowing data to cross their border.<br>**Section 9.6.1 Data Collection Schedule**
Updated version	**Section 9.6 Data Management**<br>Approval from the respective National Data Protection Agencies in the two countries will be required before the start of the study.<br><br>Data from the two countries will be gathered at SD, as Denmark is the only country not allowing data to cross their border.<br><br>**Section 9.6.1 Data Collection Schedule** has been deleted**.**

Page 46-47

Updated version	**Section 9.6 Data Management**<br>The numbering of the headlines has been updated throughout section 9.6 Data

	Management as section 9.6.1 Data Collection Schedule has been deleted.

Page 48

Original version	**Section 9.7 Data Analysis**<br>Due to lack of availability from all the Nordic countries data from Denmark will be used to estimate the risk of miscarriage. Information on spontaneous abortions from the remaining Nordic countries will be gathered in accordance with national law.
Updated version	**Section 9.7 Data Analysis**<br>Due to lack of availability, data from Denmark will be used to estimate the risk of miscarriage. Information on spontaneous abortions from Sweden will be gathered in accordance with national law.

Page 49

Original version	**Section 9.9 Limitation of the Research Methods**<br>The Nordic national health registers are unique due to their completeness and follow-up time.
Updated version	**Section 9.9 Limitation of the Research Methods**<br>The national health registers are unique due to their completeness and follow-up time.

Page 50

Original version	**Section 9.9 Limitation of the Research Methods**<br>Illicit drug use is not believed to be a challenge in the studied population in the Nordic countries.<br><br>Since the national health registers from the Nordic countries cover the whole nation there is minimal risk of selection bias.
Updated version	**Section 9.9 Limitation of the Research Methods**<br>Illicit drug use is not believed to be a challenge in the studied population.<br><br>Since the national health registers from Denmark and Sweden cover the whole nation

	there is minimal risk of selection bias.

Page 55

Updated version	**Section 13 References**<br>The references linked to Finland and Norway have been deleted.

Page 76

Original version	**Annex 3. Additional Information**<br>Information on main responsible parties.
Updated version	**Annex 3. Additional Information**<br>Information on country responsible in Finland and Norway deleted.
