# Supplementary material for: Exposure to duloxetine during pregnancy and risk of congenital malformations and stillbirth: A nationwide cohort study in Denmark and Sweden
Source: PLoS Med. 2021 Nov 22;18(11):e1003851. doi: 10.1371/journal.pmed.1003851 (PMC8654175; doi:10.1371/journal.pmed.1003851)
Supplement: S1 Tables — Table A. Minor malformation ICD-10 codes. Table B. ICD-10 codes for major malformation subtypes. Table C. ICD-10, ATC codes, and time periods for comorbidity. Table D. ATC codes for comedication. Table E. Covariates in the models of the primary analyses (the figures shown in the manuscript). Table F. Baseline table for all covariates, major and minor malformation analyses. Before and after propensity score matching. Table G. Baseline table for all covariates, stillbirth analyses. Before and after propensity score matching. Table H. Number of events per thousand pregnancies (95% Wald confidence intervals). Table I. Major malformation, sensitivity analyses. Table J. Minor malformation, sensitivity analyses. Table K. Malformation subtype: Heart defect. Table L. Malformation subtype: Digestive system. Table M. Malformation subtype: Ear, face, and neck. Table N. Malformation subtype: Eye. Table O. Malformation subtype: Genitals. Table P. Malformation subtype: Abdominal wall. Table Q. Malformation subtype: Limb. Table R. Malformation subtype: Nervous system. Table S. Malformation subtype: Orofacial clefts. Table T. Malformation subtype: Respiratory. Table U. Malformation subtype: Urinary tract. Table V. Malformation subtype: Other anomalies/syndromes. Table W. Stillbirth, sensitivity analyses. (DOCX) [file pmed.1003851.s003.docx]

**Supplementary material**

**Exposure to duloxetine during pregnancy and risk of congenital malformations and stillbirth: A nationwide cohort study in Denmark and Sweden**

**Authors**

Mikkel Zöllner Ankarfeldt 1*

Janne Petersen 1, 2

Jon Trærup Andersen 3, 4

Hu Li 5

Stephen Paul Motsko 5

Thomas Fast 6

Simone Møller Hede 6

Espen Jimenez-Solem 1, 3, 4

1 Copenhagen Phase IV Unit (Phase4CPH), Department of Clinical Pharmacology and Center for Clinical Research and Prevention, Copenhagen University Hospital Bispebjerg and Frederiksberg, Copenhagen, Denmark

2 Section of Biostatistics, Department of Public Health, University of Copenhagen, Copenhagen, Denmark

3 Department of Clinical Pharmacology, Copenhagen University Hospital Bispebjerg and Frederiksberg, Copenhagen, Denmark

4 Faculty of Health and Medical Sciences, University of Copenhagen, Copenhagen, Denmark

5 Eli Lilly and Company, Indianapolis, IN, USA

6 Institute of Applied Economics & Health Research, Copenhagen, Denmark

**Contact information for the corresponding author**

mikkelza@gmail.com

Contents

[Table A Minor malformation ICD-10 codes 4](#_Toc86065511)

[Table B ICD-10 codes for major malformation subtypes 6](#_Toc86065512)

[Table C ICD-10, ATC codes, and time periods for comorbidity 7](#_Toc86065513)

[Table D ATC codes for comedication 8](#_Toc86065514)

[Table E Covariates in the models of the primary analyses (the figures shown in the manuscript) 9](#_Toc86065515)

[Table F baseline table for all covariates, major and minor malformation analyses. Before and after propensity score matching. 11](#_Toc86065516)

[Table G baseline table for all covariates, stillbirth analyses. Before and after propensity score matching. 14](#_Toc86065517)

[Table H Number of events per thousand pregnancies (95% Wald confidence intervals) 17](#_Toc86065518)

[Table I Major malformation, sensitivity analyses 18](#_Toc86065519)

[Table J Minor malformation, sensitivity analyses 20](#_Toc86065520)

[Table K Malformation subtype: Heart defect 22](#_Toc86065521)

[Table L Malformation subtype: Digestive system 25](#_Toc86065522)

[Table M Malformation subtype: Ear, face and neck 26](#_Toc86065523)

[Table N Malformation subtype: Eye 27](#_Toc86065524)

[Table O Malformation subtype: Genital 28](#_Toc86065525)

[Table P Malformation subtype: Abdominal wall 29](#_Toc86065526)

[Table Q Malformation subtype: Limb 30](#_Toc86065527)

[Table R Malformation subtype: Nervous system 31](#_Toc86065528)

[Table S Malformation subtype: Oro-facial clefts 32](#_Toc86065529)

[Table T Malformation subtype: Respiratory 33](#_Toc86065530)

[Table U Malformation subtype: Urinary tract 34](#_Toc86065531)

[Table V Malformation subtype: Other anomalies/syndromes 35](#_Toc86065532)

[Table W Stillbirth, sensitivity analyses 36](#_Toc86065533)

# Table A Minor malformation ICD-10 codes

| **EUROCAT Classifications** | **ICD-10 code** |
| --- | --- |
| Compression facies | Q671 |
| Depressions in skull | Q6740 |
| Dolichocephaly | Q672 |
| Dysmorphic face | Q189 |
| Facial asymmetry | Q670 |
| Plagiocephaly – head asymmetry | Q673 |
| Macrocephalus | Q753 |
| Other congenital deformities of skull, face and jaw | Q674 |
| Blue sclera | Q135 |
| Congenital ectropion | Q101 |
| Congenital entropion | Q102 |
| Crocodile tears | Q0782 |
| Hypertelorism | Q752 |
| Other congenital malformations of eyelid | Q103 |
| Stenosis or stricture of lacrimal duct | Q105 |
| Synophrys | Q1880 |
| Accessory auricle, preauricular appendage, tag, or lobule | Q170 |
| Asymmetric size | Q173 |
| Bat ear, prominent ear | Q175 |
| Double lobule | Q170 |
| Lack of helical fold | Q173 |
| Low set ears | Q174 |
| Macrotia | Q171 |
| Microtia | Q172 |
| Posterior angulation | Q173 |
| Preauricular sinus or cyst | Q181 |
| Primitive shape | Q173 |
| Protuberant ears | Q173 |
| Unspecified and minor malformation of ear | Q179 |
| Deviation of nasal septum | Q6741 |
| Dysmorphic nose | Q189 |
| High arched palate | Q3850 |
| Macrocheilia | Q186 |
| Macroglossia | Q382 |
| Macrostomia | Q184 |
| Microcheilia | Q187 |

| **EUROCAT Classifications** | **ICD-10 code** |
| --- | --- |
| Microstomia | Q185 |
| Retrognathia | Q674 |
| Tongue tie or cyst of tongue | Q381 |
| Congenital malformation of face and neck, unspecified | Q189 |
| Other branchial cleft malformations | Q182 |
| Preauricular sinus or cyst | Q181 |
| Sinus, fistula or cyst of branchial | Q180 |
| Torticollis | Q680 |
| Accessory carpal bones | Q7400 |
| Clinodactyly (5th finger) | Q6810 |
| Enlarged or hypertrophic nails | Q845 |
| Single/abnormal palmar crease | Q8280 |
| Clicking hip subluxation or unstable hip | Q653 |
| Clicking hip subluxation or unstable hip | Q654 |
| Clicking hip subluxation or unstable hip | Q655 |
| Clicking hip subluxation or unstable hip | Q656 |
| Clubfoot of postural origin – other congenital deformities of feet | Q668 |
| Congenital deformity of feet, unspecified | Q669 |
| Congenital pes planus | Q665 |
| Enlarged or hypertrophic nails | Q845 |
| Hallux varus – other congenital varus deformities of feet | Q663 |
| Metatarsus varus – other congenital valgus deformities of feet | Q666 |
| Metatarsus varus or metatarsus adductus | Q662 |
| Pes cavus | Q667 |
| Talipes or pes calcaneovalgus | Q664 |
| Accessory nipples | Q833 |
| Mongoloid spot (whites) | Q8252 |
| Neavus flammeus | Q8250 |
| Pigmented naevus – congenital non-neoplastic naevus | Q825 |

**Table A** continued

| **EUROCAT Classifications** | **ICD-10 code** |
| --- | --- |
| Strawberry naevus | Q8251 |
| Absence of rib | Q7660 |
| Accessory rib | Q7662 |
| Cervical rib | Q765 |
| Congenital bowing of femur | Q683 |
| Congenital bowing of fibula and tibia | Q684 |
| Congenital bowing of long bones of leg, unspecified | Q685 |
| Congenital deformity of spine | Q675 |
| Congenital lordosis, postural | Q7643 |
| Depressed sternum | Q676 |
| Genu recurvatum | Q6821 |
| Prominent sternum | Q677 |
| Shieldlike chest, other congenital deformities of chest | Q678 |
| Spina bifida occulta | Q760 |
| Sternum bifidum | Q7671 |
| Single congenital cerebral cyst | Q0461 |
| Absence or hypoplasia of umbilical artery, single umbilical artery | Q270 |
| Patent ductus arteriosus, if GA <37 weeks | Q250 |
| Patent or persistent foramen ovale | Q2111 |
| Peripheral pulmonary artery stenosis, if GA < 37 weeks | Q256 |
| Persistent left superior vena cava | Q261 |
| Persistent right aortic arch | Q2541 |
| Accessory lobe of lung | Q331 |
| Azygos lobe of lung | Q3310 |
| **EUROCAT Classifications** | **ICD-10 code** |
| Congenital laryngeal stridor | Q314 |
| Laryngomalacia | Q314 |
| Laryngomalacia | Q315 |
| Tracheomalacia | Q320 |
| Functional gastro-intestinal disorders | Q4021 |
| Functional gastro-intestinal disorders | Q4320 |
| Functional gastro-intestinal disorders | Q4381 |
| Functional gastro-intestinal disorders | Q4382 |
| Hiatus hernia | Q401 |
| Meckel’s diverticulum | Q430 |
| Pyloric stenosis | Q400 |
| Hyperplastic and giant kidney | Q633 |
| Single renal cyst | Q610 |
| Vesico-ureteral-renal reflux | Q627 |
| Bifid scrotum | Q5521 |
| Congenital malformation of vulva | Q527 |
| Fusion of labia | Q525 |
| Hymen imperforatum | Q523 |
| Retractile testis | Q5520 |
| Undescended testicle | Q53 |
| Congenital malformation, unspecified | Q899 |
| Balanced translocations or inversions in normal individuals | Q950 |
| Balanced translocations or inversions in normal individuals | Q951 |

# Table B ICD-10 codes for major malformation subtypes

| **Major malformation subtype** | **ICD-10** | **Note** |
| --- | --- | --- |
| Nervous system | Q00, Q01, Q02, Q03, Q04, Q05, Q06, Q07 |  |
| Eye | Q10, Q11, Q12, Q13, Q14, Q15 |  |
| Ear, face and neck | Q16, Q17, Q18 |  |
| Congenital heart defects | Q20, Q21, Q22, Q23, Q24, Q25, Q26 | Exclude Q250 or Q256 with gestational age <37 weeks |
| Respiratory | Q300, Q32, Q33, Q34 | Exclude Q336 |
| Oro facial clefts | Q35, Q36, Q37 | Exclude Q000 or Q042 |
| Digestive system | Q38, Q39, Q40, Q41, Q42, Q43, Q44, Q45, Q790 |  |
| Abdominal wall defects | Q792, Q793, Q795 |  |
| Urinary | Q61, Q62, Q63, Q64, Q794 |  |
| Genital | Q50, Q51, Q52, Q54, Q55, Q56 |  |
| Limb | Q65, Q66, Q67, Q68, Q69, Q70, Q71, Q72, Q73, Q74 |  |
| Other anomalies | Q7402, Q77, Q7800, Q782, Q783, Q784, Q785, Q786, Q787, Q788, Q750, Q7980, Q893, Q894, Q80, Q81, Q82, Q8726, Q0435, Q411, Q412, Q418, Q710, Q712, Q713, Q720, Q722, Q723, Q730, Q793, Q795, Q7980, Q206, Q240, Q3381, Q890, Q893, Q86, Q860, Q8680, P350, P351, P371, Q4471, Q6190, Q7484, Q7484, Q751, Q754, Q7581, Q87, Q936, D830 | Skeletal dysplasias  Craniosynostosis  Congenital constriction bands /amniotic band  Situs inversus  Conjoined twins  Congenital skin disorders  VATER/VACTERL  Vascular disruption anomalies  Laterality anomalies  Teratogenic syndromes with malformations  Fetal alcohol syndrome  Valproate syndrome  Maternal infections resulting in malformations  Genetic syndromes microdeletions |

# Table C ICD-10, ATC codes, and time periods for comorbidity

| **Comorbidity** | **ICD-10, up to 5 years prior to LMP** | **ATC, up to 1 year prior to LMP** |
| --- | --- | --- |
| Diabetes | E10, E11, E12, E13, E14 | A10AB01, A10AB04, A10AB05, A10AB06, A10AC01, A10AD01, A10AD04, A10AD05, A10AD06, A10AE04, A10AE05, A10AE06, A10BA02, A10BB01, A10BB03, A10BB07, A10BB09, A10BB12, A10BD07, A10BD08, A10BD09, A10BD10, A10BD11, A10BD13, A10BD15, A10BD16, A10BD20, A10BF, A10BG03, A10BH01, A10BH02, A10BH03, A10BH04, A10BH05, A10BX02, A10BX04, A10BX07, A10BX09, A10BX10, A10BX11, A10BX12, A10BX14, |
| Diabetes during pregnancy | O24 |  |
| Hyper- and hypothyroidism | E05 | H03AA01, H03BA02, H03BB01, H03BB02, H03CA |
| Hypertension | I10, I11, I12, I13, I15 |  |
| Obesity | E66 | A08AB01, A08AA03 |
| Renal failure | N17, N18, N19 |  |
| Depression | F32, F320, F321, F322, F323, F328, F329, F33, F330, F331, F333, F334, F338, F339 |  |
| Affective disorder | F34, F340, F341, F348, F349, F38, F380, F381, F388, F39 |  |
| Anxiety, phobia, OCD | F400, F401, F402, F410, F411, F42 |  |
| Severe stress reaction | F430, F431, F432 |  |
| Stress urinary incontinence | DN393 |  |
| Diabetic peripheral neuropathic pain | E114, E104 |  |

# Table D ATC codes for comedication

| **Medication** | **ATC** |
| --- | --- |
| Antiepileptics | N03 |
| Antihypertensive | C02, C03, C07, C08, C09 |
| Antipsychotics | N05A |
| Anxiolytics | N05B |
| Danazol | G03XA01 |
| Estradiol | G03AA01, G03AA02, G03AA03, G03AA04, G03AA05, G03AA06, G03AA07, G03AA08, G03AA09, G03AA10, G03AA11, G03AA12, G03AA13, G03AA14, G03AA15, G03AA16, G03AB01, G03AB02, G03AB03, G03AB04, G03AB05, G03AB06, G03AB07, G03AB08, G03CA01, G03CA03, G03CA53, L02AA03, V09IX11 |
| Fluconazole | D01AC15, J01RA07, J02AC01 |
| Glucose-lowering | A10 |
| NSAID | M01A |
| Opioids | N02A |
| Thyroid | H03A |
| Triptans | N02CC |
| Steroid (grouped) | - triamcinolone: A01AC01, C05AA12, H02AB08 - dexamethasone: A01AC02, H02AB02 - cortisone: A01AC03, A07EA02, C05AA01, H02AA02, H02AB09, H02AB10, S01BA03 - prednisolone: A07EA01, C05AA04, H02AB04, H02AB06, H02BX01 - budesonide: A07EA06 - mometasone: D07AC13, R01AD09, R03BA07 - betamethasone: H02AB01 - prednisone: H02AB07, H02AB15 - fluticasone: R01AD08 |
| Progesterone (grouped) | - progesterone: G03DA04, G03FA04, G03XB - hydroxyprogesterone: G03DA03, G03FA02, G03AC06, G03DA02, G03FA12, G03FB06, L02AB02 - medroxyprogesterone: G03AC06, G03DA02, G03FA12, G03FB06, L02AB02 |
| Thyroid hormone (grouped) | - antithyroid: H03B - propylthiouracil: H03BA02 - methimazole: H03BB02 |

Comedication is defined as minimum 1 redeemed prescription from 90 days prior to LMP and 140 days post LMP, or the end of the pregnancy, whichever came first. LMP: last menstrual period.

# Table E Covariates in the models of the primary analyses (the figures shown in the manuscript)

| **Major Malformation. Primary analyses** | | |
| --- | --- | --- |
| Comparator | Adjusted analyses | Propensity score matched analyses |
| Duloxetine non-exposed | Adjusted for country, age (grouped), education (grouped), household income, year (grouped), psychiatric hospital, psychiatric outpatient, smoking, previous spontaneous abortion, previous stillbirths, gestational diabetes during index pregnancy, diabetes, hyper- or hypothyroidism, hypertension, obesity, renal failure, depression, affective disorder, anxiety or phobia, severe stress reaction, diabetic peripheral neuropathy, glucose-lowering, antihypertensive, fluconazole, estradiol, thyroid, NSAID, opioids, triptans, antiepileptics, antipsychotics, anxiolytics, corticosteroid (combination), progesterone (combination), antithyroid (combination). | Propensity score based on country, age (grouped), education (grouped), household income, year (grouped), psychiatric hospital, psychiatric outpatient, smoking, previous spontaneous abortion, previous stillbirths, gestational diabetes during index pregnancy, diabetes, hyper- or hypothyroidism, hypertension, obesity, renal failure, depression, affective disorder, anxiety or phobia, severe stress reaction, diabetic peripheral neuropathy, glucose-lowering, antihypertensive, fluconazole, thyroid, NSAID, opioids, triptans, antiepileptics, antipsychotics, anxiolytics, corticosteroid (combination). |
| SSRI-exposed |  |  |
| Venlafaxine-exposed | Adjusted for country, age (grouped), education (grouped), household income, year (grouped), psychiatric hospital, psychiatric outpatient, smoking, previous spontaneous abortion, previous stillbirths, gestational diabetes during index pregnancy, diabetes, hyper- or hypothyroidism, hypertension, obesity, depression, affective disorder, anxiety or phobia, severe stress reaction, glucose-lowering, antihypertensive, thyroid, NSAID, opioids, antiepileptics, antipsychotics, anxiolytics, corticosteroid (combination), progesterone (combination). |  |
| Duloxetine discontinuers | Adjusted for country, age (grouped), household income, year (grouped), psychiatric hospital, psychiatric outpatient, smoking, previous spontaneous abortion, previous stillbirths, gestational diabetes during index pregnancy, diabetes, obesity, depression, affective disorder, anxiety or phobia, severe stress reaction, glucose-lowering, antihypertensive, estradiol, thyroid, NSAID, opioids, antiepileptics, antipsychotics, anxiolytics, corticosteroid (combination), progesterone (combination). |  |
| **Minor malformation. Primary analyses** | | |
| Comparator | Adjusted analyses | Propensity score matched analyses |
| Duloxetine non-exposed | Adjusted for country, age (grouped), education (grouped), household income, year (grouped), psychiatric hospital, psychiatric outpatient, smoking, previous spontaneous abortion, previous stillbirths, gestational diabetes during index pregnancy, diabetes, hyper- or hypothyroidism, hypertension, obesity, renal failure, depression, affective disorder, anxiety or phobia, severe stress reaction, diabetic peripheral neuropathy, glucose-lowering, antihypertensive, fluconazole, estradiol, thyroid, NSAID, opioids, triptans, antiepileptics, antipsychotics, anxiolytics, corticosteroid (combination), progesterone (combination), antithyroid (combination). | Propensity score based on country, age (grouped), education (grouped), household income, year (grouped), psychiatric hospital, psychiatric outpatient, smoking, previous spontaneous abortion, previous stillbirths, gestational diabetes during index pregnancy, diabetes, hyper- or hypothyroidism, hypertension, obesity, renal failure, depression, affective disorder, anxiety or phobia, severe stress reaction, diabetic peripheral neuropathy, glucose-lowering, antihypertensive, fluconazole, thyroid, NSAID, opioids, triptans, antiepileptics, antipsychotics, anxiolytics, corticosteroid (combination). |
| SSRI-exposed |  |  |
| Venlafaxine-exposed | Adjusted for country, age (grouped), education (grouped), household income, year (grouped), psychiatric hospital, psychiatric outpatient, smoking, previous spontaneous abortion, gestational diabetes during index pregnancy, diabetes, hyper- or hypothyroidism, hypertension, obesity, depression, affective disorder, anxiety or phobia, severe stress reaction, glucose-lowering, antihypertensive, fluconazole, thyroid, NSAID, opioids, triptans, antiepileptics, antipsychotics, anxiolytics, corticosteroid (combination), progesterone (combination). |  |
| Duloxetine discontinuers |  |  |
| **Stillbirth. Primary analyses** | | |
| Comparator | Propensity score matched analyses | |
| Duloxetine non-exposed | Propensity score based on country, age (grouped), education (grouped), household income, year (grouped), psychiatric hospital, psychiatric outpatient, smoking, previous spontaneous abortion, previous stillbirths, gestational diabetes during index pregnancy, diabetes, hyper- or hypothyroidism, hypertension, obesity, renal failure, depression, affective disorder, anxiety or phobia, severe stress reaction, diabetic peripheral neuropathy, glucose-lowering, antihypertensive, fluconazole, thyroid, NSAID, opioids, triptans, antiepileptics, antipsychotics, anxiolytics, corticosteroid (combination), antithyroid (combination). | |
| SSRI-exposed |  |  |
| Venlafaxine-exposed |  |  |
| Duloxetine discontinuers |  |  |

SSRI: Selective serotonin reuptake inhibitors.

# Table F baseline table for all covariates, major and minor malformation analyses. Before and after propensity score matching.

| Variable | Value | Duloxetine  Before matching  n=1,512 | Duloxetine vs. duloxetine non-exposed | | | | | Duloxetine vs. SSRI | | | | | Duloxetine vs. venlafaxine | | | | | Duloxetine vs. duloxetine discontinuers | | | | |
| --- | --- | --- | --- | --- | --- | --- | --- | --- | --- | --- | --- | --- | --- | --- | --- | --- | --- | --- | --- | --- | --- | --- |
|  |  |  | Before matching | | After matching | | | Before matching | | After matching | | | Before matching | | After matching | | | Before matching | | After matching | | |
|  |  |  | Duloxetine  non-exposed  n=2,074,652 | Std mean diff. | Duloxetine  n=1,438 | Duloxetine  non-exposed  n=5,751 | Std mean diff. | SSRI  n=39,959 | Std mean diff. | Duloxetine  n=1,437 | SSRI  n=2,874 | Std mean diff. | Venlafaxine  n=5,240 | Std mean diff | Duloxetine  n=1,429 | Venlafaxine  n=1,429 | Std mean diff | Duloxetine discontinuers  n=2,876 | Std mean diff | Duloxetine  n=1,434 | Discontinuers  n=1,435 | Std mean diff |
| Age, continuous | Mean (interquartile range) | 30.8  (27.2; 35.1) | 30.2  (26.7; 33.7) | 0.15 | 30.7  (27.2; 35.0) | 30.8  (26.8; 34.7) | 0.04 | 30.6  (26.8; 34.4) | 0.08 | 30.7  (27.2; 35.0) | 30.4  (26.6; 34.5) | 0.08 | 30.5  (26.4; 34.5) | 0.09 | 30.7  (27.2; 35.0) | 30.4  (26.3; 34.6) | 0.07 | 30.3  (26.6; 34.3) | 0.11 | 30.7  (27.2; 35.0) | 30.5  (26.7; 34.7) | 0.04 |
| Age, grouped | 18-24 years | 231  (15.3%) | 323541  (15.6%) | 0.16 | 222  (15.4%) | 888  (15.4%) | 0.05 | 6399  (16.0%) | 0.06 | 222  (15.4%) | 469  (16.3%) | 0.04 | 947  (18.1%) | 0.09 | 222  (15.5%) | 240  (16.8%) | 0.03 | 470  (16.3%) | 0.07 | 222  (15.5%) | 230  (16.0%) | 0.05 |
|  | 25-29 years | 447  (29.6%) | 684195  (33.0%) |  | 429  (29.8%) | 1659  (28.8%) |  | 11740  (29.4%) |  | 428  (29.8%) | 887  (30.9%) |  | 1488  (28.4%) |  | 424  (29.7%) | 429  (30.0%) |  | 914  (31.8%) |  | 427  (29.8%) | 435  (30.3%) |  |
|  | 30-34 years | 379  (25.1%) | 591115  (28.5%) |  | 359  (25.0%) | 1532  (26.6%) |  | 10855  (27.2%) |  | 359  (25.0%) | 704  (24.5%) |  | 1359  (25.9%) |  | 356  (24.9%) | 364  (25.5%) |  | 710  (24.7%) |  | 359  (25.0%) | 336  (23.4%) |  |
|  | 35-60 years | 455  (30.1%) | 475801  (22.9%) |  | 428  (29.8%) | 1672  (29.1%) |  | 10965  (27.4%) |  | 428  (29.8%) | 814  (28.3%) |  | 1446  (27.6%) |  | 427  (29.9%) | 396  (27.7%) |  | 782  (27.2%) |  | 426  (29.7%) | 434  (30.2%) |  |
| BMI, continuous | Mean (interquartile range) | 25.4  (22.3; 30.0) | 23.5  (21.3; 26.7) | 0.38 | 25.4  (22.3; 29.8) | 24.6  (21.9; 28.9) | 0.11 | 24.3  (21.8; 28.3) | 0.18 | 25.4  (22.3; 29.9) | 24.8  (22.0; 29.1) | 0.10 | 25.1  (22.1; 29.4) | 0.05 | 25.4  (22.3; 29.8) | 25.2  (22.3; 29.4) | 0.04 | 25.0  (22.2; 29.7) | 0.04 | 25.4  (22.3; 29.8) | 25.5  (22.5; 29.8) | -0.02 |
| BMI, grouped | BMI <21 | 210  (14.9%) | 422401  (21.7%) | 0.39 | 206  (14.9%) | 953  (17.3%) | 0.12 | 6878  (18.3%) | 0.17 | 206  (14.9%) | 471  (16.9%) | 0.09 | 792  (16.1%) | 0.04 | 206  (15.0%) | 207  (15.1%) | 0.03 | 370  (13.8%) | 0.08 | 206  (14.9%) | 177  (12.8%) | 0.06 |
|  | BMI 21-26< | 555  (39.3%) | 953277  (49.1%) |  | 545  (39.5%) | 2354  (42.6%) |  | 16598  (44.2%) |  | 545  (39.5%) | 1182  (42.3%) |  | 1994  (40.5%) |  | 543  (39.6%) | 560  (40.8%) |  | 1154  (43.0%) |  | 545  (39.6%) | 577  (41.6%) |  |
|  | BMI 26-30< | 294  (20.8%) | 323197  (16.6%) |  | 287  (20.8%) | 1047  (19.0%) |  | 7120  (18.9%) |  | 286  (20.7%) | 537  (19.2%) |  | 1010  (20.5%) |  | 283  (20.6%) | 292  (21.3%) |  | 516  (19.2%) |  | 287  (20.8%) | 295  (21.3%) |  |
|  | BMI >=30 | 353  (25.0%) | 243826  (12.6%) |  | 342  (24.8%) | 1169  (21.2%) |  | 6977  (18.6%) |  | 342  (24.8%) | 604  (21.6%) |  | 1133  (23.0%) |  | 340  (24.8%) | 312  (22.8%) |  | 644  (24.0%) |  | 340  (24.7%) | 337  (24.3%) |  |
| Household income | income quartile1 | 569  (37.8%) | 458644  (22.2%) | 0.41 | 549  (38.2%) | 2189  (38.1%) | 0.00 | 12032  (30.3%) | 0.19 | 548  (38.1%) | 1115  (38.8%) | 0.03 | 1895  (36.4%) | 0.05 | 545  (38.1%) | 569  (39.8%) | 0.05 | 1051  (36.7%) | 0.05 | 549  (38.3%) | 560  (39.0%) | 0.05 |
|  | income quartile2 | 391  (25.9%) | 514943  (25.0%) |  | 370  (25.7%) | 1481  (25.8%) |  | 10306  (26.0%) |  | 370  (25.7%) | 757  (26.3%) |  | 1418  (27.3%) |  | 367  (25.7%) | 369  (25.8%) |  | 815  (28.5%) |  | 367  (25.6%) | 346  (24.1%) |  |
|  | income quartile3 | 317  (21.0%) | 547822  (26.6%) |  | 300  (20.9%) | 1222  (21.2%) |  | 9760  (24.6%) |  | 300  (20.9%) | 610  (21.2%) |  | 1106  (21.3%) |  | 298  (20.9%) | 280  (19.6%) |  | 597  (20.9%) |  | 300  (20.9%) | 317  (22.1%) |  |
|  | income quartile4 | 230  (15.3%) | 540500  (26.2%) |  | 219  (15.2%) | 859  (14.9%) |  | 7557  (19.1%) |  | 219  (15.2%) | 392  (13.6%) |  | 784  (15.1%) |  | 219  (15.3%) | 211  (14.8%) |  | 399  (13.9%) |  | 218  (15.2%) | 212  (14.8%) |  |
| Education | < 11 years | 341  (22.7%) | 252162  (12.3%) | 0.40 | 327  (22.7%) | 1462  (25.4%) | 0.05 | 7390  (18.6%) | 0.19 | 327  (22.8%) | 685  (23.8%) | 0.03 | 1221  (23.5%) | 0.06 | 324  (22.7%) | 309  (21.6%) | 0.03 | 714  (25.0%) | 0.06 | 325  (22.7%) | 301  (21.0%) | 0.05 |
|  | 11-15 years | 806  (53.7%) | 1000210  (48.8%) |  | 771  (53.6%) | 3010  (52.3%) |  | 19650  (49.5%) |  | 770  (53.6%) | 1540  (53.6%) |  | 2641  (50.9%) |  | 765  (53.5%) | 774  (54.2%) |  | 1452  (50.8%) |  | 770  (53.7%) | 791  (55.1%) |  |
|  | >16 years | 355  (23.6%) | 797606  (38.9%) |  | 340  (23.6%) | 1279  (22.2%) |  | 12630  (31.8%) |  | 340  (23.7%) | 649  (22.6%) |  | 1326  (25.6%) |  | 340  (23.8%) | 346  (24.2%) |  | 692  (24.2%) |  | 339  (23.6%) | 343  (23.9%) |  |
| Smoking | YES | 301  (20.7%) | 179398  (9.0%) | 0.34 | 298  (20.7%) | 1339  (23.3%) | -0.06 | 6640  (17.2%) | 0.09 | 297  (20.7%) | 570  (19.8%) | 0.02 | 1303  (25.6%) | -0.12 | 296  (20.7%) | 272  (19.0%) | 0.04 | 578  (20.9%) | -0.01 | 296  (20.6%) | 285  (19.9%) | 0.02 |
| Data source | Sweden | 1010  (66.8%) | 1324668  (63.9%) | 0.06 | 959  (66.7%) | 3900  (67.8%) | -0.02 | 25975  (65.0%) | 0.04 | 958  (66.7%) | 1881  (65.4%) | 0.03 | 2948  (56.3%) | 0.22 | 950  (66.5%) | 960  (67.2%) | -0.01 | 1821  (63.3%) | 0.07 | 955  (66.6%) | 950  (66.2%) | 0.01 |
| Previous stillbirth | YES | 6  (0.4%) | 10401  (0.5%) | -0.02 | 6  (0.4%) | 76  (1.3%) | -0.10 | 250  (0.6%) | -0.03 | 6  (0.4%) | 8  (0.3%) | 0.02 | 22  (0.4%) | -0.00 | 6  (0.4%) | 6  (0.4%) | 0.00 | 17  (0.6%) | -0.03 | 6  (0.4%) | 5  (0.3%) | 0.01 |
| Parity | 0 | 711  (47.2%) | 943927  (45.7%) | 0.25 | 663  (46.3%) | 2502  (43.6%) | 0.11 | 18711  (47.0%) | 0.17 | 663  (46.3%) | 1337  (46.7%) | 0.16 | 2546  (48.7%) | 0.10 | 660  (46.4%) | 689  (48.3%) | 0.06 | 1429  (49.8%) | 0.06 | 661  (46.3%) | 714  (49.8%) | 0.09 |
|  | 1 | 401  (26.6%) | 747860  (36.2%) |  | 390  (27.2%) | 1845  (32.2%) |  | 12997  (32.6%) |  | 389  (27.2%) | 915  (31.9%) |  | 1541  (29.5%) |  | 387  (27.2%) | 391  (27.4%) |  | 734  (25.6%) |  | 388  (27.2%) | 370  (25.8%) |  |
|  | 2 | 254  (16.9%) | 269308  (13.0%) |  | 244  (17.0%) | 846  (14.8%) |  | 5486  (13.8%) |  | 244  (17.1%) | 401  (14.0%) |  | 749  (14.3%) |  | 242  (17.0%) | 217  (15.2%) |  | 451  (15.7%) |  | 244  (17.1%) | 229  (16.0%) |  |
|  | >2 | 140  (9.3%) | 105852  (5.1%) |  | 135  (9.4%) | 541  (9.4%) |  | 2618  (6.6%) |  | 135  (9.4%) | 213  (7.4%) |  | 392  (7.5%) |  | 134  (9.4%) | 130  (9.1%) |  | 253  (8.8%) |  | 135  (9.5%) | 120  (8.4%) |  |
| Prev. spontaneous abortions | 0 | 1186  (78.4%) | 1654343  (79.7%) | 0.09 | 1126  (78.3%) | 4273  (74.3%) | 0.10 | 31098  (77.8%) | 0.05 | 1125  (78.3%) | 2299  (80.0%) | 0.05 | 4206  (80.3%) | 0.09 | 1117  (78.2%) | 1151  (80.5%) | 0.07 | 2205  (76.7%) | 0.07 | 1122  (78.2%) | 1134  (79.0%) | 0.03 |
|  | 1 | 238  (15.7%) | 335312  (16.2%) |  | 228  (15.9%) | 1045  (18.2%) |  | 6867  (17.2%) |  | 228  (15.9%) | 432  (15.0%) |  | 815  (15.6%) |  | 228  (16.0%) | 196  (13.7%) |  | 511  (17.8%) |  | 228  (15.9%) | 218  (15.2%) |  |
|  | 2+ | 88  (5.8%) | 84997  (4.1%) |  | 84  (5.8%) | 433  (7.5%) |  | 1994  (5.0%) |  | 84  (5.8%) | 143  (5.0%) |  | 219  (4.2%) |  | 84  (5.9%) | 82  (5.7%) |  | 160  (5.6%) |  | 84  (5.9%) | 83  (5.8%) |  |
| Outpatient visits, grouped | 0 | 1390  (91.9%) | 1988265  (95.8%) | 0.17 | 1325  (92.1%) | 5246  (91.2%) | 0.06 | 37333  (93.4%) | 0.11 | 1324  (92.1%) | 2632  (91.6%) | 0.00 | 4877  (93.1%) | 0.04 | 1316  (92.1%) | 1341  (93.8%) | 0.09 | 2583  (89.8%) | 0.08 | 1322  (92.2%) | 1284  (89.5%) | 0.13 |
|  | 1 | 89  (5.9%) | 60078  (2.9%) |  | 83  (5.8%) | 318  (5.5%) |  | 1777  (4.4%) |  | 83  (5.8%) | 163  (5.7%) |  | 267  (5.1%) |  | 83  (5.8%) | 63  (4.4%) |  | 198  (6.9%) |  | 82  (5.7%) | 95  (6.6%) |  |
|  | >1 | 33  (2.2%) | 26309  (1.3%) |  | 30  (2.1%) | 187  (3.3%) |  | 849  (2.1%) |  | 30  (2.1%) | 79  (2.7%) |  | 96  (1.8%) |  | 30  (2.1%) | 25  (1.7%) |  | 95  (3.3%) |  | 30  (2.1%) | 56  (3.9%) |  |
| Hospital, grouped | 0 | 1286  (85.1%) | 1924859  (92.8%) | 0.29 | 1230  (85.5%) | 4920  (85.6%) | 0.00 | 35722  (89.4%) | 0.12 | 1230  (85.6%) | 2447  (85.1%) | 0.05 | 4487  (85.6%) | 0.05 | 1223  (85.6%) | 1232  (86.2%) | 0.06 | 2495  (86.8%) | 0.07 | 1228  (85.6%) | 1242  (86.6%) | 0.08 |
|  | 1 | 152  (10.1%) | 115773  (5.6%) |  | 141  (9.8%) | 564  (9.8%) |  | 2985  (7.5%) |  | 141  (9.8%) | 283  (9.8%) |  | 509  (9.7%) |  | 140  (9.8%) | 126  (8.8%) |  | 243  (8.4%) |  | 140  (9.8%) | 111  (7.7%) |  |
|  | >1 | 74  (4.9%) | 34020  (1.6%) |  | 67  (4.7%) | 267  (4.6%) |  | 1252  (3.1%) |  | 66  (4.6%) | 144  (5.0%) |  | 244  (4.7%) |  | 66  (4.6%) | 71  (5.0%) |  | 138  (4.8%) |  | 66  (4.6%) | 82  (5.7%) |  |
| Emergency depart., grouped | 0 | 1430  (94.6%) | 2007115  (96.7%) | 0.20 | 1358  (94.4%) | 5491  (95.5%) | 0.06 | 37719  (94.4%) | 0.05 | 1357  (94.4%) | 2692  (93.7%) | 0.08 | 4854  (92.6%) | 0.10 | 1349  (94.4%) | 1337  (93.6%) | 0.08 | 2696  (93.7%) | 0.05 | 1354  (94.4%) | 1350  (94.1%) | 0.00 |
|  | 1 | 51  (3.4%) | 53990  (2.6%) |  | 50  (3.5%) | 161  (2.8%) |  | 1554  (3.9%) |  | 50  (3.5%) | 115  (4.0%) |  | 262  (5.0%) |  | 50  (3.5%) | 63  (4.4%) |  | 113  (3.9%) |  | 50  (3.5%) | 49  (3.4%) |  |
|  | >1 | 31  (2.1%) | 13547  (0.7%) |  | 30  (2.1%) | 99  (1.7%) |  | 686  (1.7%) |  | 30  (2.1%) | 67  (2.3%) |  | 124  (2.4%) |  | 30  (2.1%) | 29  (2.0%) |  | 67  (2.3%) |  | 30  (2.1%) | 36  (2.5%) |  |
| Psychiatric outpatient, grouped | 0 | 1043  (69.0%) | 2046478  (98.6%) | 0.88 | 998  (69.4%) | 3963  (68.9%) | -0.01 | 33236  (83.2%) | 0.34 | 998  (69.5%) | 2016  (70.1%) | 0.02 | 3993  (76.2%) | 0.16 | 996  (69.7%) | 969  (67.8%) | -0.04 | 2055  (71.5%) | 0.05 | 994  (69.3%) | 1019  (71.0%) | 0.04 |
|  | 1+ | 469  (31.0%) | 28174  (1.4%) |  | 440  (30.6%) | 1788  (31.1%) |  | 6723  (16.8%) |  | 439  (30.5%) | 858  (29.9%) |  | 1247  (23.8%) |  | 433  (30.3%) | 460  (32.2%) |  | 821  (28.5%) |  | 440  (30.7%) | 416  (29.0%) |  |
| Psychiatric hospital, grouped | 0 | 1416  (93.7%) | 2070449  (99.8%) | 0.35 | 1351  (93.9%) | 5470  (95.1%) | 0.05 | 39020  (97.7%) | 0.20 | 1351  (94.0%) | 2716  (94.5%) | 0.02 | 4981  (95.1%) | 0.06 | 1344  (94.1%) | 1353  (94.7%) | 0.03 | 2731  (95.0%) | 0.06 | 1348  (94.0%) | 1345  (93.7%) | -0.01 |
|  | 1+ | 96  (6.3%) | 4203  (0.2%) |  | 87  (6.1%) | 281  (4.9%) |  | 939  (2.3%) |  | 86  (6.0%) | 158  (5.5%) |  | 259  (4.9%) |  | 85  (5.9%) | 76  (5.3%) |  | 145  (5.0%) |  | 86  (6.0%) | 90  (6.3%) |  |
| Gestational diabetes  during index pregnancy | YES | 88  (5.8%) | 50587  (2.4%) | 0.17 | 81  (5.6%) | 472  (8.2%) | -0.10 | 1496  (3.7%) | 0.10 | 81  (5.6%) | 168  (5.8%) | -0.01 | 272  (5.2%) | 0.03 | 80  (5.6%) | 76  (5.3%) | 0.01 | 151  (5.3%) | 0.02 | 81  (5.6%) | 84  (5.9%) | -0.01 |
| Diabetes | YES | 40  (2.6%) | 25400  (1.2%) | 0.10 | 36  (2.5%) | 287  (5.0%) | -0.13 | 783  (2.0%) | 0.05 | 36  (2.5%) | 59  (2.1%) | 0.03 | 121  (2.3%) | 0.02 | 36  (2.5%) | 26  (1.8%) | 0.05 | 71  (2.5%) | 0.01 | 36  (2.5%) | 30  (2.1%) | 0.03 |
| Hyper or hypothyroidism | YES | 100  (6.6%) | 56107  (2.7%) | 0.19 | 96  (6.7%) | 517  (9.0%) | -0.09 | 2011  (5.0%) | 0.07 | 96  (6.7%) | 173  (6.0%) | 0.03 | 262  (5.0%) | 0.07 | 95  (6.6%) | 104  (7.3%) | -0.02 | 135  (4.7%) | 0.08 | 95  (6.6%) | 92  (6.4%) | 0.01 |
| Hypertension | YES | 14  (0.9%) | 6766  (0.3%) | 0.08 | 13  (0.9%) | 107  (1.9%) | -0.08 | 240  (0.6%) | 0.04 | 13  (0.9%) | 26  (0.9%) | 0.00 | 41  (0.8%) | 0.02 | 13  (0.9%) | 8  (0.6%) | 0.04 | 21  (0.7%) | 0.02 | 12  (0.8%) | 15  (1.0%) | -0.02 |
| Obesity | YES | 111  (7.3%) | 63686  (3.1%) | 0.19 | 108  (7.5%) | 502  (8.7%) | -0.04 | 2059  (5.2%) | 0.09 | 108  (7.5%) | 214  (7.4%) | 0.00 | 363  (6.9%) | 0.02 | 107  (7.5%) | 93  (6.5%) | 0.04 | 200  (7.0%) | 0.02 | 106  (7.4%) | 102  (7.1%) | 0.01 |
| Depression | YES | 517  (34.2%) | 39131  (1.9%) | 0.93 | 486  (33.8%) | 2082  (36.2%) | -0.05 | 7326  (18.3%) | 0.37 | 485  (33.8%) | 1002  (34.9%) | -0.02 | 1324  (25.3%) | 0.20 | 478  (33.4%) | 509  (35.6%) | -0.05 | 812  (28.2%) | 0.13 | 482  (33.6%) | 477  (33.2%) | 0.01 |
| Affective disorder | YES | 53  (3.5%) | 3167  (0.2%) | 0.25 | 50  (3.5%) | 151  (2.6%) | 0.05 | 553  (1.4%) | 0.14 | 50  (3.5%) | 86  (3.0%) | 0.03 | 120  (2.3%) | 0.07 | 49  (3.4%) | 54  (3.8%) | -0.02 | 83  (2.9%) | 0.04 | 48  (3.3%) | 42  (2.9%) | 0.02 |
| Anxiety or phobia | YES | 193  (12.8%) | 19287  (0.9%) | 0.48 | 181  (12.6%) | 769  (13.4%) | -0.02 | 4022  (10.1%) | 0.08 | 181  (12.6%) | 341  (11.9%) | 0.02 | 508  (9.7%) | 0.10 | 177  (12.4%) | 202  (14.1%) | -0.05 | 327  (11.4%) | 0.04 | 180  (12.6%) | 162  (11.3%) | 0.04 |
| Severe stress reaction | YES | 171  (11.3%) | 21233  (1.0%) | 0.44 | 153  (10.6%) | 576  (10.0%) | 0.02 | 2010  (5.0%) | 0.23 | 153  (10.6%) | 296  (10.3%) | 0.01 | 353  (6.7%) | 0.16 | 149  (10.4%) | 158  (11.1%) | -0.02 | 278  (9.7%) | 0.05 | 151  (10.5%) | 137  (9.5%) | 0.03 |
| Stress urinary incontinence | NO | 1512  (100.0%) | 2074652  (100.0%) | 0 | 1438  (100.0%) | 5751  (100.0%) | 0 | 39959  (100.0%) | 0 | 1437  (100.0%) | 2874  (100.0%) | 0 | 5240  (100.0%) | 0 | 1429  (100.0%) | 1429  (100.0%) | 0 | 2876  (100.0%) | 0 | 1434  (100.0%) | 1435  (100.0%) | 0 |
| Renal failure | YES | <5  ( 0%) | 1073  (0.1%) | 0.01 | <5  ( 0%) | 7  (0.1%) | -0.02 | 39  (0.1%) | -0.01 | <5  ( 0%) | <5  ( 0%) | 0.02 | 5  (0.1%) | -0.01 | <5  ( 0%) | <5  ( 0%) | -0.02 | <5  ( 0%) | -0.00 | <5  ( 0%) | <5  ( 0%) | 0.00 |
| Triamcinolone | YES | <5  ( 0%) | 564  (0.0%) | 0.05 | <5  ( 0%) | <5  ( 0%) | 0.04 | 25  (0.1%) | 0.04 | <5  ( 0%) | <5  ( 0%) | 0.05 | <5  ( 0%) | 0.04 | <5  ( 0%) | <5  ( 0%) | 0.02 | <5  ( 0%) | 0.02 | <5  ( 0%) | <5  ( 0%) | 0.04 |
| Dexamethasone | YES | <5  ( 0%) | 36  (0.0%) | -0.01 | <5  ( 0%) | <5  ( 0%) | 0.00 | <5  ( 0%) | -0.01 | <5  ( 0%) | <5  ( 0%) | 0.00 | <5  ( 0%) | 0.00 | <5  ( 0%) | <5  ( 0%) | 0.00 | <5  ( 0%) | 0.00 | <5  ( 0%) | <5  ( 0%) | 0.00 |
| Cortisone | YES | 15  (1.0%) | 10884  (0.5%) | 0.05 | 15  (1.0%) | 80  (1.4%) | -0.03 | 276  (0.7%) | 0.03 | 15  (1.0%) | 19  (0.7%) | 0.04 | 41  (0.8%) | 0.02 | 15  (1.0%) | 17  (1.2%) | -0.01 | 17  (0.6%) | 0.05 | 15  (1.0%) | 10  (0.7%) | 0.04 |
| Prednisolone | YES | 23  (1.5%) | 16863  (0.8%) | 0.07 | 23  (1.6%) | 137  (2.4%) | -0.06 | 594  (1.5%) | 0.00 | 23  (1.6%) | 63  (2.2%) | -0.04 | 64  (1.2%) | 0.03 | 23  (1.6%) | 26  (1.8%) | -0.02 | 51  (1.8%) | -0.02 | 23  (1.6%) | 25  (1.7%) | -0.01 |
| Budesonide | YES | 5  (0.3%) | 476  (0.0%) | 0.07 | 5  (0.3%) | <5  ( 0%) | 0.07 | 27  (0.1%) | 0.06 | 5  (0.3%) | 5  (0.2%) | 0.03 | <5  ( 0%) | 0.06 | 5  (0.3%) | <5  ( 0%) | 0.08 | <5  ( 0%) | 0.06 | 5  (0.3%) | <5  ( 0%) | 0.06 |
| Glucose lowering | YES | 29  (1.9%) | 20726  (1.0%) | 0.08 | 27  (1.9%) | 239  (4.2%) | -0.13 | 645  (1.6%) | 0.02 | 27  (1.9%) | 47  (1.6%) | 0.02 | 99  (1.9%) | 0.00 | 27  (1.9%) | 20  (1.4%) | 0.04 | 64  (2.2%) | -0.02 | 27  (1.9%) | 24  (1.7%) | 0.02 |
| Antihypertensive | YES | 74  (4.9%) | 18035  (0.9%) | 0.24 | 70  (4.9%) | 321  (5.6%) | -0.03 | 1117  (2.8%) | 0.11 | 69  (4.8%) | 125  (4.3%) | 0.02 | 179  (3.4%) | 0.07 | 67  (4.7%) | 69  (4.8%) | -0.01 | 97  (3.4%) | 0.08 | 70  (4.9%) | 67  (4.7%) | 0.01 |
| Fluconazole | YES | 54  (3.6%) | 38057  (1.8%) | 0.11 | 50  (3.5%) | 289  (5.0%) | -0.08 | 1172  (2.9%) | 0.04 | 50  (3.5%) | 91  (3.2%) | 0.02 | 181  (3.5%) | 0.01 | 50  (3.5%) | 51  (3.6%) | -0.00 | 109  (3.8%) | -0.01 | 50  (3.5%) | 35  (2.4%) | 0.06 |
| Mometasone | YES | 93  (6.2%) | 48899  (2.4%) | 0.19 | 89  (6.2%) | 354  (6.2%) | 0.00 | 1766  (4.4%) | 0.08 | 89  (6.2%) | 145  (5.0%) | 0.05 | 217  (4.1%) | 0.09 | 87  (6.1%) | 80  (5.6%) | 0.02 | 155  (5.4%) | 0.03 | 89  (6.2%) | 81  (5.6%) | 0.02 |
| Estradiol | YES | 99  (6.5%) | 116053  (5.6%) | 0.04 | 91  (6.3%) | 375  (6.5%) | -0.01 | 2700  (6.8%) | -0.01 | 91  (6.3%) | 200  (7.0%) | -0.03 | 430  (8.2%) | -0.06 | 90  (6.3%) | 109  (7.6%) | -0.05 | 199  (6.9%) | -0.01 | 91  (6.3%) | 98  (6.8%) | -0.02 |
| Medroxyprogesterone | YES | 9  (0.6%) | 16278  (0.8%) | -0.02 | 9  (0.6%) | 70  (1.2%) | -0.06 | 317  (0.8%) | -0.02 | 9  (0.6%) | 22  (0.8%) | -0.02 | 45  (0.9%) | -0.03 | 9  (0.6%) | 16  (1.1%) | -0.05 | 21  (0.7%) | -0.02 | 9  (0.6%) | 6  (0.4%) | 0.03 |
| Hydroxyprogesterone | NO | 1512  (100.0%) | 2074652  (100.0%) | 0.00 | 1438  (100.0%) | 5751  (100.0%) | 0.00 | 39959  (100.0%) | 0.00 | 1437  (100.0%) | 2874  (100.0%) | 0.00 | 5240  (100.0%) | 0.00 | 1429  (100.0%) | 1429  (100.0%) | 0.00 | 2876  (100.0%) | 0.00 | 1434  (100.0%) | 1435  (100.0%) | 0.00 |
| Progesterone | YES | 33  (2.2%) | 76654  (3.7%) | -0.09 | 29  (2.0%) | 267  (4.6%) | -0.15 | 1569  (3.9%) | -0.10 | 29  (2.0%) | 107  (3.7%) | -0.10 | 159  (3.0%) | -0.05 | 29  (2.0%) | 38  (2.7%) | -0.04 | 89  (3.1%) | -0.06 | 29  (2.0%) | 43  (3.0%) | -0.06 |
| Danazol | YES | <5  ( 0%) | <5  ( 0%) | -0.00 |  |  | 0 | <5  ( 0%) | 0 |  |  | 0 | <5  ( 0%) | 0 |  |  | 0 | <5  ( 0%) | 0 |  |  | 0 |
| Betamethasone | YES | 17  (1.1%) | 10101  (0.5%) | 0.07 | 17  (1.2%) | 92  (1.6%) | -0.04 | 398  (1.0%) | 0.01 | 17  (1.2%) | 40  (1.4%) | -0.02 | 51  (1.0%) | 0.01 | 17  (1.2%) | 17  (1.2%) | 0.00 | 33  (1.1%) | -0.00 | 17  (1.2%) | 18  (1.3%) | -0.01 |
| Prednisone | YES | <5  ( 0%) | 1132  (0.1%) | -0.03 | <5  ( 0%) | <5  ( 0%) | -0.04 | 20  (0.1%) | -0.03 | <5  ( 0%) | <5  ( 0%) | -0.05 | <5  ( 0%) | -0.02 |  |  | 0 | <5  ( 0%) | -0.03 | <5  ( 0%) | <5  ( 0%) | -0.04 |
| Thyroid | YES | 105  (6.9%) | 58379  (2.8%) | 0.19 | 101  (7.0%) | 510  (8.9%) | -0.07 | 2156  (5.4%) | 0.06 | 101  (7.0%) | 182  (6.3%) | 0.03 | 266  (5.1%) | 0.08 | 99  (6.9%) | 111  (7.8%) | -0.03 | 151  (5.3%) | 0.07 | 101  (7.0%) | 103  (7.2%) | -0.01 |
| Antithyroid | YES | <5  ( 0%) | 2439  (0.1%) | -0.02 | <5  ( 0%) | 20  (0.3%) | -0.06 | 56  (0.1%) | -0.02 | <5  ( 0%) | <5  ( 0%) | -0.01 | 7  (0.1%) | -0.02 | <5  ( 0%) | <5  ( 0%) | -0.05 | 5  (0.2%) | -0.03 | <5  ( 0%) | <5  ( 0%) | -0.04 |
| Propylthiouracil | YES | <5  ( 0%) | 1414  (0.1%) | -0.04 | <5  ( 0%) | 13  (0.2%) | -0.07 | 24  (0.1%) | -0.03 | <5  ( 0%) | <5  ( 0%) | -0.05 | <5  ( 0%) | -0.03 | <5  ( 0%) | <5  ( 0%) | -0.04 | <5  ( 0%) | -0.04 | <5  ( 0%) | <5  ( 0%) | -0.04 |
| Methimazole | YES | <5  ( 0%) | 1328  (0.1%) | 0.00 | <5  ( 0%) | 12  (0.2%) | -0.04 | 38  (0.1%) | -0.01 | <5  ( 0%) | <5  ( 0%) | 0.02 | <5  ( 0%) | -0.00 | <5  ( 0%) | <5  ( 0%) | -0.04 | <5  ( 0%) | -0.02 | <5  ( 0%) | <5  ( 0%) | -0.02 |
| NSAID | YES | 228  (15.1%) | 92230  (4.4%) | 0.36 | 211  (14.7%) | 947  (16.5%) | -0.05 | 3410  (8.5%) | 0.20 | 210  (14.6%) | 378  (13.2%) | 0.04 | 567  (10.8%) | 0.13 | 204  (14.3%) | 189  (13.2%) | 0.03 | 381  (13.2%) | 0.05 | 210  (14.6%) | 198  (13.8%) | 0.02 |
| Opioids | YES | 250  (16.5%) | 43589  (2.1%) | 0.51 | 238  (16.6%) | 1017  (17.7%) | -0.03 | 2503  (6.3%) | 0.33 | 237  (16.5%) | 493  (17.2%) | -0.02 | 441  (8.4%) | 0.25 | 229  (16.0%) | 221  (15.5%) | 0.02 | 421  (14.6%) | 0.05 | 237  (16.5%) | 215  (15.0%) | 0.04 |
| Triptans | YES | 81  (5.4%) | 21455  (1.0%) | 0.25 | 79  (5.5%) | 356  (6.2%) | -0.03 | 1136  (2.8%) | 0.13 | 79  (5.5%) | 144  (5.0%) | 0.02 | 193  (3.7%) | 0.08 | 74  (5.2%) | 61  (4.3%) | 0.04 | 109  (3.8%) | 0.08 | 79  (5.5%) | 74  (5.2%) | 0.02 |
| Antiepileptics | YES | 222  (14.7%) | 11510  (0.6%) | 0.55 | 208  (14.5%) | 603  (10.5%) | 0.12 | 1587  (4.0%) | 0.37 | 207  (14.4%) | 407  (14.2%) | 0.01 | 418  (8.0%) | 0.21 | 201  (14.1%) | 185  (12.9%) | 0.03 | 273  (9.5%) | 0.16 | 204  (14.2%) | 192  (13.4%) | 0.02 |
| Antipsychotics | YES | 171  (11.3%) | 7043  (0.3%) | 0.48 | 164  (11.4%) | 523  (9.1%) | 0.08 | 1680  (4.2%) | 0.27 | 163  (11.3%) | 301  (10.5%) | 0.03 | 438  (8.4%) | 0.10 | 162  (11.3%) | 151  (10.6%) | 0.02 | 229  (8.0%) | 0.11 | 161  (11.2%) | 161  (11.2%) | 0.00 |
| Anxiolytics | YES | 319  (21.1%) | 21582  (1.0%) | 0.67 | 301  (20.9%) | 1060  (18.4%) | 0.06 | 5418  (13.6%) | 0.20 | 300  (20.9%) | 565  (19.7%) | 0.03 | 855  (16.3%) | 0.12 | 296  (20.7%) | 281  (19.7%) | 0.03 | 433  (15.1%) | 0.16 | 298  (20.8%) | 290  (20.2%) | 0.01 |
| Corticosteroid  (combination) | YES | 145  (9.6%) | 88888  (4.3%) | 0.21 | 141  (9.8%) | 644  (11.2%) | -0.05 | 3006  (7.5%) | 0.07 | 141  (9.8%) | 258  (9.0%) | 0.03 | 369  (7.0%) | 0.09 | 139  (9.7%) | 137  (9.6%) | 0.00 | 253  (8.8%) | 0.03 | 141  (9.8%) | 132  (9.2%) | 0.02 |
| Fluticasone | YES | 6  (0.4%) | 5005  (0.2%) | 0.03 | 6  (0.4%) | 24  (0.4%) | -0.00 | 145  (0.4%) | 0.01 | 6  (0.4%) | 15  (0.5%) | -0.02 | 20  (0.4%) | 0.00 | 6  (0.4%) | 5  (0.3%) | 0.01 | 9  (0.3%) | 0.01 | 6  (0.4%) | <5  ( 0%) | 0.02 |
| Progesterone  (combination) | YES | 41  (2.7%) | 88603  (4.3%) | -0.09 | 37  (2.6%) | 315  (5.5%) | -0.15 | 1803  (4.5%) | -0.10 | 37  (2.6%) | 121  (4.2%) | -0.09 | 193  (3.7%) | -0.06 | 37  (2.6%) | 49  (3.4%) | -0.05 | 103  (3.6%) | -0.05 | 37  (2.6%) | 46  (3.2%) | -0.04 |
| Antithyroid  (combination) | YES | <5  ( 0%) | 2439  (0.1%) | -0.02 | <5  ( 0%) | 20  (0.3%) | -0.06 | 56  (0.1%) | -0.02 | <5  ( 0%) | <5  ( 0%) | -0.01 | 7  (0.1%) | -0.02 | <5  ( 0%) | <5  ( 0%) | -0.05 | 5  (0.2%) | -0.03 | <5  ( 0%) | <5  ( 0%) | -0.04 |
| SSRI | YES | <5  ( 0%) | 39959  (1.9%) | -0.20 | <5  ( 0%) | 923  (16.0%) | -0.62 | 39959  (100.0%) | . | <5  ( 0%) | 2874  (100.0%) | . | 710  (13.5%) | -0.56 | <5  ( 0%) | 185  (12.9%) | -0.55 | 579  (20.1%) | -0.71 | <5  ( 0%) | 302  (21.0%) | -0.73 |
| venlafaxine | YES | <5  ( 0%) | 5240  (0.3%) | -0.07 | <5  ( 0%) | 173  (3.0%) | -0.25 | 710  (1.8%) | -0.19 | <5  ( 0%) | 71  (2.5%) | -0.23 | 5240  (100.0%) | . | <5  ( 0%) | 1429  (100.0%) | . | 88  (3.1%) | -0.25 | <5  ( 0%) | 49  (3.4%) | -0.27 |

Propensity score [PS]-matched models were based on covariates covering comorbidity (up to five years prior LPM), comedication (during the relevant time period), hospital contacts, education, and income. For the complete list for the individual analyses, see supplementary material Table S5.

# Table G baseline table for all covariates, stillbirth analyses. Before and after propensity score matching.

| Variable | Value | Duloxetine  Before matching  n=1,668 | Duloxetine vs. duloxetine non-exposed | | | | | Duloxetine vs. SSRI | | | | | Duloxetine vs. venlafaxine | | | | | Duloxetine vs. duloxetine discontinuers | | | | |
| --- | --- | --- | --- | --- | --- | --- | --- | --- | --- | --- | --- | --- | --- | --- | --- | --- | --- | --- | --- | --- | --- | --- |
|  |  |  | Before matching | | After matching | | | Before matching | | After matching | | | Before matching | | After matching | | | Before matching | | After matching | | |
|  |  |  | Duloxetine  non-exposed  n=2,130,495 | Std mean diff. | Duloxetine  n=1,581 | Duloxetine  non-exposed  n=6,324 | Std mean diff. | SSRI  n=54,792 | Std mean diff. | Duloxetine  n=1,585 | SSRI  n=3,170 | Std mean diff. | Venlafaxine  n=6,005 | Std mean diff | Duloxetine  n=1,580 | Venlafaxine  n=1,580 | Std mean diff | Duloxetine discontinuers  n=2,815 | Std mean diff | Duloxetine  n=1,559 | Discontinuers  n=1,561 | Std mean diff |
| Age, continuous | Mean (interquartile range)s | 31.0  (27.3; 35.1) | 30.2  (26.7; 33.6) | 0.18 | 30.9  (27.3; 35.1) | 30.9  (26.6; 35.0) | 0.05 | 30.7  (26.8; 34.4) | 0.10 | 30.9  (27.3; 35.1) | 30.7  (26.9; 34.9) | 0.05 | 30.6  (26.6; 34.6) | 0.10 | 30.9  (27.3; 35.1) | 30.6  (26.9; 35.1) | 0.02 | 30.3  (26.6; 34.3) | 0.13 | 30.9  (27.3; 35.1) | 30.8  (27.0; 34.7) | 0.04 |
| Age, grouped | 18-24 years | 245  (14.7%) | 335954  (15.8%) | 0.16 | 235  (14.9%) | 1087  (17.2%) | 0.07 | 8706  (15.9%) | 0.05 | 236  (14.9%) | 464  (14.6%) | 0.05 | 1038  (17.3%) | 0.06 | 235  (14.9%) | 227  (14.4%) | 0.03 | 470  (16.7%) | 0.11 | 236  (15.1%) | 231  (14.8%) | 0.00 |
|  | 25-29 years | 485  (29.1%) | 702496  (33.0%) |  | 466  (29.5%) | 1723  (27.2%) |  | 16148  (29.5%) |  | 467  (29.5%) | 973  (30.7%) |  | 1720  (28.6%) |  | 466  (29.5%) | 477  (30.2%) |  | 893  (31.7%) |  | 460  (29.5%) | 466  (29.9%) |  |
|  | 30-34 years | 426  (25.5%) | 604784  (28.4%) |  | 398  (25.2%) | 1529  (24.2%) |  | 14876  (27.1%) |  | 400  (25.2%) | 769  (24.3%) |  | 1566  (26.1%) |  | 398  (25.2%) | 390  (24.7%) |  | 692  (24.6%) |  | 391  (25.1%) | 395  (25.3%) |  |
|  | 35-60 years | 512  (30.7%) | 487539  (22.9%) |  | 482  (30.5%) | 1985  (31.4%) |  | 15067  (27.5%) |  | 482  (30.4%) | 964  (30.4%) |  | 1684  (28.0%) |  | 481  (30.4%) | 486  (30.8%) |  | 762  (27.1%) |  | 472  (30.3%) | 467  (30.0%) |  |
| BMI, continuous | Mean (interquartile range) | 25.4  (22.3; 30.1) | 23.5  (21.3; 26.7) | 0.39 | 25.4  (22.3; 30.0) | 24.8  (21.9; 29.4) | 0.08 | 24.3  (21.8; 28.3) | 0.19 | 25.4  (22.3; 30.0) | 24.7  (21.9; 29.1) | 0.12 | 25.1  (22.1; 29.4) | 0.06 | 25.4  (22.3; 30.0) | 25.1  (22.2; 29.3) | 0.06 | 25.0  (22.2; 29.7) | 0.04 | 25.4  (22.3; 29.9) | 25.3  (22.4; 29.7) | -0.01 |
| BMI, grouped | BMI <21 | 229  (14.7%) | 435948  (21.9%) | 0.39 | 225  (14.9%) | 1080  (17.7%) | 0.09 | 9361  (18.2%) | 0.17 | 225  (14.8%) | 541  (17.7%) | 0.11 | 889  (15.8%) | 0.06 | 225  (14.9%) | 219  (14.4%) | 0.08 | 362  (13.8%) | 0.08 | 223  (14.9%) | 204  (13.6%) | 0.05 |
|  | BMI 21-26< | 613  (39.4%) | 977469  (49.0%) |  | 599  (39.5%) | 2466  (40.4%) |  | 22836  (44.4%) |  | 601  (39.6%) | 1280  (41.9%) |  | 2332  (41.4%) |  | 597  (39.4%) | 641  (42.2%) |  | 1131  (43.0%) |  | 593  (39.7%) | 627  (41.8%) |  |
|  | BMI 26-30< | 323  (20.7%) | 331642  (16.6%) |  | 313  (20.7%) | 1196  (19.6%) |  | 9793  (19.0%) |  | 313  (20.6%) | 563  (18.4%) |  | 1134  (20.1%) |  | 313  (20.7%) | 328  (21.6%) |  | 503  (19.1%) |  | 308  (20.6%) | 304  (20.3%) |  |
|  | BMI >=30 | 392  (25.2%) | 249532  (12.5%) |  | 378  (25.0%) | 1356  (22.2%) |  | 9418  (18.3%) |  | 380  (25.0%) | 670  (21.9%) |  | 1273  (22.6%) |  | 379  (25.0%) | 331  (21.8%) |  | 633  (24.1%) |  | 371  (24.8%) | 364  (24.3%) |  |
| householdincome | income quartile1 | 627  (37.7%) | 481338  (22.8%) | 0.39 | 601  (38.0%) | 2371  (37.5%) | 0.04 | 16538  (30.4%) | 0.19 | 605  (38.2%) | 1232  (38.9%) | 0.03 | 2172  (36.4%) | 0.05 | 603  (38.2%) | 606  (38.4%) | 0.06 | 1031  (36.8%) | 0.08 | 595  (38.2%) | 603  (38.7%) | 0.03 |
|  | income quartile2 | 427  (25.7%) | 529514  (25.1%) |  | 402  (25.4%) | 1544  (24.4%) |  | 14121  (26.0%) |  | 402  (25.4%) | 793  (25.0%) |  | 1617  (27.1%) |  | 401  (25.4%) | 423  (26.8%) |  | 806  (28.8%) |  | 396  (25.4%) | 393  (25.2%) |  |
|  | income quartile3 | 353  (21.2%) | 554446  (26.2%) |  | 334  (21.1%) | 1406  (22.2%) |  | 13320  (24.5%) |  | 334  (21.1%) | 677  (21.4%) |  | 1261  (21.1%) |  | 333  (21.1%) | 331  (20.9%) |  | 578  (20.6%) |  | 326  (20.9%) | 327  (21.0%) |  |
|  | income quartile4 | 255  (15.3%) | 547490  (25.9%) |  | 244  (15.4%) | 1003  (15.9%) |  | 10383  (19.1%) |  | 244  (15.4%) | 468  (14.8%) |  | 913  (15.3%) |  | 243  (15.4%) | 220  (13.9%) |  | 388  (13.8%) |  | 242  (15.5%) | 236  (15.1%) |  |
| Education | < 11 years | 374  (22.6%) | 262602  (12.5%) | 0.35 | 359  (22.7%) | 1617  (25.6%) | 0.07 | 10068  (18.5%) | 0.17 | 360  (22.7%) | 707  (22.3%) | 0.03 | 1386  (23.3%) | 0.07 | 357  (22.6%) | 333  (21.1%) | 0.05 | 708  (25.3%) | 0.05 | 355  (22.8%) | 348  (22.3%) | 0.03 |
|  | 11-15 years | 878  (53.1%) | 1020571  (48.6%) |  | 837  (52.9%) | 3191  (50.5%) |  | 26933  (49.6%) |  | 840  (53.0%) | 1703  (53.7%) |  | 2995  (50.5%) |  | 838  (53.0%) | 868  (54.9%) |  | 1425  (50.9%) |  | 824  (52.9%) | 842  (54.0%) |  |
|  | >16 years | 402  (24.3%) | 816010  (38.9%) |  | 385  (24.4%) | 1516  (24.0%) |  | 17306  (31.9%) |  | 385  (24.3%) | 760  (24.0%) |  | 1555  (26.2%) |  | 385  (24.4%) | 379  (24.0%) |  | 667  (23.8%) |  | 380  (24.4%) | 369  (23.7%) |  |
| smoking | YES | 336  (20.9%) | 183149  (8.9%) | 0.34 | 332  (21.0%) | 1447  (22.9%) | -0.05 | 9011  (17.0%) | 0.10 | 332  (20.9%) | 661  (20.9%) | 0.00 | 1469  (25.3%) | -0.10 | 331  (20.9%) | 321  (20.3%) | 0.02 | 560  (20.7%) | 0.01 | 324  (20.8%) | 316  (20.3%) | 0.01 |
| Data source | Sweden | 1120  (67.1%) | 1360775  (63.9%) | 0.07 | 1059  (67.0%) | 4293  (67.9%) | -0.02 | 36616  (66.8%) | 0.01 | 1063  (67.1%) | 2086  (65.8%) | 0.03 | 3414  (56.8%) | 0.21 | 1058  (67.0%) | 1084  (68.6%) | -0.04 | 1770  (62.8%) | 0.09 | 1040  (66.7%) | 1007  (64.6%) | 0.04 |
| Previous stillbirth | YES | 6  (0.4%) | 10898  (0.5%) | -0.02 | 6  (0.4%) | 90  (1.4%) | -0.11 | 339  (0.6%) | -0.04 | 6  (0.4%) | 10  (0.3%) | 0.01 | 29  (0.5%) | -0.02 | 6  (0.4%) | 8  (0.5%) | -0.02 | 19  (0.7%) | -0.04 | 6  (0.4%) | 5  (0.3%) | 0.01 |
| Parity | 0 | 779  (46.9%) | 975066  (45.9%) | 0.25 | 724  (46.0%) | 2710  (43.0%) | 0.13 | 25110  (46.0%) | 0.15 | 726  (46.0%) | 1373  (43.4%) | 0.14 | 2907  (48.5%) | 0.10 | 725  (46.1%) | 728  (46.2%) | 0.06 | 1402  (49.9%) | 0.06 | 715  (46.0%) | 758  (48.7%) | 0.07 |
|  | 1 | 450  (27.1%) | 762954  (35.9%) |  | 436  (27.7%) | 2088  (33.1%) |  | 18090  (33.1%) |  | 437  (27.7%) | 1085  (34.3%) |  | 1761  (29.4%) |  | 436  (27.7%) | 471  (29.9%) |  | 708  (25.2%) |  | 431  (27.8%) | 414  (26.6%) |  |
|  | 2 | 275  (16.5%) | 275134  (13.0%) |  | 263  (16.7%) | 914  (14.5%) |  | 7658  (14.0%) |  | 264  (16.7%) | 443  (14.0%) |  | 871  (14.5%) |  | 262  (16.6%) | 251  (15.9%) |  | 453  (16.1%) |  | 258  (16.6%) | 241  (15.5%) |  |
|  | >2 | 158  (9.5%) | 109606  (5.2%) |  | 152  (9.7%) | 594  (9.4%) |  | 3746  (6.9%) |  | 152  (9.6%) | 259  (8.2%) |  | 450  (7.5%) |  | 151  (9.6%) | 126  (8.0%) |  | 245  (8.7%) |  | 149  (9.6%) | 144  (9.2%) |  |
| previous spontaneous abortions | 0 | 1300  (77.9%) | 1701280  (79.8%) | 0.09 | 1229  (77.7%) | 4629  (73.2%) | 0.12 | 42484  (77.5%) | 0.05 | 1232  (77.7%) | 2468  (77.9%) | 0.00 | 4822  (80.3%) | 0.05 | 1229  (77.8%) | 1241  (78.5%) | 0.04 | 2162  (76.7%) | 0.07 | 1212  (77.7%) | 1229  (78.8%) | 0.03 |
|  | 1 | 268  (16.1%) | 342719  (16.1%) |  | 258  (16.3%) | 1227  (19.4%) |  | 9489  (17.3%) |  | 258  (16.3%) | 506  (16.0%) |  | 923  (15.4%) |  | 257  (16.3%) | 247  (15.6%) |  | 499  (17.7%) |  | 254  (16.3%) | 241  (15.5%) |  |
|  | 2+ | 100  (6.0%) | 86703  (4.1%) |  | 94  (5.9%) | 468  (7.4%) |  | 2819  (5.1%) |  | 95  (6.0%) | 196  (6.2%) |  | 260  (4.3%) |  | 94  (5.9%) | 92  (5.8%) |  | 156  (5.5%) |  | 93  (6.0%) | 89  (5.7%) |  |
| Outpatient visits, grouped | 0 | 1525  (91.4%) | 2042308  (95.8%) | 0.21 | 1448  (91.6%) | 5733  (90.7%) | 0.06 | 51234  (93.5%) | 0.09 | 1452  (91.6%) | 2904  (91.6%) | 0.08 | 5594  (93.1%) | 0.08 | 1447  (91.6%) | 1473  (93.2%) | 0.04 | 2533  (89.9%) | 0.04 | 1431  (91.8%) | 1392  (89.3%) | 0.13 |
|  | 1 | 102  (6.1%) | 61509  (2.9%) |  | 95  (6.0%) | 371  (5.9%) |  | 2407  (4.4%) |  | 95  (6.0%) | 172  (5.4%) |  | 305  (5.1%) |  | 95  (6.0%) | 73  (4.6%) |  | 191  (6.8%) |  | 90  (5.8%) | 114  (7.3%) |  |
|  | >1 | 41  (2.5%) | 26956  (1.3%) |  | 38  (2.4%) | 220  (3.5%) |  | 1156  (2.1%) |  | 38  (2.4%) | 94  (3.0%) |  | 109  (1.8%) |  | 38  (2.4%) | 34  (2.2%) |  | 93  (3.3%) |  | 38  (2.4%) | 53  (3.4%) |  |
| Hospital, grouped | 0 | 1417  (85.0%) | 1977458  (92.8%) | 0.29 | 1351  (85.5%) | 5426  (85.8%) | 0.05 | 49169  (89.7%) | 0.15 | 1354  (85.4%) | 2672  (84.3%) | 0.04 | 5153  (85.8%) | 0.05 | 1350  (85.4%) | 1365  (86.4%) | 0.03 | 2442  (86.7%) | 0.07 | 1333  (85.5%) | 1349  (86.5%) | 0.08 |
|  | 1 | 168  (10.1%) | 118433  (5.6%) |  | 156  (9.9%) | 612  (9.7%) |  | 3955  (7.2%) |  | 156  (9.8%) | 323  (10.2%) |  | 584  (9.7%) |  | 156  (9.9%) | 146  (9.2%) |  | 239  (8.5%) |  | 154  (9.9%) | 130  (8.3%) |  |
|  | >1 | 83  (5.0%) | 34882  (1.6%) |  | 74  (4.7%) | 286  (4.5%) |  | 1673  (3.1%) |  | 75  (4.7%) | 175  (5.5%) |  | 271  (4.5%) |  | 74  (4.7%) | 69  (4.4%) |  | 136  (4.8%) |  | 72  (4.6%) | 80  (5.1%) |  |
| Emergency depart., grouped | 0 | 1581  (94.8%) | 2061789  (96.8%) | 0.20 | 1496  (94.6%) | 6005  (95.0%) | 0.00 | 51970  (94.8%) | 0.10 | 1500  (94.6%) | 2991  (94.4%) | 0.06 | 5575  (92.8%) | 0.10 | 1495  (94.6%) | 1492  (94.4%) | 0.05 | 2635  (93.5%) | 0.05 | 1475  (94.6%) | 1467  (94.1%) | 0.05 |
|  | 1 | 55  (3.3%) | 55142  (2.6%) |  | 54  (3.4%) | 197  (3.1%) |  | 1961  (3.6%) |  | 54  (3.4%) | 110  (3.5%) |  | 297  (4.9%) |  | 54  (3.4%) | 64  (4.1%) |  | 114  (4.0%) |  | 54  (3.5%) | 59  (3.8%) |  |
|  | >1 | 32  (1.9%) | 13842  (0.6%) |  | 31  (2.0%) | 122  (1.9%) |  | 866  (1.6%) |  | 31  (2.0%) | 69  (2.2%) |  | 136  (2.3%) |  | 31  (2.0%) | 24  (1.5%) |  | 68  (2.4%) |  | 30  (1.9%) | 33  (2.1%) |  |
| Psychiatric outpatient, grouped | 0 | 1145  (68.6%) | 2102059  (98.7%) | 0.89 | 1094  (69.2%) | 4374  (69.2%) | -0.00 | 46068  (84.1%) | 0.37 | 1094  (69.0%) | 2174  (68.6%) | -0.01 | 4585  (76.3%) | 0.17 | 1094  (69.2%) | 1079  (68.3%) | -0.02 | 2022  (71.8%) | 0.07 | 1084  (69.5%) | 1071  (68.7%) | -0.02 |
|  | 1+ | 523  (31.4%) | 28714  (1.3%) |  | 487  (30.8%) | 1950  (30.8%) |  | 8729  (15.9%) |  | 491  (31.0%) | 996  (31.4%) |  | 1423  (23.7%) |  | 486  (30.8%) | 501  (31.7%) |  | 795  (28.2%) |  | 475  (30.5%) | 488  (31.3%) |  |
| Psychiatric hospital, grouped | 0 | 1562  (93.6%) | 2126433  (99.8%) | 0.35 | 1486  (94.0%) | 6026  (95.3%) | 0.06 | 53567  (97.8%) | 0.20 | 1489  (93.9%) | 2979  (94.0%) | 0.00 | 5714  (95.1%) | 0.06 | 1485  (94.0%) | 1488  (94.2%) | 0.01 | 2673  (94.9%) | 0.05 | 1467  (94.1%) | 1465  (94.0%) | -0.01 |
|  | 1+ | 106  (6.4%) | 4340  (0.2%) |  | 95  (6.0%) | 298  (4.7%) |  | 1230  (2.2%) |  | 96  (6.1%) | 191  (6.0%) |  | 294  (4.9%) |  | 95  (6.0%) | 92  (5.8%) |  | 144  (5.1%) |  | 92  (5.9%) | 94  (6.0%) |  |
| gestational diabetes during index pregnancy | YES | 99  (5.9%) | 52218  (2.5%) | 0.17 | 88  (5.6%) | 594  (9.4%) | -0.15 | 1998  (3.6%) | 0.11 | 89  (5.6%) | 164  (5.2%) | 0.02 | 303  (5.0%) | 0.04 | 89  (5.6%) | 81  (5.1%) | 0.02 | 149  (5.3%) | 0.03 | 89  (5.7%) | 79  (5.1%) | 0.03 |
| diabetes | YES | 47  (2.8%) | 25924  (1.2%) | 0.11 | 43  (2.7%) | 326  (5.2%) | -0.13 | 1059  (1.9%) | 0.06 | 43  (2.7%) | 96  (3.0%) | -0.02 | 138  (2.3%) | 0.03 | 43  (2.7%) | 43  (2.7%) | 0.00 | 67  (2.4%) | 0.03 | 42  (2.7%) | 35  (2.2%) | 0.03 |
| hyper or hypothyroidism | YES | 108  (6.5%) | 57237  (2.7%) | 0.18 | 104  (6.6%) | 532  (8.4%) | -0.07 | 2746  (5.0%) | 0.06 | 104  (6.6%) | 179  (5.6%) | 0.04 | 303  (5.0%) | 0.06 | 102  (6.5%) | 112  (7.1%) | -0.03 | 133  (4.7%) | 0.08 | 97  (6.2%) | 98  (6.3%) | -0.00 |
| Hypertension | YES | 17  (1.0%) | 6875  (0.3%) | 0.09 | 16  (1.0%) | 140  (2.2%) | -0.10 | 334  (0.6%) | 0.05 | 16  (1.0%) | 32  (1.0%) | 0.00 | 45  (0.7%) | 0.03 | 16  (1.0%) | 15  (0.9%) | 0.01 | 19  (0.7%) | 0.04 | 14  (0.9%) | 14  (0.9%) | 0.00 |
| Obesity | YES | 119  (7.1%) | 64541  (3.0%) | 0.19 | 116  (7.3%) | 582  (9.2%) | -0.07 | 2849  (5.2%) | 0.08 | 116  (7.3%) | 221  (7.0%) | 0.01 | 403  (6.7%) | 0.02 | 115  (7.3%) | 102  (6.5%) | 0.03 | 195  (6.9%) | 0.01 | 113  (7.2%) | 108  (6.9%) | 0.01 |
| depression | YES | 578  (34.7%) | 39620  (1.9%) | 0.94 | 541  (34.2%) | 2342  (37.0%) | -0.06 | 9984  (18.2%) | 0.38 | 545  (34.4%) | 1129  (35.6%) | -0.03 | 1529  (25.4%) | 0.20 | 540  (34.2%) | 572  (36.2%) | -0.04 | 775  (27.5%) | 0.15 | 524  (33.6%) | 525  (33.7%) | -0.00 |
| affective disorder | YES | 58  (3.5%) | 3221  (0.2%) | 0.25 | 55  (3.5%) | 195  (3.1%) | 0.02 | 757  (1.4%) | 0.14 | 55  (3.5%) | 115  (3.6%) | -0.01 | 137  (2.3%) | 0.07 | 54  (3.4%) | 58  (3.7%) | -0.01 | 80  (2.8%) | 0.04 | 52  (3.3%) | 50  (3.2%) | 0.01 |
| anxiety or phobia | YES | 217  (13.0%) | 19510  (0.9%) | 0.49 | 202  (12.8%) | 811  (12.8%) | -0.00 | 5348  (9.8%) | 0.10 | 205  (12.9%) | 442  (13.9%) | -0.03 | 575  (9.6%) | 0.11 | 202  (12.8%) | 210  (13.3%) | -0.02 | 312  (11.1%) | 0.06 | 194  (12.4%) | 185  (11.9%) | 0.02 |
| Severe stress reaction | YES | 203  (12.2%) | 21580  (1.0%) | 0.46 | 181  (11.4%) | 679  (10.7%) | 0.02 | 2815  (5.1%) | 0.25 | 184  (11.6%) | 363  (11.5%) | 0.00 | 399  (6.6%) | 0.19 | 180  (11.4%) | 176  (11.1%) | 0.01 | 260  (9.2%) | 0.10 | 173  (11.1%) | 175  (11.2%) | -0.00 |
| Stress urinary incontinence | NO | 1668 (100.0%) | 2130773  (100.0%) | 0.00 | 1581  (100.0%) | 6324  (100.0%) | 0.00 | 54797  (100.0%) | 0.00 | 1585  (100.0%) | 3170  (100.0%) | 0.00 | 6008  (100.0%) | 0.00 | 1580  (100.0%) | 1580  (100.0%) | 0.00 | 2817  (100.0%) | 0.00 | 1559  (100.0%) | 1559  (100.0%) | 0.00 |
| Renal failure | YES | <5  (0%) | 1103  (0.1%) | 0.00 | <5  (0%) | 13  (0.2%) | -0.04 | 49  (0.1%) | -0.01 | <5  (0%) | <5  (0%) | 0.00 | 6  (0.1%) | -0.01 | <5  (0%) | <5  (0%) | 0.00 | <5  (0%) | -0.00 | <5  (0%) | <5  (0%) | 0.00 |
| Triamcinolone | YES | <5  (0%) | 853  (0.0%) | 0.04 | <5  (0%) | 5  (0.1%) | 0.03 | 36  (0.1%) | 0.03 | <5  (0%) | <5  (0%) | 0.05 | 7  (0.1%) | 0.02 | <5  (0%) | <5  (0%) | 0.06 | <5  (0%) | 0.02 | <5  (0%) | <5  (0%) | 0.06 |
| Dexamethasone | YES | <5  (0%) | 53  (0.0%) | -0.01 | <5  (0%) | <5  (0%) | 0.00 | <5  (0%) | -0.01 | <5  (0%) | <5  (0%) | 0.00 | <5  (0%) | 0.00 | <5  (0%) | <5  (0%) | 0.00 | <5  (0%) | 0.00 | <5  (0%) | <5  (0%) | 0.00 |
| Cortisone | YES | 46  (2.8%) | 44861  (2.1%) | 0.04 | 45  (2.8%) | 220  (3.5%) | -0.04 | 1288  (2.4%) | 0.03 | 45  (2.8%) | 89  (2.8%) | 0.00 | 182  (3.0%) | -0.02 | 45  (2.8%) | 39  (2.5%) | 0.02 | 78  (2.8%) | -0.00 | 45  (2.9%) | 38  (2.4%) | 0.03 |
| Prednisolone | YES | 51  (3.1%) | 27937  (1.3%) | 0.12 | 48  (3.0%) | 243  (3.8%) | -0.04 | 1289  (2.4%) | 0.04 | 48  (3.0%) | 99  (3.1%) | -0.01 | 129  (2.1%) | 0.06 | 48  (3.0%) | 44  (2.8%) | 0.02 | 81  (2.9%) | 0.01 | 45  (2.9%) | 50  (3.2%) | -0.02 |
| Budesonide | YES | 7  (0.4%) | 676  (0.0%) | 0.08 | 7  (0.4%) | <5  (0%) | 0.08 | 48  (0.1%) | 0.07 | 7  (0.4%) | <5  (0%) | 0.06 | 5  (0.1%) | 0.07 | 7  (0.4%) | <5  (0%) | 0.08 | <5  (0%) | 0.07 | 7  (0.4%) | <5  (0%) | 0.08 |
| Glucoselowering | YES | 58  (3.5%) | 28528  (1.3%) | 0.14 | 51  (3.2%) | 337  (5.3%) | -0.10 | 1221  (2.2%) | 0.08 | 52  (3.3%) | 106  (3.3%) | -0.00 | 172  (2.9%) | 0.04 | 52  (3.3%) | 53  (3.4%) | -0.00 | 86  (3.1%) | 0.02 | 51  (3.3%) | 41  (2.6%) | 0.04 |
| antihypertensive | YES | 127  (7.6%) | 35742  (1.7%) | 0.28 | 121  (7.7%) | 567  (9.0%) | -0.05 | 2237  (4.1%) | 0.15 | 123  (7.8%) | 231  (7.3%) | 0.02 | 349  (5.8%) | 0.07 | 121  (7.7%) | 120  (7.6%) | 0.00 | 133  (4.7%) | 0.12 | 110  (7.1%) | 97  (6.2%) | 0.03 |
| fluconazole | YES | 83  (5.0%) | 48458  (2.3%) | 0.14 | 77  (4.9%) | 432  (6.8%) | -0.08 | 1977  (3.6%) | 0.07 | 78  (4.9%) | 136  (4.3%) | 0.03 | 246  (4.1%) | 0.04 | 77  (4.9%) | 75  (4.7%) | 0.01 | 152  (5.4%) | -0.02 | 77  (4.9%) | 76  (4.9%) | 0.00 |
| Mometasone | YES | 157  (9.4%) | 84854  (4.0%) | 0.22 | 150  (9.5%) | 551  (8.7%) | 0.03 | 3863  (7.0%) | 0.09 | 151  (9.5%) | 251  (7.9%) | 0.06 | 395  (6.6%) | 0.10 | 151  (9.6%) | 127  (8.0%) | 0.05 | 246  (8.7%) | 0.02 | 144  (9.2%) | 142  (9.1%) | 0.00 |
| estradiol | YES | 105  (6.3%) | 118878  (5.6%) | 0.03 | 97  (6.1%) | 385  (6.1%) | 0.00 | 3602  (6.6%) | -0.01 | 97  (6.1%) | 216  (6.8%) | -0.03 | 483  (8.0%) | -0.07 | 97  (6.1%) | 109  (6.9%) | -0.03 | 204  (7.2%) | -0.04 | 96  (6.2%) | 106  (6.8%) | -0.03 |
| Medroxyprogesterone | YES | 9  (0.5%) | 16886  (0.8%) | -0.03 | 9  (0.6%) | 80  (1.3%) | -0.07 | 442  (0.8%) | -0.03 | 9  (0.6%) | 29  (0.9%) | -0.04 | 51  (0.8%) | -0.04 | 9  (0.6%) | 13  (0.8%) | -0.03 | 23  (0.8%) | -0.03 | 9  (0.6%) | 15  (1.0%) | -0.04 |
| Hydroxyprogesterone | YES | <5  (0%) | <5  (0%) | -0.00 |  |  | 0 | <5  (0%) | 0 |  |  | 0 | <5  (0%) | 0 |  |  | 0 | <5  (0%) | 0 |  |  | 0 |
| Progesterone | YES | 40  (2.4%) | 80648  (3.8%) | -0.08 | 36  (2.3%) | 312  (4.9%) | -0.14 | 2234  (4.1%) | -0.09 | 36  (2.3%) | 140  (4.4%) | -0.12 | 200  (3.3%) | -0.06 | 36  (2.3%) | 54  (3.4%) | -0.07 | 93  (3.3%) | -0.05 | 34  (2.2%) | 48  (3.1%) | -0.06 |
| Danazol | YES | <5  (0%) | <5  (0%) | -0.00 |  |  | 0 | <5  (0%) | 0 |  |  | 0 | <5  (0%) | 0 |  |  | 0 | <5  (0%) | 0 |  |  | 0 |
| Betamethasone | YES | 28  (1.7%) | 13665  (0.6%) | 0.10 | 28  (1.8%) | 135  (2.1%) | -0.03 | 718  (1.3%) | 0.03 | 28  (1.8%) | 51  (1.6%) | 0.01 | 87  (1.4%) | 0.02 | 28  (1.8%) | 32  (2.0%) | -0.02 | 43  (1.5%) | 0.01 | 27  (1.7%) | 29  (1.9%) | -0.01 |
| Prednisone | YES | <5  (0%) | 1309  (0.1%) | -0.04 | <5  (0%) | <5  (0%) | -0.04 | 38  (0.1%) | -0.04 | <5  (0%) | <5  (0%) | -0.05 | <5  (0%) | -0.03 |  |  | 0 | <5  (0%) | -0.03 | <5  (0%) | <5  (0%) | -0.04 |
| thyroid | YES | 137  (8.2%) | 74842  (3.5%) | 0.20 | 132  (8.3%) | 695  (11.0%) | -0.09 | 3577  (6.5%) | 0.06 | 132  (8.3%) | 238  (7.5%) | 0.03 | 381  (6.3%) | 0.07 | 130  (8.2%) | 150  (9.5%) | -0.04 | 180  (6.4%) | 0.07 | 125  (8.0%) | 131  (8.4%) | -0.01 |
| Antithyroid | YES | 5  (0.3%) | 3051  (0.1%) | 0.03 | 5  (0.3%) | 49  (0.8%) | -0.06 | 80  (0.1%) | 0.03 | 5  (0.3%) | 5  (0.2%) | 0.03 | 9  (0.1%) | 0.03 | 5  (0.3%) | <5  (0%) | 0.04 | 5  (0.2%) | 0.03 | <5  (0%) | <5  (0%) | 0.00 |
| Propylthiouracil | YES | <5  (0%) | 1926  (0.1%) | 0.01 | <5  (0%) | 35  (0.6%) | -0.07 | 43  (0.1%) | 0.01 | <5  (0%) | <5  (0%) | 0.01 | <5  (0%) | 0.02 | <5  (0%) | <5  (0%) | 0.05 | <5  (0%) | 0.00 | <5  (0%) | <5  (0%) | -0.02 |
| Methimazole | YES | <5  (0%) | 1568  (0.1%) | 0.03 | <5  (0%) | 21  (0.3%) | -0.03 | 48  (0.1%) | 0.03 | <5  (0%) | <5  (0%) | 0.04 | 5  (0.1%) | 0.03 | <5  (0%) | <5  (0%) | 0.02 | <5  (0%) | 0.01 | <5  (0%) | <5  (0%) | -0.02 |
| NSAID | YES | 271  (16.2%) | 116672  (5.5%) | 0.35 | 252  (15.9%) | 1138  (18.0%) | -0.05 | 5448  (9.9%) | 0.19 | 253  (16.0%) | 483  (15.2%) | 0.02 | 749  (12.5%) | 0.11 | 249  (15.8%) | 221  (14.0%) | 0.05 | 416  (14.8%) | 0.04 | 239  (15.3%) | 241  (15.5%) | -0.00 |
| opioids | YES | 368  (22.1%) | 86696  (4.1%) | 0.55 | 348  (22.0%) | 1491  (23.6%) | -0.04 | 5945  (10.8%) | 0.31 | 351  (22.1%) | 734  (23.2%) | -0.02 | 767  (12.8%) | 0.25 | 346  (21.9%) | 343  (21.7%) | 0.00 | 560  (19.9%) | 0.05 | 339  (21.7%) | 314  (20.1%) | 0.04 |
| triptanes | YES | 103  (6.2%) | 25462  (1.2%) | 0.27 | 97  (6.1%) | 442  (7.0%) | -0.03 | 1723  (3.1%) | 0.14 | 98  (6.2%) | 184  (5.8%) | 0.02 | 257  (4.3%) | 0.09 | 95  (6.0%) | 100  (6.3%) | -0.01 | 116  (4.1%) | 0.09 | 88  (5.6%) | 90  (5.8%) | -0.01 |
| antiepileptics | YES | 255  (15.3%) | 12735  (0.6%) | 0.56 | 235  (14.9%) | 727  (11.5%) | 0.10 | 2181  (4.0%) | 0.39 | 239  (15.1%) | 445  (14.0%) | 0.03 | 495  (8.2%) | 0.22 | 234  (14.8%) | 220  (13.9%) | 0.03 | 269  (9.5%) | 0.17 | 224  (14.4%) | 212  (13.6%) | 0.02 |
| antipsychotics | YES | 214  (12.8%) | 8685  (0.4%) | 0.52 | 202  (12.8%) | 646  (10.2%) | 0.08 | 2547  (4.6%) | 0.29 | 205  (12.9%) | 381  (12.0%) | 0.03 | 566  (9.4%) | 0.11 | 204  (12.9%) | 198  (12.5%) | 0.01 | 246  (8.7%) | 0.13 | 193  (12.4%) | 186  (11.9%) | 0.01 |
| anxiolytics | YES | 385  (23.1%) | 25277  (1.2%) | 0.71 | 361  (22.8%) | 1288  (20.4%) | 0.06 | 7678  (14.0%) | 0.23 | 365  (23.0%) | 699  (22.1%) | 0.02 | 1054  (17.5%) | 0.14 | 360  (22.8%) | 350  (22.2%) | 0.02 | 458  (16.3%) | 0.17 | 344  (22.1%) | 348  (22.3%) | -0.01 |
| Corticosteroid  (combination) | YES | 262  (15.7%) | 170758  (8.0%) | 0.24 | 250  (15.8%) | 1072  (17.0%) | -0.03 | 6906  (12.6%) | 0.09 | 251  (15.8%) | 473  (14.9%) | 0.03 | 778  (12.9%) | 0.08 | 251  (15.9%) | 231  (14.6%) | 0.04 | 423  (15.0%) | 0.02 | 241  (15.5%) | 243  (15.6%) | -0.00 |
| Fluticasone | YES | 12  (0.7%) | 9090  (0.4%) | 0.04 | 11  (0.7%) | 44  (0.7%) | 0.00 | 361  (0.7%) | 0.01 | 11  (0.7%) | 29  (0.9%) | -0.02 | 39  (0.6%) | 0.01 | 11  (0.7%) | 8  (0.5%) | 0.02 | 16  (0.6%) | 0.02 | 11  (0.7%) | 9  (0.6%) | 0.02 |
| Progesterone  (combination) | YES | 48  (2.9%) | 93074  (4.4%) | -0.08 | 44  (2.8%) | 369  (5.8%) | -0.15 | 2559  (4.7%) | -0.09 | 44  (2.8%) | 162  (5.1%) | -0.12 | 238  (4.0%) | -0.06 | 44  (2.8%) | 64  (4.1%) | -0.07 | 108  (3.8%) | -0.05 | 42  (2.7%) | 58  (3.7%) | -0.06 |
| antithyroid  (combination) | YES | 5  (0.3%) | 3051  (0.1%) | 0.03 | 5  (0.3%) | 49  (0.8%) | -0.06 | 80  (0.1%) | 0.03 | 5  (0.3%) | 5  (0.2%) | 0.03 | 9  (0.1%) | 0.03 | 5  (0.3%) | <5  (0%) | 0.04 | 5  (0.2%) | 0.03 | <5  (0%) | <5  (0%) | 0.00 |
| SSRI | YES | <5  (0%) | 54797  (2.6%) | -0.23 | <5  (0%) | 1219  (19.3%) | -0.69 | 54797  (100.0%) | . | <5  (0%) | 3170  (100.0%) | . | 1255  (20.9%) | -0.73 | <5  (0%) | 322  (20.4%) | -0.72 | 778  (27.6%) | -0.87 | <5  (0%) | 472  (30.3%) | -0.93 |
| venlafaxine | YES | <5  (0%) | 6008  (0.3%) | -0.08 | <5  (0%) | 249  (3.9%) | -0.29 | 1255  (2.3%) | -0.22 | <5  (0%) | 107  (3.4%) | -0.26 | 6008  (100.0%) | . | <5  (0%) | 1580  (100.0%) | . | 95  (3.4%) | -0.26 | <5  (0%) | 59  (3.8%) | -0.28 |

Propensity score [PS]-matched models were based on covariates covering comorbidity (up to five years prior LPM), comedication (during the relevant time period), hospital contacts, education, and income. For the complete list for the individual analyses, see supplementary material Table S5.

# Table H Number of events per thousand pregnancies (95% Wald confidence intervals)

| **Exposure cohort** | **Duloxetine  exposed** | **Duloxetine  non-exposed** | **SSRI  exposed** | **Venlafaxine  exposed** | **Duloxetine discontinuers** |
| --- | --- | --- | --- | --- | --- |
| **Major malformation** | 43 (32.8;53.2) | 38.9 (38.6;39.2) | 44.5 (42.4;46.5) | 45 (39.4;50.7) | 49.4 (41.5;57.3) |
| **Minor malformation** | 43.7 (33.4;53.9) | 31.1 (30.9;31.3) | 34.7 (32.9;36.5) | 38.7 (33.5;44.0) | 34.1 (27.4;40.7) |
| **Stillbirth** | 3 (0.4;5.6) | 3.6 (3.5;3.7) | 4.6 (4.0;5.2) | 7.3 (5.2;9.5) | 3.5 (1.4;5.8) |

Exposure definition: ≥1 redeemed prescriptions. SSRI: Selective serotonin reuptake inhibitors.

# Table I Major malformation, sensitivity analyses

| *Sensitivity analysis* | *Comparator* | *Model* | *Total N* | *Duloxetine events (%)* | *Comparator events (%)* | *OR (95% CI)* | *P-value* | *note* |
| --- | --- | --- | --- | --- | --- | --- | --- | --- |
| Two prescriptions |  |  |  |  |  |  |  |  |
|  | Duloxetine non-exposed | Unadjusted | 2,076,164 | 25 (4.7%) | 80,735 (3.9%) | 1.21 (0.81;1.81) | 0.349 |  |
|  |  | Adjusted | 1,966,871 | 24 (4.7%) | 75,918 (3.9%) | 1.04 (0.69;1.57) | 0.861 | 1 |
|  |  | PS-matched | 2,522 | 24 (4.8%) | 84 (4.2%) | 1.15 (0.72;1.83) | 0.556 | 2 |
|  | SSRI exposed | Unadjusted | 10,341 | 25 (4.7%) | 414 (4.2%) | 1.11 (0.74;1.68) | 0.615 |  |
|  |  | Adjusted | 9,775 | 24 (4.7%) | 387 (4.2%) | 1.08 (0.70;1.66) | 0.731 | 3 |
|  |  | PS-matched | 1,501 | 24 (4.8%) | 45 (4.5%) | 1.07 (0.65;1.77) | 0.796 | 2 |
|  | Venlafaxine exposed | Unadjusted | 2,422 | 25 (4.7%) | 88 (4.7%) | 1.00 (0.64;1.58) | 0.993 |  |
|  |  | Adjusted | 2,319 | 24 (4.7%) | 86 (4.7%) | 1.03 (0.64;1.67) | 0.900 | 4 |
|  |  | PS-matched | 1,001 | 24 (4.8%) | 17 (3.4%) | 1.41 (0.76;2.63) | 0.277 | 2 |
|  | Duloxetine discontinue | Unadjusted | 3,233 | 25 (4.7%) | 123 (4.6%) | 1.03 (0.66;1.59) | 0.908 |  |
|  |  | Adjusted | 3,090 | 24 (4.7%) | 113 (4.4%) | 1.06 (0.67;1.67) | 0.818 | 5 |
|  |  | PS-matched | 1,006 | 24 (4.8%) | 25 (5.0%) | 0.96 (0.53;1.72) | 0.882 | 2 |
| Overlap of exposure period and days duration |  |  |  |  |  |  |  |  |
|  | Duloxetine non-exposed | Unadjusted | 2,076,762 | 106 (5.0%) | 80,694 (3.9%) | 1.29 (1.06;1.57) | 0.011 |  |
|  |  | Adjusted | 1,967,442 | 101 (5.0%) | 75,878 (3.9%) | 1.09 (0.89;1.34) | 0.389 | 1 |
|  |  | PS-matched | 10,159 | 100 (4.9%) | 369 (4.5%) | 1.10 (0.87;1.38) | 0.420 | 6 |
|  | SSRI exposed | Unadjusted | 64,906 | 106 (5.0%) | 2,733 (4.4%) | 1.15 (0.94;1.40) | 0.181 |  |
|  |  | Adjusted | 61,784 | 101 (5.0%) | 2,563 (4.3%) | 1.12 (0.91;1.37) | 0.294 | 1 |
|  |  | PS-matched | 6,092 | 100 (4.9%) | 170 (4.2%) | 1.19 (0.93;1.54) | 0.170 | 6 |
|  | Venlafaxine exposed | Unadjusted | 9,387 | 106 (5.0%) | 325 (4.5%) | 1.11 (0.89;1.39) | 0.360 |  |
|  |  | Adjusted | 8,928 | 101 (5.0%) | 310 (4.5%) | 1.07 (0.85;1.36) | 0.571 | 7 |
|  |  | PS-matched | 4,060 | 100 (4.9%) | 75 (3.7%) | 1.35 (0.99;1.83) | 0.055 | 6 |
|  | Duloxetine discontinue | Unadjusted | 4,603 | 106 (5.0%) | 109 (4.4%) | 1.13 (0.86;1.48) | 0.394 |  |
|  |  | Adjusted | 4,402 | 101 (4.9%) | 99 (4.2%) | 1.19 (0.89;1.59) | 0.240 | 8 |
|  |  | PS-matched | 3,628 | 86 (4.7%) | 73 (4.0%) | 1.18 (0.86;1.61) | 0.303 | 6 |
| Limited to first observed pregnancy |  |  |  |  |  |  |  |  |
|  | Duloxetine non-exposed | Unadjusted | 1,288,702 | 43 (4.7%) | 50,533 (3.9%) | 1.22 (0.90;1.65) | 0.211 |  |
|  |  | Adjusted | 1,213,359 | 41 (4.7%) | 47,318 (3.9%) | 1.04 (0.76;1.43) | 0.809 | 1 |
|  |  | PS-matched | 4,337 | 40 (4.6%) | 148 (4.3%) | 1.08 (0.76;1.55) | 0.657 | 6 |
|  | SSRI exposed | Unadjusted | 24,400 | 43 (4.7%) | 1,073 (4.6%) | 1.04 (0.76;1.42) | 0.815 |  |
|  |  | Adjusted | 23,163 | 41 (4.7%) | 1,007 (4.5%) | 1.01 (0.73;1.40) | 0.944 | 1 |
|  |  | PS-matched | 2,589 | 40 (4.6%) | 77 (4.5%) | 1.04 (0.70;1.54) | 0.840 | 6 |
|  | Venlafaxine exposed | Unadjusted | 4,132 | 43 (4.7%) | 139 (4.3%) | 1.10 (0.78;1.56) | 0.588 |  |
|  |  | Adjusted | 3,938 | 41 (4.7%) | 134 (4.4%) | 1.08 (0.74;1.56) | 0.701 | 7 |
|  |  | PS-matched | 1,713 | 40 (4.7%) | 37 (4.3%) | 1.08 (0.69;1.70) | 0.729 | 6 |
|  | Duloxetine discontinue | Unadjusted | 2,704 | 43 (4.7%) | 85 (4.7%) | 1.00 (0.69;1.45) | 0.995 |  |
|  |  | Adjusted | 2,598 | 41 (4.7%) | 80 (4.6%) | 1.03 (0.69;1.52) | 0.891 | 9 |
|  |  | PS-matched | 1,693 | 40 (4.7%) | 39 (4.6%) | 1.05 (0.67;1.65) | 0.819 | 6 |
| Including BMI as covariate |  |  |  |  |  |  |  |  |
|  | Duloxetine non-exposed | Adjusted | 1,888,629 | 59 (4.3%) | 72,896 (3.9%) | 0.94 (0.72;1.22) | 0.636 | 10 |
|  |  | PS-matched | 6,880 | 58 (4.2%) | 266 (4.8%) | 0.89 (0.66;1.19) | 0.416 | 11 |
|  | SSRI exposed | Adjusted | 37,997 | 59 (4.3%) | 1,597 (4.4%) | 0.94 (0.72;1.24) | 0.678 | 10 |
|  |  | PS-matched | 4,129 | 58 (4.2%) | 97 (3.5%) | 1.20 (0.86;1.67) | 0.276 | 11 |
|  | Venlafaxine exposed | Adjusted | 6,191 | 59 (4.3%) | 216 (4.5%) | 0.95 (0.70;1.28) | 0.723 | 12 |
|  |  | PS-matched | 2,741 | 56 (4.1%) | 62 (4.5%) | 0.90 (0.62;1.30) | 0.571 | 11 |
|  | Duloxetine discontinue | Adjusted | 4,030 | 59 (4.3%) | 122 (4.6%) | 0.91 (0.66;1.26) | 0.575 | 13 |
|  |  | PS-matched | 2,729 | 58 (4.3%) | 61 (4.5%) | 0.95 (0.66;1.37) | 0.780 | 11 |

OR: odds ratio for major malformation for duloxetine vs comparator, CI: Wald 95% confidence intervals. PS-matched: propensity score matched analyses based on conditional logistic regression. SSRI: Selective serotonin reuptake inhibitors.

Note 1: Adjusted for data source (Sweden/Denmark), age (grouped), education, household income, year (grouped), psychiatric hospital (grouped), psychiatric outpatient visits (grouped), smoking, previous spontaneous abortions, previous stillbirth, gestational diabetes during index pregnancy, diabetes, hyper- or hypothyroidism, hypertension, obesity, renal failure, depression, affective disorder, anxiety or phobia, severe stress reaction, diabetic peripheral neuropathic pain, glucose-lowering, antihypertensive, fluconazole, estradiol, thyroid, NSAID, opioids, triptans, antiepileptics, antipsychotics, anxiolytics, corticosteroid (combination), progesterone (combination), antithyroid (combination).

Note 2: Propensity score based on data source (Sweden/Denmark), age (grouped), education, household income, year (grouped), psychiatric hospital (grouped), psychiatric outpatient visits (grouped), smoking, previous spontaneous abortions, previous stillbirth, gestational diabetes during index pregnancy, diabetes, hyper- or hypothyroidism, hypertension, obesity, depression, affective disorder, anxiety or phobia, severe stress reaction, antihypertensive, thyroid, opioids, triptans, antiepileptics, antipsychotics, anxiolytics, corticosteroid (combination).

Note 3: Adjusted for data source (Sweden/Denmark), age (grouped), education, household income, year (grouped), psychiatric hospital (grouped), psychiatric outpatient visits (grouped), smoking, previous spontaneous abortions, previous stillbirth, gestational diabetes during index pregnancy, diabetes, hyper- or hypothyroidism, hypertension, obesity, renal failure, depression, affective disorder, anxiety or phobia, severe stress reaction, diabetic peripheral neuropathic pain, glucose-lowering, antihypertensive, thyroid, NSAID, opioids, triptans, antiepileptics, antipsychotics, anxiolytics, corticosteroid (combination).

Note 4: Adjusted for data source (Sweden/Denmark), education, household income, year (grouped), psychiatric hospital (grouped), psychiatric outpatient visits (grouped), smoking, previous spontaneous abortions, previous stillbirth, gestational diabetes during index pregnancy, diabetes, hyper- or hypothyroidism, hypertension, obesity, depression, affective disorder, anxiety or phobia, severe stress reaction, glucose-lowering, antihypertensive, NSAID, opioids, antiepileptics, antipsychotics, anxiolytics, corticosteroid (combination).

Note 5: Adjusted for data source (Sweden/Denmark), age (grouped), household income, year (grouped), psychiatric hospital (grouped), psychiatric outpatient visits (grouped), smoking, previous spontaneous abortions, previous stillbirth, gestational diabetes during index pregnancy, diabetes, hyper- or hypothyroidism, obesity, depression, affective disorder, anxiety or phobia, severe stress reaction, antihypertensive, thyroid, NSAID, opioids, antiepileptics, antipsychotics, anxiolytics, corticosteroid (combination), progesterone (combination), antithyroid (combination).

Marker 6: Propensity score based on data source (Sweden/Denmark), age (grouped), education, Household income, year (grouped), psychiatric hospital (grouped), psychiatric outpatient visits (grouped), Smoking, previous spontaneous abortions, previous stillbirth, Gestational diabetes during index pregnancy, Diabetes, hyper- or hypothyroidism, obesity, renal failure, Depression, Affective disorder, anxiety or phobia, severe stress reaction, diabetic peripheral neuropathic pain, glucose-lowering, antihypertensive, fluconazole, thyroid, NSAID, opioids, triptanes, antiepileptics, antipsychotics, anxiolytics, corticosteroid (combination).

Marker 7: Adjusted for data source (Sweden/Denmark), age (grouped), education, Household income, year (grouped), psychiatric hospital (grouped), psychiatric outpatient visits (grouped), Smoking, previous spontaneous abortions, previous stillbirth, Gestational diabetes during index pregnancy, Diabetes, hyper- or hypothyroidism, obesity, Depression, Affective disorder, anxiety or phobia, severe stress reaction, glucose-lowering, antihypertensive , thyroid, NSAID, opioids, antiepileptics, antipsychotics, anxiolytics, corticosteroid (combination), progesterone (combination).

Marker 8: Adjusted for data source (Sweden/Denmark), age (grouped), Household income, year (grouped), psychiatric hospital (grouped), psychiatric outpatient visits (grouped), Smoking, previous spontaneous abortions, previous stillbirth, gestational diabetes during index pregnancy, diabetes, obesity, depression, Affective disorder, anxiety or phobia, severe stress reaction, glucose-lowering, antihypertensive, estradiol, thyroid, NSAID, opioids, antiepileptics, antipsychotics, anxiolytics, corticosteroid (combination), progesterone (combination).

Note 9: Adjusted for data source (Sweden/Denmark), age (grouped), household income, year (grouped), psychiatric hospital (grouped), psychiatric outpatient visits (grouped), smoking, previous spontaneous abortions, gestational diabetes during index pregnancy, diabetes, obesity, depression, affective disorder, anxiety or phobia, severe stress reaction, glucose-lowering, antihypertensive, estradiol, thyroid, NSAID, opioids, antiepileptics, antipsychotics, anxiolytics, corticosteroid (combination), progesterone (combination).

Note 10: Adjusted for data source (Sweden/Denmark), age (grouped), education, household income, year (grouped), psychiatric hospital (grouped), psychiatric outpatient visits (grouped), smoking, previous spontaneous abortions, previous stillbirth, gestational diabetes during index pregnancy, diabetes, hyper- or hypothyroidism, hypertension, obesity, renal failure, depression, affective disorder, anxiety or phobia, severe stress reaction, diabetic peripheral neuropathic pain, glucose-lowering, antihypertensive, fluconazole, estradiol, thyroid, NSAID, opioids, triptans, antiepileptics, antipsychotics, anxiolytics, corticosteroid (combination), progesterone (combination), antithyroid (combination), BMI (grouped).

Note 11: Propensity score based on data source (Sweden/Denmark), age (grouped), education, household income, year (grouped), psychiatric hospital (grouped), psychiatric outpatient visits (grouped), smoking, previous spontaneous abortions, previous stillbirth, gestational diabetes during index pregnancy, diabetes, hyper- or hypothyroidism, hypertension, obesity, renal failure, depression, affective disorder, anxiety or phobia, severe stress reaction, diabetic peripheral neuropathic pain, glucose-lowering, antihypertensive, fluconazole, thyroid, NSAID, opioids, triptans, antiepileptics, antipsychotics, anxiolytics, corticosteroid (combination), BMI (grouped).

Note 12: Adjusted for data source (Sweden/Denmark), age (grouped), education, household income, year (grouped), psychiatric hospital (grouped), psychiatric outpatient visits (grouped), smoking, previous spontaneous abortions, previous stillbirth, gestational diabetes during index pregnancy, diabetes, hyper- or hypothyroidism, hypertension, obesity, depression, affective disorder, anxiety or phobia, severe stress reaction, glucose-lowering, antihypertensive, thyroid, NSAID, opioids, antiepileptics, antipsychotics, anxiolytics, corticosteroid (combination), progesterone (combination), BMI (grouped).

Note 13: Adjusted for data source (Sweden/Denmark), age (grouped), household income, year (grouped), psychiatric hospital (grouped), psychiatric outpatient visits (grouped), smoking, previous spontaneous abortions, previous stillbirth, gestational diabetes during index pregnancy, diabetes, obesity, depression, affective disorder, anxiety or phobia, severe stress reaction, glucose-lowering, antihypertensive, estradiol, thyroid, NSAID, opioids, antiepileptics, antipsychotics, anxiolytics, corticosteroid (combination), progesterone (combination), BMI (grouped).

# Table J Minor malformation, sensitivity analyses

| *Analysis* | *Comparator* | *Model* | *Total N* | *Duloxetine events (%)* | *Comparator events (%)* | *OR (95% CI)* | *P-value* | *note* |
| --- | --- | --- | --- | --- | --- | --- | --- | --- |
| Two prescriptions |  |  |  |  |  |  |  |  |
|  | Duloxetine non-exposed | Unadjusted | 2,076,164 | 21 (3.9%) | 64,573 (3.1%) | 1.27 (0.82;1.97) | 0.278 |  |
|  |  | Adjusted | 1,966,871 | 20 (4.0%) | 60,930 (3.1%) | 1.04 (0.67;1.64) | 0.851 | 1 |
|  |  | PS-matched | 2,522 | 20 (4.0%) | 64 (3.2%) | 1.26 (0.76;2.10) | 0.377 | 2 |
|  | SSRI exposed | Unadjusted | 10,341 | 21 (3.9%) | 308 (3.1%) | 1.26 (0.80;1.98) | 0.315 |  |
|  |  | Adjusted | 9,775 | 20 (4.0%) | 294 (3.2%) | 1.18 (0.73;1.89) | 0.502 | 3 |
|  |  | PS-matched | 1,501 | 20 (4.0%) | 31 (3.1%) | 1.30 (0.73;2.29) | 0.369 | 2 |
|  | Venlafaxine exposed | Unadjusted | 2,422 | 21 (3.9%) | 73 (3.9%) | 1.02 (0.62;1.67) | 0.952 |  |
|  |  | Adjusted | 2,337 | 21 (4.1%) | 71 (3.9%) | 0.95 (0.57;1.61) | 0.861 | 4 |
|  |  | PS-matched | 1,001 | 20 (4.0%) | 12 (2.4%) | 1.67 (0.81;3.41) | 0.162 | 2 |
|  | Duloxetine discontinue | Unadjusted | 3,233 | 21 (3.9%) | 104 (3.9%) | 1.02 (0.63;1.64) | 0.938 |  |
|  |  | Adjusted | 3,090 | 21 (4.1%) | 101 (3.9%) | 1.01 (0.62;1.66) | 0.958 | 5 |
|  |  | PS-matched | 1,006 | 20 (4.0%) | 18 (3.6%) | 1.13 (0.57;2.27) | 0.724 | 2 |
| Overlap of exposure period and days duration |  |  |  |  |  |  |  |  |
|  | Duloxetine non-exposed | Unadjusted | 2,076,762 | 91 (4.3%) | 64,527 (3.1%) | 1.39 (1.12;1.71) | 0.002 |  |
|  |  | Adjusted | 1,967,442 | 87 (4.3%) | 60,887 (3.1%) | 1.14 (0.92;1.42) | 0.228 | 1 |
|  |  | PS-matched | 10,159 | 86 (4.3%) | 325 (4.0%) | 1.07 (0.84;1.37) | 0.575 | 6 |
|  | SSRI exposed | Unadjusted | 64,906 | 91 (4.3%) | 2,206 (3.5%) | 1.22 (0.98;1.51) | 0.069 |  |
|  |  | Adjusted | 61,784 | 87 (4.3%) | 2,095 (3.5%) | 1.17 (0.93;1.46) | 0.172 | 7 |
|  |  | PS-matched | 6,092 | 86 (4.2%) | 155 (3.8%) | 1.12 (0.86;1.47) | 0.402 | 6 |
|  | Venlafaxine exposed | Unadjusted | 9,387 | 91 (4.3%) | 293 (4.0%) | 1.05 (0.83;1.34) | 0.664 |  |
|  |  | Adjusted | 8,928 | 87 (4.3%) | 278 (4.0%) | 1.03 (0.80;1.32) | 0.844 | 8 |
|  |  | PS-matched | 4,060 | 86 (4.2%) | 77 (3.8%) | 1.12 (0.82;1.54) | 0.470 | 6 |
|  | Duloxetine discontinue | Unadjusted | 4,603 | 91 (4.3%) | 83 (3.4%) | 1.27 (0.94;1.73) | 0.117 |  |
|  |  | Adjusted | 4,371 | 87 (4.3%) | 80 (3.4%) | 1.25 (0.91;1.71) | 0.177 | 8 |
|  |  | PS-matched | 3,628 | 78 (4.3%) | 60 (3.3%) | 1.33 (0.94;1.90) | 0.110 | 6 |
| Limited to first observed pregnancy |  |  |  |  |  |  |  |  |
|  | Duloxetine non-exposed | Unadjusted | 1,288,702 | 38 (4.2%) | 40,250 (3.1%) | 1.35 (0.98;1.87) | 0.068 |  |
|  |  | Adjusted | 1,213,359 | 37 (4.3%) | 37,852 (3.1%) | 1.10 (0.79;1.54) | 0.566 | 1 |
|  |  | PS-matched | 4,337 | 36 (4.2%) | 154 (4.4%) | 0.93 (0.65;1.35) | 0.712 | 6 |
|  | SSRI exposed | Unadjusted | 24,400 | 38 (4.2%) | 801 (3.4%) | 1.24 (0.89;1.72) | 0.211 |  |
|  |  | Adjusted | 23,163 | 37 (4.3%) | 758 (3.4%) | 1.22 (0.87;1.72) | 0.252 | 9 |
|  |  | PS-matched | 2,589 | 36 (4.2%) | 73 (4.2%) | 0.99 (0.66;1.48) | 0.945 | 6 |
|  | Venlafaxine exposed | Unadjusted | 4,132 | 38 (4.2%) | 134 (4.2%) | 1.01 (0.70;1.45) | 0.976 |  |
|  |  | Adjusted | 3,938 | 37 (4.3%) | 129 (4.2%) | 0.93 (0.63;1.36) | 0.699 | 8 |
|  |  | PS-matched | 1,713 | 36 (4.2%) | 34 (4.0%) | 1.06 (0.66;1.72) | 0.806 | 6 |
|  | Duloxetine discontinue | Unadjusted | 2,704 | 38 (4.2%) | 66 (3.7%) | 1.14 (0.76;1.72) | 0.520 |  |
|  |  | Adjusted | 2,581 | 37 (4.3%) | 64 (3.7%) | 1.13 (0.74;1.72) | 0.581 | 10 |
|  |  | PS-matched | 1,693 | 35 (4.1%) | 25 (2.9%) | 1.40 (0.84;2.34) | 0.199 | 6 |
| Including BMI as covariate |  |  |  |  |  |  |  |  |
|  | Duloxetine non-exposed | Adjusted | 1,888,629 | 57 (4.1%) | 58,600 (3.1%) | 1.10 (0.84;1.43) | 0.499 | 11 |
|  |  | PS-matched | 6,880 | 56 (4.1%) | 205 (3.7%) | 1.12 (0.83;1.52) | 0.464 | 12 |
|  | SSRI exposed | Adjusted | 37,997 | 57 (4.1%) | 1,266 (3.5%) | 1.14 (0.86;1.50) | 0.362 | 13 |
|  |  | PS-matched | 4,129 | 56 (4.1%) | 101 (3.7%) | 1.12 (0.80;1.58) | 0.516 | 12 |
|  | Venlafaxine exposed | Adjusted | 6,191 | 57 (4.1%) | 186 (3.9%) | 1.00 (0.74;1.37) | 0.976 | 14 |
|  |  | PS-matched | 2,741 | 55 (4.0%) | 40 (2.9%) | 1.39 (0.92;2.12) | 0.118 | 12 |
|  | Duloxetine discontinue | Adjusted | 4,004 | 57 (4.1%) | 91 (3.5%) | 1.21 (0.86;1.71) | 0.275 | 15 |
|  |  | PS-matched | 2,729 | 55 (4.0%) | 50 (3.7%) | 1.08 (0.74;1.59) | 0.695 | 12 |

OR: odds ratio for minor malformation for duloxetine vs comparator, CI: Wald 95% confidence intervals. PS-matched: propensity score matched analyses based on conditional logistic regression. SSRI: Selective serotonin reuptake inhibitors.

Note 1: Adjusted for data source (Sweden/Denmark), age (grouped), education, household income, year (grouped), psychiatric hospital (grouped), psychiatric outpatient visits (grouped), smoking, previous spontaneous abortions, previous stillbirth, gestational diabetes during index pregnancy, diabetes, hyper- or hypothyroidism, hypertension, obesity, renal failure, depression, affective disorder, anxiety or phobia, severe stress reaction, diabetic peripheral neuropathic pain, glucose-lowering, antihypertensive, fluconazole, estradiol, thyroid, NSAID, opioids, triptans, antiepileptics, antipsychotics, anxiolytics, corticosteroid (combination), progesterone (combination), antithyroid (combination).

Note 2: Propensity score based on data source (Sweden/Denmark), age (grouped), education, household income, year (grouped), psychiatric hospital (grouped), psychiatric outpatient visits (grouped), smoking, previous spontaneous abortions, previous stillbirth, gestational diabetes during index pregnancy, diabetes, hyper- or hypothyroidism, hypertension, obesity, depression, affective disorder, anxiety or phobia, severe stress reaction, antihypertensive, thyroid, opioids, triptans, antiepileptics, antipsychotics, anxiolytics, corticosteroid (combination).
Note 3: Adjusted for data source (Sweden/Denmark), age (grouped), education, household income, year (grouped), psychiatric hospital (grouped), psychiatric outpatient visits (grouped), smoking, previous spontaneous abortions, previous stillbirth, gestational diabetes during index pregnancy, diabetes, hyper- or hypothyroidism, hypertension, obesity, renal failure, depression, affective disorder, anxiety or phobia, severe stress reaction, glucose-lowering, antihypertensive, fluconazole, estradiol, thyroid, NSAID, opioids, triptans, antiepileptics, antipsychotics, anxiolytics, corticosteroid (combination), progesterone (combination).

Note 4: Adjusted for data source (Sweden/Denmark), age (grouped), household income, year (grouped), psychiatric hospital (grouped), psychiatric outpatient visits (grouped), smoking, previous spontaneous abortions, gestational diabetes during index pregnancy, diabetes, hyper- or hypothyroidism, hypertension, obesity, depression, affective disorder, anxiety or phobia, severe stress reaction, NSAID, opioids, antiepileptics, antipsychotics, anxiolytics, corticosteroid (combination), progesterone (combination).

Note 5: Adjusted for data source (Sweden/Denmark), age (grouped), household income, year (grouped), psychiatric hospital (grouped), psychiatric outpatient visits (grouped), smoking, previous spontaneous abortions, gestational diabetes during index pregnancy, diabetes, hyper- or hypothyroidism, obesity, depression, affective disorder, anxiety or phobia, severe stress reaction, antihypertensive, fluconazole, thyroid, NSAID, opioids, triptans, antiepileptics, antipsychotics, anxiolytics, corticosteroid (combination), progesterone (combination).

Note 6: Propensity score based on data source (Sweden/Denmark), age (grouped), education, household income, year (grouped), psychiatric hospital (grouped), psychiatric outpatient visits (grouped), smoking, previous spontaneous abortions, previous stillbirth, gestational diabetes during index pregnancy, diabetes, hyper- or hypothyroidism, hypertension, obesity, renal failure, depression, affective disorder, anxiety or phobia, severe stress reaction, diabetic peripheral neuropathic pain, glucose-lowering, antihypertensive, fluconazole, thyroid, NSAID, opioids, triptans, antiepileptics, antipsychotics, anxiolytics, corticosteroid (combination).

Note 7: Adjusted for data source (Sweden/Denmark), age (grouped), education, household income, year (grouped), psychiatric hospital (grouped), psychiatric outpatient visits (grouped), smoking, previous spontaneous abortions, previous stillbirth, gestational diabetes during index pregnancy, diabetes, hyper- or hypothyroidism, hypertension, obesity, renal failure, depression, affective disorder, anxiety or phobia, severe stress reaction, diabetic peripheral neuropathic pain, glucose-lowering, antihypertensive, fluconazole, estradiol, thyroid, NSAID, opioids, triptans, antiepileptics, antipsychotics, anxiolytics, corticosteroid (combination), progesterone (combination).

Note 8: Adjusted for data source (Sweden/Denmark), age (grouped), education, household income, year (grouped), psychiatric hospital (grouped), psychiatric outpatient visits (grouped), smoking, previous spontaneous abortions, gestational diabetes during index pregnancy, diabetes, hyper- or hypothyroidism, hypertension, obesity, depression, affective disorder, anxiety or phobia, severe stress reaction, glucose-lowering, antihypertensive, fluconazole, thyroid, NSAID, opioids, triptans, antiepileptics, antipsychotics, anxiolytics, corticosteroid (combination), progesterone (combination).

Note 9: Adjusted for data source (Sweden/Denmark), age (grouped), education, household income, year (grouped), psychiatric hospital (grouped), psychiatric outpatient visits (grouped), smoking, previous spontaneous abortions, previous stillbirth, gestational diabetes during index pregnancy, diabetes, hyper- or hypothyroidism, hypertension, obesity, renal failure, depression, affective disorder, anxiety or phobia, severe stress reaction, diabetic peripheral neuropathic pain, glucose-lowering, antihypertensive, estradiol, thyroid, NSAID, opioids, triptans, antiepileptics, antipsychotics, anxiolytics, corticosteroid (combination), progesterone (combination).

Note 10: Adjusted for data source (Sweden/Denmark), age (grouped), education, household income, year (grouped), psychiatric hospital (grouped), psychiatric outpatient visits (grouped), smoking, previous spontaneous abortions, previous stillbirth, gestational diabetes during index pregnancy, diabetes, hyper- or hypothyroidism, hypertension, obesity, renal failure, depression, affective disorder, anxiety or phobia, severe stress reaction, diabetic peripheral neuropathic pain, glucose-lowering, antihypertensive, fluconazole, estradiol, thyroid, NSAID, opioids, triptans, antiepileptics, antipsychotics, anxiolytics, corticosteroid (combination), progesterone (combination).
Note 11: Adjusted for data source (Sweden/Denmark), age (grouped), education, household income, year (grouped), psychiatric hospital (grouped), psychiatric outpatient visits (grouped), smoking, previous spontaneous abortions, previous stillbirth, gestational diabetes during index pregnancy, diabetes, hyper- or hypothyroidism, hypertension, obesity, renal failure, depression, affective disorder, anxiety or phobia, severe stress reaction, diabetic peripheral neuropathic pain, glucose-lowering, antihypertensive, fluconazole, estradiol, thyroid, NSAID, opioids, triptans, antiepileptics, antipsychotics, anxiolytics, corticosteroid (combination), progesterone (combination), antithyroid (combination), BMI (grouped).

Note 12: Adjusted for data source (Sweden/Denmark), age (grouped), education, household income, year (grouped), psychiatric hospital (grouped), psychiatric outpatient visits (grouped), smoking, previous spontaneous abortions, previous stillbirth, gestational diabetes during index pregnancy, diabetes, hyper- or hypothyroidism, hypertension, obesity, renal failure, depression, affective disorder, anxiety or phobia, severe stress reaction, diabetic peripheral neuropathic pain, glucose-lowering, antihypertensive, fluconazole, thyroid, NSAID, opioids, triptans, antiepileptics, antipsychotics, anxiolytics, corticosteroid (combination), BMI (grouped).

Note 13: Adjusted for data source (Sweden/Denmark), age (grouped), education, household income, year (grouped), psychiatric hospital (grouped), psychiatric outpatient visits (grouped), smoking, previous spontaneous abortions, previous stillbirth, gestational diabetes during index pregnancy, diabetes, hyper- or hypothyroidism, hypertension, obesity, renal failure, depression, affective disorder, anxiety or phobia, severe stress reaction, diabetic peripheral neuropathic pain, glucose-lowering, antihypertensive, estradiol, thyroid, NSAID, opioids, triptans, antiepileptics, antipsychotics, anxiolytics, corticosteroid (combination), progesterone (combination), BMI (grouped).

Note 14: Adjusted for data source (Sweden/Denmark), age (grouped), education, household income, year (grouped), psychiatric hospital (grouped), psychiatric outpatient visits (grouped), smoking, previous spontaneous abortions, gestational diabetes during index pregnancy, diabetes, hyper- or hypothyroidism, hypertension, obesity, depression, affective disorder, anxiety or phobia, severe stress reaction, glucose-lowering, antihypertensive, fluconazole, thyroid, NSAID, opioids, triptans, antiepileptics, antipsychotics, anxiolytics, corticosteroid (combination), progesterone (combination), BMI (grouped).

Note 15: Adjusted for data source (Sweden/Denmark), age (grouped), education, household income, year (grouped), psychiatric hospital (grouped), psychiatric outpatient visits (grouped), smoking, previous spontaneous abortions, gestational diabetes during index pregnancy, diabetes, hyper- or hypothyroidism, hypertension, obesity, depression, affective disorder, anxiety or phobia, severe stress reaction, glucose-lowering, antihypertensive, fluconazole, estradiol, thyroid, NSAID, opioids, triptans, antiepileptics, antipsychotics, anxiolytics, corticosteroid (combination), progesterone (combination), BMI (grouped).

# Table K Malformation subtype: Heart defect

| *Analysis* | *Comparator* | *Model* | *Total N* | *Duloxetine events (%)* | *Comparator events (%)* | *OR (95% CI)* | *P-value* | *note* |
| --- | --- | --- | --- | --- | --- | --- | --- | --- |
| Main analyses (one prescription) |  |  |  |  |  |  |  |  |
|  | Duloxetine non-exposed | Unadjusted | 2,076,164 | 25 (1.7%) | 27,508 (1.3%) | 1.25 (0.84;1.86) | 0.266 |  |
|  |  | Adjusted | 1,966,871 | 23 (1.6%) | 25,755 (1.3%) | 0.95 (0.63;1.44) | 0.817 | 1 |
|  |  | PS-matched | 7,189 | 23 (1.6%) | 91 (1.6%) | 1.01 (0.64;1.60) | 0.962 | 6 |
|  | SSRI exposed | Unadjusted | 41,471 | 25 (1.7%) | 709 (1.8%) | 0.93 (0.62;1.39) | 0.726 |  |
|  |  | Adjusted | 39,444 | 23 (1.6%) | 666 (1.8%) | 0.84 (0.55;1.29) | 0.433 | 2 |
|  |  | PS-matched | 4,311 | 23 (1.6%) | 58 (2.0%) | 0.79 (0.49;1.29) | 0.344 | 6 |
|  | Venlafaxine exposed | Unadjusted | 6,752 | 25 (1.7%) | 92 (1.8%) | 0.94 (0.60;1.47) | 0.788 |  |
|  |  | Adjusted | 6,432 | 23 (1.6%) | 90 (1.8%) | 0.86 (0.54;1.39) | 0.545 | 3 |
|  |  | PS-matched | 2,858 | 23 (1.6%) | 29 (2.0%) | 0.78 (0.44;1.38) | 0.388 | 6 |
|  | Duloxetine discontinue | Unadjusted | 4,388 | 25 (1.7%) | 42 (1.5%) | 1.13 (0.69;1.87) | 0.620 |  |
|  |  | Adjusted | 4,168 | 23 (1.6%) | 37 (1.4%) | 1.19 (0.70;2.05) | 0.522 | 4 |
|  |  | PS-matched | 2,869 | 23 (1.6%) | 25 (1.7%) | 0.92 (0.51;1.63) | 0.768 | 6 |
| Two prescriptions |  |  |  |  |  |  |  |  |
|  | Duloxetine non-exposed | Unadjusted | 2,076,164 | 10 (1.9%) | 27,523 (1.3%) | 1.42 (0.76;2.65) | 0.271 |  |
|  |  | Adjusted | 1,966,871 | 9 (1.8%) | 25,769 (1.3%) | 1.05 (0.54;2.04) | 0.885 | 1 |
|  |  | PS-matched | 2,522 | 9 (1.8%) | 30 (1.5%) | 1.20 (0.57;2.55) | 0.629 | 9 |
|  | SSRI exposed | Unadjusted | 10,341 | 10 (1.9%) | 160 (1.6%) | 1.15 (0.60;2.19) | 0.674 |  |
|  |  | Adjusted | 9,775 | 9 (1.8%) | 153 (1.7%) | 0.99 (0.49;1.97) | 0.967 | 7 |
|  |  | PS-matched | 1,501 | 9 (1.8%) | 20 (2.0%) | 0.90 (0.40;2.00) | 0.789 | 9 |
|  | Venlafaxine exposed | Unadjusted | 2,422 | 10 (1.9%) | 37 (2.0%) | 0.95 (0.47;1.93) | 0.892 |  |
|  |  | Adjusted | 2,319 | 9 (1.8%) | 36 (2.0%) | 0.92 (0.42;1.99) | 0.832 | 3 |
|  |  | PS-matched | 1,001 | 9 (1.8%) | 7 (1.4%) | 1.29 (0.48;3.45) | 0.618 | 9 |
|  | Duloxetine discontinue | Unadjusted | 3,233 | 10 (1.9%) | 40 (1.5%) | 1.27 (0.63;2.55) | 0.509 |  |
|  |  | Adjusted | 3,074 | 9 (1.8%) | 35 (1.4%) | 1.23 (0.57;2.64) | 0.595 | 8 |
|  |  | PS-matched | 1,006 | 9 (1.8%) | 6 (1.2%) | 1.50 (0.53;4.21) | 0.442 | 9 |
| Overlap of exposure period and days duration |  |  |  |  |  |  |  |  |
|  | Duloxetine non-exposed | Unadjusted | 2,076,762 | 35 (1.6%) | 27,508 (1.3%) | 1.24 (0.89;1.73) | 0.210 |  |
|  |  | Adjusted | 1,967,442 | 32 (1.6%) | 25,755 (1.3%) | 0.92 (0.65;1.31) | 0.646 | 1 |
|  |  | PS-matched | 10,159 | 32 (1.6%) | 148 (1.8%) | 0.87 (0.59;1.29) | 0.494 | 6 |
|  | SSRI exposed | Unadjusted | 64,906 | 35 (1.6%) | 1,031 (1.6%) | 1.00 (0.71;1.40) | 0.982 |  |
|  |  | Adjusted | 61,784 | 32 (1.6%) | 971 (1.6%) | 0.88 (0.62;1.27) | 0.497 | 2 |
|  |  | PS-matched | 6,092 | 32 (1.6%) | 65 (1.6%) | 0.99 (0.65;1.52) | 0.971 | 6 |
|  | Venlafaxine exposed | Unadjusted | 9,387 | 35 (1.6%) | 133 (1.8%) | 0.89 (0.61;1.30) | 0.543 |  |
|  |  | Adjusted | 8,928 | 32 (1.6%) | 130 (1.9%) | 0.76 (0.51;1.14) | 0.189 | 3 |
|  |  | PS-matched | 4,060 | 32 (1.6%) | 37 (1.8%) | 0.86 (0.54;1.39) | 0.548 | 6 |
|  | Duloxetine discontinue | Unadjusted | 4,603 | 35 (1.6%) | 37 (1.5%) | 1.09 (0.68;1.74) | 0.714 |  |
|  |  | Adjusted | 4,371 | 32 (1.6%) | 33 (1.4%) | 1.05 (0.63;1.74) | 0.853 | 4 |
|  |  | PS-matched | 3,628 | 26 (1.4%) | 25 (1.4%) | 1.04 (0.60;1.80) | 0.889 | 6 |
| Limited to first observed pregnancy |  |  |  |  |  |  |  |  |
|  | Duloxetine non-exposed | Unadjusted | 1,288,702 | 16 (1.8%) | 16,949 (1.3%) | 1.34 (0.82;2.20) | 0.240 |  |
|  |  | Adjusted | 1,213,359 | 14 (1.6%) | 15,812 (1.3%) | 0.98 (0.58;1.67) | 0.948 | 1 |
|  |  | PS-matched | 4,337 | 14 (1.6%) | 52 (1.5%) | 1.08 (0.60;1.94) | 0.806 | 6 |
|  | SSRI exposed | Unadjusted | 24,400 | 16 (1.8%) | 414 (1.8%) | 1.00 (0.60;1.65) | 0.996 |  |
|  |  | Adjusted | 23,163 | 14 (1.6%) | 393 (1.8%) | 0.85 (0.49;1.46) | 0.550 | 16 |
|  |  | PS-matched | 2,589 | 14 (1.6%) | 31 (1.8%) | 0.90 (0.48;1.70) | 0.752 | 6 |
|  | Venlafaxine exposed | Unadjusted | 4,132 | 16 (1.8%) | 54 (1.7%) | 1.05 (0.60;1.85) | 0.861 |  |
|  |  | Adjusted | 3,938 | 14 (1.6%) | 53 (1.7%) | 0.92 (0.50;1.70) | 0.785 | 3 |
|  |  | PS-matched | 1,713 | 14 (1.6%) | 19 (2.2%) | 0.74 (0.37;1.47) | 0.386 | 6 |
|  | Duloxetine discontinue | Unadjusted | 2,704 | 16 (1.8%) | 30 (1.7%) | 1.05 (0.57;1.94) | 0.866 |  |
|  |  | Adjusted | 2,581 | 14 (1.6%) | 28 (1.6%) | 1.00 (0.51;1.95) | 0.990 | 10 |
|  |  | PS-matched | 1,693 | 14 (1.7%) | 16 (1.9%) | 0.88 (0.43;1.79) | 0.715 | 6 |
| Including BMI as covariate |  |  |  |  |  |  |  |  |
|  | Duloxetine non-exposed | Adjusted | 1,888,629 | 23 (1.7%) | 24,737 (1.3%) | 0.99 (0.65;1.50) | 0.972 | 11 |
|  |  | PS-matched | 6,880 | 23 (1.7%) | 100 (1.8%) | 0.95 (0.60;1.50) | 0.817 | 15 |
|  | SSRI exposed | Adjusted | 37,997 | 23 (1.7%) | 639 (1.7%) | 0.88 (0.58;1.35) | 0.563 | 12 |
|  |  | PS-matched | 4,129 | 23 (1.7%) | 40 (1.4%) | 1.15 (0.69;1.92) | 0.593 | 15 |
|  | Venlafaxine exposed | Adjusted | 6,191 | 23 (1.7%) | 89 (1.8%) | 0.87 (0.54;1.40) | 0.565 | 13 |
|  |  | PS-matched | 2,741 | 23 (1.7%) | 25 (1.8%) | 0.92 (0.52;1.62) | 0.773 | 15 |
|  | Duloxetine discontinue | Adjusted | 4,004 | 23 (1.7%) | 34 (1.3%) | 1.30 (0.75;2.25) | 0.352 | 14 |
|  |  | PS-matched | 2,729 | 23 (1.7%) | 19 (1.4%) | 1.21 (0.66;2.22) | 0.538 | 15 |

PS-matched: propensity score matched analyses based on conditional logistic regression.

OR: odds ratio for heart defect for duloxetine vs comparators. CI: Wald 95% confidence intervals. SSRI: Selective serotonin reuptake inhibitors.

Note1: Adjusted for data source (Sweden/Denmark), age (grouped), education (grouped), household income, year (grouped), psychiatric hospital, psychiatric outpatient, smoking, previous spontaneous abortion, previous stillbirths, gestational diabetes during index pregnancy, diabetes, hyper- or hypothyroidism, hypertension, obesity, renal failure, depression, affective, anxiety or phobia, severe stress reaction, diabetic peripheral neuropathic pain, glucose-lowering, antihypertensive, fluconazole, estradiol, thyroid, NSAID, opioids, triptans, antiepileptics, antipsychotics, anxiolytics, corticosteroid (combination), progesterone (combination), antithyroid (combination).

Note2: Adjusted for data source (Sweden/Denmark), age (grouped), education (grouped), household income, year (grouped), psychiatric hospital, psychiatric outpatient, smoking, previous spontaneous abortion, previous stillbirths, gestational diabetes during index pregnancy, diabetes, hyper- or hypothyroidism, hypertension, obesity, renal failure, depression, affective, anxiety or phobia, severe stress reaction, glucose-lowering, antihypertensive, fluconazole, estradiol, thyroid, NSAID, opioids, triptans, antiepileptics, antipsychotics, anxiolytics, corticosteroid (combination), progesterone (combination), antithyroid (combination).

Note3: Adjusted for data source (Sweden/Denmark), age (grouped), education (grouped), household income, year (grouped), psychiatric hospital, psychiatric outpatient, smoking, previous spontaneous abortion, previous stillbirths, gestational diabetes during index pregnancy, diabetes, hyper- or hypothyroidism, hypertension, obesity, depression, affective, anxiety or phobia, severe stress reaction, glucose-lowering, antihypertensive, fluconazole, estradiol, thyroid, NSAID, opioids, triptans, antiepileptics, antipsychotics, anxiolytics, corticosteroid (combination), progesterone (combination).

Note4: Adjusted for data source (Sweden/Denmark), age (grouped), education (grouped), household income, year (grouped), psychiatric hospital, psychiatric outpatient, smoking, previous spontaneous abortion, gestational diabetes during index pregnancy, diabetes, hyper- or hypothyroidism, obesity, depression, affective, anxiety or phobia, severe stress reaction, glucose-lowering, antihypertensive, fluconazole, estradiol, thyroid, NSAID, opioids, triptans, antiepileptics, antipsychotics, anxiolytics, corticosteroid (combination), progesterone (combination), antithyroid (combination).

Note6: Propensity score based on data source (Sweden/Denmark), age (grouped), education (grouped), household income, year (grouped), psychiatric hospital, psychiatric outpatient, smoking, previous spontaneous abortion, previous stillbirths, gestational diabetes during index pregnancy, diabetes, hyper- or hypothyroidism, hypertension, obesity, renal failure, depression, affective, anxiety or phobia, severe stress reaction, diabetic peripheral neuropathic pain, glucose-lowering, antihypertensive, fluconazole, thyroid, NSAID, opioids, triptans, antiepileptics, antipsychotics, anxiolytics, corticosteroid (combination).

Note7: Adjusted for data source (Sweden/Denmark), age (grouped), education, household income, year (grouped), psychiatric hospital (grouped), psychiatric outpatient visits (grouped), smoking, previous spontaneous abortions, previous stillbirth, gestational diabetes during index pregnancy, diabetes, hyper- or hypothyroidism, hypertension, obesity, renal failure, depression, affective disorder, anxiety or phobia, severe stress reaction, glucose-lowering, antihypertensive, fluconazole, estradiol, thyroid, NSAID, opioids, triptans, antiepileptics, antipsychotics, anxiolytics, corticosteroid (combination), progesterone (combination).

Note8: Adjusted for data source (Sweden/Denmark), age (grouped), education, household income, year (grouped), psychiatric outpatient visits (grouped), smoking, previous spontaneous abortions, gestational diabetes during index pregnancy, hyper- or hypothyroidism, obesity, depression, affective disorder, anxiety or phobia, severe stress reaction, antihypertensive, fluconazole, estradiol, thyroid, NSAID, opioids, triptans, antiepileptics, antipsychotics, anxiolytics, corticosteroid (combination), progesterone (combination), antithyroid (combination).

Note9: Propensity score based on data source (Sweden/Denmark), age (grouped), education, household income, year (grouped), psychiatric hospital (grouped), psychiatric outpatient visits (grouped), smoking, previous spontaneous abortions, previous stillbirth, gestational diabetes during index pregnancy, diabetes, hyper- or hypothyroidism, hypertension, obesity, depression, affective disorder, anxiety or phobia, severe stress reaction, antihypertensive, thyroid, opioids, triptans, antiepileptics, antipsychotics, anxiolytics, corticosteroid (combination)

Note10: Adjusted for data source (Sweden/Denmark), age (grouped), education, household income, year (grouped), psychiatric hospital (grouped), psychiatric outpatient visits (grouped), smoking, previous spontaneous abortions, gestational diabetes during index pregnancy, hyper- or hypothyroidism, obesity, depression, affective disorder, anxiety or phobia, severe stress reaction, glucose-lowering, antihypertensive, fluconazole, estradiol, thyroid, NSAID, opioids, triptans, antiepileptics, antipsychotics, anxiolytics, corticosteroid (combination), progesterone (combination).

Note16: Adjusted for data source (Sweden/Denmark), age (grouped), education, household income, year (grouped), psychiatric hospital (grouped), psychiatric outpatient visits (grouped), smoking, previous spontaneous abortions, gestational diabetes during index pregnancy, diabetes, hyper- or hypothyroidism, hypertension, obesity, renal failure, depression, affective disorder, anxiety or phobia, severe stress reaction, glucose-lowering, antihypertensive, fluconazole, estradiol, thyroid, NSAID, opioids, triptans, antiepileptics, antipsychotics, anxiolytics, corticosteroid (combination), progesterone (combination).
Note11: Adjusted for data source (Sweden/Denmark), age (grouped), education, household income, year (grouped), psychiatric hospital (grouped), psychiatric outpatient visits (grouped), smoking, previous spontaneous abortions, previous stillbirth, gestational diabetes during index pregnancy, diabetes, hyper- or hypothyroidism, hypertension, obesity, renal failure, depression, affective disorder, anxiety or phobia, severe stress reaction, diabetic peripheral neuropathic pain, glucose-lowering, antihypertensive, fluconazole, estradiol, thyroid, NSAID, opioids, triptans, antiepileptics, antipsychotics, anxiolytics, corticosteroid (combination), progesterone (combination), antithyroid (combination), BMI (grouped).
Note12: Adjusted for data source (Sweden/Denmark), age (grouped), education, household income, year (grouped), psychiatric hospital (grouped), psychiatric outpatient visits (grouped), smoking, previous spontaneous abortions, previous stillbirth, gestational diabetes during index pregnancy, diabetes, hyper- or hypothyroidism, hypertension, obesity, renal failure, depression, affective disorder, anxiety or phobia, severe stress reaction, glucose-lowering, antihypertensive, fluconazole, estradiol, thyroid, NSAID, opioids, triptans, antiepileptics, antipsychotics, anxiolytics, corticosteroid (combination), progesterone (combination), antithyroid (combination), BMI (grouped).
Note13: Adjusted for data source (Sweden/Denmark), age (grouped), education, household income, year (grouped), psychiatric hospital (grouped), psychiatric outpatient visits (grouped), smoking, previous spontaneous abortions, previous stillbirth, gestational diabetes during index pregnancy, diabetes, hyper- or hypothyroidism, hypertension, obesity, depression, affective disorder, anxiety or phobia, severe stress reaction, glucose-lowering, antihypertensive, fluconazole, estradiol, thyroid, NSAID, opioids, triptans, antiepileptics, antipsychotics, anxiolytics, corticosteroid (combination), progesterone (combination), BMI (grouped).
Note14: Adjusted for data source (Sweden/Denmark), age (grouped), education, household income, year (grouped), psychiatric hospital (grouped), psychiatric outpatient visits (grouped), smoking, previous spontaneous abortions, gestational diabetes during index pregnancy, diabetes, hyper- or hypothyroidism, obesity, depression, affective disorder, anxiety or phobia, severe stress reaction, glucose-lowering, antihypertensive, fluconazole, estradiol, thyroid, NSAID, opioids, triptans, antiepileptics, antipsychotics, anxiolytics, corticosteroid (combination), progesterone (combination), antithyroid (combination), BMI (grouped).
Note15: Propensity score based on data source (Sweden/Denmark), age (grouped), education, household income, year (grouped), psychiatric hospital (grouped), psychiatric outpatient visits (grouped), smoking, previous spontaneous abortions, previous stillbirth, gestational diabetes during index pregnancy, diabetes, hyper- or hypothyroidism, hypertension, obesity, renal failure, depression, affective disorder, anxiety or phobia, severe stress reaction, diabetic peripheral neuropathic pain, glucose-lowering, antihypertensive, fluconazole, thyroid, NSAID, opioids, triptans, antiepileptics, antipsychotics, anxiolytics, corticosteroid (combination), BMI (grouped).

# **Table L** Malformation subtype: Digestive system

| *Analysis* | *Comparator* | *Model* | *Total N* | *Duloxetine events (%)* | *Comparator events (%)* | *OR (95% CI)* | *P-value* | *note* |
| --- | --- | --- | --- | --- | --- | --- | --- | --- |
| Main analyses (one prescription) |  |  |  |  |  |  |  |  |
|  | Duloxetine non-exposed | Unadjusted | 2,076,164 | 25 (1.7%) | 19,290 (0.9%) | 1.79 (1.21;2.66) | 0.004 |  |
|  |  | PS-matched | 7,189 | 24 (1.7%) | 77 (1.3%) | 1.25 (0.79;1.99) | 0.341 | 2 |
|  | SSRI exposed | Unadjusted | 41,471 | 25 (1.7%) | 460 (1.2%) | 1.44 (0.96;2.17) | 0.076 |  |
|  |  | PS-matched | 4,311 | 24 (1.7%) | 35 (1.2%) | 1.38 (0.82;2.33) | 0.229 | 2 |
|  | Venlafaxine exposed | Unadjusted | 6,752 | 25 (1.7%) | 69 (1.3%) | 1.26 (0.79;2.00) | 0.326 |  |
|  |  | PS-matched | 2,858 | 24 (1.7%) | 19 (1.3%) | 1.26 (0.69;2.31) | 0.447 | 2 |
|  | Duloxetine discontinue | Unadjusted | 4,388 | 25 (1.7%) | 32 (1.1%) | 1.49 (0.88;2.53) | 0.135 |  |
|  |  | PS-matched | 2,869 | 24 (1.7%) | 15 (1.0%) | 1.60 (0.84;3.05) | 0.153 | 2 |
| Overlap of exposure period and days duration |  |  |  |  |  |  |  |  |
|  | Duloxetine non-exposed | Unadjusted | 2,076,762 | 38 (1.8%) | 19,289 (0.9%) | 1.93 (1.40;2.66) | <0.001 |  |
|  |  | PS-matched | 10,159 | 36 (1.8%) | 111 (1.4%) | 1.31 (0.89;1.91) | 0.170 | 2 |
|  | SSRI exposed | Unadjusted | 64,906 | 38 (1.8%) | 740 (1.2%) | 1.52 (1.09;2.11) | 0.013 |  |
|  |  | PS-matched | 6,092 | 36 (1.8%) | 45 (1.1%) | 1.61 (1.04;2.50) | 0.034 | 2 |
|  | Venlafaxine exposed | Unadjusted | 9,387 | 38 (1.8%) | 98 (1.4%) | 1.32 (0.90;1.93) | 0.150 |  |
|  |  | PS-matched | 4,060 | 36 (1.8%) | 29 (1.4%) | 1.24 (0.76;2.02) | 0.386 | 2 |
|  | Duloxetine discontinue | Unadjusted | 4,603 | 38 (1.8%) | 21 (0.9%) | 2.10 (1.23;3.60) | 0.007 |  |
|  |  | PS-matched | 3,628 | 32 (1.8%) | 11 (0.6%) | 3.10 (1.52;6.32) | 0.002 | 2 |
| Including BMI as covariate |  |  |  |  |  |  |  |  |
|  | Duloxetine non-exposed | PS-matched | 6,880 | 22 (1.6%) | 82 (1.5%) | 1.10 (0.69;1.77) | 0.691 | 3 |
|  | SSRI exposed | PS-matched | 4,129 | 22 (1.6%) | 29 (1.1%) | 1.53 (0.87;2.68) | 0.136 | 3 |
|  | Venlafaxine exposed | PS-matched | 2,741 | 22 (1.6%) | 11 (0.8%) | 2.10 (0.99;4.46) | 0.053 | 3 |
|  | Duloxetine discontinue | PS-matched | 2,729 | 21 (1.5%) | 16 (1.2%) | 1.25 (0.65;2.41) | 0.506 | 3 |

PS-matched: propensity score matched analyses based on conditional logistic regression.

OR: odds ratio for major malformations in the digestive system for duloxetine vs comparators. CI: Wald 95% confidence intervals. SSRI: Selective serotonin reuptake inhibitors.

Note 2: Propensity score based on data source (Sweden/Denmark), age (grouped), education (grouped), household income, year (grouped), psychiatric hospital, psychiatric outpatient, smoking, previous spontaneous abortion, previous stillbirths, gestational diabetes during index pregnancy, diabetes, hyper- or hypothyroidism, hypertension, obesity, renal failure, depression, affective, anxiety or phobia, severe stress reaction, diabetic peripheral neuropathic pain, glucose-lowering, antihypertensive, fluconazole, thyroid, NSAID, opioids, triptans, antiepileptics, antipsychotics, anxiolytics, corticosteroid (combination).

Note 3: Propensity score based on data source (Sweden/Denmark), age (grouped), education (grouped), household income, year (grouped), psychiatric hospital, psychiatric outpatient, smoking, previous spontaneous abortion, previous stillbirths, gestational diabetes during index pregnancy, diabetes, hyper- or hypothyroidism, hypertension, obesity, renal failure, depression, affective, anxiety or phobia, severe stress reaction, diabetic peripheral neuropathic pain, glucose-lowering, antihypertensive, fluconazole, thyroid, NSAID, opioids, triptans, antiepileptics, antipsychotics, anxiolytics, corticosteroid (combination), BMI (grouped).

# Table M Malformation subtype: Ear, face and neck

| *Analysis* | *Comparator* | *Model* | *Total N* | *Duloxetine events (%)* | *Comparator events (%)* | *OR (95% CI)* | *P-value* | *note* |
| --- | --- | --- | --- | --- | --- | --- | --- | --- |
| Main analyses (one prescription) |  |  |  |  |  |  |  |  |
|  | Duloxetine non-exposed | Unadjusted | 2,076,164 | 6 (0.4%) | 4,912 (0.2%) | 1.68 (0.75;3.74) | 0.206 |  |
|  |  | PS-matched | 7,189 | 6 (0.4%) | 15 (0.3%) | 1.62 (0.62;4.24) | 0.325 | 2 |
|  | SSRI exposed | Unadjusted | 41,471 | 6 (0.4%) | 101 (0.3%) | 1.57 (0.69;3.59) | 0.282 |  |
|  |  | PS-matched | 4,311 | 6 (0.4%) | 5 (0.2%) | 2.40 (0.73;7.86) | 0.148 | 2 |
|  | Venlafaxine exposed | Unadjusted | 6,752 | 6 (0.4%) | 10 (0.2%) | 2.08 (0.76;5.74) | 0.156 |  |
|  |  | PS-matched | 2,858 | 6 (0.4%) | <5 (0%) | 3.00 (0.61;14.86) | 0.178 | 2 |
|  | Duloxetine discontinue | Unadjusted | 4,388 | 6 (0.4%) | <5 (0%) | 5.73 (1.15;28.40) | 0.033 |  |
|  |  | PS-matched | 2,869 | 6 (0.4%) | <5 (0%) | 6.00 (0.72;49.84) | 0.097 | 2 |
| Overlap of exposure period and days duration |  |  |  |  |  |  |  |  |
|  | Duloxetine non-exposed | Unadjusted | 2,076,762 | 6 (0.3%) | 4,912 (0.2%) | 1.19 (0.53;2.64) | 0.678 |  |
|  |  | PS-matched | 10,159 | 6 (0.3%) | 24 (0.3%) | 1.00 (0.41;2.46) | 1.000 | 2 |
|  | SSRI exposed | Unadjusted | 64,906 | 6 (0.3%) | 156 (0.2%) | 1.13 (0.50;2.55) | 0.771 |  |
|  |  | PS-matched | 6,092 | 6 (0.3%) | 6 (0.1%) | 2.00 (0.65;6.20) | 0.230 | 2 |
|  | Venlafaxine exposed | Unadjusted | 9,387 | 6 (0.3%) | 13 (0.2%) | 1.57 (0.59;4.12) | 0.364 |  |
|  |  | PS-matched | 4,060 | 6 (0.3%) | <5 (0%) | 1.50 (0.42;5.32) | 0.530 | 2 |
|  | Duloxetine discontinue | Unadjusted | 4,603 | 6 (0.3%) | <5 (0%) | 2.31 (0.58;9.24) | 0.237 |  |
|  |  | PS-matched | 3,628 | 5 (0.3%) | <5 (0%) | 2.50 (0.49;12.89) | 0.273 | 2 |
| Including BMI as covariate |  |  |  |  |  |  |  |  |
|  | Duloxetine non-exposed | PS-matched | 6,880 | 6 (0.4%) | 13 (0.2%) | 1.85 (0.70;4.86) | 0.214 | 3 |
|  | SSRI exposed | PS-matched | 4,129 | 6 (0.4%) | 8 (0.3%) | 1.50 (0.52;4.32) | 0.453 | 3 |
|  | Venlafaxine exposed | PS-matched | 2,741 | 6 (0.4%) | <5 (0%) | 6.00 (0.72;49.84) | 0.097 | 3 |
|  | Duloxetine discontinue | PS-matched | 2,729 | 6 (0.4%) | <5 (0%) | 6.00 (0.72;49.84) | <0.001 | 3 |

OR: odds ratio for major malformations in the ear, face or neck for duloxetine vs comparators. CI: Wald 95% confidence intervals. SSRI: Selective serotonin reuptake inhibitors.

Note2: Propensity score based on data source (Sweden/Denmark), age (grouped), education (grouped), household income, year (grouped), psychiatric hospital, psychiatric outpatient, smoking, previous spontaneous abortion, previous stillbirths, gestational diabetes during index pregnancy, diabetes, hyper- or hypothyroidism, hypertension, obesity, renal failure, depression, affective, anxiety or phobia, severe stress reaction, diabetic peripheral neuropathic pain, glucose-lowering, antihypertensive, fluconazole, thyroid, NSAID, opioids, triptans, antiepileptics, antipsychotics, anxiolytics, corticosteroid (combination).

Note3: Propensity score based on data source (Sweden/Denmark), age (grouped), education (grouped), household income, year (grouped), psychiatric hospital, psychiatric outpatient, smoking, previous spontaneous abortion, previous stillbirths, gestational diabetes during index pregnancy, diabetes, hyper- or hypothyroidism, hypertension, obesity, renal failure, depression, affective, anxiety or phobia, severe stress reaction, diabetic peripheral neuropathic pain, glucose-lowering, antihypertensive, fluconazole, thyroid, NSAID, opioids, triptans, antiepileptics, antipsychotics, anxiolytics, corticosteroid (combination), BMI (grouped).

# **Table N** Malformation subtype: Eye

| *Analysis* | *Comparator* | *Model* | *Total N* | *Duloxetine events (%)* | *Comparator events (%)* | *OR (95% CI)* | *P-value* | *Note* |
| --- | --- | --- | --- | --- | --- | --- | --- | --- |
| Main analyses (one prescription) |  |  |  |  |  |  |  |  |
|  | Duloxetine non-exposed | Unadjusted | 2,076,164 | 5 (0.3%) | 5,405 (0.3%) | 1.27 (0.53;3.06) | 0.593 |  |
|  |  | PS-matched | 7,189 | 5 (0.3%) | 16 (0.3%) | 1.25 (0.46;3.41) | 0.663 | 2 |
|  | SSRI exposed | Unadjusted | 41,471 | 5 (0.3%) | 98 (0.2%) | 1.35 (0.55;3.32) | 0.512 |  |
|  |  | PS-matched | 4,311 | 5 (0.3%) | 6 (0.2%) | 1.67 (0.51;5.46) | 0.399 | 2 |
|  | Venlafaxine exposed | Unadjusted | 6,752 | 5 (0.3%) | 13 (0.2%) | 1.33 (0.48;3.75) | 0.584 |  |
|  |  | PS-matched | 2,858 | 5 (0.3%) | 7 (0.5%) | 0.71 (0.23;2.25) | 0.566 | 2 |
|  | Duloxetine discontinue | Unadjusted | 4,388 | 5 (0.3%) | 16 (0.6%) | 0.59 (0.22;1.62) | 0.309 |  |
|  |  | PS-matched | 2,869 | 5 (0.3%) | 8 (0.6%) | 0.63 (0.20;1.91) | 0.410 | 2 |
| Overlap of exposure period and days duration |  |  |  |  |  |  |  |  |
|  | Duloxetine non-exposed | Unadjusted | 2,076,762 | 11 (0.5%) | 5,404 (0.3%) | 1.98 (1.09;3.58) | 0.024 |  |
|  |  | PS-matched | 10,159 | 10 (0.5%) | 22 (0.3%) | 1.90 (0.90;4.04) | 0.094 | 2 |
|  | SSRI exposed | Unadjusted | 64,906 | 11 (0.5%) | 158 (0.3%) | 2.05 (1.11;3.78) | 0.022 |  |
|  |  | PS-matched | 6,092 | 10 (0.5%) | 16 (0.4%) | 1.22 (0.55;2.68) | 0.629 | 2 |
|  | Venlafaxine exposed | Unadjusted | 9,387 | 11 (0.5%) | 16 (0.2%) | 2.34 (1.08;5.04) | 0.031 |  |
|  |  | PS-matched | 4,060 | 10 (0.5%) | <5 (0%) | 2.50 (0.78;7.97) | 0.121 | 2 |
|  | Duloxetine discontinue | Unadjusted | 4,603 | 11 (0.5%) | 10 (0.4%) | 1.27 (0.54;2.99) | 0.587 |  |
|  |  | PS-matched | 3,628 | 7 (0.4%) | 7 (0.4%) | 1.00 (0.35;2.85) | 1 | 2 |
| Including BMI as covariate |  |  |  |  |  |  |  |  |
|  | Duloxetine non-exposed | PS-matched | 6,880 | <5 (0%) | 13 (0.2%) | 1.00 (0.28;3.54) | 1 | 3 |
|  | SSRI exposed | PS-matched | 4,129 | <5 (0%) | 12 (0.4%) | 0.50 (0.14;1.77) | 0.283 | 3 |
|  | Venlafaxine exposed | PS-matched | 2,741 | <5 (0%) | <5 (0%) | 0.50 (0.09;2.73) | 0.423 | 3 |
|  | Duloxetine discontinue | PS-matched | 2,729 | <5 (0%) | 6 (0.4%) | 0.50 (0.13;2.00) | 0.327 | 3 |

PS-matched: propensity score matched analyses based on conditional logistic regression.

OR: odds ratio for major malformations in the eye for duloxetine vs comparators. CI: Wald 95% confidence intervals. SSRI: Selective serotonin reuptake inhibitors.

Note2: Propensity score based on data source (Sweden/Denmark), age (grouped), education (grouped), household income, year (grouped), psychiatric hospital, psychiatric outpatient, smoking, previous spontaneous abortion, previous stillbirths, gestational diabetes during index pregnancy, diabetes, hyper- or hypothyroidism, hypertension, obesity, renal failure, depression, affective, anxiety or phobia, severe stress reaction, diabetic peripheral neuropathic pain, glucose-lowering, antihypertensive, fluconazole, thyroid, NSAID, opioids, triptans, antiepileptics, antipsychotics, anxiolytics, corticosteroid (combination).

Note3: Propensity score based on data source (Sweden/Denmark), age (grouped), education (grouped), household income, year (grouped), psychiatric hospital, psychiatric outpatient, smoking, previous spontaneous abortion, previous stillbirths, gestational diabetes during index pregnancy, diabetes, hyper- or hypothyroidism, hypertension, obesity, renal failure, depression, affective, anxiety or phobia, severe stress reaction, diabetic peripheral neuropathic pain, glucose-lowering, antihypertensive, fluconazole, thyroid, NSAID, opioids, triptans, antiepileptics, antipsychotics, anxiolytics, corticosteroid (combination), BMI (grouped).

# **Table O** Malformation subtype: Genital

| *Analysis* | *Comparator* | *Model* | *Total N* | *Duloxetine events (%)* | *Comparator events (%)* | *OR (95% CI)* | *P-value* | *Note* |
| --- | --- | --- | --- | --- | --- | --- | --- | --- |
| Main analyses (one prescription) |  |  |  |  |  |  |  |  |
|  | Duloxetine non-exposed | Unadjusted | 2,076,164 | 6 (0.4%) | 8,998 (0.4%) | 0.92 (0.41;2.04) | 0.834 |  |
|  |  | PS-matched | 7,189 | 6 (0.4%) | 32 (0.6%) | 0.75 (0.31;1.80) | 0.515 | 2 |
|  | SSRI exposed | Unadjusted | 41,471 | 6 (0.4%) | 180 (0.5%) | 0.88 (0.39;1.99) | 0.760 |  |
|  |  | PS-matched | 4,311 | 6 (0.4%) | 9 (0.3%) | 1.33 (0.47;3.75) | 0.585 | 2 |
|  | Venlafaxine exposed | Unadjusted | 6,752 | 6 (0.4%) | 13 (0.2%) | 1.60 (0.61;4.22) | 0.341 |  |
|  |  | PS-matched | 2,858 | 6 (0.4%) | <5 (0%) | 1.50 (0.42;5.32) | 0.530 | 2 |
|  | Duloxetine discontinue | Unadjusted | 4,388 | 6 (0.4%) | 15 (0.5%) | 0.76 (0.29;1.96) | 0.571 |  |
|  |  | PS-matched | 2,869 | 6 (0.4%) | 7 (0.5%) | 0.86 (0.29;2.55) | 0.782 | 2 |
| Overlap of exposure period and days duration |  |  |  |  |  |  |  |  |
|  | Duloxetine non-exposed | Unadjusted | 2,076,762 | 13 (0.6%) | 8,998 (0.4%) | 1.41 (0.82;2.43) | 0.220 |  |
|  |  | PS-matched | 10,159 | 13 (0.6%) | 38 (0.5%) | 1.37 (0.73;2.59) | 0.327 | 2 |
|  | SSRI exposed | Unadjusted | 64,906 | 13 (0.6%) | 282 (0.4%) | 1.36 (0.78;2.37) | 0.284 |  |
|  |  | PS-matched | 6,092 | 13 (0.6%) | 17 (0.4%) | 1.52 (0.72;3.17) | 0.269 | 2 |
|  | Venlafaxine exposed | Unadjusted | 9,387 | 13 (0.6%) | 21 (0.3%) | 2.10 (1.05;4.21) | 0.035 |  |
|  |  | PS-matched | 4,060 | 13 (0.6%) | 5 (0.2%) | 2.60 (0.93;7.29) | 0.069 | 2 |
|  | Duloxetine discontinue | Unadjusted | 4,603 | 13 (0.6%) | 10 (0.4%) | 1.50 (0.66;3.43) | 0.336 |  |
|  |  | PS-matched | 3,628 | 12 (0.7%) | 7 (0.4%) | 1.71 (0.67;4.35) | 0.257 | 2 |
| Including BMI as covariate |  |  |  |  |  |  |  |  |
|  | Duloxetine non-exposed | PS-matched | 6,880 | 5 (0.4%) | 28 (0.5%) | 0.71 (0.27;1.85) | 0.485 | 3 |
|  | SSRI exposed | PS-matched | 4,129 | 5 (0.4%) | 19 (0.7%) | 0.53 (0.20;1.41) | 0.202 | 3 |
|  | Venlafaxine exposed | PS-matched | 2,741 | 5 (0.4%) | <5 (0%) | 1.67 (0.40;6.97) | 0.484 | 3 |
|  | Duloxetine discontinue | PS-matched | 2,729 | 5 (0.4%) | 10 (0.7%) | 0.50 (0.17;1.46) | 0.206 | 3 |

PS-matched: propensity score matched analyses based on conditional logistic regression.

OR: odds ratio for major malformations in the genitals for duloxetine vs comparators. CI: Wald 95% confidence intervals. SSRI: Selective serotonin reuptake inhibitors.

Note2: Propensity score based on data source (Sweden/Denmark), age (grouped), education (grouped), household income, year (grouped), psychiatric hospital, psychiatric outpatient, smoking, previous spontaneous abortion, previous stillbirths, gestational diabetes during index pregnancy, diabetes, hyper- or hypothyroidism, hypertension, obesity, renal failure, depression, affective, anxiety or phobia, severe stress reaction, diabetic peripheral neuropathic pain, glucose-lowering, antihypertensive, fluconazole, thyroid, NSAID, opioids, triptans, antiepileptics, antipsychotics, anxiolytics, corticosteroid (combination).

Note3: Propensity score based on data source (Sweden/Denmark), age (grouped), education (grouped), household income, year (grouped), psychiatric hospital, psychiatric outpatient, smoking, previous spontaneous abortion, previous stillbirths, gestational diabetes during index pregnancy, diabetes, hyper- or hypothyroidism, hypertension, obesity, renal failure, depression, affective, anxiety or phobia, severe stress reaction, diabetic peripheral neuropathic pain, glucose-lowering, antihypertensive, fluconazole, thyroid, NSAID, opioids, triptans, antiepileptics, antipsychotics, anxiolytics, corticosteroid (combination), BMI (grouped).

# Table P Malformation subtype: Abdominal wall

| *Analysis* | *Comparator* | *Model* | *Total N* | *Duloxetine events (%)* | *Comparator events (%)* | *OR (95% CI)* | *P-value* | *Note* |
| --- | --- | --- | --- | --- | --- | --- | --- | --- |
| Main analyses (one prescription) |  |  |  |  |  |  |  |  |
|  | Duloxetine non-exposed | Unadjusted | 2,076,164 | <5 (0%) | 531 (0.0%) | 5.19 (1.29;20.77) | 0.020 |  |
|  |  | PS-matched | 7,189 | <5 (0%) | <5 (0%) | 2.00 (0.37;10.92) | 0.423 | 2 |
|  | SSRI exposed | Unadjusted | 41,471 | <5 (0%) | 20 (0.1%) | 2.65 (0.62;11.33) | 0.190 |  |
|  |  | PS-matched | 4,311 | <5 (0%) | <5 (0%) | 4.00 (0.36;44.11) | 0.258 | 2 |
|  | Venlafaxine exposed | Unadjusted | 6,752 | <5 (0%) | <5 (0%) | 416E3 (0.00;94E212) | 0.958 |  |
|  |  | PS-matched | 2,858 | <5 (0%) | <5 (0%) | 416E3 (0.00;94E212) | <0.001 | 2 |
|  | Duloxetine discontinue | Unadjusted | 4,388 | <5 (0%) | <5 (0%) | 174E3 (0.00;15E186) | 0.955 |  |
|  |  | PS-matched | 2,869 | <5 (0%) | <5 (0%) | 174E3 (0.00;15E186) | <0.001 | 2 |
| Overlap of exposure period and days duration |  |  |  |  |  |  |  |  |
|  | Duloxetine non-exposed | Unadjusted | 2,076,762 | <5 (0%) | 531 (0.0%) | 3.66 (0.91;14.66) | 0.067 |  |
|  |  | PS-matched | 10,159 | <5 (0%) | <5 (0%) | 4.00 (0.56;28.40) | 0.166 | 2 |
|  | SSRI exposed | Unadjusted | 64,906 | <5 (0%) | 27 (0.0%) | 2.17 (0.52;9.15) | 0.289 |  |
|  |  | PS-matched | 6,092 | <5 (0%) | <5 (0%) | 2.00 (0.28;14.20) | 0.488 | 2 |
|  | Venlafaxine exposed | Unadjusted | 9,387 | <5 (0%) | <5 (0%) | 6.78 (0.61;74.83) | 0.118 |  |
|  |  | PS-matched | 4,060 | <5 (0%) | <5 (0%) | 2.00 (0.18;22.06) | 0.571 | 2 |
|  | Duloxetine discontinue | Unadjusted | 4,603 | <5 (0%) | <5 (0%) | 47456 (0.00;61E125) | 0.940 |  |
|  |  | PS-matched | 3,628 | <5 (0%) | <5 (0%) | 47456 (0.00;61E125) | <0.001 | 2 |
| Including BMI as covariate |  |  |  |  |  |  |  |  |
|  | Duloxetine non-exposed | PS-matched | 6,880 | <5 (0%) | <5 (0%) | 2.00 (0.37;10.92) | <0.001 | 3 |
|  | SSRI exposed | PS-matched | 4,129 | <5 (0%) | <5 (0%) | 4.00 (0.36;44.11) | <0.001 | 3 |
|  | Venlafaxine exposed | PS-matched | 2,741 | <5 (0%) | <5 (0%) | 416E3 (0.00;94E212) | <0.001 | 3 |
|  | Duloxetine discontinue | PS-matched | 2,729 | <5 (0%) | <5 (0%) | 174E3 (0.00;15E186) | <0.001 | 3 |

PS-matched: propensity score matched analyses based on conditional logistic regression.
OR: odds ratio for major malformations in the abdominal wall for duloxetine vs comparators. CI: Wald 95% confidence intervals. SSRI: Selective serotonin reuptake inhibitors.

Note2: Propensity score based on data source (Sweden/Denmark), age (grouped), education (grouped), household income, year (grouped), psychiatric hospital, psychiatric outpatient, smoking, previous spontaneous abortion, previous stillbirths, gestational diabetes during index pregnancy, diabetes, hyper- or hypothyroidism, hypertension, obesity, renal failure, depression, affective, anxiety or phobia, severe stress reaction, diabetic peripheral neuropathic pain, glucose-lowering, antihypertensive, fluconazole, thyroid, NSAID, opioids, triptans, antiepileptics, antipsychotics, anxiolytics, corticosteroid (combination).

Note3: Propensity score based on data source (Sweden/Denmark), age (grouped), education (grouped), household income, year (grouped), psychiatric hospital, psychiatric outpatient, smoking, previous spontaneous abortion, previous stillbirths, gestational diabetes during index pregnancy, diabetes, hyper- or hypothyroidism, hypertension, obesity, renal failure, depression, affective, anxiety or phobia, severe stress reaction, diabetic peripheral neuropathic pain, glucose-lowering, antihypertensive, fluconazole, thyroid, NSAID, opioids, triptans, antiepileptics, antipsychotics, anxiolytics, corticosteroid (combination), BMI (grouped).

# Table Q Malformation subtype: Limb

| *Analysis* | *Comparator* | *Model* | *Total N* | *Duloxetine events (%)* | *Comparator events (%)* | *OR (95% CI)* | *P-value* | *Note* |
| --- | --- | --- | --- | --- | --- | --- | --- | --- |
| Main analyses (one prescription) |  |  |  |  |  |  |  |  |
|  | Duloxetine non-exposed | Unadjusted | 2,076,164 | 23 (1.5%) | 38,703 (1.9%) | 0.81 (0.54;1.23) | 0.324 |  |
|  |  | PS-matched | 7,189 | 21 (1.5%) | 125 (2.2%) | 0.67 (0.42;1.06) | 0.089 | 2 |
|  | SSRI exposed | Unadjusted | 41,471 | 23 (1.5%) | 816 (2.0%) | 0.74 (0.49;1.13) | 0.160 |  |
|  |  | PS-matched | 4,311 | 21 (1.5%) | 48 (1.7%) | 0.88 (0.52;1.46) | 0.610 | 2 |
|  | Venlafaxine exposed | Unadjusted | 6,752 | 23 (1.5%) | 119 (2.3%) | 0.66 (0.42;1.04) | 0.075 |  |
|  |  | PS-matched | 2,858 | 20 (1.4%) | 28 (2.0%) | 0.71 (0.40;1.27) | 0.250 | 2 |
|  | Duloxetine discontinue | Unadjusted | 4,388 | 23 (1.5%) | 63 (2.2%) | 0.69 (0.43;1.12) | 0.131 |  |
|  |  | PS-matched | 2,869 | 20 (1.4%) | 36 (2.5%) | 0.56 (0.32;0.96) | 0.035 | 2 |
| Overlap of exposure period and days duration |  |  |  |  |  |  |  |  |
|  | Duloxetine non-exposed | Unadjusted | 2,076,762 | 39 (1.8%) | 38,703 (1.9%) | 0.98 (0.71;1.34) | 0.886 |  |
|  |  | PS-matched | 10,159 | 36 (1.8%) | 193 (2.4%) | 0.75 (0.52;1.08) | 0.119 | 2 |
|  | SSRI exposed | Unadjusted | 64,906 | 39 (1.8%) | 1,284 (2.0%) | 0.89 (0.64;1.23) | 0.474 |  |
|  |  | PS-matched | 6,092 | 36 (1.8%) | 94 (2.3%) | 0.76 (0.51;1.12) | 0.164 | 2 |
|  | Venlafaxine exposed | Unadjusted | 9,387 | 39 (1.8%) | 170 (2.3%) | 0.77 (0.54;1.10) | 0.152 |  |
|  |  | PS-matched | 4,060 | 36 (1.8%) | 40 (2.0%) | 0.89 (0.56;1.42) | 0.638 | 2 |
|  | Duloxetine discontinue | Unadjusted | 4,603 | 39 (1.8%) | 50 (2.0%) | 0.90 (0.59;1.37) | 0.613 |  |
|  |  | PS-matched | 3,628 | 31 (1.7%) | 38 (2.1%) | 0.81 (0.50;1.31) | 0.393 | 2 |
| Including BMI as covariate |  |  |  |  |  |  |  |  |
|  | Duloxetine non-exposed | PS-matched | 6,880 | 19 (1.4%) | 115 (2.1%) | 0.66 (0.41;1.08) | 0.098 | 3 |
|  | SSRI exposed | PS-matched | 4,129 | 19 (1.4%) | 43 (1.6%) | 0.88 (0.51;1.52) | 0.648 | 3 |
|  | Venlafaxine exposed | PS-matched | 2,741 | 19 (1.4%) | 25 (1.8%) | 0.76 (0.42;1.38) | 0.367 | 3 |
|  | Duloxetine discontinue | PS-matched | 2,729 | 19 (1.4%) | 33 (2.4%) | 0.58 (0.33;1.01) | 0.055 | 3 |

PS-matched: propensity score matched analyses based on conditional logistic regression.

OR: odds ratio for major malformations in the limb for duloxetine vs comparators. CI: Wald 95% confidence intervals. SSRI: Selective serotonin reuptake inhibitors.

Note2: Propensity score based on data source (Sweden/Denmark), age (grouped), education (grouped), household income, year (grouped), psychiatric hospital, psychiatric outpatient, smoking, previous spontaneous abortion, previous stillbirths, gestational diabetes during index pregnancy, diabetes, hyper- or hypothyroidism, hypertension, obesity, renal failure, depression, affective, anxiety or phobia, severe stress reaction, diabetic peripheral neuropathic pain, glucose-lowering, antihypertensive, fluconazole, thyroid, NSAID, opioids, triptans, antiepileptics, antipsychotics, anxiolytics, corticosteroid (combination).

Note3: Propensity score based on data source (Sweden/Denmark), age (grouped), education (grouped), household income, year (grouped), psychiatric hospital, psychiatric outpatient, smoking, previous spontaneous abortion, previous stillbirths, gestational diabetes during index pregnancy, diabetes, hyper- or hypothyroidism, hypertension, obesity, renal failure, depression, affective, anxiety or phobia, severe stress reaction, diabetic peripheral neuropathic pain, glucose-lowering, antihypertensive, fluconazole, thyroid, NSAID, opioids, triptans, antiepileptics, antipsychotics, anxiolytics, corticosteroid (combination), BMI (grouped).

# Table R Malformation subtype: Nervous system

| *Analysis* | *Comparator* | *Model* | *Total N* | *Duloxetine events (%)* | *Comparator events (%)* | *OR (95% CI)* | *P-value* | *Note* |
| --- | --- | --- | --- | --- | --- | --- | --- | --- |
| Main analyses (one prescription) |  |  |  |  |  |  |  |  |
|  | Duloxetine non-exposed | Unadjusted | 2,076,164 | <5 (0%) | 2,517 (0.1%) | 1.64 (0.53;5.08) | 0.394 |  |
|  |  | PS-matched | 7,189 | <5 (0%) | 12 (0.2%) | 1.00 (0.28;3.54) | 1 | 2 |
|  | SSRI exposed | Unadjusted | 41,471 | <5 (0%) | 54 (0.1%) | 1.47 (0.46;4.70) | 0.517 |  |
|  |  | PS-matched | 4,311 | <5 (0%) | 5 (0.2%) | 1.20 (0.29;5.02) | 0.803 | 2 |
|  | Venlafaxine exposed | Unadjusted | 6,752 | <5 (0%) | 10 (0.2%) | 1.04 (0.29;3.78) | 0.952 |  |
|  |  | PS-matched | 2,858 | <5 (0%) | <5 (0%) | 1.04 (0.29;3.78) | <0.001 | 2 |
|  | Duloxetine discontinue | Unadjusted | 4,388 | <5 (0%) | 8 (0.3%) | 0.71 (0.19;2.69) | 0.618 |  |
|  |  | PS-matched | 2,869 | <5 (0%) | <5 (0%) | 1.50 (0.25;8.98) | 0.657 | 2 |
| Overlap of exposure period and days duration |  |  |  |  |  |  |  |  |
|  | Duloxetine non-exposed | Unadjusted | 2,076,762 | 5 (0.2%) | 2,517 (0.1%) | 1.93 (0.80;4.64) | 0.142 |  |
|  |  | PS-matched | 10,159 | <5 (0%) | 11 (0.1%) | 1.45 (0.46;4.57) | 0.521 | 2 |
|  | SSRI exposed | Unadjusted | 64,906 | 5 (0.2%) | 93 (0.1%) | 1.58 (0.64;3.89) | 0.320 |  |
|  |  | PS-matched | 6,092 | <5 (0%) | 13 (0.3%) | 0.62 (0.20;1.89) | 0.396 | 2 |
|  | Venlafaxine exposed | Unadjusted | 9,387 | 5 (0.2%) | 11 (0.2%) | 1.54 (0.54;4.44) | 0.423 |  |
|  |  | PS-matched | 4,060 | <5 (0%) | <5 (0%) | 4.00 (0.45;35.79) | 0.215 | 2 |
|  | Duloxetine discontinue | Unadjusted | 4,603 | 5 (0.2%) | 6 (0.2%) | 0.96 (0.29;3.15) | 0.946 |  |
|  |  | PS-matched | 3,628 | <5 (0%) | 5 (0.3%) | 0.60 (0.14;2.51) | 0.484 | 2 |
| Including BMI as covariate |  |  |  |  |  |  |  |  |
|  | Duloxetine non-exposed | PS-matched | 6,880 | <5 (0%) | 5 (0.1%) | 1.60 (0.31;8.25) | 0.574 | 3 |
|  | SSRI exposed | PS-matched | 4,129 | <5 (0%) | <5 (0%) | 1.33 (0.22;7.98) | 0.753 | 3 |
|  | Venlafaxine exposed | PS-matched | 2,741 | <5 (0%) | <5 (0%) | 0.67 (0.11;3.99) | 0.657 | 3 |
|  | Duloxetine discontinue | PS-matched | 2,729 | <5 (0%) | <5 (0%) | 2.00 (0.18;22.06) | 0.571 | 3 |

PS-matched: propensity score matched analyses based on conditional logistic regression.
OR: odds ratio for major malformations in the nervous system for duloxetine vs comparators. CI: Wald 95% confidence intervals. SSRI: Selective serotonin reuptake inhibitors.

Note2: Propensity score based on data source (Sweden/Denmark), age (grouped), education (grouped), household income, year (grouped), psychiatric hospital, psychiatric outpatient, smoking, previous spontaneous abortion, previous stillbirths, gestational diabetes during index pregnancy, diabetes, hyper- or hypothyroidism, hypertension, obesity, renal failure, depression, affective, anxiety or phobia, severe stress reaction, diabetic peripheral neuropathic pain, glucose-lowering, antihypertensive, fluconazole, thyroid, NSAID, opioids, triptans, antiepileptics, antipsychotics, anxiolytics, corticosteroid (combination).

Note3: Propensity score based on data source (Sweden/Denmark), age (grouped), education (grouped), household income, year (grouped), psychiatric hospital, psychiatric outpatient, smoking, previous spontaneous abortion, previous stillbirths, gestational diabetes during index pregnancy, diabetes, hyper- or hypothyroidism, hypertension, obesity, renal failure, depression, affective, anxiety or phobia, severe stress reaction, diabetic peripheral neuropathic pain, glucose-lowering, antihypertensive, fluconazole, thyroid, NSAID, opioids, triptans, antiepileptics, antipsychotics, anxiolytics, corticosteroid (combination), BMI (grouped).

# Table S Malformation subtype: Oro-facial clefts

| *Analysis* | *Comparator* | *Model* | *Total N* | *Duloxetine events (%)* | *Comparator events (%)* | *OR (95% CI)* | *P-value* | *Note* |
| --- | --- | --- | --- | --- | --- | --- | --- | --- |
| Main analyses (one prescription) |  |  |  |  |  |  |  |  |
|  | Duloxetine non-exposed | Unadjusted | 2,076,164 | <5 (0%) | 3,526 (0.2%) | 0.78 (0.19;3.12) | 0.723 |  |
|  |  | PS-matched | 7,189 | <5 (0%) | 6 (0.1%) | 1.33 (0.27;6.61) | 0.725 | 2 |
|  | SSRI exposed | Unadjusted | 41,471 | <5 (0%) | 73 (0.2%) | 0.72 (0.18;2.95) | 0.653 |  |
|  |  | PS-matched | 4,311 | <5 (0%) | <5 (0%) | 1.00 (0.18;5.46) | 1 | 2 |
|  | Venlafaxine exposed | Unadjusted | 6,752 | <5 (0%) | 13 (0.2%) | 0.53 (0.12;2.36) | 0.407 |  |
|  |  | PS-matched | 2,858 | <5 (0%) | <5 (0%) | 2.00 (0.18;22.06) | 0.571 | 2 |
|  | Duloxetine discontinue | Unadjusted | 4,388 | <5 (0%) | 5 (0.2%) | 0.76 (0.15;3.92) | 0.744 |  |
|  |  | PS-matched | 2,869 | <5 (0%) | <5 (0%) | 1.00 (0.14;7.10) | 1 | 2 |
| Overlap of exposure period and days duration |  |  |  |  |  |  |  |  |
|  | Duloxetine non-exposed | Unadjusted | 2,076,762 | <5 (0%) | 3,526 (0.2%) | 0.56 (0.14;2.21) | 0.404 |  |
|  |  | PS-matched | 10,159 | <5 (0%) | 10 (0.1%) | 0.80 (0.18;3.65) | 0.773 | 2 |
|  | SSRI exposed | Unadjusted | 64,906 | <5 (0%) | 112 (0.2%) | 0.52 (0.13;2.12) | 0.365 |  |
|  |  | PS-matched | 6,092 | <5 (0%) | 9 (0.2%) | 0.41 (0.09;1.92) | 0.259 | 2 |
|  | Venlafaxine exposed | Unadjusted | 9,387 | <5 (0%) | 17 (0.2%) | 0.40 (0.09;1.72) | 0.218 |  |
|  |  | PS-matched | 4,060 | <5 (0%) | 5 (0.2%) | 0.40 (0.08;2.06) | 0.273 | 2 |
|  | Duloxetine discontinue | Unadjusted | 4,603 | <5 (0%) | 5 (0.2%) | 0.46 (0.09;2.37) | 0.354 |  |
|  |  | PS-matched | 3,628 | <5 (0%) | <5 (0%) | 0.33 (0.03;3.20) | 0.341 | 2 |
| Including BMI as covariate |  |  |  |  |  |  |  |  |
|  | Duloxetine non-exposed | PS-matched | 6,880 | <5 (0%) | 15 (0.3%) | 0.53 (0.12;2.33) | 0.404 | 3 |
|  | SSRI exposed | PS-matched | 4,129 | <5 (0%) | <5 (0%) | 1.00 (0.18;5.46) | 1 | 3 |
|  | Venlafaxine exposed | PS-matched | 2,741 | <5 (0%) | <5 (0%) | 0.50 (0.09;2.73) | 0.423 | 3 |
|  | Duloxetine discontinue | PS-matched | 2,729 | <5 (0%) | <5 (0%) | 1.00 (0.14;7.10) | 1 | 3 |

PS-matched: propensity score matched analyses based on conditional logistic regression.
OR: odds ratio for major malformations in oro-facial clefts for duloxetine vs comparators. CI: Wald 95% confidence intervals. SSRI: Selective serotonin reuptake inhibitors.

Note2: Propensity score based on data source (Sweden/Denmark), age (grouped), education (grouped), household income, year (grouped), psychiatric hospital, psychiatric outpatient, smoking, previous spontaneous abortion, previous stillbirths, gestational diabetes during index pregnancy, diabetes, hyper- or hypothyroidism, hypertension, obesity, renal failure, depression, affective, anxiety or phobia, severe stress reaction, diabetic peripheral neuropathic pain, glucose-lowering, antihypertensive, fluconazole, thyroid, NSAID, opioids, triptans, antiepileptics, antipsychotics, anxiolytics, corticosteroid (combination).

Note3: Propensity score based on data source (Sweden/Denmark), age (grouped), education (grouped), household income, year (grouped), psychiatric hospital, psychiatric outpatient, smoking, previous spontaneous abortion, previous stillbirths, gestational diabetes during index pregnancy, diabetes, hyper- or hypothyroidism, hypertension, obesity, renal failure, depression, affective, anxiety or phobia, severe stress reaction, diabetic peripheral neuropathic pain, glucose-lowering, antihypertensive, fluconazole, thyroid, NSAID, opioids, triptans, antiepileptics, antipsychotics, anxiolytics, corticosteroid (combination), BMI (grouped).

# Table T Malformation subtype: Respiratory

| *Analysis* | *Comparator* | *Model* | *Total N* | *Duloxetine events (%)* | *Comparator events (%)* | *OR (95% CI)* | *P-value* | *Note* |
| --- | --- | --- | --- | --- | --- | --- | --- | --- |
| Main analyses (one prescription) |  |  |  |  |  |  |  |  |
|  | Duloxetine non-exposed | Unadjusted | 2,076,164 | <5 (0%) | 2,720 (0.1%) | 1.52 (0.49;4.71) | 0.467 |  |
|  |  | PS-matched | 7,189 | <5 (0%) | 13 (0.2%) | 0.92 (0.26;3.24) | 0.901 | 2 |
|  | SSRI exposed | Unadjusted | 41,471 | <5 (0%) | 64 (0.2%) | 1.24 (0.39;3.95) | 0.716 |  |
|  |  | PS-matched | 4,311 | <5 (0%) | <5 (0%) | 3.00 (0.50;17.95) | 0.229 | 2 |
|  | Venlafaxine exposed | Unadjusted | 6,752 | <5 (0%) | 8 (0.2%) | 1.30 (0.34;4.91) | 0.698 |  |
|  |  | PS-matched | 2,858 | <5 (0%) | <5 (0%) | 1.00 (0.20;4.95) | 1 | 2 |
|  | Duloxetine discontinue | Unadjusted | 4,388 | <5 (0%) | 5 (0.2%) | 1.14 (0.27;4.78) | 0.856 |  |
|  |  | PS-matched | 2,869 | <5 (0%) | <5 (0%) | 1.50 (0.25;8.98) | 0.657 | 2 |
| Overlap of exposure period and days duration |  |  |  |  |  |  |  |  |
|  | Duloxetine non-exposed | Unadjusted | 2,076,762 | <5 (0%) | 2,720 (0.1%) | 1.43 (0.54;3.81) | 0.474 |  |
|  |  | PS-matched | 10,159 | <5 (0%) | 17 (0.2%) | 0.94 (0.32;2.80) | 0.913 | 2 |
|  | SSRI exposed | Unadjusted | 64,906 | <5 (0%) | 94 (0.1%) | 1.25 (0.46;3.40) | 0.663 |  |
|  |  | PS-matched | 6,092 | <5 (0%) | 6 (0.1%) | 1.33 (0.38;4.72) | 0.656 | 2 |
|  | Venlafaxine exposed | Unadjusted | 9,387 | <5 (0%) | 13 (0.2%) | 1.04 (0.34;3.20) | 0.941 |  |
|  |  | PS-matched | 4,060 | <5 (0%) | <5 (0%) | 2.00 (0.37;10.92) | 0.423 | 2 |
|  | Duloxetine discontinue | Unadjusted | 4,603 | <5 (0%) | <5 (0%) | 1.15 (0.29;4.61) | 0.841 |  |
|  |  | PS-matched | 3,628 | <5 (0%) | <5 (0%) | 1.00 (0.20;4.95) | 1 | 2 |
| Including BMI as covariate |  |  |  |  |  |  |  |  |
|  | Duloxetine non-exposed | PS-matched | 6,880 | <5 (0%) | 12 (0.2%) | 1.00 (0.28;3.54) | 1 | 3 |
|  | SSRI exposed | PS-matched | 4,129 | <5 (0%) | 7 (0.3%) | 0.86 (0.22;3.31) | 0.823 | 3 |
|  | Venlafaxine exposed | PS-matched | 2,741 | <5 (0%) | <5 (0%) | 1.50 (0.25;8.98) | 0.657 | 3 |
|  | Duloxetine discontinue | PS-matched | 2,729 | <5 (0%) | <5 (0%) | 3.00 (0.31;28.84) | 0.341 | 3 |

PS-matched: propensity score matched analyses based on conditional logistic regression.

OR: odds ratio for major malformations in the respiratory system for duloxetine vs comparators. CI: Wald 95% confidence intervals. SSRI: Selective serotonin reuptake inhibitors.

Note2: Propensity score based on data source (Sweden/Denmark), age (grouped), education (grouped), household income, year (grouped), psychiatric hospital, psychiatric outpatient, smoking, previous spontaneous abortion, previous stillbirths, gestational diabetes during index pregnancy, diabetes, hyper- or hypothyroidism, hypertension, obesity, renal failure, depression, affective, anxiety or phobia, severe stress reaction, diabetic peripheral neuropathic pain, glucose-lowering, antihypertensive, fluconazole, thyroid, NSAID, opioids, triptans, antiepileptics, antipsychotics, anxiolytics, corticosteroid (combination).

Note3: Propensity score based on data source (Sweden/Denmark), age (grouped), education (grouped), household income, year (grouped), psychiatric hospital, psychiatric outpatient, smoking, previous spontaneous abortion, previous stillbirths, gestational diabetes during index pregnancy, diabetes, hyper- or hypothyroidism, hypertension, obesity, renal failure, depression, affective, anxiety or phobia, severe stress reaction, diabetic peripheral neuropathic pain, glucose-lowering, antihypertensive, fluconazole, thyroid, NSAID, opioids, triptans, antiepileptics, antipsychotics, anxiolytics, corticosteroid (combination), BMI (grouped).

# Table U Malformation subtype: Urinary tract

| *Analysis* | *Comparator* | *Model* | *Total N* | *Duloxetine events (%)* | *Comparator events (%)* | *OR (95% CI)* | *P-value* | *Note* |
| --- | --- | --- | --- | --- | --- | --- | --- | --- |
| Main analyses (one prescription) |  |  |  |  |  |  |  |  |
|  | Duloxetine non-exposed | Unadjusted | 2,076,164 | 8 (0.5%) | 7,471 (0.4%) | 1.48 (0.74;2.96) | 0.271 |  |
|  |  | PS-matched | 7,189 | 8 (0.6%) | 27 (0.5%) | 1.19 (0.54;2.63) | 0.671 | 2 |
|  | SSRI exposed | Unadjusted | 41,471 | 8 (0.5%) | 148 (0.4%) | 1.43 (0.70;2.92) | 0.325 |  |
|  |  | PS-matched | 4,311 | 8 (0.6%) | 12 (0.4%) | 1.33 (0.55;3.26) | 0.529 | 2 |
|  | Venlafaxine exposed | Unadjusted | 6,752 | 8 (0.5%) | 15 (0.3%) | 1.85 (0.78;4.38) | 0.160 |  |
|  |  | PS-matched | 2,858 | 8 (0.6%) | <5 (0%) | 2.00 (0.60;6.64) | 0.258 | 2 |
|  | Duloxetine discontinue | Unadjusted | 4,388 | 8 (0.5%) | 10 (0.3%) | 1.52 (0.60;3.87) | 0.375 |  |
|  |  | PS-matched | 2,869 | 8 (0.6%) | 6 (0.4%) | 1.33 (0.46;3.84) | 0.594 | 2 |
| Overlap of exposure period and days duration |  |  |  |  |  |  |  |  |
|  | Duloxetine non-exposed | Unadjusted | 2,076,762 | 11 (0.5%) | 7,471 (0.4%) | 1.43 (0.79;2.59) | 0.233 |  |
|  |  | PS-matched | 10,159 | 11 (0.5%) | 31 (0.4%) | 1.42 (0.71;2.82) | 0.318 | 2 |
|  | SSRI exposed | Unadjusted | 64,906 | 11 (0.5%) | 227 (0.4%) | 1.42 (0.78;2.61) | 0.253 |  |
|  |  | PS-matched | 6,092 | 11 (0.5%) | 10 (0.2%) | 2.20 (0.93;5.18) | 0.071 | 2 |
|  | Venlafaxine exposed | Unadjusted | 9,387 | 11 (0.5%) | 24 (0.3%) | 1.56 (0.76;3.18) | 0.226 |  |
|  |  | PS-matched | 4,060 | 11 (0.5%) | <5 (0%) | 5.50 (1.22;24.81) | 0.027 | 2 |
|  | Duloxetine discontinue | Unadjusted | 4,603 | 11 (0.5%) | 8 (0.3%) | 1.59 (0.64;3.95) | 0.321 |  |
|  |  | PS-matched | 3,628 | 10 (0.6%) | 6 (0.3%) | 1.67 (0.61;4.59) | 0.323 | 2 |
| Including BMI as covariate |  |  |  |  |  |  |  |  |
|  | Duloxetine non-exposed | PS-matched | 6,880 | 7 (0.5%) | 16 (0.3%) | 1.90 (0.76;4.73) | 0.168 | 3 |
|  | SSRI exposed | PS-matched | 4,129 | 7 (0.5%) | 10 (0.4%) | 1.40 (0.53;3.68) | 0.495 | 3 |
|  | Venlafaxine exposed | PS-matched | 2,741 | 6 (0.4%) | 5 (0.4%) | 1.20 (0.37;3.93) | 0.763 | 3 |
|  | Duloxetine discontinue | PS-matched | 2,729 | 7 (0.5%) | <5 (0%) | 1.75 (0.51;5.98) | 0.372 | 3 |

PS-matched: propensity score matched analyses based on conditional logistic regression.

OR: odds ratio for major malformations in the urinary tract for duloxetine vs comparators. CI: Wald 95% confidence intervals. SSRI: Selective serotonin reuptake inhibitors.

Note2: Propensity score based on data source (Sweden/Denmark), age (grouped), education (grouped), household income, year (grouped), psychiatric hospital, psychiatric outpatient, smoking, previous spontaneous abortion, previous stillbirths, gestational diabetes during index pregnancy, diabetes, hyper- or hypothyroidism, hypertension, obesity, renal failure, depression, affective, anxiety or phobia, severe stress reaction, diabetic peripheral neuropathic pain, glucose-lowering, antihypertensive, fluconazole, thyroid, NSAID, opioids, triptans, antiepileptics, antipsychotics, anxiolytics, corticosteroid (combination).

Note3: Propensity score based on data source (Sweden/Denmark), age (grouped), education (grouped), household income, year (grouped), psychiatric hospital, psychiatric outpatient, smoking, previous spontaneous abortion, previous stillbirths, gestational diabetes during index pregnancy, diabetes, hyper- or hypothyroidism, hypertension, obesity, renal failure, depression, affective, anxiety or phobia, severe stress reaction, diabetic peripheral neuropathic pain, glucose-lowering, antihypertensive, fluconazole, thyroid, NSAID, opioids, triptans, antiepileptics, antipsychotics, anxiolytics, corticosteroid (combination), BMI (grouped).

# Table V Malformation subtype: Other anomalies/syndromes

| *Analysis* | *Comparator* | *Model* | *Total N* | *Duloxetine events (%)* | *Comparator events (%)* | *OR (95% CI)* | *P-value* | *Note* |
| --- | --- | --- | --- | --- | --- | --- | --- | --- |
| Main analyses (one prescription) |  |  |  |  |  |  |  |  |
|  | Duloxetine non-exposed | Unadjusted | 2,076,164 | 14 (0.9%) | 9,983 (0.5%) | 1.93 (1.14;3.27) | 0.014 |  |
|  |  | PS-matched | 7,189 | 14 (1.0%) | 31 (0.5%) | 1.81 (0.96;3.40) | 0.066 | 2 |
|  | SSRI exposed | Unadjusted | 41,471 | 14 (0.9%) | 234 (0.6%) | 1.59 (0.92;2.73) | 0.095 |  |
|  |  | PS-matched | 4,311 | 14 (1.0%) | 12 (0.4%) | 2.43 (1.10;5.38) | 0.028 | 2 |
|  | Venlafaxine exposed | Unadjusted | 6,752 | 14 (0.9%) | 22 (0.4%) | 2.22 (1.13;4.34) | 0.020 |  |
|  |  | PS-matched | 2,858 | 14 (1.0%) | 6 (0.4%) | 2.33 (0.90;6.07) | 0.082 | 2 |
|  | Duloxetine discontinue | Unadjusted | 4,388 | 14 (0.9%) | 13 (0.5%) | 2.06 (0.96;4.39) | 0.062 |  |
|  |  | PS-matched | 2,869 | 14 (1.0%) | 8 (0.6%) | 1.75 (0.73;4.17) | 0.207 | 2 |
| Overlap of exposure period and days duration |  |  |  |  |  |  |  |  |
|  | Duloxetine non-exposed | Unadjusted | 2,076,762 | 19 (0.9%) | 9,983 (0.5%) | 1.85 (1.18;2.91) | 0.007 |  |
|  |  | PS-matched | 10,159 | 19 (0.9%) | 43 (0.5%) | 1.77 (1.03;3.03) | 0.039 | 2 |
|  | SSRI exposed | Unadjusted | 64,906 | 19 (0.9%) | 349 (0.6%) | 1.60 (1.01;2.55) | 0.046 |  |
|  |  | PS-matched | 6,092 | 19 (0.9%) | 19 (0.5%) | 2.11 (1.11;4.02) | 0.023 | 2 |
|  | Venlafaxine exposed | Unadjusted | 9,387 | 19 (0.9%) | 29 (0.4%) | 2.23 (1.25;3.99) | 0.007 |  |
|  |  | PS-matched | 4,060 | 19 (0.9%) | 8 (0.4%) | 2.38 (1.04;5.43) | 0.040 | 2 |
|  | Duloxetine discontinue | Unadjusted | 4,603 | 19 (0.9%) | 8 (0.3%) | 2.75 (1.20;6.30) | 0.017 |  |
|  |  | PS-matched | 3,628 | 16 (0.9%) | 5 (0.3%) | 3.20 (1.17;8.73) | 0.023 | 2 |
| Including BMI as covariate |  |  |  |  |  |  |  |  |
|  | Duloxetine non-exposed | PS-matched | 6,880 | 14 (1.0%) | 30 (0.5%) | 1.87 (0.99;3.52) | 0.054 | 3 |
|  | SSRI exposed | PS-matched | 4,129 | 14 (1.0%) | 12 (0.4%) | 2.33 (1.08;5.04) | 0.031 | 3 |
|  | Venlafaxine exposed | PS-matched | 2,741 | 14 (1.0%) | <5 (0%) | 7.00 (1.59;30.80) | 0.010 | 3 |
|  | Duloxetine discontinue | PS-matched | 2,729 | 14 (1.0%) | 5 (0.4%) | 2.80 (1.01;7.77) | 0.048 | 3 |

PS-matched: propensity score matched analyses based on conditional logistic regression.

OR: odds ratio for major malformations of other anomalies/syndromes for duloxetine vs comparators. CI: Wald 95% confidence intervals. SSRI: Selective serotonin reuptake inhibitors.

Note2: Propensity score based on data source (Sweden/Denmark), age (grouped), education (grouped), household income, year (grouped), psychiatric hospital, psychiatric outpatient, smoking, previous spontaneous abortion, previous stillbirths, gestational diabetes during index pregnancy, diabetes, hyper- or hypothyroidism, hypertension, obesity, renal failure, depression, affective, anxiety or phobia, severe stress reaction, diabetic peripheral neuropathic pain, glucose-lowering, antihypertensive, fluconazole, thyroid, NSAID, opioids, triptans, antiepileptics, antipsychotics, anxiolytics, corticosteroid (combination).

Note3: Propensity score based on data source (Sweden/Denmark), age (grouped), education (grouped), household income, year (grouped), psychiatric hospital, psychiatric outpatient, smoking, previous spontaneous abortion, previous stillbirths, gestational diabetes during index pregnancy, diabetes, hyper- or hypothyroidism, hypertension, obesity, renal failure, depression, affective, anxiety or phobia, severe stress reaction, diabetic peripheral neuropathic pain, glucose-lowering, antihypertensive, fluconazole, thyroid, NSAID, opioids, triptans, antiepileptics, antipsychotics, anxiolytics, corticosteroid (combination), BMI (grouped).

# Table W Stillbirth, sensitivity analyses

| *Analysis* | *Comparator* | *Model* | *Total N* | *Duloxetine events (%)* | *Comparator events (%)* | *OR (95% CI)* | *P-value* | *note* |
| --- | --- | --- | --- | --- | --- | --- | --- | --- |
| Overlap of exposure period and days duration |  |  |  |  |  |  |  |  |
|  | Duloxetine non-exposed | Unadjusted | 2,132,699 | 6 (0.3%) | 7,694 (0.4%) | 0.74 (0.33;1.66) | 0.471 |  |
|  |  | PS-matched | 10,572 | 6 (0.3%) | 36 (0.4%) | 0.66 (0.28;1.58) | 0.352 | 1 |
|  | SSRI exposed | Unadjusted | 73,069 | 6 (0.3%) | 312 (0.4%) | 0.61 (0.27;1.37) | 0.231 |  |
|  |  | PS-matched | 6,349 | 6 (0.3%) | 19 (0.4%) | 0.63 (0.25;1.58) | 0.326 | 1 |
|  | Venlafaxine exposed | Unadjusted | 9,884 | 6 (0.3%) | 51 (0.7%) | 0.40 (0.17;0.94) | 0.035 |  |
|  |  | PS-matched | 4,210 | 6 (0.3%) | 10 (0.5%) | 0.60 (0.22;1.65) | 0.323 | 1 |
|  | Duloxetine discontinue | Unadjusted | 4,701 | 6 (0.3%) | 10 (0.4%) | 0.66 (0.24;1.83) | 0.429 |  |
|  |  | PS-matched | 3,753 | 5 (0.3%) | 10 (0.5%) | 0.50 (0.17;1.46) | 0.206 | 1 |
| Including BMI as covariate |  |  |  |  |  |  |  |  |
|  | Duloxetine non-exposed | PS-matched | 7,576 | <5 (0%) | 27 (0.4%) | 0.64 (0.22;1.84) | 0.407 | 2 |
|  | SSRI exposed | PS-matched | 4,538 | <5 (0%) | 17 (0.6%) | 0.47 (0.16;1.40) | 0.175 | 2 |
|  | Venlafaxine exposed | PS-matched | 3,008 | <5 (0%) | 9 (0.6%) | 0.44 (0.14;1.44) | 0.177 | 2 |
|  | Duloxetine discontinue | PS-matched | 2,990 | <5 (0%) | <5 (0%) | 1.00 (0.25;4.00) | 1 | 2 |

OR: odds ratio for stillbirth for duloxetine vs comparator. PS-matched: propensity score matched analyses based on conditional logistic regression. CI: Wald 95% confidence intervals. SSRI: Selective serotonin reuptake inhibitors.

Note 1: Propensity score based on data source (Sweden/Denmark), age (grouped), education, household income, year (grouped), psychiatric hospital (grouped), psychiatric outpatient visits (grouped), smoking, previous spontaneous abortions, previous stillbirth, gestational diabetes during index pregnancy, diabetes, hyper- or hypothyroidism, hypertension, obesity, renal failure, depression, affective disorder, anxiety or phobia, severe stress reaction, diabetic peripheral neuropathic pain, glucose-lowering, antihypertensive, fluconazole, thyroid, NSAID, opioids, triptans, antiepileptics, antipsychotics, anxiolytics, corticosteroid (combination), antithyroid (combination).

Note 2: Propensity score based on data source (Sweden/Denmark), age (grouped), education, household income, year (grouped), psychiatric hospital (grouped), psychiatric outpatient visits (grouped), smoking, previous spontaneous abortions, previous stillbirth, gestational diabetes during index pregnancy, diabetes, hyper- or hypothyroidism, hypertension, obesity, renal failure, depression, affective disorder, anxiety or phobia, severe stress reaction, diabetic peripheral neuropathic pain, glucose-lowering, antihypertensive, fluconazole, thyroid, NSAID, opioids, triptans, antiepileptics, antipsychotics, anxiolytics, corticosteroid (combination), antithyroid (combination), BMI (grouped).
