# Supplementary material for: Exposure to duloxetine during pregnancy and risk of congenital malformations and stillbirth: A nationwide cohort study in Denmark and Sweden
Source: PLoS Med. 2021 Nov 22;18(11):e1003851. doi: 10.1371/journal.pmed.1003851 (PMC8654175; doi:10.1371/journal.pmed.1003851)
Supplement: Attachment — Submitted filename: Ankarfeldt_Duloxetine_RebuttalLetter.docx [file pmed.1003851.s004.docx]

**Rebuttal letter**

Dear editor,

Below are the reviewer comments marked with bold. Our reply is written below and is not bold. Text from the revised manuscript is marked with italic, and added text is marked with underline.

Major changes are:

In the previous version, the supplementary material held a lot of figures with results. These have all been condensed to tables. Also, the revised version includes all sensitivity analyses as requested.

For the stillbirth analyses, one reviewer identified an inconsistency with regard to the exclusion criteria of gestational age 20 and stillbirth being defined as after week 22. The exclusion criteria should have been 22 weeks, as also specified in the protocol. We apologize for the mistake and thank the reviewer for being observant. The manuscript (both the description in the Methods sections and the results) has been updated accordingly.

We are thankful for the reviewer comments, and the revision has resulted in an improved manuscript.

On behalf of the coauthors,

Mikkel Z Ankafeldt

**Requests from the editors: No comments from the editors**

**Comments from the reviewers:**

**Reviewer #1: "Exposure to duloxetine during pregnancy and risk of congenital malformations and stillbirth A nation-wide cohort study in Denmark and Sweden" seeks to investigate the possible effect of Duloxetine (prescribed as an antidepression etc.) exposure, on (major/minor) congenital infant malformation and stillbirth. The study was conducted on a cohort of all pregnancies in Sweden and Denmark, from 2004-2016. The major finding was that duloxetine exposure did not result in increased risk of malformation/stillbirth, from adjusted and propensity score-matched (cohort details in Table S6A/B) logistic regression analyses.**

**Concerns may remain over the relatively low number of Duloxetine events (60+ for major/minor malformation, 5 stillbirths), which would seem to make drawing definitive conclusions difficult. However, given the national-level scope of the study and lack of prior large cohort studies, this would appear to be a valuable addition to the literature. Nevertheless, some issues might be addressed:**

**1. For the cohort selection flowchart from Figure 1, there appears to possibly be an inconsistency. It is presented that both malformation & stillbirth cohorts begin with the same initial cohort (N=2,193,732), and that the malformation cohort has N=1,042 subjects who "redeemed prescription of duloxetine 3 months prior to LMP and not during exposure period". However, given that the only prior exclusion criteria for the stillbirth cohort was "mother migrated 3 months prior to LMP until delivery", and that this appears a strict subset of "mother migrated 12 months prior to LMP until 12 months past delivery" for the malformation cohort (amongst other exclusion criteria for the malformation cohort), it would appear that the corresponding number of subjects excluded for "redeemed prescription of duloxetine 3 months prior to LMP and not during exposure period" for the stillbirth cohort should be not less than that for the malformation cohort (whereas it is presented as N=975). The authors might clarify if this reasoning is correct or flawed.**

We have checked data once more and we have found no mistake.

The explanation is that the exclusion criterium is *redeemed prescription of duloxetine 3 months prior to LMP and not during exposure period*. This translates differently in the malformation and the stillbirth cohort, as there are different exposure time windows. The exposure time window for malformations was from LMP to 90 days after LMP (corresponding to the first trimester of the pregnancy). The exposure time window for stillbirths was from LMP to end of pregnancy. Hence, the exposure time window in the malformation cohort is shorter than the exposure time window in the stillbirth cohort. The exclusion criterium therefore counts less in the stillbirth cohort, and fewer are excluded in this step.

The figure legend now defines the exposure time window. The flowchart has also been updated with the exclusion criterium of gestational age less than 22 weeks, as the reviewer pointed out in later comments. Finally, for transparency, the number of exposed in each group among the final cohort is added.

**2. On Page 8, it is mentioned that "When fitting each model, covariates were removed, if the model could not be estimated" for adjusted/PS models. The authors might consider explaining in greater detail why certain covariates would cause a model not to be able to be estimated, and what models were affected/covariates were removed for these affected models. Details on excluded covariates might be added to Supplementary Figures S1 to S12 as appropriate.**

The coauthors have already discussed internally how to best describe this. We suggest that an example will help explain (added text is underlined):

*When fitting each model, covariates were removed, if the model could not be estimated. This is the case if e.g. no patients in one exposure group had any severe stress reaction. Then the variable severe stress reaction cannot be part of the model and the covariate needs to be removed.*

Covariates used in the primary analyses are found in supplementary table S8. Analyses in the supplementary material (tables S9-S23) include information about covariates. If the editor agrees, we suggest to describe all covariates available (in the Methods section) and describe covariates included in each model (in the result section). We find it redundant to also describe covariates not included in each model.

**3. On Page 9, it is mentioned that missing income would be imputed from one year prior and then one year after LMP if possible, and 1 year prior for education. Does this cover all missing data cases, and if not, what was the final imputed value for cases where no income/education/etc. data was available at all?**

The reviewer read the imputation of income and education correctly and imputation did not identify a value for all missing data cases. If no value was imputed, we kept it missing, and data were analyzed assuming missing at random, meaning that persons with missing values were deleted from the analysis. We have tried to make this clearer by moving the statement of assuming missing at random after the description of imputation.

The updated text is:

*In general, there were very few missing values. If income was missing at year of LMP, first income was imputed from one year prior to LMP, and if still missing, income was imputed from one year after LMP, where possible. If education was missing at year of LMP, it was imputed from one year after LMP, where possible. Data were analyzed assuming missing at random and persons with missing values were deleted from the analysis.*

**4. On Page 9, it is noted that four sensitivity analyses were performed. However, their charts/details were not located, despite patterns on the results of these four sensitivity analyses being referenced in the text discussion.**

Apologies for not including all sensitivity analyses. The supplementary material has been updated and tables S9-S23 include all sensitivity analyses.

**In particular, for BMI, the main analyses in Figures 2 to 4 do not appear to mention BMI in their marker descriptions, while subanalyses from Figure S2 onwards mention BMI (grouped) in marker 3, which however does not appear to have been applied (also covered as a minor issue). The authors might consider including the details of these sensitivity analyses.**

It was a mistake that BMI appeared as covariates in primary analyses (in former figures S2-S12). The details on subanalyses and sensitivity analyses are found in the revised supplementary material in tables S9-S23.

**5. On Page 14, it is stated that "...adjusted analyses could not consistently be performed due to the low number of outcome events despite of the cohort including more than 2 million births. The PS matched analyses performed better with few outcome events."; it might be clarified as to what "better" in the second sentence means - better in some statistical sense, or better by the results?**

We agree that the sentence is not clear. The intention was to write that propensity analyses are possible even with few outcome events. The revised manuscript is (added text is underlined):

*In the analyses of major malformation subtypes and stillbirth, adjusted analyses could not be consistently performed due to the low number of outcome events despite the cohort including more than 2 million births. As opposed to adjusted analyses, the PS-matched analyses were possible despite few outcome events.*

**Minor issues:**

**6. On Page 8, the relevant literature contributing to covariate selection might be referenced if possible.**

We have updated the text including references used to identify covariates (added text is underlined):

*For the adjusted multiple regression and the PS, prespecified covariates were used based on previous publications^21,23^ and available data.*

**7. The motivation for using SSRI-exposed and Venlafaxine exposed patients as comparators might be moved from the discussion (Page 13), to the initial description of the methodology.**

The Methods section has been rewritten (underlined text is added):

*Four comparison groups were used, all with no redeemed prescription of duloxetine in the relevant exposure time window: 1) duloxetine non-exposed: no redeemed prescription of duloxetine in the exposure time window, 2) SSRI exposed: at least one redeemed prescription of an SSRI (ATC N06AB) in the exposure time window, 3) venlafaxine exposed: at least one redeemed prescription of venlafaxine (ATC N06AX16) in the exposure time window, and 4) duloxetine discontinuers: at least one redeemed prescription of duloxetine between 365 days prior to LMP to LMP and not during pregnancy.* *SSRI exposed and venlafaxine exposed women and duloxetine discontinuers were used as comparators to take confounding by indication and severity, and maybe even unmeasured confounding, into account, as they are expected to be similar to duloxetine exposed women with regard to e.g. the underlying psychological disease, comorbidity, and health behavior.*

**8. From Figure 1, it might be clarified whether the N=73 stillbirths (abortions after week 22) were spontaneous or intentional.**

The flowchart (Figure 1) is updated. In the previous version, the box stated that the additional 73 stillbirths were identified in the medical birth register. That is not correct. The stillbirths were identified in the patient register as abortions. This is written in the updated flowchart (Figure 1). Also, the updated figure legend specifies that these were all spontaneous.

The Methods section has also been updated regarding the data sources to identify stillbirths (added text is underlined):

*Information on stillbirth was gathered from the medical birth registers and was defined as no signs of life at birth after week 22 of pregnancy, and from the patient register if abortions were registered after week 22.*

**9. Still on the malformation subtype analyses as presented in Supplementary Figures S1 to S12, certain values are presented as <5 (e.g. in S3, S5, S6, etc.); Might the exact number of events be presented & analyzed instead?**

Due to Danish privacy regulations, subgroups with less than 5 are not presented. To make this clear, this is added to table legends and figure legends. Also, we have added the following text to the Methods section (added text is underlined):

*Due to Danish privacy regulations, subgroups with less than 5 are not presented. Less than 5 can include 0. All analyses are, however, performed with exact numbers.*

**10. For Supplementary Figure S6, unconventional OR (i.e. 416E3, 174E3) are provided, which might indicate a technical issue (e.g. division by zero) in computation of the values. This might be addressed.**

We apologize for this error. The details from the figures in the previous version of the supplementary material are now included as tables in the updated supplementary material. Subtype malformation analyses and sensitivity analyses can be found in the supplementary material tables S9-S23.

**11. For Supplementary Figures S2 to S12, while details for Markers 1, 2 and 3 are provided for each figure, it appears that only "1,2" is ever labelled for the PS-matched row for each comparator. If so, the authors might consider simply describing the propensity score criteria for PS-matched without requiring a Marker column.**

The details from the figures in the previous version of the supplementary material are now included as tables in the updated supplementary material. Subtype malformation analyses and sensitivity analyses are found in the supplementary material in tables S9-S23. These tables include information about covariates used in each PS score.

**12. There may be some** **grammatical/spelling issues, e.g. "the reproductive age"; "data collected form registers", "statistical significant findings" in the Abstract, "this tendency were reduced" (Page 10), etc. These might be addressed.**

We apologize. The manuscript has been proofread before resubmission.

**Reviewer #2: This is a study from Sweden and Denmark aiming to explore whether use of duloxetine in pregnancy could increase the risk of major and minor congenital malformation, and the risk of stillbirth. The authors linked data from nationwide registers from Sweden and Denmark covering all registered births from 2004-2016. Generally, this is an important research question although several previous studies have shown that use of duolexitine in pregnancy did not increase the risk of malformations and stillbirths, and thus the novelty of the study in the field is relatively limited. Also, some methodological aspects of the paper should be better addressed by the authors for a better interpretation of the findings. Here below are more specific comments on the various sections of the paper.**

**ABSTRACT:**

**in the "Exposure" paragraph you wrote "4) women discontinuing duloxetine treatment before pregnancy." Maybe you wanted to write during pregnancy?**

This comparison group consists of women who were exposed to duloxetine prior to pregnancy but discontinued before pregnancy start. The manuscript has been updated (added text is underlined):

*4) women exposed to duloxetine prior to, but not during pregnancy*

**INTRODUCTION:**

**You should review the English especially in the second paragraph.**

We apologize. The manuscript has been proofread before resubmission.

**METHODS:**

**In the "Cohorts" paragraph you did not specify inclusion/esclusion criteria on age at delivery and gestational age. I recommend to you to consider such inclusion/exclusion criterias: ages at delivery between 12-55 years and gestational age between 22-42/46 gestational weeks. This latter, especially, is very important following the stillbirth outcome that you chose to evaluate.**

Thank you for being observant. The reviewer is right that the cohort needs to be limited with regard to gestational age. Gestational week 22 is important, since end of pregnancy before week 22 will be considered as abortion, and therefore the stillbirth outcome is not possible before week 22, as the reviewer also points out. In our SAP and protocol we write that the cohort is limited to gestational week 22 and 45. We apologize for this mistake.

Regarding delivery age: The cohort is restricted to women >= 18 years and was so in the previous version. We have not used an upper age limit, but we can inform that in the malformation and stillbirth analyses 5 pregnancy analyses were by mothers with delivery age about 55 years. Maximum delivery age is 57 years.

The manuscript and results have been updated, but overall results did not change.

Flowchart Figure 1 is updated.

Figure 3 with stillbirth analyses is updated.

Methods section (added text is underlined):

*The cohort consisted of registered live and stillbirths from 2004 to 2016 of women with a valid personal identification number aged 18 and above.*

*[…]*

*Exclusion criteria for the stillbirth analyses were: mothers migrated between 90 days prior to LMP until delivery and gestational age shorter than 22 weeks or longer than 45 weeks.*

**"Exposure, comparison groups and outcome" paragraph:**

**(i) In the second paragraph I reccomad to specify the fourth comparison group as follow: "4) duloxetine discontinues: at least one redeemed prescription of duloxetine between 365 day priorLMP to LMP, BUT NOT DURING PREGNANCY, if it is the case.**

The text has been updated (added text is underlined):

*4) duloxetine discontinuers: at least one redeemed prescription of duloxetine between 365 days prior to LMP to LMP and not during pregnancy.*

**(ii) you stated that you excluded women with duloxetine exposure from 90 days prior to LMP, but no exposure from LMP to 90 days after LMP, why you made this choice if in the sensitivity analysis you evaluated the overlap days of supply?**

For the comparison groups of duloxetine non-exposed, SSRI and venlafaxine exposed, the exclusion criterium of “duloxetine exposure from 90 days prior to LMP” is applied. This is to avoid that the comparison groups had a prescription just before pregnancy and a possible ‘contamination’ of the comparison groups. This way, the comparison groups are as ‘clean’ as possible without duloxetine exposure. This is written in the manuscript (no changes): *This washout period was applied to avoid misclassification.*

The reviewer asks if this coincides with the sensitivity analyses evaluating the overlap of days’ supply. This is not the case. Patients excluded from comparison groups in the primary analyses (at least 1 prescription during pregnancy) due to duloxetine prescription just before pregnancy may be included in the sensitivity analyses as duloxetine exposed, if there is an overlap of days’ supply with pregnancy.

**(iii) in the last sentence you define the stilbirth as a no signs of life at birth after week 22 of pregnancy, once again it is very important that all women included in the cohort have at least 22 weeks of gestation.**

Again, we apologize for the error, and thank the reviewer for being observant. The manuscript and results have been updated.

**"Statistical methods":**

**(i) in the third paragraph you said that "when fitting each model, covariates were removed, if the model could not be estimated.". Since you consider rare outcomes I recommend you to did not performe multivariate analysis but adjusted for the propensity score. I sow that you delete a huge number of women after the PS matching, why did you not performe the propensity score stratification? I would delete the multivariate analysis.**

It is the nature of propensity score matched analyses to exclude those not matched. One could argue that this is the strength of propensity: that only patients that are similar/comparable (e.g. similar PS-scores) are compared. That is the reason PS matching was chosen. Importantly, all duloxetine exposed are kept after the PS matching. This is because a proper match is found among the given comparators. With more than two million births and up to 1,668 duloxetine exposed, not all comparators are included in a matched analyses.

We acknowledge that PS adjustment and PS stratification could be done, but we do not believe that the results will be changed.

We fully agree that the multivariate adjusted analyses are not ideal when analyzing rare outcome. This is also acknowledged in the Discussion section (added text is underlined):

*In the analyses of major malformation subtypes and stillbirth, adjusted analyses could not be consistently performed due to the low number of outcome events despite of the cohort including more than 2 million births. As opposed to adjusted analyses, the PS-matched analyses were possible despite few outcome events.*

There are strong traditions within both adjustment and PS (matching) and when writing the protocol, we decided to perform both kinds of analyses. There will be readers who would like either one or the other. The reviewer suggests deleting the multivariate analyses. If the editor agrees, we would like to report the study as it was designed in the protocol, and therefore we suggest keeping both PS matching and multivariate adjustment.

**(ii) you said that you assess the comorbidity in the 5 years prior to LMP, are you sure that all women have this follow-up prior to LMP? In case yes, you should add as inclusion/exclusion criteria.**

We cannot guarantee that all women are present in the Danish and Swedish patient registers five years prior to pregnancy. Exclusion criterium with regard to migration was 12 months prior to LMP for malformation analyses and 3 months prior to LMP for stillbirth analyses.

The comorbidity diagnoses are identified up to five years prior to LMP. Due to the universal health care system with free access for all citizens in both Denmark and Sweden, it is very likely that comorbidities are registered in the patient registers at some point five years prior to LMP.

The manuscript and legends for tables and figures have been updated to be more precise.

Methods section (added text is underlined):

*Comorbidities identified up to 5 years prior to LMP*

**(iii) when you present the four sensitivity analysis you performed, I would say that you performed them to evaluate the effect of potential misclassification of exposure.**

We agree that this is the case for the first two sensitivity analyses, and this is added to the Methods section presenting the sensitivity analyses (added text is underlined):

*To assess the exposure definition of minimum one redeemed prescription and evaluate potential misclassification of exposure, sensitivity analyses were conducted with (1) exposure redefined to minimum two redeemed prescriptions; and (2) exposure redefinition to overlap between the exposure time window and days’ supply of redeemed prescriptions.*

**(****iv) in the third sensitivity analysis you stated that you restricted the cohort to the first pregnancy within the study, why did you not use the generalized estimating equation to account for potential correlation within women with multiple pregnancies during the considered period in the unadjusted analyses? You should assess in the undjusted analysis if the estimate with and without the generalized estimating equation to account for potential correlation within women with multiple pregnancies during the considered period are different, In case not you can delete the third sensitivity analysis.**

What the reviewer suggest seems like a clever way to investigate correlation. In our study, we investigated the impact of potential correlation in sensitivity analyses limiting to first observed pregnancy. Since the conclusion of the study was not based on univariate analyses, and we did adjust prior outcomes in the adjusted analyses, we believe that the findings are still sound. If the editor agrees, we would like to report the analyses as prespecified in the protocol.

**(v) you performed the last sensitivity analysis in complete case sample with BMI information, can you consider the missing for that variable as missing at random?**

The reviewer has a good point. It may be that BMI is obtained for a reason, and therefore may not be missing at random. This is the reason why BMI is only used in sensitivity analyses and not included in the primary analyses. However, remember that the information on BMI is obtained from the medical birth registers with only 6.4% missing values. Had patient records been used to obtain information about BMI, missing would likely be more widespread and recorded non-random.

The revised manuscript has been updated (added text is underlined):

*The medical birth registers hold information on maternal BMI, but this is expected to be missing for a substantial number of women. The main analyses did not include BMI as a covariate, but (4) sensitivity analyses including BMI as covariate were conducted. The sensitivity analyses handled BMI as missing at random, although this may not be fully accurate.*

**RESULTS:**

**You shoal review the English and make all the paragraph more descriptive.**

The manuscript has been proofread before resubmission.

**TABLE and FIGURE:**

**(i) I would collapse the figure 2 and figure 3 in 1 Figure with Panel A and Panel B. You would have only the unadjusted and PS-matched (or I recommend to you to try the PS stratification) estimate for each comparison.**

Figure 2 and Figure 3 are now combined into one figure (Figure 2 with panel A and B).

As written above, we see the idea of using PS, especially with rare events. However, if the editor agrees, we suggest to report the study as described in the protocol and show unadjusted, adjusted and PS-matched results.

**(ii) I suggest to move the Table 2 in the supplementary materials**

We agree and have moved Table 2 to the supplementary materials. It is now Table S7.

**(iii) Table S6 I would take only the Post STD diff.**

As suggested by the reviewer, Table S6 (A and B) is updated and includes only post STD.

**Reviewer #3: This is a well-written revised manuscript that describes a study examining whether duloxetine exposure in pregnancy is associated with malformations or stillbirths. The study is well designed and controls for confounding by indication in several different ways which extremely important for this type of study. I have no suggestions for changes. Well done**

We thank the reviewer for the positive comment.
