# Supplementary material for: Exposure to duloxetine during pregnancy and risk of congenital malformations and stillbirth: A nationwide cohort study in Denmark and Sweden
Source: PLoS Med. 2021 Nov 22;18(11):e1003851. doi: 10.1371/journal.pmed.1003851 (PMC8654175; doi:10.1371/journal.pmed.1003851)
Supplement: Attachment — Submitted filename: Ankarfeldt_Duloxetine_rebuttal_letter.docx [file pmed.1003851.s005.docx]

Dear Editors,

Thank you for the opportunity to revise our manuscript. We have followed the Requests from the Editors, and answered point by points.

Below you will find the comments from the Editors and the reviewers marked with bolt font.

Our answer is written without bolt font, and cited text from the manuscript is marked with italic font, where added text is marked with italic, underlined font.

**Requests from the editors:**

**1. Reviewer 2 Comment: Please do address the remaining comments of Reviewer 2. The editors feel that it is acceptable to present both multivariable and propensity-score matched analyses. Please do address the Reviewer’s request regarding restricting the analysis to those with at least 1 year of prior registry data. If this is not feasible we request that you include a thorough discussion of the limitation related to identifying a confounder in those with 5 years of back follow up in registry vs those with fewer years available in registry.**

Thank you for suggesting that both the multivariable and the propensity-score matched analyses can be published. It will allow for the manuscript being aligned with the protocol.

We understand the reviewers concern regarding a five year vs. one year “back-follow-up” period. However, the study did not require that all women had at least five years “back-follow up” available to identify comorbidities. We looked for comorbidity in data up to five years prior LMP. This way we have included as many patients as possible.

We know that in e.g. U.S. claims data with multiple data sources, based on private insurances, researchers must ensure that the cohort is actually present in the data. This is not the case in Sweden and Denmark. There is a single data source in Sweden and a single data source in Denmark where patients’ diagnoses are captured, when they visit the universal, tax-funded health care system. Furthermore, there will be no difference in “back-follow-up” period between the exposure groups, and we therefore do not believe that it will be a source of confounding.

We have revised the manuscript to be more precise, and hope that the Editors agree, that sensitivity analyses restricting on various “back-follow-up” periods does not improve the interpretation of the results.

Copy of the revised text (changes are underlined):

Method Section - Regarding registers: *The patient registers [37,38] that include discharge diagnoses of all in- and outpatients in contact with a hospital.*

Method Section – Regarding covariates: *Comorbidities (identified up to maximum 5 years prior to LMP. See supplementary material for ICD-10 codes and ATC codes):*

**2. Data availability statement: For data that are freely or publicly available, please note this and provide more specific/direct links to access the Statistics Denmark and Statistics Sweden, and** **Swedish National Board of Health and Welfare data used in this study. For more information, please see the policy at**

**http://journals.plos.org/plosmedicine/s/data-availability**

**and FAQs at**

**http://journals.plos.org/plosmedicine/s/data-availability#loc-faqs-for-data-policy**

A Data availability statement is added to the manuscript with relevant links.

Copy of the revised text (changes are underlined):

***Data availability***
*Data from the Danish and Swedish registers are third party data, meaning that we as researchers do not hold the data, but have got data after application at relevant parties. The Danish data can be applied for at Statistics Denmark (*[*https://www.dst.dk/en/TilSalg/Forskningsservice*](https://www.dst.dk/en/TilSalg/Forskningsservice)*). The Swedish data can be applied for at Statistic Sweden (*[*https://www.scb.se/en/About-us/contact-us/*](https://www.scb.se/en/About-us/contact-us/)*) and Swedish National Board of Health and Welfare data (*[*https://www.socialstyrelsen.se/en/statistics-and-data/statistics/statistical-databases/*](https://www.socialstyrelsen.se/en/statistics-and-data/statistics/statistical-databases/)*).*

**3. Throughout text: Please include line numbers with the revised version.**

Line numbers are added

**4. Throughout text: Please use square brackets for in-text references, placed before the sentence punctuation. For multiple references within brackets, please do not include spaces.**

The in-text references are updated. Not marked as track changes.

**5. Abstract: Please structure your abstract using the PLOS Medicine headings (Background, Methods and Findings, Conclusions).**

**6. Abstract: Please fully define all abbreviations at first use in the text (SNRI, SSRI, etc).**

**7. Abstract: Methods and Findings: In this section, please clearly describe the study design, population and setting, number of participants, years during which the study took place, length of follow up, and main outcome measures. Please quantify the main results (with 95% CIs and p values). Please note the important dependent variables that are adjusted for in the analyses. In the last sentence of the Abstract Methods and Findings section, please describe the main limitation(s) of the study's methodology.**

**8. Abstract: Conclusion: We suggest revising to “...no increased risk of major or minor congenital malformations or stillbirth was associated with exposure to duloxetine during pregnancy.”**

Editors request 5-8 are implemented in the revised abstract.

Copy of the revised text (changes are underlined):

***Background****: The prevalence of depression and the exposure to antidepressants are high among women of reproductive age and during pregnancy. Duloxetine is a selective serotonin-norepinephrine reuptake inhibitor (SNRI) approved in the United States and Europe in 2004 for the treatment of depression. Fetal safety of duloxetine is not well established. The present study evaluates the association of exposure to duloxetine during pregnancy and the risk of major and minor congenital malformations and the risk of stillbirths.*

***Methods and Findings****: A population-based observational study based on data from registers in Sweden and Denmark. All registered births and stillbirths in the medical birth registers between 2004-2016 were included. Malformation diagnoses were identified up to one year after birth. Logistic regression analyses were used. Potential confounding was addressed through multiple regression, propensity score matching, and sensitivity analyses. Confounder variables included sociodemographic information (income, education, age, sex, year of birth and country), comorbidity and comedication, previous psychiatric contacts and birth related information (smoking during pregnancy and previous spontaneous abortions and stillbirths).*

*Duloxetine exposed women were compared with four comparators: 1) duloxetine non-exposed women, 2) Selective serotonin reuptake inhibitors (SSRI) exposed women, 3) venlafaxine exposed women, and 4) women exposed to duloxetine prior to, but not during pregnancy. Exposure was defined as redemption of a prescription during the first trimester and throughout pregnancy for the analyses of malformations and stillbirths, respectively. Outcomes were major and minor malformations and stillbirths gathered from the national patient registers. The cohorts consisted of more than 2 million births with 1,512 duloxetine exposed pregnancies. No increased risk for major malformations, minor malformations, or stillbirth was found across comparison groups in adjusted and propensity score matched analyses. Duloxetine exposed vs duloxetine non-exposed propensity score matched analyses showed for major malformations odds ratio (OR) 0.98 (95% confidence interval [CI] 0.74-1.30); for minor malformation OR 1.09 (95% CI 0.82-1.45); for stillbirths 1.18 (95% CI 0.43-3.19). For the individual malformation subtypes, some findings were statistically significant but were associated with large statistical uncertainty due to the extremely small number of events. The main limitations for the study were that the indication for duloxetine and a direct measurement of depression severity were not available single covariates.*

*Conclusion: Based on this observational register-based nationwide study with data from Sweden and Denmark, no increased risk of major or minor congenital malformations or stillbirth was associated with exposure to duloxetine during pregnancy.*

**9.** **Author summary: At this stage, we ask that you include a short, non-technical Author Summary of your research to make findings accessible to a wide audience that includes both scientists and non-scientists. The Author Summary should immediately follow the Abstract in your revised manuscript. This text is subject to editorial change and should be distinct from the scientific abstract. Please see our author guidelines for more information:** [**https://journals.plos.org/plosmedicine/s/revising-your-manuscript#loc-author-summary**](https://journals.plos.org/plosmedicine/s/revising-your-manuscript#loc-author-summary)

An author summary has been included.

Copy of the revised text (changes are underlined):

**Author summary**

***Why Was This Study Done?***

- ***A lot of women, in the fertile age take drugs used to treat depression***
- ***Duloxetine was approved in 2004 for the treatment of depression and its use has been increasing***
- ***There is a need for information on the possible association between exposure to duloxetine and malformation or stillbirth among offspring.***

***What Did the Researchers Do and Find?***

- *In a nationwide register based study all women with pregnancies ending in a live birth or stillbirth in Denmark and Sweden were analysed.*
- *The following information was gathered: drug exposure, comorbidities, education and income, as well as information on malformations and stillbirths.*
- *The cohort consisted of more than 2 million births identified.*
- *Based on several analyses, taking into account difference beside drug exposure consideration, the authors conclude that there is no association between exposure to duloxetine during pregnancy and the risk for malformations or stillbirth.*

***What Do These Findings Mean?***

- *This study with nationwide register data from Sweden and Denmark found no increased risk of congenital major or minor malformations or stillbirths among women exposed to duloxetine during pregnancy.*
- *The study analyzes the risk of malformations and stillbirths. Other safety outcomes (e.g. preterm birth or small for gestational age) were not addressed and need to be analyzed in future studies.*

**10. Introduction: The final paragraph of the section “The present study is based on a safety study…” seems more appropriate for the Methods section.**

We have moved the mentioned paragraph to be the first paragraph in the Methods section.

**11. Methods: Please provide more description of the Prescription, Patient, and Medical Birth registers used in the study.**

We have updated the manuscript with more information about the registers.

Copy of the revised text (changes are underlined):

*The following Danish and Swedish nationwide registers were used. The prescription registers [34–36], containing electronically submitted information on prescriptions dispensed by pharmacies, classified according to the global ATC system. The patient registers [37,38] that include discharge diagnoses of all in- and outpatients in contact with a hospital. The medical birth registers [39–41] were all livebirths as well as stillbirths from varying gestational ages in the different countries are notified to the registers with information on the mother, the neonate and the father as well. Registers holding information about education and household income [42–44] based on national statistics on education (highest obtained education) and annual tax reports.*

**12. Methods: Where the four non-exposed groups are described, it might be helpful to mention that venlafaxine is another SNRI.**

Good idea. We have added that.

Copy of the revised text (changes are underlined):

*3) venlafaxine exposed: at least one redeemed prescription of venlafaxine (ATC N06AX16. Venlafaxine is an SNRI like duloxetine) in the exposure time window*

**13. Methods: From Figure 3 and Table S8 it seems that adjusted analyses for the stillbirth outcome were not done. Please clarify this in the text, similar to: “For analyses of malformation subtypes with less than outcome events in the exposed group, only unadjusted and PS-matched analyses were performed.”**

Thank you. This clarified.

Copy of the revised text (changes are underlined):

*For the analyses of stillbirth and malformation subtypes with less than 30 outcome events in the exposed group, only unadjusted and PS-matched analyses were performed.*

**14. Methods: Did your study have a prospective protocol or analysis plan? Please state this (either way) early in the Methods section.**

**a) If a prospective analysis plan (from your funding proposal, IRB or other ethics committee submission, study protocol, or other planning document written before analyzing the data) was used in designing the study, please include the relevant prospectively written document with your revised manuscript as a Supporting Information file to be published alongside your study, and cite it in the Methods section. A legend for this file should be included at the end of your manuscript.**

**b) If no such document exists, please make sure that the Methods section transparently describes when analyses were planned, and when/why any data-driven changes to analyses took place.**

A prespecified protocol is publicly available via the ENCePP website. We suggest keeping the study registration, and protocol, at the ENCePP website, and not included it at the PLoS One website also, if the Editors agree.

This is mentioned in the first paragraph of the Methods section, and a link direct to the protocol is added as reference.

Copy of the revised text (changes are underlined):

*The present study is based on a safety study regarding duloxetine and pregnancy outcomes, with the protocol and the full study report available via the European Network of Centres for Pharmacoepidemiology and Pharmacovigilance (ENCePP, EUPAS20253) [34].*

**c) In either case, changes in the analysis-- including those made in response to peer review comments-- should be identified as such in the Methods section of the paper, with rationale.**

Deviation from the protocol is described in the revised manuscript.

Copy of the revised text (changes are underlined):

*The protocol was followed, with a few exceptions; 1) incomes should have been standardized to 2015-year level, but when grouped in quartiles, stratified on calendar year, standardization was not needed. 2) ATC codes were included to identify comorbidity, although not described in the protocol. Utilizing both ICD-10 and ATC codes more women with the given comorbidities will be identified. 3) According to the protocol, comedication should have been identified one-year prior LMP, but was changes to be during the relevant exposure time window, since is more likely that comedication during pregnancy will affect the outcomes of malformation and stillbirth, and therefore act as confounders.*

**15. Methods: Please ensure that the study is reported according to the STROBE guideline, and include the completed STROBE checklist as Supporting Information. Please add the following statement, or similar, to the Methods: "****This study is reported as per the Strengthening the Reporting of Observational Studies in Epidemiology (STROBE) guideline (S1 Checklist)."**

**The STROBE guideline can be found here: http://www.equator-network.org/reporting-guidelines/strobe/**

**When completing the checklist, please use section and paragraph numbers, rather than page numbers.**

The STROBE checklist is added as supporting information

**16. Results: Please move the baseline table for stillbirth analyses to the main text.**

We agree with this. Baseline table for stillbirth analyses is Table 2 in the revised manuscript. The table is inserted without track changes.

Also, to increase the readability of the manuscript we have included baseline values for both the full cohort and propensity score matched cohorts. Table 1 for the analyses of malformation and Table 2 for the analyses of stillbirth.

Copy of the revised text (changes are underlined):

*Table 1 and Table 2 show baseline characteristics for the analyses of malformation and stillbirth, respectively.*

**17. Results: For all analyses described, please present the complete main results together with 95% CIs and p values. For example, please present the results for this analysis, with 95% CIs and p values. “For minor malformations, the unadjusted analysis of duloxetine exposed compared with duloxetine nonexposed showed an increased risk. However, in the adjusted and PS-matched analyses the risk was lower and showed no statistically significant increase.”**

We understand that generally all analyses described should include point estimates, confidence intervals and p-value. However, in the present study, with several comparators, unadjusted, adjusted and PS-matched analyses, we believe that including all the data will decrease the readability. We suggest keeping the PS-matched results only and the references to figures and tables where all results are available. This is exemplified below.

We hope that the Editors agree with this. If the Editors think differently, please let us know.

The text referred to in the comment 17 above is taken out of context (“*For minor malformations, the unadjusted analysis of duloxetine exposed compared with duloxetine nonexposed showed an increased risk. However, in the adjusted and PS-matched analyses the risk was lower and showed no statistically significant increase*.”), the full paragraph should be read, where PS-matched analyses are mentioned, and there is a reference to a figure.

Copy of the revised text (changes are underlined):

*For minor malformations, the unadjusted analysis of duloxetine exposed compared with duloxetine non-exposed showed an increased risk. However, in the adjusted and PS-matched analyses the risk was lower and showed no statistically significant increase. When compared with SSRI exposed, venlafaxine exposed, and duloxetine discontinuers, some point estimates indicate an increased risk for minor malformations for duloxetine exposed, however, the wide confidence intervals suggest great uncertainty (Figure 2). The PS-matched analysis for duloxetine exposed compared with duloxetine non-exposed was OR 1.09 (95% CI 0.82-1.45); for SSRI exposed, 1.39 (95% CI 1.00-1.94); for venlafaxine exposed, 1.20 (95% CI 0.82-1.76); and for duloxetine discontinuers, 1.11 (95% CI 0.77-1.60).*

**18. Results: Rather than describing “Similar patterns were observed in the sensitivity analyses.” for the stillbirth and minor malformations analyses, please describe what was observed.**

The manuscript has been revised.

Copy of the revised text (changes are underlined):

*The sensitivity analyses for major malformation the OR and 95% CI also centered around 1 and suggested no increased risk (Supplementary material Table S7).*

*The sensitivity analyses for minor malformation also suggested no increased risk (Supplementary material Table S8).*

*Results for the major malformation subtypes are found in the supplementary material (both main and sensitivity analyses Table S9-S20).*

*The sensitivity analyses for stillbirth also suggested no increased risk (Supplementary material Table S21).*

**19. Results: “It is of note that a statistically significant increased risk of “other anomalies and syndromes” was found when duloxetine exposed women were compared with SSRI exposed. The PS-matched analyses yielded OR 2.43 (95% CI 1.10-5.38).” Please make it more clear that the PS-matched result presented here is for the SSRI-exposed comparison.**

The manuscript has been revised.

Copy of the revised text (changes are underlined):

*It is of note that a statistically significant increased risk of “other anomalies and syndromes” was found when duloxetine exposed women were compared with SSRI exposed (PS-matched analyses of duloxetine vs. SSRI: OR 2.43 [95% CI 1.10-5.38]).*

**20. Discussion: Please present and organize the Discussion as follows: a short, clear summary of the article's findings; what the study adds to existing research and where and why the results may differ from previous research; strengths and limitations of the study; implications and next steps for research, clinical practice, and/or public policy; one-paragraph conclusion.**

The paragraph is revised. We want to keep the text about generalizability, needed according to the STROBE guideline/checklist.

Copy of the revised text (changes are underlined):

*The study included all registered pregnancies resulting in a birth (live or stillbirth).* *We believe the results have a high external validity especially applicable to other western European countries with free and universal healthcare, where treatment regimens and population characteristics are comparable, as well as to the US, where indications and treatment guidelines are similar to the studied population. Despite the large cohort, the number of duloxetine exposed was limited, and future studies should focus on analyzing larger cohorts and additional safety outcomes (e.g. preterm birth, abortions and small for gestational age).* *Improved measurements of exposure, outcome and covariates* *could also yield more precise estimates. In addition, information on duloxetine indication and depression severity could add relevant information.*

**21. Discussion: Please clarify whether this refers to increased risk of malformation or stillbirth: “Like the present study, a small Swedish study analyzing the association between SNRI/NRIs (not including duloxetine) and stillbirth found no increased risk.”**

The manuscript has been revised.

Copy of the revised text (changes are underlined):

*A small Swedish study investigating SNRI/NRIs (not including duloxetine) found no increased risk* *for stillbirth*

**22. Discussion: We suggest replacing “compliance” with “adherence” when referring to recommended folic acid supplementation.**

The manuscript has been revised

Copy of the revised text (changes are underlined):

*poor adherence to folic acid supplementation during pregnancy*

**23. Discussion: Generalizability: In this paragraph, we suggest a broader discussion on clinical or policy implications and further directions based on the findings reported here.**

See our response to the Editors Request 20

**24. Disclosure of conflicts of interest: These can be removed from the main text of the manuscript, and please make sure all information is completely and accurately entered in to the manuscript submission metadata.**

This text is removed from the manuscript and entered into the manuscript submission metadata.

**25. References: Please use the "Vancouver" style for reference formatting, and see our website for other reference guidelines** <https://journals.plos.org/plosmedicine/s/submission-guidelines>

The references are updated

**26. Reference List: Reference 49: Papers cannot be listed in the reference list until they have been accepted for publication or are publicly available on a preprint archive. The information may be cited in the text as a personal communication with the author if the author provides written permission to be named. Alternatively please provide a different appropriate reference.**

We understand. We have removed the former reference 49 (Dalton 2008), and only kept reference 48 (Yang 2012). The current reference 48 (Yang 2012) also covers the former reference 49 (Dalton 2008).

**27. Reference 51: Please provide the complete citation information.**

The former reference 51 is removed and replace by the current reference 31 (Einarson 2012). The former reference 51 was an incomplete reference to the same publication as the current reference 31.

**28. Figure 1: Please define all abbreviations in the legend (SSRI, LMP).**

**29. Figure 2 and Figure 3: Please define all abbreviations in the legend (SSRI).**

Figure legend now include abbreviations.

**30. Table S5: Please move the table to the main section of the paper.**

We agree with this. Baseline table for stillbirth analyses is Table 2 in the revised manuscript. The table is inserted without track changes.

Copy of the revised text (changes are underlined):

*Table 1 and Table 2 show baseline characteristics for the analyses of malformation and stillbirth, respectively.*

**31. All tables: Please make sure to define all abbreviations used in the tables in the legends.**

Abbreviations have been defined in table legends

**Comments from the reviewers:**

**Reviewer #1: We thank the authors for addressing the previous issues raised, and have no further concerns.**

**Reviewer #2: Dear Editor and Authors,**

**my comment is regarding only the statistical methodology. I think that it has no sense to perform both the multivariate and ps matching adjustment, especially in this case where the prevalence of the outcomes are very rare.**

**The PS's methodology is, in fact, a methodology used to (i) avoid the overfitting problem (you should record at least 8 events for each confounder included in the model), and (ii) compare exposed and unexposed comparable.**

Based on the comment from the Editors (Request 1), we keep both the multivariable and the propensity-score matched analyses in the manuscript.

**Moreover, you should consider that if you do not require that all women have at least 5 years back-follow up available, the probability to find a confounder in women with 5 years vs those with fewer years is different. Since you considered chronic conditions and you assessed the concomitant medications only in the year before LMP I will recommend you to ask as inclusion criteria that all women have at least one year prior to the LMP and to evaluate the confounders in this time window.**

See our response to the Editors Request 1.
